# Hidden consequences of olfactory dysfunction: a patient report series

**DOI:** 10.1186/1472-6815-13-8

**Published:** 2013-07-23

**Authors:** Andreas Keller, Dolores Malaspina

**Affiliations:** 1 Laboratory of Neurogenetics and Behavior, Rockefeller University, New York, NY, USA; 2 Department of Psychiatry, New York University School of Medicine, New York, NY, USA; 3 Creedmoor Psychiatric Center, New York State Office of Mental Health, New York, NY, USA

**Keywords:** Olfaction, Quality of life, Anosmia, Phantosmia, Parosmia, Anhedonia

## Abstract

**Background:**

The negative consequences of olfactory dysfunction for the quality of life are not widely appreciated and the condition is therefore often ignored or trivialized.

**Methods:**

1,000 patients with olfactory dysfunction participated in an online study by submitting accounts of their subjective experiences of how they have been affected by their condition. In addition, they were given the chance to answer 43 specific questions about the consequences of their olfactory dysfunction.

**Results:**

Although there are less practical problems associated with impaired or distorted odor perception than with impairments in visual or auditory perception, many affected individuals report experiencing olfactory dysfunction as a debilitating condition. Smell loss-induced social isolation and smell loss-induced anhedonia can severely affect quality of life.

**Conclusions:**

Olfactory dysfunction is a serious condition for those affected by it and it deserves more attention from doctors who treat affected patients as well as from scientist who research treatment options.

## Background

Two recent patient memoires describe vividly the often unanticipated consequences of changes to one’s sense of smell from the patient’s perspective [1,2]. Olfactory perceptual changes can be quantitative (smell loss) or qualitative (smell distortions). Smell loss can be partial, a condition called hyposmia, or total, a condition called anosmia. Patients with partial smell loss often also suffer from distorted olfactory perception. Distorted olfactory perception can be subdivided into parosmia (distorted olfactory experiences in the presence of an odor) and phantosmia (distorted olfactory experience in the absence of an odor) [for overviews, see [3,4]]. Phantosmia and parosmia often co-occur [5] and parosmia is more common than phantosmia [5-8].

Olfactory dysfunction is a very common condition with a reported prevalence between 4 and 25% [9-12]. Men are more likely to suffer from it than women [13,14] and smoking [9-11,15-17], working in a factory environment [18], low level of education [19], and having a low household income [9] have been reported as risk factors. Olfactory dysfunction, like visual and auditory impairment, becomes more prevalent with increasing age [12,20]. Of those who suffer from smell loss, between 10 and 60% also have distorted olfactory perceptions [5,8,21,22]. Distorted perception is more common when the smell loss is less severe [22].

**Figure 1:**
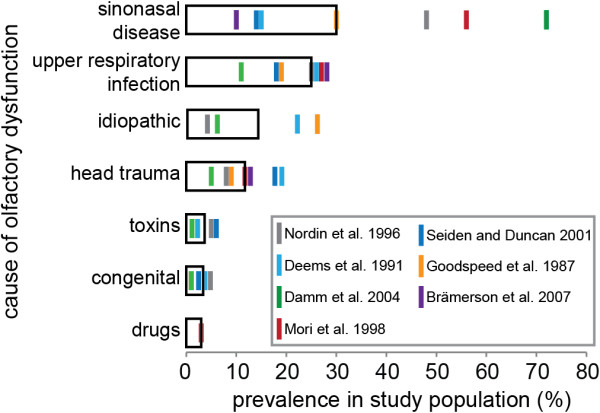
**Causes of olfactory dysfunction.** The relative prevalence of different causes of olfactory dysfunction as reported in seven studies [5,22,25-29] is shown. Median values are indicated by the open black bar. Sinonasal disease, upper respiratory infection, and head trauma are the three most frequent causes of olfactory dysfunction.

There are many causes of olfactory dysfunction [for an overview, see [23,24]]. The three most common causes are sinonasal disease, upper respiratory infection, and head trauma (Figure 1). Sinonasal diseases like nasal polyps or chronic inflammation of the nasal passages and/or paranasal sinuses (rhinitis, sinusitis, rhinosinusitis) are the most common cause of olfactory dysfunction (Figure 1) [for an overview see [30-32]]. Chronic inflammation in the nose and sinuses is the most common chronic medical condition in the United States of America [33-35] and more than half of the affected individuals have olfactory symptoms [36]. The cause of the olfactory problems in sinonasal diseases is in many cases nasal obstruction. The second most common cause of olfactory dysfunction are upper respiratory tract infections that result in permanent damage to the olfactory sensory system (Figure 1). As a consequence of the damage, smell loss will continue long after the infection and its other symptoms have subsided [for overviews, see [21,37,38]]. Patients with postviral olfactory loss often retain some smell capacity [22,25,39] and olfactory distortion is very common in these patients [8,21,40,41]. The third most common cause of olfactory dysfunction is head trauma (Figure 1) [for an overview, see [42-45]]. Head trauma often leads to very severe olfactory loss [41] with sudden onset. In addition to the three main causes of olfactory dysfunction, surgical procedures [46] (both sinonasal surgeries [47] and other types of surgery [48]) can affect olfactory function. Some of these cases are likely due to side-effects of the drugs used for general anesthesia [49-51]. Other drugs can also have side-effects on olfactory function [52-55]. Similarly, toxic chemicals in the environment can damage the olfactory system [56-60]. Finally, around 3% of those suffering from olfactory dysfunction have congenital anosmia: they were born without a sense of smell (Figure 1) [for an overview, see [61]]. In rare cases, olfactory perception can be disturbed due to processes in the central nervous system, as in epilepsy [62,63], migraine [64,65], Parkinson’s disease [66], or schizophrenia [63,67,68].

Not all cases of olfactory dysfunction are permanent. Partial spontaneous recovery has been reported especially in younger patients [69] and in patients with postviral olfactory dysfunction [69-71]. Remarkably, spontaneous recovery can occur years after the symptoms appeared [69,71-73], but the likelihood of recovery decreases with the duration of smell loss [22,69,70]. Olfactory dysfunction caused by sinonasal disease usually fluctuates over time [25] and can be modulated, for example, by physical exercise [26] and hot showers [25]. If olfactory impairment is a symptom of sinonasal disease, then treating the underlying disease will often improve olfactory function. Among the treatments of sinonasal disease that have been evaluated for their influence on olfactory function are antihistamines [74], nasally and systemically administered corticosteroids [25,75-79], and surgery [46,47,80-85]. For postviral and posttraumatic olfactory loss several treatments have been suggested [for an overview, see [54,86]]. Zinc [87,88], vitamin A [89], and the antibiotic minocycline [90] have been shown to be ineffective in placebo-controlled studies. α-lipoic acid [91] and the phosphodiesterase inhibitors theophylline [92,93] and pentoxifylline [94] have not been tested in placebo-controlled studies yet. Peroral caroverine, an N-methyl-D-aspartic acid (NMDA) receptor antagonist [88], as well as sodium citrate nasal spray [95] have been shown to be effective in placebo-controlled studies. In addition to drug treatments, acupuncture [96-99] and olfactory training [100,101] have also been investigated. It is likely that any successful treatment of smell loss would also improve the associated symptoms of distorted olfactory perception. However, some treatments, like the surgical excision of olfactory sensory neurons [3,102,103], bilateral olfactory nerve sections [104], and repetitive transcranial magnetic stimulation [105] have been specifically targeted at smell distortions.

None of the treatments that have been investigated are in wide use and in most cases olfactory dysfunction is untreatable. This is unfortunate because the disease burden of olfactory dysfunction is high [for an overview, see: [106-108]]. Quality of life in patients with olfactory impairments is reduced compared to matched controls [108] and patients in which the condition improves report a higher satisfaction with life than patients in which the dysfunction persists [109]. Practical problems of olfactory dysfunction include difficulties avoiding hazardous events [110] and the struggle to maintain healthy eating behaviors [70,111-113]. Without a sense of smell, natural gas leaks [109,110,114], fires [109,110,114,115], and hazardous chemical vapors [115] cannot be detected. Similarly, it is more difficult for these individuals to detect spoiled food [70,109,110,114]. In addition, food intake has been reported to be affected by olfactory dysfunction. Some patients report losing weight after losing their sense of smell, while others report gaining weight [112,113]. Weight gain is more common [112]. Both weight gain and weight loss seem to be a consequence of food being less enjoyable in the absence of olfactory input [70,112,115]. In most subjects, in addition to the change in how much food is consumed, olfactory impairment also induced a shift in food preferences. Taste and mechanosensation have to compensate for the lost olfactory input and as a consequence spicy food becomes more attractive [112,116,117].

The practical problems of not being able to sense the odorous environment are exacerbated by smell loss-induced social isolation. The social problems that the condition causes for relationships with friends, colleagues, family members, and romantic partners [27,118,119] are partially a consequence of social insecurity caused by worries about undetected body odor and partially a consequence of frustration over the perceived lack of sympathy for the patients [120]. Interactions with medical service providers can also be a source of frustration. One study showed that in Germany and Switzerland, 25% of patients felt that they had not been managed well and 6% felt that their condition had been trivialized [121].

In addition to practical and social consequences, olfactory loss also correlates with reduced ability to experience pleasure and motivation to engage in pleasurable activities: smell loss-induced anhedonia. Smell loss-induced anhedonia is the least-appreciated consequence of smell loss because affected individuals are often not aware of the connection between their olfactory dysfunction and the reduced enjoyment of formerly enjoyable activities. Although the mechanism is unknown, there is a correlation between smell loss and depressive symptoms and mood changes [22,70,115,118,120,122].

Distorted olfactory perception is even more detrimental to the quality of life than smell loss [3,4,22,123]. In one study, over half of the patients with distorted odor perception reported that their condition *severely* affected their quality of life [40]. Leopold and colleagues write about phantosmia patients that “it is usual for the patients to have thought about suicide because they had been offered no hope for resolution…” [102].

In this paper, the collected first-person accounts from 1,000 patients suffering from olfactory dysfunction confirm that severe consequences of this condition on life style and life satisfaction are common.

## Methods

Between 10/16/2009 and 08/08/2012, subjects submitted their experiences with olfactory dysfunction online under the IRB-approved protocol NYU-SoM 09–0226. One thousand subjects were selected for inclusion in this paper. For edited versions of the one thousand reports, see Additional file 1. The free-form reports were not analyzed quantitatively. Excerpts of the reports are used as examples. However, in addition to submitting the free-form reports, subjects were given the chance to complete a questionnaire and the responses to this questionnaire have been quantified. A 43-point questionnaire that asked questions about specific aspects of life that are known or suspected to be affected by a change in olfactory acuity has been used. The questionnaire was adapted from the one used by Frasnelli and Hummel [123]. The complete results of the questionnaire, which was completed by 725 of the 1,000 subjects are shown in Additional file 2.

**Figure 2:**
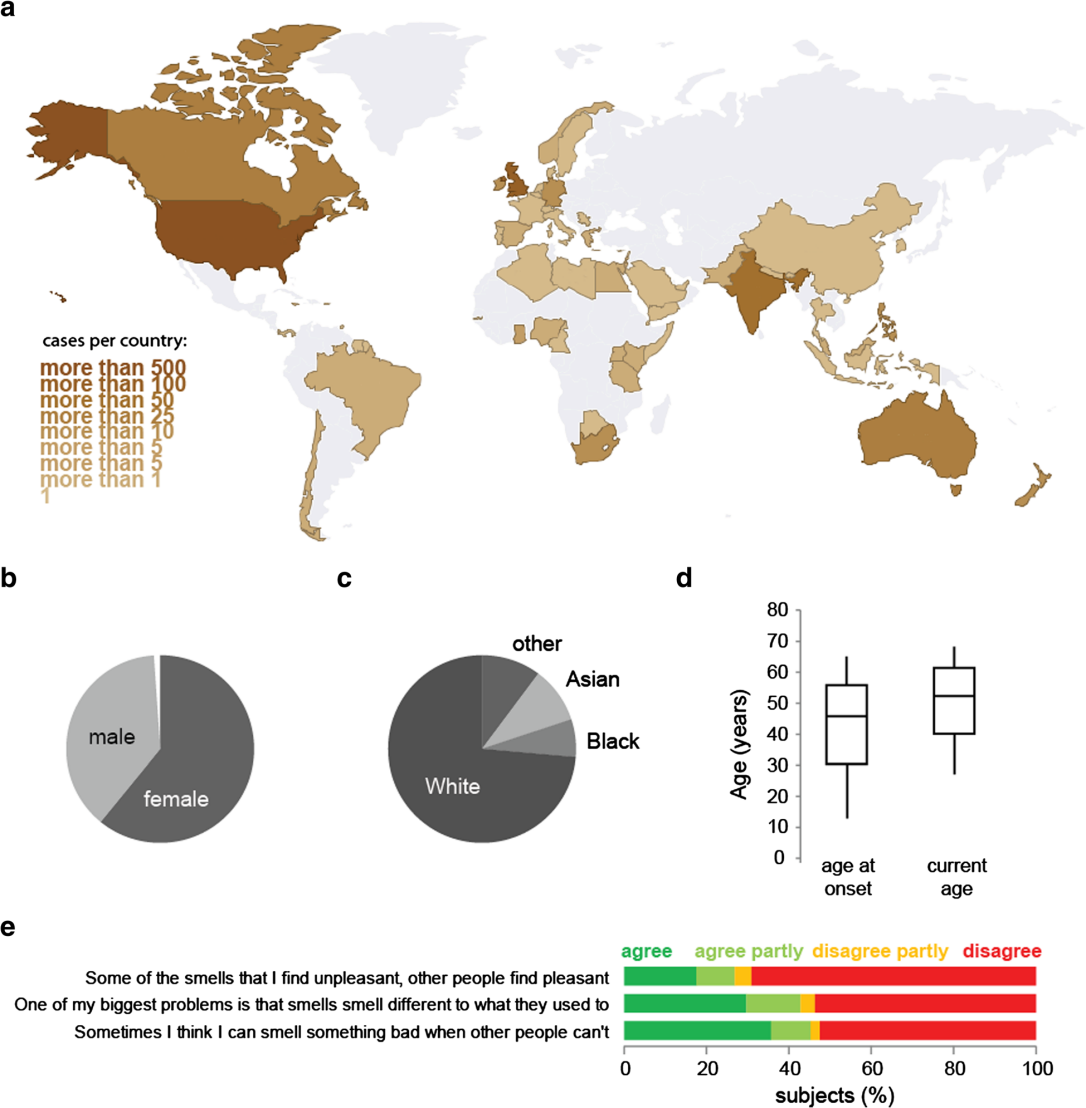
**Study population.** The subjects of this study are from 64 different countries with most from the USA, the UK, India, and Canada. **(a)** The gender **(b)**, self-reported race **(c)**, and age as well as age at onset of the olfactory dysfunction **(d)** of the subjects is shown. In **(e)** the responses to three of the questionnaire questions are shown.

All the participants in the study gave informed consent. Due to the fact that this was an anonymous online study, they consented by responding with “I agree” in response to the question “By submitting your story you give us the right to use your story for our research and to include it in research presentations and in publications”. The subjects were from 64 different countries. Because the website was in English, most reports were from English-speaking countries (Figure 2a). Sixty-two percent of the subjects who reported their gender were female and 38% male (Figure 2b). Almost three quarters self-identified as White or Caucasian (Figure 2c). The subjects range in the age at which they submitted their report from 6 to 85 with a median of 52. The median age of onset of the problems was 46, with a range from 0 to 83 (Figure 2d). Fifty-nine percent of the subjects had previously seen a doctor for their olfactory dysfunction (Additional file 2). A third to half of the subjects reported experiencing smell distortions in addition to smell loss (Figure 2e). For full methods see Additional file 3.

## Results and discussion

### Experiencing smell loss

Even patients with no sense of smell usually have some type of perceptual experience when they inhale high concentrations of volatile organic molecules [124]. In this study, subjects with olfactory loss report that “vapors feel differently” (subject 0002) or that smells coat their mouths (subject 0063) when there is a source of volatile chemicals nearby. Individuals with smell loss often experience their conditions as still being able to detect the presence or absence of smells, but being unable to identify smells or discriminate between different smells (subjects 0268, 0771, 0823 and 0831). Different metaphors and comparisons are employed to describe this experience. One subject (subject 0212) says that it is “like the form of a smell is there, but there really isn't a smell”, another subject (subject 0248) says that her “smell is now ‘black & white’ and no longer ‘in color’”. Smelling without a sense of smell is like eating chick peas that you can feel but not taste when you eat them (subject 0818). It’s like being an almost blind person who still can recognize silhouettes (subject 0723).

If the smell loss is caused by sinonasal disease it is often not due to permanent damage and the olfactory symptoms can therefore fluctuate over time [25] and can be modulated. Subjects experience improved olfactory function for example during exercise [26] (subjects 0003, 0013, 0160, 0586, 0691, 0763, and 0964). At least one subject used exercise to “switch-on” his sense of smell when desired:

During a dinner party when a good wine was served I would excuse myself from the room and run up and down the stairs several times. This restored my sense of smell for a period. (subject 0013)

Changes in altitude (and the resulting changes in air pressure) are also reported to have the effect of temporarily bringing back some olfactory perception in patients with sinonasal disease. Scuba diving (subject 0013), air travel (subject 0015), hiking in the mountains (subject 0964), or simply going to Colorado, which has a mean elevation of over 2,000 meters, (subject 0817), are all reported to improve odor detection in these patients. Hot showers have been reported to trigger this change [25] and the subjects of this study also report that changes in the temperature of the inhaled air can temporarily improve their olfactory acuity (subjects 0140, 0160, and 0586).

Because of the large contribution of the olfactory system to flavour perception, olfactory loss also dramatically changes the experience of eating food. Subjects compare their eating experience to eating sawdust (subject 0632), cardboard (subjects 0114, 0241, 0714 and 0912), or paper with glue (subjects 0004 and 0804). Coffee and other hot beverages taste like hot water (subject 0123). Subjects cannot distinguish cola from lemonade or cream soda (subject 0712), whiskey from rum (subject 0226), or coffee from tea (subject 0160). One subject reports that she noticed that she accidentally sprinkled paprika instead of cinnamon on her oatmeal only after she had finished eating (subject 0995).

### Practical problems of smell loss

The practical problems of not having a sense of smell can be grouped into three groups: problems with hazard avoidance, food-related problems, and problems with managing odors.

#### Hazard avoidance

72% of the subjects of this study are scared of getting exposed to dangers because of their olfactory dysfunction (Additional file 2). The main concern is the inability to detect a gas leak or a fire. Several subjects report that they have actually failed to detect a gas leak (subjects 0009, 0028, 0413, 0826, and 0985). Similarly, the inability to detect fires has resulted in dangerous situations for some subjects (subjects 0009, 0140, 0530, and 0531). For firefighters, olfactory loss and the resulting inability to locate fires through smell is a particular challenge (subjects 0139 and 0334). One subject (subject 0531) reports that, while cleaning the bathroom with a strong solvent that was odorless to him, he exposed himself to the volatile chemical until he was coughing up blood. Other subjects with reduced olfactory acuity also experienced adverse effects due to exposure to undetected volatile chemicals (subjects 0031, 0532 and 0913).

#### Food-related

**Figure 3:**
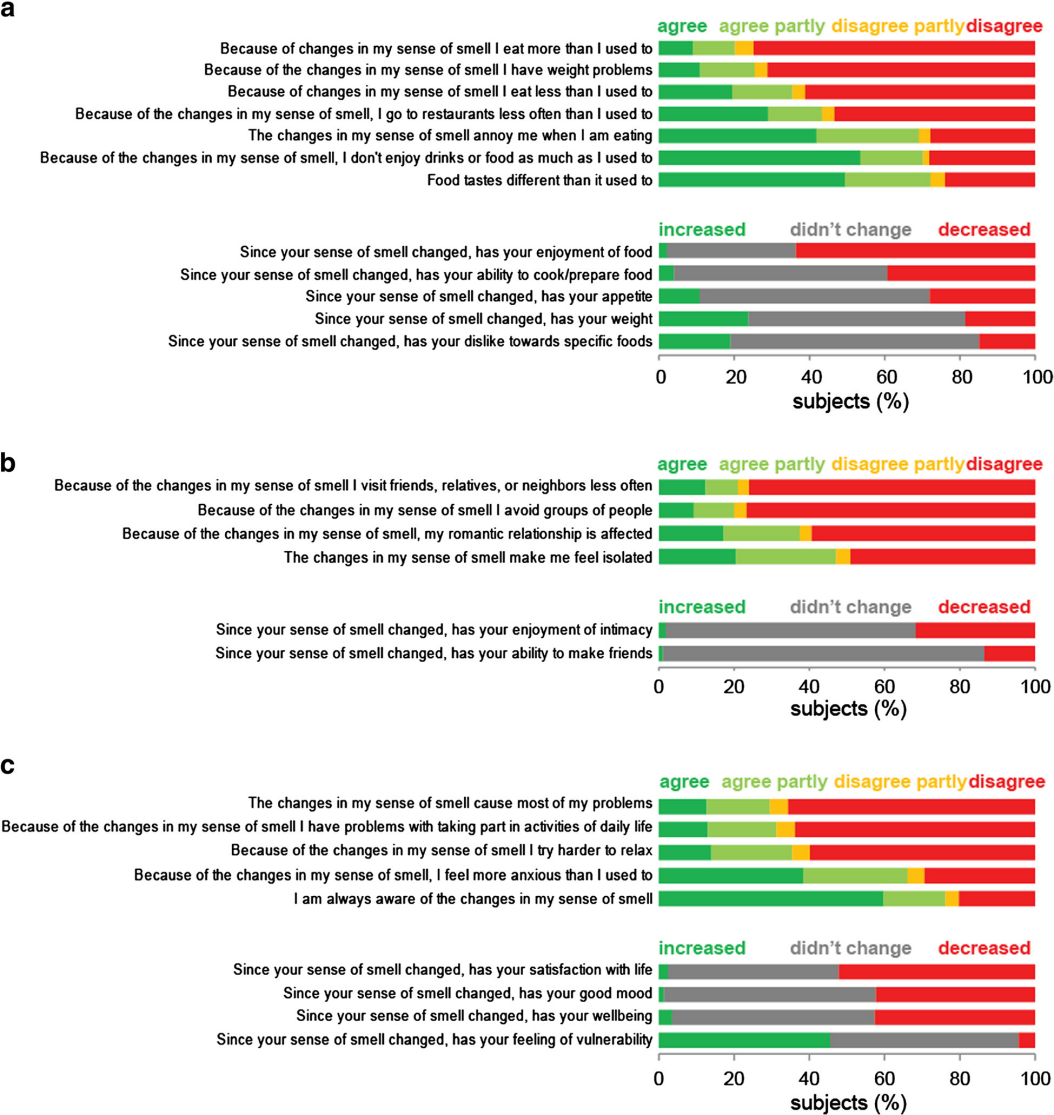
**Consequences of olfactory dysfunction.** Food-related consequences **(a)**, changes in social behaviors **(b)**, and general consequences **(c)** of changes in the sense of smell are shown. The results are based on the 725 subjects that have completed the questionnaire.

Many of the practical problems of not having a sense of smell have to do with food. Among the subjects of this study, accidentally eating spoiled food is common (subjects 0009, 0066, 0285, and 0637) and subjects report that they have become overly careful and tend to discard food when in doubt (subjects 0061 and 0319). Those who have been cooking before their smell loss often no longer cook or do not enjoy cooking anymore after they lost their sense of smell (subjects 0049, 0059, 0226, 0504, 0548, 0798, 0913, and 0930). Thirty-nine percent report that since the change in their sense of smell their ability to prepare food has decreased (Figure 3a). For those who work as chefs (subjects 0041, 0199, and 0287) or sommeliers (subject 0299) olfactory dysfunction makes it extremely difficult to continue their careers.

Probably the most striking consequence of smell loss for those affected is that they no longer enjoy eating. Sixty-four percent of the subjects report that their enjoyment of food has decreased (Figure 3a). Different subjects respond with different behaviors to the reduced hedonic value of food. Some lose the motivation to eat (subjects 0413 and 0981) and eat less (36%; Figure 3a). There are subjects reporting that they have to force themselves to eat (subjects 0421 and 0776) and a subject (subject 0875) who says “I now only eat to fill up and for no other reason”. Other comments include: “I just let myself get so hungry before I eat that the taste does not matter. It's just for fuel”. (subject 0284), “The only reason I eat now is to relieve hunger pains. I get no enjoyment from eating”. (subject 0049), “I don’t care anymore about eating, but I know I have to eat when I am hungry”. (subject 0548), and “I am only eating because I’m hungry, I don't enjoy any food”. (subject 0551)

These subjects respond to the reduced enjoyment they find in eating by eating less. However, other subjects (20%; Figure 3a) respond by eating more in an attempt to find the enjoyment that they remember:

I ended up gaining almost twenty pounds before realizing I was consuming more of every food in an effort to taste it. (subject 0004)

I think I’ve gained weight as a result of my loss of my sense of smell. I get the taste for certain foods, but my appetite isn’t satisfied because I cannot fully enjoy the foods I eat because I can’t smell them. (subject 0310)

I have gained a substantial amount of weight and I am wondering if it is because I am never fully satisfied as a person with normal smell and taste is. (subject 0327)

Instead of becoming disinterested in food, I find myself eating very spicy things, or sweet or sour, all in the interest of just having a sensation. But nothing much gets through except texture. I keep searching, and have even experienced weight gain since I’m ever looking for something… (subject 0560)

Some subjects will eat less and others will eat more after losing their sense of smell. As a consequence, some (19%; Figure 3a) report weight loss (subjects 0024, 0035, 0046, 0048, 0061, 0084, and 0086) while others (24%; Figure 3a) report gaining weight (subjects 0004, 0310, 0327, and 0560). This pattern has been observed before [112,113], but the factors that determine if an individual responds to food being less enjoyable by eating more or less are unexplored.

Losing their sense of smell not only influences how much affected individuals eat, but also what they eat. For 72% the taste of food changed as a consequence of the olfactory dysfunction and 19% report an increased dislike towards specific foods (Figure 3a). Many subjects of this study report that the role of texture became more important in deciding what to eat after they lost their sense of smell (subjects 0002, 0028, 0048, 0156, 0186, 0312, 0376, 0560, 0712, and 0723). Examples of textures that are sought out by these subjects are crunchy and crispy (subjects 0312 and 0723) and smooth and creamy (subjects 0004 and 0723). Irritants also can contribute to the flavour of food through non-olfactory pathways. Pungent irritants like horseradish (subjects 0028 and 0061), chili peppers (subjects 0028, 0061), and pepper (0606) are popular. Taste is the most obvious contributor to flavour in the absence of olfactory perception. The subjects in this study report that their loss of smell resulted in a preference for salty (subjects 0004, 0010, 0358, 0502, 0526, and 0723), sweet (subjects 0004, 0022, 0502, and 0723), and sour (subjects 0560 and 0723).

#### Odor management

For those who lost their sense of smell, odor management is also difficult. Without a sense of smell it is impossible to verify that one’s body, children, or home do smell acceptable:

However, I do get paranoid about personal hygiene, how my house smells — i.e. something gone rotten in the fridge, musty smells — this has resulted in me being absolutely fanatical about both personal hygiene and household cleaning — to the point where people close to me have asked if I suffer from obsessive-compulsive disorder. (subject 0532)

My poor kids sat in dirty diapers longer than they should have because I couldn’t smell the soiled diaper. […] I’ve put on way too much perfume many, many, many times. I shower every day because I’m nervous I might have body odor. (subject 0354)

The most important odor to manage is one’s body odor. There are severe social consequences of failing to maintain the culturally expected body odor and many individuals who suffer from smell loss therefore are worried about their olfactory appearance. A large proportion of individuals with a diminished sense of smell complain about the difficulties of perceiving their own body odor and the resulting challenges for personal hygiene [70,115]. Of the subjects in this study, 72% report a change in the perception of their own body odor (Additional file 2). Managing their own body odor without being able to perceive it is challenging (subjects 0004, 0014, 0025, 0087, 0186, 0310, 0335, 0531, 0550, 0589, 0627, 0823, 0829, 0925). The obvious strategy is a rigid personal hygiene regime and to have others help with the assessment of one’s body odors (subjects 0965 and 0984).

Another challenge for those with smell loss, especially for elderly people who live by themselves, is to manage the odor of their homes (subjects 0087, 0310, and 0756):

I lost my sense of smell for no apparent reason five years ago at age 72. I never noticed it until my daughter said my house had a terrible odor and we then discovered a dead rodent that caused the odor. (subject 0449)

Other subjects also report that they could not perceive the foul smell of decay filling their house after an animal died underneath furniture (subjects 0637 and 0874). Living in a house filled with a foul smell can be very embarrassing and the fear of this embarrassment can lead to social isolation when the affected person refuses to let people in their house (subject 0756).

Keeping pets also poses problems for those without a sense of smell:

[As a child] I was told I could have a pet cat as long as I took care of it and one of those responsibilities was to change the cat box as soon as I smelled it. Needless to say I was in constant trouble and was thought to be irresponsible and lazy. Eventually I was able to convince others that I just couldn’t smell. (subject 0014)

… just recently one of our cats urinated on a piece of carpet, and it apparently reeked, and the smell was making my boyfriend nuts, and I couldn’t smell it at all. His reaction to me was complete disbelief, as if I was faking that I couldn’t smell something horrid. (subject 0666)

Other subjects report having not noticed that a dog relieved itself on the floor (subjects 0087 and 0539).

Young children also need olfactory attention. Diapers need to be changed when they are “stinky” (subjects 0004, 0285, 0332, and 0747). Child care is a sensitive subject and parenting without an olfactory system can be anxiety inducing:

Another embarrassing thing I’ve run into was when my last two children were babies — I wouldn’t notice that they had a dirty diaper, of course. […] I remember two different mothers who treated me with great disgust, as if I didn’t care about my child or hygiene, almost as if I were abusive. I had another horrible experience when I apparently left a load of laundry in the washing machine for too long. The head of the day camp where I was sending my daughter took me aside on the third day of the week and asked why I was sending my daughter to camp in clothing that smelled of mildew […] It’s a source of enormous anxiety for me. (subject 0004)

People whose jobs involve odor management like nurses (subjects 0705 and 0782) or building managers (subject 0012) face problems in their professional lives after losing their sense of smell.

In summary, there are several practical challenges of having an olfactory impairment. Problem-focused coping strategies are available for affected individuals [115,118,119]. Fire detectors can ease anxiety about undetected fires. There are recipes designed specifically to be enjoyable for those with a reduced sense of smell [125]. Perhaps most importantly, affected individuals can learn to rely on others noses and trust their judgment. However, this social dimension of olfactory impairments causes its own set of problems, which will be discussed in the next section.

### Smell loss-induced social isolation

The subjects of this study report that many of the problems that are associated with living with an olfactory dysfunction have to do with the responses by friends and family-members to the condition. It seems to be common to doubt the existence of olfactory dysfunction or to trivialize the condition. This often leads to embarrassment, alienation, anger, and withdrawal on the side of the patient. Half of the subjects report that their condition makes them feel angry (Additional file 2) and 47% report that it makes them feel isolated (Figure 3b).

Affected individuals have to continuously explain their condition and defend themselves against explicit or implicit suggestions that they are lying about their inability to smell (subjects 0008, 0272, 0666, and 0928). For those with congenital olfactory impairment the challenge starts with convincing their parents and other adults that they cannot smell (subjects 0009 and 0071). Children with congenital smell loss are usually unaware of the dysfunction and only “discover” their condition as teenagers [70,126]. One subject reports her experience when she was six years old and came home from school where cinnamon rolls were baked, wondering what this “smell” everybody else got so excited about was:

My mother got surprised, because she had absolutely no clue about this condition before that. We went to the hospital to check it out, but with little result. I was asked to smell several different things while being blindfolded, and I couldn’t smell anything. The result was however that I was a stubborn child who lied, so not much more was done. (subject 0071)

For those who lose their sense of smell as adults it is equally challenging to convince people of their condition:

I get the feeling that people think I am lying about my sense of smell a lot; especially once they see me enjoy food. That’s pretty aggravating. (subject 0020)

By many, including the patients themselves, olfactory impairment is considered to be “strange” (subjects 0078 and 0172), not “normal” (subjects 0048 and 0686), and “weird” (subjects 0005, 0078, 0729, and 0982), Those affected are labeled “freaks” (subjects 0008 and 0580) or “crazy” (subjects 0078, 0094, 0172, 0340, 0359, 0447, 0470, and 0729).

Once affected individuals have convinced others of the existence of their condition, they often face a lack of sympathy. Olfactory impairment is not considered to be a serious disability (subjects 0063 and 0072) and sometimes affected individuals are even told that they should be happy about their inability to smell unpleasant odors (subjects 0025 and 0048):

It's a weird affliction. People don't really get it. They think it’s not as big a deal as it is. After all, they figure anosmics aren’t disabled. We don't need seeing-eye dogs or sign language to interact with our environment. And they are right — partly. We can function without drawing attention to our plight. We can do virtually everything we could before we lost our sense of smell, except enjoy the immensely important aspects of human life that most people take for granted. (subject 0005)

I have written at some length here because there seems to be a total lack of interest in the very distressing condition of anosmia — most people dismiss it as a joke: "aren't you lucky you can change babies' nappies without noticing", whereas if I had gone blind or become deaf everyone would be sympathetic. I have learned not to mention it anymore and work my way around it without letting anyone know. (subject 0025)

A symptom of the lack of sympathy for affected individuals is that others regularly keep forgetting about the condition (subjects 0008, 0029, 0063, 0292, 0589, and 0925) and are unforgiving about problems caused by it (subjects 0014, 0539, 0666, and 0897):

Life can be hell sometimes but no one seems to take it seriously. It is a disablement that is invisible. People are always saying “smell that”, “taste this”. It is very annoying; you wouldn't tell a blind man to look at the lovely scenery. (subject 0029)

If you're blind, people forgive you if you are wearing mismatched socks, but they can't see if you have anosmia and therefore a reason why you may have undetected body odor. (subject 0014)

…one day someone aggressively accused me of ignoring the burning meal on the stove on purpose. (subject 0897)

I was watching TV, and my dog pooped right behind my chair, and I didn't notice until my mother came down and yelled at me for not picking it up. (subject 0539)

It is especially aggravating for the patients when members of the medical profession to which they turn for help trivialize their condition:

My doctor did not know such a symptom existed. He was stunned that this could happen and stuck a couple things like coffee under my nose to test me. […] When he found out it had a name, anosmia, he looked up possible causes in their computer and decided to send me to get an MRI and a CT scan. He refused to send me to an ear nose throat specialist because “I cannot send you unless I first diagnose a condition they can treat, like sinusitis”. (subject 0012)

I don't care as much ultimately about what the public awareness of this condition is; I'm resigned to anosmia being a joke for those who don't have it. I do wish that doctors took it more seriously. I have talked to too many doctors who did not believe that I cannot smell. […] One especially ignorant fellow just didn't believe that I'm unable to smell anything at all and treated me as if I were some hysterical female, telling me it was entirely psychosomatic. This needs to change, and this is why I've just spent the last 45 minutes pouring all this out for you. (subject 0004)

My doctor first said “You're lucky! You won't have to smell the diapers!” When that upset me, he replied that loss of the sense of smell was “no big deal” and I would “probably get it back in a few weeks”. That was more than a month ago and there has been no improvement. It is more terrible than I could have imagined… (subject 0079)

Other subjects also report disappointing interactions with their doctors (subjects 0030, 0035, 0054, 0073, 0080, 0121, 0133, 0196, 0238, 0267, 0271, 0395, 0402, 0428, 0571, 0897, and 0942). Doctors told them that olfactory dysfunction is “a good thing to have” (subject 0423), that it “is not a sickness” (subject 0001), “just a psychological feeling” (subject 0043), or they treated the patient's complaint as “a trivial matter” (subject 0019). These doctors are a small minority and most doctors handle complaints of olfactory impairments professionally and show compassion towards their patients. Much of the patients' frustration is caused by the lack of treatment options. However, the fact that there are some doctors who never heard of a chronic condition that affects a large percentage of the population is indicative of a problem.

The affected individuals' response to the perception that their condition is “weird” and a trivial matter is that they lie about their condition to avoid having to discuss it. Children who do not have a sense of smell often just mimic others' reactions to smell without actually perceiving any smells (subjects 0009, 0031, 0064, 0304, 0308, 0319, 0532, 0611, and 0686):

Smelling seemed to me like religion, you just had to have enough faith to make it true. (subject 0002)

When I was little I used to pretend that I was able to because I thought I had to be able to “learn” how and I just wasn't good enough at it yet. (subject 0067)

I had always figured a sense of smell was something that developed as you got older. (subject 0077)

Adults often continue to pretend having a sense of smell to avoid having to discuss their impairment with often skeptical and unsympathetic people (subjects 0002, 0186, 0292, 0666, 0883, and 0925) or because they are embarrassed about it and fear to be labeled (subjects 0021, 0064, 0074, 0172, and 0319).

It was bad when I would eat with other people. They always ask about my food or my family would ask me to taste something and I would have to explain again that I still can't taste… It seemed that in some cases my loss of taste and smell took away their pleasures, so I just started lying if someone asked me how my dinner was. (subject 0292)

… people keep asking me how I like their new perfume. Sometimes I just lie because telling my story makes me feel even worse and I don't want pity, I don't want to respond to the same questions over and over again, and I don't want the questioning looks in people's eyes starring in disbelief. (subject 0925)

The subjects of this study often comment that most people do not understand how it feels to live without a sense of smell (subjects 0005, 0008, 0023, 0027, 0137, 0530, and 0666). This perceived lack of understanding by others leads to social isolation of the affected individuals. They feel alone (subject 0102), left out (subject 0071), apart from the rest of us (subject 0030), or like outsiders (subject 0925). They are less interested in social situations (subjects 0014, 0125 and 0431), have reduced libido (subjects 0061, 0199, 0275, and 0655), and wonder if their problems with forming emotional attachments and establishing long-term romantic relationships may be due to their condition (subjects 0317 and 0912). Not being able to smell other people's odors can make it more difficult to feel close to them (subjects 0025 and 0544). Twenty and twenty-one percent of the subjects, respectively, report that since their change in olfactory perception they avoid groups of people and visit friends or relatives less frequently (Figure 3b). Thirty-eight percent report that it has affected their romantic relationship and 32% report a decreased enjoyment of intimacy (Figure 3b).

In this social isolation, affected individuals are often relieved when they find out that there are others with the same problems (subjects 0040, 0149, 0293, 0471, and 0592):

I looked it up online and found several stories about people smelling smoke just like me. […] it was nice to know that I'm not alone. (subject 0470)

I eventually met a number of other people who lost their smell after a head injury. It didn't make us friends but it helps knowing they're out there. (subject 0020)

I have not found anyone else who has this problem and have not been able to talk about it with others. It's good to write this at least. Thanks for the opportunity. (subject 0010)

The social problems that are associated with olfactory dysfunction would be in large parts avoidable if the condition and its consequences would be more widely known:

My greatest wish (obviously right after being able to smell) is that society will know more about anosmia and that they are aware of us and that there is a broader support for affected people. (subject 0925)

### Smell loss-induced anhedonia

In addition to the practical problems associated with olfactory impairment and the social consequences, not having a sense of smell can also result in anhedonia, the inability to experience pleasure from activities usually found enjoyable:

I have a two year old daughter and I've never been able to smell her. I miss the smell of pickles, early September mornings, the ocean, gasoline, matches and garlic… (subject 0008)

The sad thing, I find, is not being able to appreciate the everyday smells which we take for granted: perfume, freshly mown grass, freshly baked bread, scent of bluebells/roses/flowers in general. Living by the sea, I used to love the smell of the seaweed around the tide pools. The list is endless. (subject 0028)

My life is far less rich and my enjoyment of things (food, going to the seaside, pretty much anything) is often greatly reduced. (subject 0100)

It's made me quite sad at times as I can't get excited about a new restaurant, the smell of summer, or any other smell that brings out an emotive response. (subject 0147)

Over 40% of the affected individuals report decreased wellbeing, mood, and satisfaction with life (Figure 3c). Sixty-six percent of the subjects in this study feel more anxious than before the change in their sense of smell and 46% feel more vulnerable (Figure 3c).

One important function of smell is contributing to the experience of places. Although we are often not aware of the smells of different locations, those smells greatly contribute to our experience and enjoyment of them:

It was extremely depressing to have no sense of smell at all. You realize that rooms have smells, water has a taste, etc. (subject 0036)

It improved slightly and I forgot about it until yesterday when realized I really missed the subtle spring scents, like bluebells, grass. (subject 0701).

I went to the seaside two years ago, but was unable to smell the sea and I felt quite sad about this. Also, on rainy days, I am unable to smell the damp earth or freshly cut grass… (subject 0510)

Many subjects of this study report that they miss characteristic nature smells, like the smells of flowers (subjects 0028, 0051, 0063, 0087, 0097, 0137, 0421, 0450, 0456, 0572, 0651, 0671, 0695, 0743, 0757, and 0984), grass (subjects 0058, 0587, 0701, 0705, and 0889), the forest (subject 0005), or the ocean (subjects 0008, 0028, 0058, 0100, 0441, 0510, and 0651).

Certain times or events also have characteristic smells. The subjects of this study complain about no longer being able to smell the smell of early morning (subject 0058), rain (subject 0450), an approaching snow storm (subject 0049), spring (subject 0701), summer (subject 0147), and fall (subject 0049). Similarly, holiday seasons have distinctive smells and subjects report missing the smells of Thanksgiving (subjects 0005 and 0888) and Christmas (subjects 0243, 0441, 0888, 0950, and 0998). The strong emotional effect that the characteristic smell of summer or Christmas has is partially mediated by memory. Smells are powerful elicitors of vivid personal memories [127-129] and those without a sense of smell miss these memories (subjects 0005, 0175, 0913, 0998, and 0999).

In addition to places, times, and events, people also have characteristic smells. Many subjects in this study note that they cannot smell their babies or children (subjects 0004, 0008, 0035, 0041, 0063, 0097, 0119, 0131, 0327, 0355, 0538, 0651, 0695, 0889, and 0925). Others complain about not being able to smell their romantic partner (subjects 0035, 0041, 0087, 0137, and 0912) and wonder if their olfactory impairment influences their romantic relationships (subject 0912):

I have become afraid: does my lack of sense of smell keep me from finding someone I'd like to spend the rest of my life with? (subject 0317)

Anhedonia can be subdivided into consummatory anhedonia, the inability to enjoy an activity, and motivational anhedonia, the lack of the desire to engage in enjoyable activities [130]. Motivational anhedonia can have a large negative impact on the lives of individuals suffering from smell loss. The smell of food does not only make eating food more enjoyable, it also motivates us to eat, to cook, and to go to restaurants. The smell of a romantic partner is not only a pleasant sensory experience, more importantly it motivates us to engage with him or her. An effect of smell-loss on motivation could explain the unexpectedly large life-changes experienced by patients with smell loss. The way some subjects in this study describe their experience of having no sense of smell is consistent with motivational anhedonia. They describe not having a sense of smell as dampening the colors of the world (subject 0175). The world without smells is described as “artificial” (subject 0019) and empty, like “living in a box and looking out at the world” (subject 0082). The world becomes less rich (subjects 0082 and 0100) and “smaller, darker, and sad” (subject 0925). Life becomes strange and depressing (subject 0930):

At first I felt very out of touch with myself — like I was out of step or like I had constantly forgotten something important. (subject 0606)

It is very difficult for me now to make plans, feel desire, feel good and happy. I live in a permanent present, I have lost the sensations linked to memories, I have no particular desire for the future.... (subject 0999)

### Experiencing smell distortions

Between 10 and 60% of individuals with partial or complete smell loss also experience distorted smells [5,8,21,22]. The experiences of these subjects and the problems that they face are different from those of individuals with smell loss.

#### Quality of distorted smells

The distorted smells experienced by those with parosmia or phantosmia have been described as “burned”, “foul”, “rotten”, “fecal”, “chemical” [6]; “burned”, “foul”, “unpleasant”, “spoiled”, “rotten” [3]; “off”, “rotten”, “burnt” [5]; and “burned”, “foul”, “rotten”, “sewage”, “chemically” [102]. Some patients do not have any associations with the distorted smell, describing it as an unpleasant unknown odor [6]. Not all distorted odor perception is unpleasant, though [131]. It is useful to follow Leopold [3] and differentiate between two types of smell distortion. In the more common type every distorted perception is the same (for example the perception of cigarette smoke), regardless of the trigger. In the second type different triggers cause different perceptions.

Describing odors and identifying them out of their usual context is notoriously difficult [132] and this is also true for distorted smells. Regardless, many subjects attempt to describe their distorted odor perceptions:

It's not good or bad, sweet or bitter, it's not chemical or organic, it's just there. (subject 0888)

… a constant odor — not sweet, not sharp, not foul. I had never smelled it before. (subject 0017)

I cannot identify the smell; I try to associate it with something but I come up empty. (subject 0026)

Many subjects in this study label the distorted odor they perceive as “strange” (subjects 0028, 0097, 0221, 0225, 0416, 0448, 0465, 0500, 0535, 0605, 0630, 0687, 0694, 0767, 0773, 0791, 0887, and 0962) or “weird” (subjects 0028, 0060, 0097, 0164, 0427, 0470, 0752, 0785, 0916, and 0963). Based on these reports, it is tempting to speculate that the distorted smell is the consequence of random firing of neurons, similar to the smell of complex mixtures of odors with the same intensity that has been named “olfactory white” [133]. This would be analogous to white noise or the hissing sound perceived by those suffering from tinnitus [134,135]. However, many subjects who experience distorted smells describe them as readily identifiable specific odors (see below).

The distorted perception is rarely pleasant and frequently described as “unpleasant” (subjects 0011, 0018, 0025, 0040, 0041, 0100, 0164, 0169, 0172, 0182, 0260, 0381, 0383, 0509, 0544, 0606, 0694, 0814, 0825, and 0933), “foul” (subjects 0136, 0216, 0475, 0511, 0670, 0674, 0745, and 0968), or simply “bad” (subjects 0027, 0052, 0068, 0084, 0097, 0114, 0132, 0164, 0210, 0235, 0295, 0339, 0351, 0439, 0441, 0451, 0525, 0651, 0730, 0774, 0803, 0815, 0840, 0854, 0860, 0872, 0887, 0895, 0900, and 0952).

Rarely, the olfactory distortion inverts the olfactory pleasantness spectrum; pleasant smells become unpleasant and unpleasant smells become more pleasant:

Smells that I did not like before suddenly seemed pleasant to me, and some of my favorite smells from before were generally less appealing to me. (subject 0085)

The perfumes I used to use, which smelled very pleasant to me prior to anosmia, now don't smell so pleasant. Some foods don't taste as good as they used to (for example I can't stand bananas now), and other foods taste much better than they used to. Cigarette smoke smells almost minty to me, and not nearly as unpleasant as it used to. (subject 0869)

**Figure 4:**
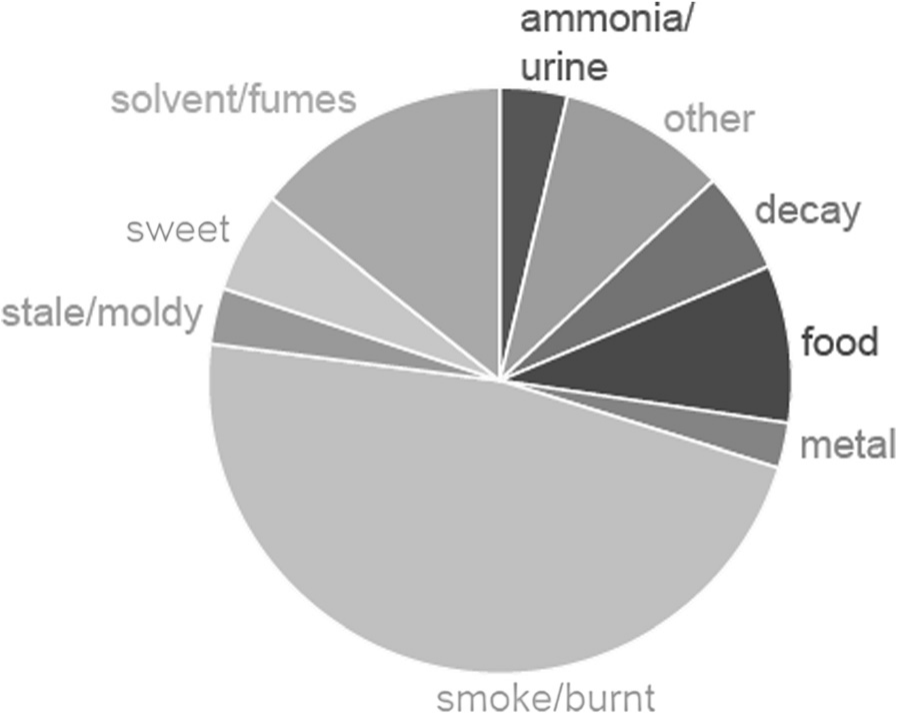
**Perceived quality of distorted smells.** Of the 161 subjects that reported quality of distorted smells, the largest group experienced a burnt or smoky smell. The second largest group reported that they experience the distorted smells as smelling like solvent or fumes.

Among those who attempt to describe or identify the odor, in almost half of the cases, the odor is associated with fire. “Cigarette smoke” is the most common description used by the subjects in this study. “Car exhaust” is also a common association and some subjects are reminded of burning rubber or electrical fire. One subject describes the experience as being “stuck behind a school bus” (subject 0306). Other subject describe the distorted perception as “burnt vegetables in a fetid swamp” (subject 0832), or as “like chili that has burnt to the bottom of the pan or a coffee pot that's been on the burner all day” (subject 0592). There are also other odor categories that are commonly used to describe the distorted smell (Figure 4). Many subjects are reminded of the smell of solvents or fumes from volatile chemicals by their olfactory distortions. Diesel fumes, paint smell, and plastic smells are often mentioned in this category. The next largest category is food smells, in which a variety of different and diverse experiences from “part cooking oil, part garlic, part fish oil” (subject 0235) to “oranges and cloves mixed with mulled wine” (subject 0066) are grouped.

Other common experiences are the odors of decay, like rotten meat (subjects 0126 and 0619) or “rotten peanuts soaked in vinegar” (subject 0674) and the smell of urine/ammonia (subjects 0036, 0197, 0278, 0340, 0359, 0396, and 0998), the smell (or taste) of metal (subjects 0062, 0098, 0177, 0448, 0500, 0525, 0713, 0773, 0787, and 0906), and sweet smell (subjects 0034, 0055, 0152, 0225, 0274, 0483, 0534, 0563, and 0721). There are also idiosyncratic olfactory experiences that are only mentioned by one subject each, for example blood smell (subject 0603) and lavender (subject 0934).

For most subjects, the distorted experience is always the same. However, as has been reported previously [3], for some subjects the distorted experiences can have different perceptual qualities:

I started to get phantom smells which ranged from the smell of the tumble dryer to oil and petrol. […] Many other smells like peppers, celery, and perfumes also smell different than they used to smell. Eggs and certain other foods smell like urine. (subject 0895)

The odors range from floral/perfume, wood or paper smoke, petroleum or solvent based (petrol, WD-40, butane) to cooked foods. (subject 0781)

At times it is like the room is filled with cigarette smoke so full it burns the back of my throat. […] Other odor variations are cherry pipe tobacco, the smell of brick being cut with a brick saw, jasmine, a smell so sweet it makes me sick. All body washes and shampoos make me sick; they smell like poop. (subject 0007)

Interestingly, several subjects of this study report that the distorted smell is similar to the last actual smell that they experienced; they are “stuck” with the last smell they smelled before losing their sense of smell (subjects 0078, 0116, 0150, 0173, 0225, 0234, and 0379). One subject lost her sense of smell in a car accident and now experiences an odor reminiscent of “the odor of the gray smoky powder that filled the car when the airbags deployed” (subject 0060). Other subjects report to continuously experience the lavender smell encountered on vacation (subject 0934) or the smell of caramelized sugar experienced at a friend's house while they were making fudge (subject 0065). For one subject the distorted smell alternated between two of the last smells she experienced, a sandwich served on a plane and the smell of guano from visiting a penguin colony (subject 0112).

Many subjects of this study report that their distorted experiences are not purely olfactory. The distorted “smells” are often accompanied by burning eyes (subjects 0047, 0106, 0172, 0203, 0263, 0391, 0397, 0451, 0545, and 0734), an irritated throat (subjects 0007, 0047, 0194, 0203, 0358, 0391, 0397, 0408, 0451, 0545, 0694, and 0739), and pain or burning sensation in the nose (subjects 0097, 0243, 0372, and 0860). These are experiences that cannot be mediated by the olfactory sensory system, but instead are likely to be a consequence of trigeminal nerve activity [136,137]. Furthermore, the “smells” elicit responses that are typical for trigeminal activation like sneezing (subjects 0127 and 0397) and nose rubbing (subject 0696). Subjects also report being woken up by the sensation (subjects 0178, 0412, and 0678), although actual olfactory sensations do not have the potential to wake subjects [138], and they locate the “smell” only in one nostril, which is also possible only for trigeminal stimuli but not for olfactory stimuli [139].

#### Triggers of distorted smells

In most cases of distorted smell perception a trigger for the experience can be identified. Distorted perceptions can be triggered by mechanical stimuli like sneezing [3] or changes in nasal airflow [102]:

The episodes are triggered by coughing, shouting, and sneezing. […] Introduction of water into the nasal passages/sinus cavity (underwater swimming, saline nasal rinse, etc.) can also trigger an episode. (subject 0302)

Hot or cold air can also trigger distorted olfactory experiences. Sensations can be triggered by furnaces (subjects 0083, 0118, 0136, and 0503), the hot air inside a car that sat in the sun (subject 0766), hot weather (subject 0555), heat from a blow dryer (subject 0084) or the interior of fridges and freezers (subjects 0084 and 0321).

By far the most frequently mentioned trigger of distorted olfactory experiences among the subjects of this study are volatile chemicals (like perfumes and scented beauty products) (subjects 0007, 0040, 0097, 0136, 0164, 0199, 0249, 0274, 0322, 0326, 0370, 0388, 0397, 0441, 0465, 0468, 0475, 0576, 0605, 0774, 0825, 0869, 0916, 0936, and 0941) and food or beverages, multisensory stimuli that include volatile chemicals (subjects 0007, 0011, 0062, 0249, 0284, 0322, 0326, 0441, 0451, 0465, 0468, 0475, 0501, 0513, 0605, 0774, 0825, 0837, 0840, 0847, 0855, 0858, 0869, 0911, and 0951). It has been shown that different stimuli have different potential to trigger distorted perception. In a survey of 46 patients, the most common stimuli eliciting distorted olfaction were gasoline (30%), tobacco (28%), coffee (28%), perfume (22%), fruits (15%, mainly citrus fruits and melon), and chocolate (13%) [40]. The subjects of this study also report differences in the effectiveness of potential triggers of distorted olfactory perception:

Within a few weeks the weird taste got stronger and started to affect more foods, including chocolate, yogurt, cottage cheese, fried foods, onions, green peppers, pancake syrup, beer, wine, grape fruit juice, and most snack foods such as chips (potato, corn, tortilla), crackers, pretzels, plus cakes and candies of all kinds. I could not even eat my birthday cake. Fruits of all kinds are at least a little bit weird with the taste becoming worse when the fruits become overripe. Water, skim milk, eggs, honey, and some cheeses taste normal. (subject 0164)

I could not tolerate certain smells that are usually pleasant, for example coffee, cut grass, celery (absolutely the worst), butter (especially buttered popcorn), apples, peaches, cucumbers, melons, perfumes, shampoos, soap, grilled meats, and poultry. I also discovered that I could no longer stomach certain related tastes, such as vegetable juice, carbonated beverages — especially colas, orange juice, red wines, anything melony. (subject 0027)

Some of the distorted sensations triggered by food are almost certainly triggered by heat activating the trigeminal nerve:

Especially when food was hot it smelled so very bad. (subject 0295)

I can smell the steam from ramen. (subject 0766)

Coffee, which is usually consumed hot, also appears to be an efficient trigger (subjects 0027, 0129, 0210, 0329, 0343, 0381, 0416, 0484, 0598, 0606, 0632, 0887, 0888, 0895, and 0911). Coffee is often mentioned together with chocolate (subjects 0164, 0445, 0632, 0855, and 0888):

Coffee and chocolate taste horrible!! (subject 0343)

I woke up one morning and immediately I could not stand the following smells and tastes: coffee, chocolates, all meat except processed meats like salami, peaches, watermelons, etc. (subject 0911)

… coffee and chocolate smelling just awful… (subject 0329)

I started to smell coffee but not like I used to smell it. Within weeks I was getting the same smell for cigarette smoke. I still get these smells — also for chocolate. (subject 0895)

I have never been able to smell or eat chocolate and coffee while I have the smell/taste because they are just too bad to handle. (subject 0129)

Citrus fruits like lemons (subject 0900), orange juice (subjects 0027, and 0210), citrus odors (subject 0095 and 0636), and orange peel (subject 0210) are also mentioned here as potent triggers. Melons are also often mentioned (subject 0027, 0312, 0349, 0381, and 0911), whereas other fruits appear to be less potent triggers of distorted smells (pineapple, kiwi fruit, and pears are never mentioned; strawberry and banana are mentioned by one subject each and apples by two subjects). Other foods that are indicated to be triggers by several subjects are peanuts or peanut butter (subjects 0036, 0381, 0416, and 0468), fried food (0226, 0588, and 0951), and onions (subjects 0226, 0349, 0416, 0568, and 0840).

### Problems of smell distortions

Individuals with a distorted smell perception face most of the problems that those with smell loss face. It is difficult to manage odors or to avoid hazards based on odors when the perception of odors is not veridical. However, olfactory distortions have a much stronger effect on the patients' quality of life than loss of smell [40,123]. There are multiple reasons for the severity of the effect of olfactory distortions. One problem seems to be that the distorted olfactory experiences provide a constant reminder of the condition for the affected individual, thereby drawing attention to the problem. However, the three main problems that are associated with experiencing smell distortions are the (usual) unpleasantness of the experience, maladaptive responses to the condition, and the stigma that comes with experiencing things that do not exist.

#### Unpleasantness of distorted smells

Regularly smelling an unpleasant smell for long periods of time can have a severe impact on the affected individual's mood. Their experience is unlike any experience people with an intact sense of smell ever have because they do not adapt to the phantom smells:

It's not like perfume, where you smell it for a while and then it fades. (subject 0383)

The unpleasant distorted smell can also be triggered by all types of stimuli and can thereby complicate activities of daily living like taking public transportation:

… commuting by subway was especially difficult for some reason. (0035)

… This was particularly disturbing when I was riding on public transportation, where I was bombarded with dirty hair smell, underarm and genital body odors, strong colognes and especially bad breath and garlic odors emanating from my fellow passengers. (subject 0423)

Smell distortions can also be triggered by romantic partners, which can result in relationship problems:

My relationship with my wife has degenerated since I cringe when she comes within smelling distance. (subject 0011)

My condition is killing my sex life because when I ask “can you smell that” my partner thinks I am referring to her. (subject 0047)

I have not told my partner because I did not want him to think he smells bad to me and he has never mentioned that I have a different smell either. (subject 0052)

The most common trigger of distorted olfactory perception is food. When food elicits unpleasant olfactory experiences, it is not possible to enjoy food and it can even become difficult to eat (subjects 0043, 0045, 0046, 0284, 0429, and 0483).

A month after the accident I started having horrible phantom smells. They were so awful that I couldn't eat and was feeling horribly nauseous most of the day […]. In a few months I had lost 30 lbs. because I wasn't eating. (subject 0035)

For the last six months I am smelling only a smell like burning vegetable oil. The smell is so strong that I use a mask, but it is of no help. […] I am so hungry because I can't eat any food, even without oil. Everything smells the same. My life is hell. (subject 0096)

#### Maladaptive responses to experienced odors

Another big problem of distorted olfactory experiences is that it is an often difficult and long process for the affected individuals to determine that their perception is non-veridical. During this process the affected individuals behave as if there is a source of the unpleasant odor and these odor management behaviors can create practical and social problems:

About three years ago I started smelling diesel fumes which nobody else around me seemed to notice. This went on for about two years and I started to think that I was just oversensitive to fumes so I tried to live with it. Then, three months ago, I stayed for a few weeks in a home with a fryer that was giving off an awful chip fat smell (at least that was how I perceived it) and since then I have this smell with me everywhere I go. Until a few days ago I thought I was smelling of chip fat. I thought it was in my hair and on my clothes. I kept putting my clothes in the wash even before I wore them, kept washing my hair, really scrubbing it, and when I still smelled it, I sprayed my hair with deodorizing body spray. Whenever anyone came close to me I would pull away, conscious that they may be smelling “chip fat” on me. I then realized that there are times when I didn't notice the smell so I asked a family member to smell my hair. She said all she could smell was shampoo, which surprised me. I described what was happening to me and she assured me that there wasn't even the slightest whiff of chip fat. (subject 0116)

About a year ago I started smelling a strong unusual smell that I had never smelled before. At first I thought it was my urine. Then I thought it was my cat that had an abscess. I started smelling it more and more. I thought it was from the cat sleeping on the couch or my bed. I bought a rug shampooer and shampooed my carpets, upholstery, and washed all the linen in the house, but the smell continued. Then I started smelling it at work and on the bus; so I figured it was me. Recently, I had the nerve to start asking people if they could smell it on me. No one could. I started researching olfactory hallucinations. (subject 0070)

These two examples illustrate common phases through which people with distorted olfactory perception go as they come to realize that their perception is not caused by odor molecules. Often, they first suspect that there is an odor source in their environment. When they notice the odor in many different environments, they suspect that their body or their hair or their clothes are the source of the odor. When others assure them that they do not smell, they sometimes conclude that the source of the odor is in their nose or they start to suspect that their experience is not coinciding with reality.

If affected individuals suspect the odor source in their environment they often engage in excessive odor management efforts. Subjects in this study with distorted smell perception report cleaning their home thoroughly and repeatedly in an attempt to remove the odor source (subjects 0039, 0070, and 0278). These attempts can cause problems with those they live with (subject 0511). One subject reports that she is “gagging my family with air deodorizers” (subject 0216). Others have people come to check out their oven (subject 0392) or the drains (subject 0084). However, sometimes more drastic steps like giving away clothes and furniture (subject 0042) or changing windows to insulate them against smells entering from the outside (subject 0047) are taken. One subject started wearing a mask (subject 0096).

From the experiences reported by the subjects of this study, it seems as if subjects are more likely to have distorted olfactory experiences when resting. Consequently, distorted odor experiences are more frequent at night (subjects 0047, 0068, 0094, 0105, 0106, 0169, 0223, 0337, and 0575), and during passive activities like watching TV (subjects 0050, 0060, 0083, and 0118) and using a computer (subjects 0050, 0118, 0422, 0654, and 0887). Often the affected individuals notice these patterns and, for a period of time, believe the smells to be associated with their TV or computer.

If affected individuals suspect that they themselves are the source of the odor, they focus on their personal hygiene (subjects 0033, 0089, and 0116). More detrimentally, they often become socially reclusive out of fear of embarrassing themselves with their unpleasant body odor (subjects 0033, 0089, 0116, and 0341):

My sense of smell within a few months' time turned to always smelling a bad distasteful smell. I first noticed it in my bedroom and then began to associate the smell with my partner first and then with myself. I have washed everything and cleaned, changed soaps, done everything I can think of but the smell seems to now follow me around and I hate it! Even when I use perfume I can still smell it. My partner and I have a good relationship. I have this nagging feeling that I am sick and that is the reason I have this bad smell around me, but I have no basis for it. (subject 0052)

Some individuals with distorted olfactory perception also suspect the source of the odor they experience in their nasal cavity, which would explain why nobody else can smell it:

The problem is that there seems to be a smell from my nose or mouth. The ear nose throat doctor says it's phantosmia, but can you have that if there is a smell? (subject 0827)

These individuals often try do manage the odor with sinus rinses and similar procedures (subjects 0150 and 0777).

Most of the ways in which affected individuals respond to odor distortion as if it were a real odor are harmless. They do however contribute to the patient's frustration because they do not result in a change in the symptoms and contribute to the feeling of not being in control. Some responses can also lead to conflict with others who have a veridical perception of the olfactory environment. One reason why affected individuals are willing to accept implausible explanations for their odor experiences, like that the smell of a sandwich got stuck in their nose for several months, is that there is a widespread unjustified stigma associated with having non-veridical sensory experiences [140].

#### Stigma

Phantosmia is the olfactory equivalent of phantom pain in pain perception [141,142] and tinnitus in auditory perception [134,135]. There are striking parallels between these conditions. In each case the phantom perception usually occurs with the loss of veridical perception. Phantom pain, which refers to pain in a body part that has been amputated or deafferented, is believed to be a consequence of plastic changes in the brain due to the changed sensory input [141]. Maladaptive plasticity has also been suggested to be the cause of tinnitus [135] and it has been speculated to play a role in distorted olfactory perception [4]. Phantom pain and tinnitus are both associated with a stigma and phantom pain has often been viewed as a type of mental disorder before the underlying neurological mechanisms were understood [141].

Patients with phantosmia seem to face a greater stigma than those suffering from phantom pains and tinnitus. That phantosmia is likely to be stigmatized is also clear to those that suffer from the problem and many therefore chose to not tell others (subjects 0052 and 0470). Others are worried that their phantosmia is not merely the consequence of neuronal plasticity after smell loss but a sign of a serious psychiatric problem (subject 0745). Others told their family or coworkers about their condition and have been labeled as “crazy” (subjects 0359 and 0729).

## General discussion

The patient reports evaluated here show that olfactory dysfunction has a severe impact on affected individuals. This is in contrast to the low importance usually assigned to olfaction. In a survey amongst Canadian college students, for example, smell was ranked as the least important sense [143] (page 106). In the “Guides to the Evaluation of Permanent Impairment”, published by the American Medical Association [144] a person's impairment due to several conditions is quantified. A complete loss of the sense of smell is suggested to be a 1% to 5% impairment. Deafness is a 35% impairment and blindness an 85% impairment. Smell loss is considered much less severe than the loss of the other modalities because: “Only rarely does complete loss of the closely related senses of olfaction and taste seriously affect an individual's performance of the usual ADLs [activities of daily living]. For this reason, a value of 1% to 5% impairment of the whole person is suggested…” (page 270). “Activities of daily living” include bathing, feeding, eating, personal hygiene, and sexual activity, activities that are shown here to be seriously affected by olfactory loss. Distorted olfactory perception is not discussed at all in the “Guides to the Evaluation of Permanent Impairment”, although tinnitus, the perception of sound in the absence of an external stimulus, is considered to be a 5% impairment because it interferes with “sleep, reading (and other tasks requiring concentration), enjoyment of quiet recreation, and emotional well-being” (page 248). The reports in this paper show that in some cases a constant unpleasant smell also interferes with these activities.

It has to be noted that collecting patient reports online has two major limitations. First, it is likely that the reports collected here are not representative. The more severe the consequences of the olfactory dysfunction are experienced, the more likely is the affected individual to participate in a study about the condition. Because the subjects of this study are self-selecting and not necessarily representative, it is not possible to derive statements about the percentage of the total population of patients with olfactory dysfunction who experience any of the discussed consequences. This, however, is also true for studies of the consequences of olfactory dysfunction that enroll those patients that seek medical help for their condition (for example [27,108-110,115,123]). In fact, the barrier for participation in an online study like the one presented here is presumably lower than the barrier for participation in studies at smell clinics. Many affected individuals will be motivated enough by their condition to fill out an online survey, but not motivated enough to schedule an appointment and visit a smell clinic during office hours. An additional advantage of an online study is that there are fewer geographical and economic barriers to participation than to a study based at a smell clinic.

The major disadvantage of an online study compared to a study at a smell clinic is that the patient reports in an online study cannot be verified. This is the second major limitation of this study. Self-report of olfactory function in healthy individuals is notoriously unreliable [145,146] and although individuals with functional anosmia are generally aware of their impairment [147], the actual extend of olfactory loss in the subjects of this study is unclear. Similarly, the accuracy of all other information provided by the subjects anonymously cannot be independently verified. Confabulations by the subjects are also possible in studies in smell clinics, but olfactory function can be assessed objectively in these studies and the accuracy of basic facts about the subject such as age and gender are also more reliable. Furthermore, the higher entry barrier of smell clinic-based studies results presumably in more reliable data. It is easier to imagine that somebody mischievously submits wrong information to an online survey than that somebody takes a day off and drives to a smell clinic to lie about their condition there. Regardless of these limitations, the current study illustrates vividly the diversity and severity of the consequences of olfactory dysfunction.

## Conclusion

Olfactory dysfunction, although it is often ignored or trivialized, can have severe consequences for those affected by it. While the practical problems of olfactory dysfunction are dwarfed by those of visual impairments, smell loss-induced social isolation and smell loss-induced motivational anhedonia have outsized detrimental effects on the quality of life of these patients.

Better educating the patients, the public, and medical professionals about disorders of olfaction would improve the quality of life for those affected by reducing the practical and social problems they often face. However, a comprehensive solution can only be provided by research into an effective treatment.

## Competing interests

The authors declare that they have no competing interests.

## Authors’ contributions

AK conceived of the study and collected and edited the patient reports and evaluated the questionnaire. AK and DM together designed the study and wrote the manuscript. All authors read and approved the final manuscript.

## Pre-publication history

The pre-publication history for this paper can be accessed here:

http://www.biomedcentral.com/1472-6815/13/8/prepub

## Supplementary Material

Additional file 1 **1,000 reports about olfactory dysfunction: Edited excerpts from the 1,000 reports about olfactory dysfunction submitted online through our website.** These reports and the questionnaire that was filled out by the same subjects are the basis of this paper. Click here for file

Additional file 2 **Results of the questionnaire: The complete results of the questionnaire are shown.** The 43 questions shown here include the 27 questions shown in Figure 3. Click here for file

Additional file 3 Methods: A more detailed description of the methods. Click here for file
