# Supplementary material for: Hidden consequences of olfactory dysfunction: a patient report series
Source: BMC Ear Nose Throat Disord. 2013 Jul 23;13:8. doi: 10.1186/1472-6815-13-8 (PMC3733708; doi:10.1186/1472-6815-13-8)
Supplement: Additional file 1 — 1,000 reports about olfactory dysfunction: Edited excerpts from the 1,000 reports about olfactory dysfunction submitted online through our website. These reports and the questionnaire that was filled out by the same subjects are the basis of this paper. [file 1472-6815-13-8-S1.docx]

# case 0001

I lost my sense of smell four years ago. The doctors told me that it is natural and that it is not a sickness.

# case 0002

As far back as I can remember I never had a sense of smell. It may have been caused by a head injury that I received as an infant. Up until I was about 8 or 9 I would lie about my disability, since what kid wants to be different? Smelling seemed to me like religion, you just had to have enough faith to make it true. I have never had any tests or received any treatment. I am still a decent smell liar. "Darling, what is that perfume you are wearing? It smells intoxicating!" I read an article recently about somebody with no sense of smell who could not taste the difference between mint, peanut butter, or cherry ice cream. I can easily taste the differences between these foods, but I would have no sensation if each were placed in front of my face. I have always assumed that my taste buds became more acute to make up for my faulty sniffer. So obviously texture also plays a major role for me. I have never been able to receive any sensation from smelling things. I do notice gasoline if it were nearby. The vapors feel differently when I breathe.

# case 0003

The following facts should be of great interest to you as I have discovered ways of regaining a lost sense of smell and taste. I lost my sense of smell 25 years ago. A few years later I was diagnosed with nasal polyps. The polyps were surgically removed, however, the sense of smell did not return. Ten years ago I had a second surgery for polyps and this time my sense of smell returned within hours of the surgery. Since then my sense of smell came and went several times. During a time when my senses had disappeared I developed polymyalgia rheumatica and was treated with Prednisolone. The strange thing was that my senses re-appeared and have been fairly reliable since. Earlier this year they disappeared again, but immediately came back again following a game of tennis — I have been convinced that lack of circulation could be part of the problem. Just over a week ago my senses disappeared again and coincidentally my polymyalgia rheumatica flared up — I took 15 mg of prednisolone at 9.30 am and my senses returned at 4.00 pm the same day. I hope that this information will help you to shed some light on this dreadful infliction.

# case 0004

At one point my asthma was particularly troubling me, so my allergist put me on a course of systemic steroids for about a week. On the second or third day as I picked up my baby son, I realized that I could smell him and this in turn made me realized that this was the first time I ever smelled him; I must have had no sense of smell for at least several months and the steroid brought it back. After the systemic steroids were discontinued, I lost my sense of smell again. The ear nose throat doctor found conspicuous polyps in my nose. I spent ridiculous amounts of time every day with my nose to my son's little head, just inhaling his smell. I don't know if anyone can comprehend what it's like missing that primal connection to your child. There is something profound and powerful about a mother smelling her baby that I cannot explain, but it is viscerally important. So I don't know when I ceased to smell, but it was gradual enough that I didn't notice. That said, the absence of smell is unspeakably painful. I am not a tearful person, but thinking about how I was unable to smell my second and third children I feel something I can only describe as mourning. It is equivalent to missing out on important stages of their development, like first steps. I have always loved to cook, but since I cannot taste food... can you even comprehend what this does to a cook? One of the things that gave me greatest joy in life was cooking for my extended family, and now it's nearly impossible to do so. Perhaps this sounds strange, but I ended up gaining almost twenty pounds before realizing I was consuming more of every food in an effort to taste it. I now gravitate toward salty or sweet foods. A salad is no longer appetizing because the subtle flavor of the greens is gone, as is the complexity of any dressing, so it is like eating paper with glue. Creamy textures are more satisfying somehow. I treat the rare occasions on which I have to take systemic steroids to control my asthma as holidays during which I try to schedule nothing else but interesting meals and time to smell my children. The other thing I've noticed during my anosmia vacations is just how rich the world becomes when you can smell. The rich smell of the earth after a rain, the offensive smells of cow manure or diapers, the smell of exhaust from other cars. Another embarrassing thing I've run into was when my last two children were babies — I wouldn't notice that they had a dirty diaper, of course. I had to set an alarm to check my third child's diaper every 30 minutes because I never knew when she was going to be dirty, and she was very susceptible to rashes. I remember two different mothers who treated me with great disgust, as if I didn't care about my child or hygiene, almost as if I were abusive. I had another horrible experience when I apparently left a load of laundry in the washing machine for too long. The head of the day camp where I was sending my daughter took me aside on the third day of the week and asked why I was sending my daughter to camp in clothing that smelled of mildew, and if there was some problem at home, etc. It was absolutely mortifying. My daughter was too young to say anything about it, so there I was. It's a source of enormous anxiety for me. People simply don't believe you when you say you have no sense of smell! Speaking of hygiene, I also cannot tell when I smell and need to take precautions to make sure I'm not offending others. I've realized that it's much easier for me to forget to put on deodorant now, and also to brush my teeth. I know this is difficult to believe for others, but I don't think people realize how closely tied hygiene and smell really are in terms of actual self-care. I don't care as much ultimately about what the public awareness of this condition is; I'm resigned to anosmia being a joke for those who don't have it. I do wish that doctors took it more seriously. I have talked to too many doctors who did not believe that I cannot smell. Some want to blame me, saying I'm simply not taking my nasal steroid properly or often enough. (I have gotten quite religious about it, but it works only intermittently and never to the extent of the systemic prednisone.) One especially ignorant fellow just didn't believe that I'm unable to smell anything at all and treated me as if I were some hysterical female, telling me it was entirely psychosomatic. This needs to change, and this is why I've just spent the last 45 minutes pouring all this out for you.

# case 0005

For at least 20 years, I have had seasonal allergies. When affected by the allergies, I often suffered from a diminished sense of smell — but only temporarily. The sense would return, much like many people encounter when hit with a common cold. Then about six years ago, I noticed my sense of smell wasn't bouncing back as quickly or as completely when my allergies subsided. At first, the difference was barely perceptible. Eventually, the bounce back never really happened. My sense of smell just kind of faded away. Other than very minor hints of smell/taste that on rare occasion occur first thing in the morning, my sense of smell has yet to return after six years. Years ago, I went to an ear nose throat doctor. I then went to a neurologist. He was utterly unprepared to deal with the issue. He started searching the web for information on anosmia while I was in the office. It's a huge loss. I fully understand the risk of depression from this condition. Besides the loss of smell, I've suffered a complete loss of flavor-tasting ability. That is an immense loss as well. Even more so is the loss of memories that smell used to so vividly unlock. I so miss the fragrance of a pine forest to take me back to my childhood camping in the mountains. I want to smell the turkey cooking on Thanksgiving. I want to smell the chocolate when I walk into a candy store! It's a weird affliction. People don't really get it. They think it's not as big a deal as it is. After all, they figure anosmics aren't disabled. We don't need seeing-eye dogs or sign language to interact with our environment. And they are right -- partly. We can function without drawing attention to our plight. We can do virtually everything we could before we lost our sense of smell, except enjoy the immensely important aspects of human life that most people take for granted.

# case 0006

About ten years ago I started to strongly react to different smells. I particularly react to chemical odors, but also to spearmint. One odor, I don't know what is, I noticed in church so I had to sit at the back to make a quick getaway if needed. I smelled the same smell in a public toilet, so I am guessing it's a cleaning agent. I can go for weeks without encountering the odor and then it hits me again. I usually sleep with the fan blowing on my face and Vicks plugged up my nose. I haven't told my doctor as I have always thought I was alone in this.

# case 0007

For about a year I get these clouds of smell (as I call them). No one else smells them, but they are very real to me. They are very different and then at times all the same. At times it is like the room is filled with cigarette smoke so full it burns the back of my throat. No one in our house smokes and no one else smells it. Other odor variations are cherry pipe tobacco, the smell of brick being cut with a brick saw, jasmine, a smell so sweet it makes me sick. All body washes and shampoos make me sick; they smell like poop. I know that sounds crazy. Some food, Asian food especially, just reeks like an outhouse.

# case 0008

Nine years ago I was in a serious car accident. In the hospital, I realized food tasted different, but I thought that it was just because it was hospital food. When I got out of the hospital, it took maybe a week or two for me to realize that I couldn't smell anything. All I could taste was salt and sugar. Sometimes I thought I could smell lemons. A CAT scan showed that I had sheered the olfactory nerves completely, and that I would never smell again. It took a long time to stop feeling like a freak. But now, almost 10 years later, people still don't understand what it's like for me. I'm constantly asked to smell things, by people who know I can't smell, but forget. Some people don't believe me and try to force me to smell things. I have a two year old daughter and I've never been able to smell her. I miss the smell of pickles, early September mornings, the ocean, gasoline, matches and garlic; I could go on and on. I would do anything to stop having to fear smoke or fire or spoiled milk, to feel like a normal person again. There isn't anything I wouldn't do.

# case 0009

At an early age I realized I couldn't smell. My parents were skeptical about it when I reported it. They didn't follow up. I pretended to be able to smell, rather than tell people. It was like a point of shame, oddly. I used to pretend I could smell, mimicking disgust when bad smells were detected by my friends. I have come to rely on my vision, and taste to detect spoiled food. I have been fooled, and gotten sick a few times, however. I have managed to compensate for the lack of a working nose. I had an undetected kitchen stove fire and gas leak. I detect ammonia, and very strong cleaners, but mainly from the effect of the vapors which invade my mouth, rather than my nose. I visited an ear nose throat doctor who examined me a few years back, but nothing came of it. A co-worker went to an ear nose throat doctor recently, and endured a surgical procedure which restored her sense of smell.

# case 0010

I do not have any idea when I actually lost my ability to smell but first noticed it when I was about 20. I can't actually remember smelling any odor, so onset is hard to pinpoint. It has definitely affected my taste. All food is bland (unless it's salty, bitter, sweet, or sour). It is hard to describe the effect on my eating. My enthusiasm for food has almost vanished with the exception of my unhealthy love of very salty food. Conversations about food are weird and I often pretend to taste things. My wife is aware of my disability but she can't quite understand it. My sleep is impaired sometimes because I wake up fearing a house fire and can sometimes convince myself that I can see smoke. I often need to get up and check the house for fires or gas leaks. I cannot eat leftovers unless my wife smells them first and when I cook meals I am usually guessing with many of the ingredients and sometimes overdo things. I have not found anyone else who has this problem and have not been able to talk about it with others. It's good to write this at least. Thanks for the opportunity.

# case 0011

About 6 years ago I noticed that many smells were becoming more intense. Unfortunately this only occurred with unpleasant smells. Nearly all of my wife's beauty products became nearly unbearable and my wife's breath took on a deeply sour note (that only I can smell.) Many foods are now too intense to be around. I have become depressed since I can rarely escape bad smells in many places and have researched methods to desensitize my olfaction. I have gone so far as to attempt to self-induce anosmia. My relationship with my wife has degenerated since I cringe when she comes within smelling distance. My wife is not the only source of these unpleasant smells (I even went to therapy about it, in case it was hypochondria).

# case 0012

I lost all sense of smell about four years ago. No head trauma, no illness, no allergy to anything or any drug, no specific exposure event that I can recall. I don't know if it was sudden or not. I didn't notice myself, people would tell me they smell things and I just wouldn't. I manage an apartment building and tenants would sometimes come to me saying things like "it smells bad in the laundry room, like a dead animal" but I would smell nothing. I would search the corners and find nothing, and tighten the gas connections, and they would say it was fine now. At some point I realized I could not smell coffee grounds or coffee brewing, nor toast starting to burn when it would set off the smoke detector. After I realized I could not smell I tried everything, newly opened spice jars, pickles, rubbing alcohol, every sort of strong food odor, etc. I can no longer smell soup simmering or hamburgers frying, but I can still taste all I need to. All fruits taste different, as do all cold-cuts. I can taste various condiments and distinguish brands of salami and types of cheese. So lack of smell does not really make me despair about diet. But then, I never was a foodie. Never could get interested in cooking or fussing about savoring foods in restaurants. My doctor did not know such a symptom existed. He was stunned that this could happen and stuck a couple things like coffee under my nose to test me. He didn't even look up my nose with a light, much less a scope. I felt sure he'd never looked up anyone's nose, wouldn't know what to look for. When he found out it had a name, anosmia, he looked up possible causes in their computer and decided to send me to get an MRI and a CT scan. He refused to send me to an ear nose throat specialist because "I cannot send you unless I first diagnose a condition they can treat, like sinusitis". I recently realized there are two things I can smell, but now learned they are probably not being smelled by my main smell organ. Still, it was news. I could smell "locker room mildew" in a pile of rags that sat on the patio for a week. But when I tried to repeat the smell I couldn't. Perhaps I broke up the scent when first disturbing the pile. Then yesterday I smelled a diesel truck that had just come to a stop. But again, when I tried to repeat it the smell was gone.

# case 0013

I had a good sense of smell and had been able to identify many wines by taste/smell. Then I found that my sense of smell was disappearing. During this period I found that when I was exercising — mostly running — I could regain my sense of smell. During a dinner party when a good wine was served I would excuse myself from the room and run up and down the stairs several times. This restored my sense of smell for a period. But eventually I stopped smelling entirely. I visited the doctor to enquire and was referred to an ear nose throat specialist. Two surgeries and several nasal sprays did not help. However, a couple of months later I went scuba diving and after a very slight nose bleed my sense of smell reappeared dramatically and I could smell strongly and it stayed there for almost a month. It was a very enjoyable period and meals were like eating in "technicolor". After returning home I revisited the doctor and told him of my experience. After almost a year I was referred, last summer to an ear nose throat specialist again. The specialist said that he could observe polyps again and prescribed an inhaler but my sense of smell still did not return so I was given a short course of strong steroid tablets and my sense of smell again returned. The specialist said that it was not advisable to continue on such steroids for a prolonged period. Shortly after stopping the steroids the sense of smell again disappeared.

# case 0014

When I was four I fell and had a head injury. This probably caused my anosmia but it wasn't until years later, at thirteen, that my family realized I couldn't smell. I was told I could have a pet cat as long as I took care of it and one of those responsibilities was to change the cat box as soon as I smelled it. Needless to say I was in constant trouble and was thought to be irresponsible and lazy. Eventually I was able to convince others that I just couldn't smell. Whenever I tried to inquire about it doctors would tell me to forget about it since I had learned to live without it all these years. I had health insurance, but they would tell me that I should be glad I couldn't smell most things. Life is not easy without it though. I wear perfume to please the people who buy it for me, but I am never sure how much to use unless I ask someone to "smell me to see if I'm okay". Furthermore, I am constantly worried about offending people around me by body odor, especially people I have just met or am trying to make a favorable impression. If you're blind, people forgive you if you are wearing mismatched socks, but they can't see if you have anosmia and therefore a reason why you may have undetected body odor. I am now retired. I don't socialize too often although I have always had an outgoing personality and love to be with people.

# case 0015

As far as I can remember my sense of smell must have deteriorated so slowly that I did not realize that it had gone until it would return very occasionally, sometimes only for a couple of hours. This was noticeable since I was 50. It often returns with no warning and it is as though someone has turned my nose on with the flick of a switch and it disappears again just as quickly. The strangest thing is that following a long haul flight my sense of smell returns sometimes. After the last three long haul flights my sense of smell returned. The first time it took a week after landing to return. The second time it returned within two hours of landing and the third time it returned within 20 minutes of landing. Each time my sense of smell stayed with me for about 6 weeks and then gradually disappeared again. It does not always work, as I make many intercontinental trips and it doesn't come back on every trip.

# case 0016

Six months after sinus surgery I started to notice periods were my sense of smell and taste was compromised. The periods would last for a few days. My ear nose throat surgeon showed a complete lack of empathy and said this sometimes happened. Over the next couple of years I saw three other ear nose throat specialists but no one could help. Two years ago my loss of taste and smell was almost permanent, I would regain it every now and again but only for hours up to a day or so. I decided to see another ear nose throat specialist. I had another surgery and afterwards I had a slight return of smell and taste but once more this disappeared shortly after. Since then I have seen my ear nose throat specialist on a regular basis. Nothing has really worked but oddly when I have the endoscopic examination, after the anesthetic has worn off I have had recovery of taste and smell. Out of the last five examinations I have regained the senses on four occasions. It comes back suddenly — like someone has flicked on a switch. The last time I had my sense of smell and taste only for about an hour... the previous time it lasted about a month. My ear nose throat doctor cannot explain it and he does not know why it should be happening. I would love to get my sense of smell and taste back but at the moment I have no clear way forward on how to achieve this. I'm sure it is a physical thing, I mean basically shoving a rod up my nose and wiggling it about (the endoscopic procedure!!) seems to have an 80% chance of restoring my sense of smell albeit temporarily! My ear nose throat doctor says my sinuses are completely open and currently I have no infection.

# case 0017

About a month ago I began to sense a constant odor — not sweet, not sharp, not foul. I had never smelled it before. It is dull, most like a medicine smell. It is constantly with me. If I hold my nose I do not smell it. I've thought about seeing an ear nose throat doctor, but in reading up on the net many folks with a constant smell sensation have found a visit to the doctor unhelpful. During the day when I am focused on other matters I am not bothered by it.

# case 0018

I first noticed a distinct unpleasant smell rather like smoke when no one else did and I found the smell of grease very unpleasant as I drove past fast food restaurants with fryers. My sense of smell became less acute from that point on. Now I have largely lost the ability to smell certain things. It's not all bad since I can't smell most unpleasant biological odors — changing the litter box or cleaning up vomit is no problem. But this condition can also be dangerous since I don't smell smoke unless it's quite concentrated. I do smell mints, many herbs, fruits, and a variety of other things, albeit less intensely. It seems to have stabilized. I find it disconcerting that in ordinary medical practice physicians appear to be unconcerned and that routine physical exams of elderly people like me don't even include the question.

# case 0019

I lost my sense of smell after suffering a concussion due to a fall. I didn't realize it was gone until the day after my injury. When I started to become aware of the fact that I could not smell, I went around my home, trying to smell anything — coffee, perfume, lotion, cat litter. I was quite surprised. I did not know it was possible to lose one's sense of smell, and no one in the emergency room had warned me that it was a possibility. The loss was very hard to take. Not only had I completely lost my sense of smell, but I could not distinguish flavors in food. Going from a world of smell to a world without smell overnight is quite devastating. Without any fragrance, my days felt artificial. But I think the most painful part of the loss was the total lack of compassion from others. The doctors that I visited told me coldly that my sense of smell may return or it may not, as if it were a trivial matter. Colleagues and acquaintances joked that I wouldn't have to smell my daughter's dirty diapers anymore. A family member, upon hearing of my loss, asked me to give her all of my bath soaps. It has been about five months since my injury. I've had the phantom smell, sometimes constantly and sometimes intermittently, for over three months (none of the doctors warned me about that, either). I've adapted to the loss; I no longer feel like I'm trapped in an artificial world. I've found foods that I can enjoy, and stopped drinking soda, which now tastes metallic. I have some hope that my sense of smell will return. I have been greatly disappointed with the information provided to me by doctors since my injury. I have gathered most of my knowledge on head injuries and anosmia from books, articles, and others with similar injuries. The medical professionals seem either clueless about this condition or completely unwilling to offer any information or advice.

# case 0020

I lost my sense of smell after injuring my head in an accident when I was 12. I could not smell anything for 11 years, really forgot what "smell" meant not too long after losing my sense of smell. A couple of years ago I started taking medication to treat seizures from the head injury. Since then I sometimes perceive scents. I've "smelled", I think, pizza, coffee, laundry from the dryer, Buffalo sauce, and a cigarette. The smelling sensation is very rare but I haven't had anything like it since pre-head injury. I think I taste quite a bit. I have a hard time naming the flavor of something if the choices are in the same family (i.e. which fruit flavor an unmarked candy is). But I know which ones I like and don't like. I know I like certain brands of coffee and not others. Lack of smell does not decrease my sex drive at all. I worry about smelling genetic differences but that's the only sexual hindrance. I get the feeling that people think I am lying about my smell a lot; especially once they see me enjoy food. That's pretty aggravating. When I was 12 and first told my doctors I couldn't smell anymore they acted like it was impossible. I eventually met a number of other people who lost their smell after a head injury. It didn't make us friends but it helps knowing they're out there. I often feel paranoid about how I smell. Often when people say something smells bad I wonder if they mean me. In fact, my friends tell me I wash clothes too much; that my clothes smell like laundry detergent. I still wash them a lot. I stay away from wearing fragrance. The only things I get really sad that I can't smell are Yankee candles. They have such pretty pictures and colors. I worked at Hallmark at one point and looking at the Yankee candle display could make me cry every now and then. I do get angry that people don't treat anosmia like a real problem. There is very little awareness or acceptance or understanding from many.

# case 0021

When I was twelve I had an accident and my nose was bleeding profusely. From that day onwards I can smell only very few things. Because of this it is difficult for me to live with people around me as they smell and I don't. If people ask me about smells I feel shy.

# case 0022

My son, who is 6 years old, starting asking me this year how to smell. He is a bright child and I couldn't understand how he didn't know. I told him to just sniff in through his nose and smell will happen. Well, this was the beginning of many "smell tests" that I created. He apparently has no sense of smell at all. Interestingly, the biggest (and only) issue I've had with him is his eating. He is an extremely picky eater. I thought he might have sensory issues because he focused so much on what foods look like. However, he loved all sorts of junk food despite texture. It turns out that he loves sweet and salty tastes. I even remember noticing he never commented on smell. Unfortunately, I have no idea if/what to do at this point.

# case 0023

I had a head injury and sheared my olfactory nerve three years ago. There is no chance of my sense of smell or taste returning and I am very depressed. I saw a psychologist but with no experience in this condition she wasn't much help. No attitude advice — just medication. I cannot seem to accept my fate. Not a terrible cross to bear but cooking was my hobby. I'm beginning to just eat the cheapest most nutritious foods but my husband is worried that I am purposefully denying myself pleasure. He, like most people, cannot understand what isolation this condition causes.

# case 0024

I had a tumor that was wrapped around my "smeller" removed and as a consequence of the surgery I lost my sense of smell. I feel fortunate to have had this sense for 33 years but am unsure how best to establish a daily routine without it. I can only taste sweet, sour, salty, and spicy. Food in general is completely unappetizing and within six months after surgery I lost almost 20 lbs. (I've always been tall and skinny anyway) and my hair began to fall out, I assume because of the drastic weight loss. I'm currently coping by relying heavily on my family, to "be my nose", and I put a more than average amount of hot sauce and/or salt on just about everything I eat. I feel stressed about not knowing... not knowing if there's a garbage can in the house that needs attention, or if the cookies in the oven are burning, or if the house smells like "dog", or if that yogurt or sour cream is still good. However, getting satisfaction from food and keeping weight on seems to be the most challenging task for me.

# case 0025

I had an extremely well developed sense of smell all my life but lost it suddenly after contracting a flu-like upper respiratory tract infection six years ago. I was diagnosed as anosmic by an ear nose throat consultant after brain scans and taking a smell test about six weeks later and told that I was unlikely ever to recover my sense of smell. My sense of taste in its strictest definition i.e. sweet, sour, salt, bitter, and umami was unaffected but I was unable to identify anything I was eating other than by texture (all cheese whether cheddar or Roquefort just tasted "salty"; all cakes just tasted "sweet"; eggs, cream, and milk had no flavor at all). I could not smell anything. Flowers, perfume, coffee, petrol, garlic, rotten fish, smoke, human or animal odors, nothing had a smell for me. Curiously, although I couldn't identify any "complete" smells, after a few weeks I began to detect a very few single odor "elements" - which I had never smelled in isolation before. The first one I noticed was something present in the peel of citrus fruits (which I normally love) which made me feel sick- I was so sensitive to this that on one occasion I touched an orange at about 9 pm, washed my hands, washed them again before filling a glass of water to put by my bedside, woke at about 2 am and took a sip of water and could still smell the "orange" element on the surface of the glass — 5 hours after touching the fruit. The second element I noticed was something which must be present in the base of a number of household cleaning products and toiletries — again a chemical smell which I had never smelled in isolation and found hard to describe but which was very unpleasant to me and seemed to be everywhere. The third smell was something in the skin of cucumber which made me feel nauseous. Although I really disliked all these smells I was somewhat reassured because it suggested that something must be still working in my olfactory system. I was very disturbed and depressed by how much I was affected by my anosmia, I lost my appetite, became very cautious about eating away from home because I knew I couldn't detect meat or fish which was off, was worried that I might have left the gas on or set fire to something without knowing it, felt extremely anxious in company unless I had just washed , cleaned my teeth and was wearing fresh clothes as I could not tell whether I had body odor or not and felt strangely distant from my family because I couldn't "smell" them. After a couple of years I gradually became aware of two more "single" odors which were "aromatic" in nature — something common to cedar and sandalwood (and petrol) and something common to lavender and rosemary but I still couldn't smell any "whole" smells — just these faint strands .However, using this limited smell vocabulary, I learned to identify more and more things. For example although I couldn't smell petrol I learned to detect the aromatic in it. I was so delighted to smell something nice that I sniffed everything eagerly for even a hint of a smell — pencil sharpenings, leaves, tomato stalks, old books. At first the smells would fade after a few seconds but gradually persisted for a few minutes. I also began to be aware that there were smells around that I couldn't actually perceive but which seemed to be pressing on an olfactory threshold. I could "feel" they were present (just outside the "smell" door) but had no idea what they were ,for example someone might walk past me and I could tell they smelled of something but couldn't tell if it was perfume or body odor, or I might be in a particular aisle in the supermarket and be aware that it had an odor but without looking not know if it was pet food, vegetables, or toiletries. When I asked other people who were with me about it they would confirm there was a smell but often that it was very faint. It was as though, in my smell deprived state, I was becoming hyper-sensitive to the very existence of odors even when they were not recognizable. Two years ago I experienced, for the first time since I lost my sense of smell, a recognizable odor. Since then I can detect more and more recognizable odors (lily of the valley, hyacinth, chrysanthemums, pine sap, cigars, onions, thyme, etc.). This has made a huge difference to me — I have regained much of my appetite for food, I drink wine again (though my appreciation of the subtle differences has not returned), I enjoy gardening more, I rejoice whenever I detect some new odor and, in general, feel much more positive about life. Unexpectedly, much of this recent improvement has taken place since I started smoking again. I have written at some length here because there seems to be a total lack of interest in the very distressing condition of anosmia — most people dismiss it as a joke: "aren't you lucky you can change babies' nappies without noticing", whereas if I had gone blind or become deaf everyone would be sympathetic. I have learned not to mention it anymore and work my way around it without letting anyone know. There also seems to be very little helpful research available so I am very grateful for the opportunity to describe my personal experience to people who may be interested.

# case 0026

Years ago I started to smell smells that I thought were real. However, others could not smell those smells. As this problem began to occur more frequently, I began to wonder why only I can smell it. I investigated it on the internet and found out my brain is making a phantom smell, it's not in the environment and that is why no one else could smell it. I haven't approached a doctor about it yet, out of fear, because I don't want a label. Now the smell comes at least monthly and it can linger for a day or up to a week. I cannot identify the smell; I try to associate it with something but I come up empty.

# case 0027

I realized that my sense of smell had changed when I could not tolerate certain smells that are usually pleasant, for example coffee, cut grass, celery (absolutely the worst), butter (especially buttered popcorn), apples, peaches, cucumbers, melons, perfumes, shampoos, soap, grilled meats, and poultry. I also discovered that I could no longer stomach certain related tastes, such as vegetable juice, carbonated beverages — especially colas, orange juice, red wines, anything melony, pretty much anything that smells bad to me I cannot stand to taste. I can drink coffee as long as I don't have to smell it brewing. This happened to me over the past eight months or so. My quality of life is suffering badly. No one can understand how badly this weirdness affects me and how miserable I feel.

# case 0028

I fell and my head hit the ground. I was unconscious for around 20 minutes. About two weeks later, I started to notice a weird smell around my house, like nothing I had ever smelled before; slightly aromatic and pungent. A couple of weeks later I visited somebody and they apologized for the strong curry smell in their house as I walked in — I could smell nothing!! For some weeks, all I could smell was the weird pungent smell - it drove me nuts. I would wake up with it and go to sleep with it but, at this stage, I still didn't realize I was also losing my sense of taste. An ear nose throat doctor explained what had happened inside my skull when I hit the ground. He also told me the strange smell usually kicks in almost straight away and forewarned me that it was unlikely I would ever regain my senses. In the ensuing months I came to realize how serious this condition was. On one occasion I forgot that I had a pan on the simmer until I saw smoke. On another occasion, I was told that there was a very strong smell of gas in the neighborhood which everyone was worried about; again, I could smell nothing. The only enjoyment I get from food is if it is highly spiced, i.e. with chili, horseradish, tabasco, etc. I am unable to differentiate between types of meat so have to rely on texture, if possible. I get some tang from strong, blue cheese. The sad thing, I find, is not being able to appreciate the everyday smells which we take for granted: perfume, freshly mown grass, freshly baked bread, scent of bluebells/roses/flowers in general. Living by the sea, I used to love the smell of the seaweed around the tide pools. The list is endless.

# case 0029

I lost my sense of smell during a cold ten years ago. Life can be hell sometimes but no one seems to take it seriously. It is a disablement that is invisible. People are always saying "smell that", "taste this". It is very annoying; you wouldn't tell a blind man to look at the lovely scenery.

# case 0030

I developed anosmia after suffering from severe nasal congestion about six months ago. The medical profession does not seem to think that this is a significant problem and offers no help or suggestions as to how or indeed if it can be treated. As a keen amateur cook, I can no longer tell how food tastes or smells and need to rely increasingly on my partner to help me. The loss of two of the five major senses is a strange phenomenon and puts you apart from others. Taste and smell seems to be replaced by texture in finding foods that satisfy - this may be a great thing as I no longer have sweet cravings and would now prefer food such as celery, toast and crunchy nuts than sweeter foods. So perhaps this is a blessing in disguise. Roll on a dropped dress size!!

# case 0031

As far as I can remember, I first noticed that I am not able to smell at the age of six. I would often react the same as friends and family to bad smells e.g. flatulence, but I could never actually detect the smell. Later in life I discovered that head trauma could often cause the loss of smell and was told that before the age of five I was almost knocked unconscious by a blow to the head. Throughout my teenage years the inability to smell would often cause angst with me as I was unaware of my body odor as a result of exercise or perspiring. Equally, I can also recall a number of occasions throughout my childhood at which I made myself physically ill from overexposure to chemicals, glues and cleaners. As I've grown older the anosmia has been the subject of conversation a number of times with friends and family and often leads to the subject of taste. As reported in a number of related articles, loss of smell is directly connected to taste. I believe this may be the reason for my fondness for very spicy foods. Should I have the full range of taste, my palate may lead me to dislike a lot of the foods that I currently enjoy. Of particular interest for me was a recent comment I saw online about anosmia causing depression. I have been recently diagnosed with depression and now believe my long standing loss of smell may have been a contributing factor to my illness.

# case 0032

I had the flu. For a couple of months afterwards I didn't notice that I couldn't smell anything. However, eventually I started experiencing the smell of something like onions and garlic, but not as I remembered them, but as intense, horrible smells that I did not want to be anywhere near. After another three months I became aware of the fruity smell of a shampoo that I was using — I named it "chemical fruit", it was harsh but not unpleasant. In the meantime things I had previously enjoyed such as wine and chocolate had become completely unpalatable, vinegar and margarine tastes respectively. I didn't eat chocolate for over three years. About 18 months after the initial loss I was able to smell roses, which was wonderful, I could smell a bar of rose scented soap as if a whole roomful of roses were present instead of a tiny bar in the bathroom. As each smell returns, I am overwhelmed by the intensity of the odor, when fish came back I was unable to eat fish for a couple of years and I still find it a bit too strong. Two and a half years ago, after a lifetime of drinking coffee, I found myself unable to even take a sip of coffee and have been unable to drink it since; all I can smell is the burnt part. I think of myself having a sense of smell a bit like a piano keyboard where some of the notes don't work. If an odor is a tune then I can only hear certain notes of it, so sometimes I have no idea what I am smelling. I still can't smell smoke, gas, burning, manure, and probably lots of other things that I am unaware that I am supposed to be detecting.

# case 0033

Periodically I have what I refer to as a smell memory issue where I have a recurring odor/smell for days, and sometime weeks. Often it is the same smell but mostly not so. I scrub daily and change clothes daily and change soap and shampoos. I have found that I would not want to go out or socialize for fear of others smelling what I smell but have never found friend or relative who admits to smelling the odor that I perceive. I am generally healthy and never smoked.

# case 0034

Within the last two weeks I smell a strong smell that no one else smells. The only way that I can describe it is that it smells like a strong flowery, earthy smell, almost sickeningly sweet. When I am really busy doing something I don't notice the smell but when I am working alone or don't have any other distraction I smell this same smell. It is now giving me a dull headache. I have never experienced this type of thing before and am worried that there is some type of underlying illness.

# case 0035

After a traffic accident I was in the hospital and visitors mentioned something smelling bad but I didn't notice and thought it was because I was in a lot of pain. When I started eating and drinking, it was horrible, not what it was supposed to taste like. I first thought it was because I was on a lot of medications and maybe they were affecting my taste. When I was home a week later someone was cutting onions and I realized that I couldn't smell anything. I only realized this because my eyes were burning although I couldn't smell anything. It all made sense but it is sad that doctors don't think about your sense smell or see if you've lost it while in the hospital. It was only when I was in for a follow-up exam that we confirmed I had indeed lost my sense of smell. A month after the accident I started having horrible phantom smells. They were so awful that I couldn't eat and was feeling horribly nauseous most of the day; commuting by subway was especially difficult for some reason. In a few months I had lost 30 lbs. because I wasn't eating. Since the horrible phantom smells have for the most part passed I regained the weight because I'm eating again but still have the phantom smells; some days they are pleasant and some days awful. You can't move or run away from them; it's a constant and that has been frustrating. From my experience the medical community doesn't take the loss of one's sense of smell seriously. I know I am lucky and am grateful that I am alive but it is distressing to not smell a baby, my favorite perfume, the man in my life, and enjoying eating out at restaurants with friends.

# case 0036

I lost my smell completely during the worst virus of my life. I had nothing for about two weeks except that many things smelled and tasted like ammonia for the first week, then nothing. Gradually I began to get whiffs of certain things. One evening I had cooked salmon which I could not taste and then when I went back into the kitchen later I distinctly smelled salmon. Smell came back very gradually over several months, I think it was still coming back some a few weeks ago and I would say it was 70 - 80% returned. I still occasionally had an ammonia-like smell, especially when I ate certain foods like peanut butter. Also, I could tolerate rancid foods much less. For example, previously I could eat some old potato chips without it bothering me, but since I lost my sense of smell they taste really rotten. Interestingly, noxious smells in some ways became worse, I can't understand that. I could taste Pepsi but not Coke. Eventually I could taste most things, at least for a little while. It was extremely depressing to have no sense of smell at all. You realize that rooms have smells, water has a taste, etc. I am grateful that it has returned as much as it has.

# case 0037

I had a persistent head cold for two weeks and used a nasal wash to irrigate my sinuses. Since then I have not been able to smell anything. I cannot even smell ammonia. I used to have a very good sense of smell and could smell a package of bubble gum being opened in the next room. Now I can't smell an onion under my nose...

# case 0038

I caught a cold which developed into a chest infection. Over six months I was constantly coughing, sneezing and had a runny nose. Over this time I lost my sense of smell (and taste). A couple of years after the onset of the problem I eventually went to a doctor. I was diagnosed with a chest infection, asthma and a nasal infection and given prednisone tablets for the asthma. I noticed that when the asthma was really bad and I had to take the prednisone my sense of smell returned (as long as my nose wasn’t too bad at the time). After a few days from stopping the tablets my sense of smell would gradually fade. Because of the side effects I am reluctant to take them for more than the recommended three days. In fact, during a trip to the US, I went to a doctor to get a refill on my prescription and he was horrified at the dosage I was taking (25 mg over three days). Because of the loss of smell and consequent loss of taste, my diet consists mainly of highly spiced foods – Indian, North African and Thai. Not that that is a problem, I have always liked those cuisines.

# case 0039

I was exposed to chemicals 65 years ago at age 17. Since then the smell of detergent, perfumes, and other chemicals bothers me. In my current home I experience a terrible smell and I cannot seem to figure out where it originates. I have cleaned everything in the house, but the odor did not go away. The odor gives me a headache and I feel dizziness. I went to the emergency room and all they said was to use an antihistamine. The smell is acrid and really gets to me so much that I have pity parties by myself and cry.

# case 0040

I have parosmia. It began about a year and a half ago. The first thing I noticed that didn't smell right was my body. Several days later I noticed that the strawberries we had didn't taste right and I thought there was something wrong with them, but my partner said they tasted normal to him. Within a few days, nearly all cosmetics, bath products, and household cleaners took on the same unpleasant odor - the smell was sort of a mixture of rotten oranges and Lysol. Each day there seemed to be another group of things that didn't smell the way they should. At the end of three months, there were four distinct distortions that encompassed nearly every odor and every flavor. Life became very unpleasant. I couldn't distinguish newly mown grass from gasoline or dish detergent. Because the perception of flavor is so heavily dependent on our sense of smell, parosmia has raised havoc with my diet. Nearly all food smells and tastes unpleasant. I eat no meat, no seafood, no poultry, no fruits (except lemons and some red grapes) and no vegetables except lettuce and mushrooms. I drink no soda, alcoholic beverages, or fruit juices. Not being able to enjoy foods is the most difficult aspect of this disorder for me because eating is one of the greatest pleasures we have in life. I've been to a specialized smell clinic and have been thoroughly tested, but there really hasn't been anything anyone can do for me. I have been lucky enough to find the Yahoo parosmia group, where I've found tremendous support. Parosmia has changed who I am. There is a level of despair that never leaves me. In the last few weeks, however, I've begun to see some glimmers of improvement, so I'm hopeful that I may not be like this forever.

# case 0041

Sometime after using a nasal spray I realized I could no longer smell anything. I tried vinegar, ammonia, garlic, even dog feces: nothing! Over the next few months I would once in a while "smell" a very unpleasant chemical smell much like insecticide, and several times something similar to hair burning. After visiting an ear nose throat doctor I had to accept the fact that my condition was probably permanent. I am a professional chef and this was devastating. I went into a profound depression and even attempted suicide. That was 6 years ago. I have learned to live with my condition and made allowances. There are smoke detectors in nearly every room in my house. My personal hygiene tends to border on the obsessive, and my poor dog runs at the sound of water. I think the most difficult part about my condition (apart from no longer being able to enjoy the foods I love) is that very few people understand the depth of my loss. They make jokes about how lucky I am that I can't smell some things. It doesn't occur to them that the loss of one sense is bad enough, but I have actually lost two senses as I can only taste a few flavors such as salt, sugar and vinegar. Anything else is blah to me. As a chef, to not be able to taste garlic or herbs breaks my heart. People think: "Wow, big deal, you can still see, communicate". They don't understand that cooking was one of my ways of communicating. It was everything to me. I eventually ended up divorced... The odd part is I still can smell in my dreams, and when I wake up and realize it was just a dream I grieve all over again. I am in a new relationship now and I have no idea what my lover smells like. I had not realized it but even lovemaking is affected by my lack of smell and taste. My grandchildren are a joy to me, but I cannot hold them and smell their sweet smell the way I once could. Losing you sense of smell is no less devastating than losing any other of your senses. You go through all the same emotions: anger, denial, resentment, depression and eventually acceptance. I wish people could understand that and be more sympathetic. I hope your study will make people more aware. Especially doctors, who I have found are the worst ones to say "It could be a lot worse". Thanks! That makes me feel sooooo much better.

# case 0042

Wherever I go I am overwhelmed by very strong odors. They come from my clothes, furniture, and my new apartment. I have given most of my clothes away and have given my furniture away. It seemed to get better, but now it has come back. I cannot sleep most of the time. Wherever I go, I smell the strong odors from homes, furniture, and even my friends' or children's homes.

# case 0043

I was suffering from fever for ten days and now I'm getting a rotten smell when I breathe. In curries, in perfumes, in malls, etc... wherever I go I get a rotten smell. Therefore, I'm not able to eat any kind of food. When I consulted my doctor, he told me that it is just a psychological feeling.

# case 0044

I started losing my sense of smell three months ago. I noticed I did not smell things others did smell. I would have to really breath in deeply through my nose and maybe catch a hint of what they smelled. I went to several doctors but no underlying cause was found and none of the suggested treatments helped. I was very sad and felt like I was "missing out" on so much. Tasting food, smelling good smells as well as dangerous ones. I would give anything to smell again. I have lost one of my five senses! I get from others at least you are not blind or deaf. Well, yeah, I guess so, but I would still like to have my smell back!

# case 0045

I had a head injury. Several weeks afterwards, I noticed that I had lost my sense of smell and taste. However, I can tell whether something is sweet or sour, and feel the heat from chili. Several months after the accident I developed an aversion to certain foods, as they smelled and tasted of something I had never smelled/tasted before. It was the same smell and taste for all the foods and it is not something that I can identify. This has now lasted for 10 months.

# case 0046

I had bone surgery on my left femur three weeks ago. Soon afterwards while still in rehab I noticed a loss of appetite, mild nausea, and loss of enjoyment eating foods I used to like. I soon realized I had a diminished sense of smell and taste. I used to have an excellent appetite and would usually clean my plate at meals. Now I frequently experience revulsion after only a few bites of what I used to consider a tasty meal. This has had a profound effect on my life causing depression and general disinterest in many things that used to be rewarding. I never realized how important the enjoyment of food was to my psychological wellbeing. I have lost about 10 pounds since surgery and I expect to continue losing weight since I do not eat much. Fortunately I am still about 15 pounds above the ideal weight for my height. I continue to try to find nutritious foods that I can tolerate. I am surprised that my loss of smell and taste could cause me to feel nauseous at the thought of eating something I would otherwise be hungry for if I could smell it. One more thing: during the last few days of my two weeks in the hospital, I experienced extreme revulsion at the smell of a cleaning agent that was used heavily throughout the hospital. The continual smell of it night and day began to make me feel so nauseous; I did not want to eat any more meals. I can only hope that my olfactory function will gradually return but from other accounts I have been reading about post-surgical loss of smell/taste/appetite, I am worried it will never return. If that happens, I hope I can overcome this depression and live a normal life again.

# case 0047

About three months ago I started smelling exhaust fumes and I thought the window in my bedroom wasn't sealed so I had it changed. The smell didn't subside so I went to the doctor who referred me to an ear nose throat doctor. I have a perforated septum and have had two operations, one being a silicone button the other a skin graft with skin being taken from my buttocks. The skin graft lasted a month and the hole was back. The ear nose throat doctor treated me with antibiotic but the smell remained and I have resorted to eliminating smells one by one. My coworkers stopped wearing perfume, but that hasn't helped. It seems worse when I lay down at night. My eyes sting, my throat stings and all I can smell is exhaust fumes. This has to be the most frustrating illness I have ever had and the doctor has told me there is nothing more he can do. My condition is killing my sex life because when I ask "can you smell that" my partner thinks I am referring to her.

# case 0048

I didn't notice any particular dulling of my sense of smell until my later teen years. I remember when my grandmother was making pizza and complaining about the intense stench of parmesan cheese, which I couldn't detect at all. Shortly after starting university, I found that my sense of smell was fading very quickly. It caused me to lose weight my first year, because I lost so much interest in eating food — I tended to fixate on textures. A lot of the flavors disappeared and I ate very few things as a result, sticking mostly to foods I could "remember" the proper taste of. I was living in a dorm, and none of the other students thought this was normal, so I didn't seek out medical consultation. I also wasn't completely surprised, as I have a great-uncle who lost his sense of smell before he reached adulthood, and a cousin on that side of the family who was much the same, so no one in my family really remarked on it being too odd. The last thing I remember smelling was when I was 19 years of age. I was driving with a somebody past an open sewer. While they fought not to vomit, I was sure I could smell pea soup. I remember that moment clearly; though I didn't know at that time it would be the last thing I ever smelled. A lot of people think that this is a blessing, I can't smell cat litter or baby diapers or skunks, I'm oblivious to people with bad breath or body odor. But I also miss out on the smells of food and cooking, I miss out on the smells of nature, and of course there's the fear factor. I spent years being quite paranoid about fires and smoke dangers. I also almost made myself very ill at work once when a sewage leak happened in our building. I stayed at work while other people were fleeing with extreme nausea. Plus, there's the ostracizing that can happen. No one really knows about anosmia, I've had people react oddly to it (though most adults take it in stride). It's not an obvious disability like being blind or deaf, there are days when I feel like I'm normal, and days when I wish people could understand. It's a regular occurrence for even long-term friends and family to ask about how something smells, or to check on something by smell. Oftentimes I just go "oh, that's great" rather than make waves, or have to launch into an explanation, or remind someone. It doesn't make for great dinner conversation to explain that I don't get the same flavor sensations from food that they do. I've often wondered if this is genetic, which is sort of what my family had assumed all those years ago, or if it was somehow due to my grandfather's heavy smoking. I also wondered if it was some sort of brain trauma, I'd been smacked in the back of the head with a brick when I was eight years old, hard enough to black out.

# case 0049

Even though I'm a senior citizen, my senses of smell and taste were just fine until I received a concussion (blow to my forehead and back of head) in an accident almost four years ago. Since then, I cannot smell or taste anything. I was well-known for my cooking and the recipes I created. One of my greatest pleasures was cooking for friends — having them over for dinner. Now I can't do that because I can't season the food. When I eat, I try to remember what each food tastes like as I take each bite or sip. But I can't taste anything. The only reason I eat now is to relieve hunger pains. I get no enjoyment from eating. I can't smell rain or snow, or my flowers, or my dog. I can't smell clean laundry or fabric softener. I can't smell hairspray or permanent solution. I don't know whether my house stinks, or whether I stink. I can't smell chemicals, except once in a while I get a whiff of chlorine when I turn on the tap. One good thing about losing my sense of smell: I don't smell dog waste when I pick it up. I also had potentially very dangerous incidents involving gas that I could not smell. So much pleasure is lost when one loses his/her sense of smell. I used to love to smell the coffee brewing in the morning, or apple pie baking. I love the fragrance of the flowers in my garden, or the "smell" of autumn in the air. I can't smell freshly cut grass or hay. I used to be able to smell an approaching snow storm. There's a difference in the fragrance of impending rain and snow, and I could smell that.

# case 0050

When I am inactive, like watching TV or sitting at the computer, I smell cigarette smoke and it makes my throat tighten up just as it does when exposed to the real thing. I was even thinking there must be a homeless person hiding in my basement... I do not smoke, nobody I live with smokes, I do not have friends who smoke; it's a mystery. Sometimes, this goes away for weeks at a time....and then it returns.

# case 0051

One day I snorted what I thought was cocaine that was given to me by a stranger. I think it may not have been cocaine since I felt nothing but an itch in my nose that was followed by incredible pain in my upper nose. After three days I still couldn't breathe normally but it got better after five days. A couple of months later my partner noted that I can't perceive any odors anymore. Flowers are odorless for me and I don't enjoy the smell of cooked meat. Many things have changed in my life.

# case 0052

My sense of smell within a few months' time turned to always smelling a bad distasteful smell. I first noticed it in my bedroom and then began to associate the smell with my partner first and then with myself. I have washed everything and cleaned, changed soaps, done everything I can think of but the smell seems to now follow me around and I hate it! Even when I use perfume I can still smell it. My partner and I have a good relationship. I have this nagging feeling that I am sick and that is the reason I have this bad smell around me, but I have no basis for it. This has been going on for over a year. I have not told my partner because I did not want him to think he smells bad to me and he has never mentioned that I have a different smell either. I am hoping no one else can smell it but I am not sure. It is starting to drive me a little crazy.

# case 0053

I have been a welder for decades and I have noticed several coworkers that have lost their sense of smell. I am thinking it was from my work environment: diesel, welding fumes and grinding particles in the air. I started losing my sense of smell about the age of 45 and now I can hardly smell at all and have very little taste perception.

# case 0054

Over a year ago I had an accident which resulted in head trauma. It was only later that I was diagnosed with anosmia. No one has attempted treatment. I was told that unless the nerves regrow on their own there's nothing that can be done. My faith in the medical profession has been damaged. Not one doctor seems to take my loss seriously, not even the neuropsychologist. So far all I've heard is "if you have to lose one sense, smell is the easiest". But I feel like someone who is dealing with an invisible disability. I even saw a psychologist and he told me that I should join a support group.

# case 0055

More than a year ago I used nasal spray but the strong odor was very irritating to me so I stopped using it after a couple of doses. I continued to smell the medication for several weeks on a regular basis, then I went on to smell it occasionally for many more months. It smelled like a pungent burst broke loose in my sinuses. A while later I began smelling a sickly sweet chemical smell on and off. At first I attributed it to the heating, ventilation, and air conditioning, but I smelled it in many different settings. I thought I was somehow carrying it from my office everywhere else on my clothes or body. Later when I no longer worked there it finally dawned on me that it couldn't be my office. Next I thought it was my refrigerator motor, again with my clothes picking up the stench. At this point the odor took on a smoky quality. When I asked my family if they smelled it they would say they could smell something slight coming directly from the motor component, then they would say I was really sensitive to smells. I was concerned for the refrigerator, so I called a repair man. I don't think the refrigerator repair person appreciated my request to figure out what the cause was. He couldn't smell anything out of the ordinary. About a week later I went to a perfume store and found that all the scents smelled exactly the same and very toxic, a cross between ammonia and alcohol. I also noticed that I seemed to have a much reduced sense of smell in general. For example I tested eating a bite of raw onion, something that I normally would never eat alone. It tasted watery and only slightly like onion and had no strong odor. With this new stuff I finally figured out that the fake smells must be me, not something in my environment, and that something is up.

# case 0056

I was a smoker for more than 20 years. When I gave up smoking I saw a remarkable difference in my sense of smell within a month. My sense of taste and smell were fantastic.

# case 0057

Within the last 6-8 months I have noticed that the air smells like ash... somewhat like a cigarette ashtray. I quit smoking two years ago. At first I thought it was the fireplace that needed cleaning but after it was cleaned the whole house smelled ashy. Next I noticed that the outside air smells ashy as well.

# case 0058

I lost my sense of smell and taste gradually three years ago. I was using nasal spray for congested sinuses at the time. I do smoke cigarettes. Doctors have said this is the reason for anosmia in my case, but I've been smoking since I was a teenager and was always able to taste and smell. I've been to many different doctors and got very different answers and treatments. There has been no improvement in this condition that really has messed up the quality of my life: Imagine not being able to smell a delicious meal, or the lovely scent of morning/the ocean breeze/mowed grass. Maintaining regular meals is the most difficult, as is the depression that accompanies a poor diet. I never considered losing the senses of smell and taste would have such a debilitating effect, but it certainly does.

# case 0059

I was involved in an accident over a year ago. I suffered a traumatic brain injury and have since lost my sense of taste and smell. This has affected my life dramatically. I no longer enjoy cooking, eating out with friends and numerous other things. I've become extremely depressed and I would do anything to get it back.

# case 0060

Two months ago I was involved in a car accident. Three weeks after the accident I started experiencing a weird smell.... out of the blue. I'm not sure that I've correctly identified it, but I think it's the odor of the gray smoky powder that filled the car when the airbags deployed. Several times each day the smell fills my nose, without any particular thought or activity. I might be reading, watching TV, doing things around the house. I did not have a head injury, although my neck ached for some time after the accident. It is a disconcerting thing!

# case 0061

After a barbecue during which everything smelled and tasted just as it always had, I was watching a movie when I thought I smelled smoke — to the point where I went outside to see if the grill had been properly distinguished. The smoky smell came and went over the next 24 hours, and when it was finally gone, so was virtually all of my sense of smell. When, after a month, it hadn't returned, I saw my doctor. She diagnosed a sinus infection and prescribed antibiotics which did clear my nose but did nothing for my sense of smell. She referred me to an ear nose throat doctor for further examination. I was advised to wait and see if my sense of smell returned — and warned that the odds of that happening weren't all that great. I'm not entirely without a sense of smell; I get occasional whiffs of actual things in my environment — my shampoo, food, that cat's litter box — but they come and go, and even when I can smell things, they don't smell right. Citrus, for instance, smells vaguely chemical; anything cooking smells like toast. I can tell when I'm smelling perfume, but not what scent it is. Other smells, even strong ones — garlic, for instance, or bleach — are completely absent. As someone with a lifelong love of cooking and eating, I've experienced this as a serious loss. My appetite has dwindled, and I've lost about 20 pounds since last summer; I've had to refocus my cooking on the taste bud flavors (sweet, sour, salt, bitter, umami) and the painful ones (cayenne, horseradish, etc.) to find anything appetizing at all. I wind up throwing a lot of food away because I can't detect if it's spoiled. My previously healthy libido is also affected, and I've found myself experiencing periods of situational depression (I have never been chronically depressed.) All in all, it's not much fun, though I hate to complain when I'm otherwise healthy as a horse.

# case 0062

Last year I had the flu. Since then, I have had no sense of taste or smell except for the smell and taste of metal. The doctors say that there is nothing they can do. I am devastated and don't know which way to turn. Everything I eat and drink tastes of metal sometimes stronger than other times. It makes me feel sick. I'm thinking of seeing my doctor for some antidepressant because I feel so depressed and miserable.

# case 0063

I don't remember ever having a sense of smell, but sometimes I look at something and an odor memory comes to me. I assume it is because I could at some point smell. I can detect very strong odors, however, they coat my mouth and I taste them. I could walk into a perfume store and be hit with a nasty taste in my mouth, but never be able to say, "that's a vanilla perfume". It is depressing to never smell a flower or my babies. It is also scary to know that there could be a fire right under me and I wouldn't know until it was too late. It makes me angry to know that people don't consider anosmia a real disability. People forget about my condition and shove things under my nose "Here, smell this... oh.... yeah, oops". But alas, I have lived with this for as long as I remember, so I have come to accept it by now. Besides, what if I got my sense of smell back, and couldn't stand the scent of my partner?!

# case 0064

Ever since I was born I noticed something is weird. I remember that when I was four I realized that I can't smell and thought about telling somebody but then decided against it. I even remember me pretending having a sense of smell, although I don't know why. Only when I grew up it started bothering me again. I heard about anosmia for the first time on a TV show. Since then all became much clearer to me. Finally, someone performed some smell tests on me and I now have proof that I can't smell anything. For now, as weird as it sounds, only my family knows about that, not even my closest friends or extended family.

# case 0065

I smell caramelized sugar. Three months ago I was at a friend's house while they were making fudge. The smell of burnt sugar permeated the house. When I came home I continued to smell sugar. I still experience that smell. It's not constant but at times it's a strong smell. I did have a cold with weak flu like symptoms prior to that visit but was feeling good. The smell disorder does mask some odors but I can smell coffee, soap, but most odors are barely recognized. It's starting to get me concerned, what could it be?

# case 0066

I lost my sense of smell five years ago and have not smelled anything since. I first went to the doctor when I experienced a strong smell like Christmas: oranges and cloves mixed with mulled wine. This was in the middle of July so there was obviously something wrong. My doctor said it was an infection and prescribed the antibiotics which did get rid of the Christmas smell but it was then that I noticed I could not smell anything. I used to work in a pub and was told my condition may have been caused by the line cleaner or gases used, but tests at the hospital showed that nothing was wrong with my nasal passages or brain; just a perfectly healthy nose with nothing wrong except it was not smelling!!! The doctor gave up on me. I had to give up my job in the pub because of my condition. Many times I have been ill with food that looks okay but is actually out of date or gone off, milk especially! I have realized how much smell affects your daily life and cannot believe the lack of research into it, considering it is one of the five senses. It is definitely the least understood. I have tried zinc, vitamins, cutting out various food groups, and nasal sprays with no result and feel I am at a loss as to what to do next! The doctors do not help as they do not know how to and there is very little research on the problem!

# case 0067

I've never had a sense of smell. When I was little I used to pretend that I was able to smell because I thought I had to be able to "learn" how and I just wasn't good enough at it yet. I recently took a comprehensive smell test at the doctor's office and was diagnosed with anosmia. My identical twin sister is not anosmic.

# case 0068

I have chronic obstructive pulmonary disease. I have had a bad smell in my nose like a smoke or insecticide for about three months now. It seems to be worse at night. I can go outside and the smell is not there. I sprayed bug spray in my bathroom for ants and my oxygen concentrator was in there. I had it replaced but the smell is still in my nose.

# case 0069

Three years ago I suffered a series of colds/flus/sinus infections. I noticed when these all eventually cleared up, that my sense of smell and taste had diminished. In the following months, the deterioration continued. I now have virtually no sense of smell and a very diminished sense of taste. I saw an ear nose throat specialist and was told, more or less, "tough luck, it is possible that your sense of taste and smell might improve, but there are no guarantees and you may never get them back." When I asked if there was anything I could do to improve the situation, I was told there isn't.

# case 0070

About a year ago I started smelling a strong unusual smell that I had never smelled before. At first I thought it was my urine. Then I thought it was my cat that had an abscess. I started smelling it more and more. I thought it was from the cat sleeping on the couch or my bed. I bought a rug shampooer and shampooed my carpets, upholstery, and washed all the linen in the house, but the smell continued. Then I started smelling it at work and on the bus; so I figured it was me. Recently, I had the nerve to start asking people if they could smell it on me. No one could. I started researching olfactory hallucinations. This smell was also associated with the sensation of menthol in my nose prior to the strong smell (but without the menthol smell). In the last two days I have noticed a loss of smell. Today I noticed I cannot smell perfume, lit matches, candles, basil or basically any smell in my environment. If the strong smell that I originally smelled a year ago disappears, it might not be all bad.

# case 0071

I don't know if I've been anosmic from birth or if it's something that happened to me when I was very young, but I can't remember any period of my life where I actually was able to smell. My parents never noticed that I couldn't smell, so I'm not sure if I pretended to be able or if I actually was able. However, when I was six years old I apparently came home from school and asked my mother how freshly baked cinnamon rolls smelled like. We'd baked in school, and everyone was so excited about the smell when they were brought out of the oven and I instead got sad because I couldn't feel it. My mother got surprised, because she had absolutely no clue about this condition before that. We went to the hospital to check it out, but with little result. I was asked to smell several different things while being blindfolded, and I couldn't smell anything. The result was however that I was a stubborn child who lied, so not much more was done. After that I've never been able to smell. I'm 21 years old today and I get an itchy feeling in my nose when I cut garlic for example. I can feel that it's a very, very strong smell, but it's more like it's a taste stuck in my upper mouth rather than a smell going through my nose. I can stand right next to a fire and won't be able to smell the smoke. I regularly both cook and bake and besides onions and garlic I never pick up anything else. I could never smell the fertilizer in the country side. I don't know if this is any help at all, but since I myself really feel left out when other people are talking about smell, and don't really have a clue about what's wrong with me, I just wanted to contribute in any way I could.

# case 0072

I had sinus problems since I was a teenager. My sinuses would sometimes bother me with congestion and poor smell. When I went to the doctor I was diagnosed with rhinosinusitis and poor sense of smell. I cannot smell or taste almost all of the time and when I can it is poor. Different medications had no effect and after surgery to remove polyps and reduce turbinates I could smell and taste for three and a half days, and then I lost it again. It was the same as before surgery. I think this should be considered as serious as losing one of the other senses, but it is not. It is so frustrating and how it has affected my life emotionally and the choices I have made has to be negative.

# case 0073

A year ago I got the flu with sinus symptoms. As I started feeling better I realized that my sense of smell and taste didn't return. I had surgery eight months ago to remove a turbinate on the right side. At times I have fleeting smells, and I can tell if something that I eat is mentholy, minty, salty, sour, or sweet, but I can't really identify tastes or smells. I would travel to see a physician who treats anosmia, but I don't know of anyone who takes this condition seriously.

# case 0074

I have never really been able to smell. I can smell things like ammonia and bleach but it has to be right under my nose. It's very frustrating not to have this sense. I don't tell people outside of my family that I'm not able to smell because I am embarrassed. As a nurse I know this is probably more common than one would expect but I have to say it almost makes me feel ashamed and left out that I am not able to smell what a sugar cookie, a barbeque, or a rose smells like. My partner tries to describe different smells to me and I just try to imagine.

# case 0075

I was diagnosed with allergic rhinitis due to allergies but still maintained a sense of smell. However, after the birth of my first child I had a sudden and total loss of smell. I had no cold or other illness to prompt this and can think of no reason for this loss? I did have a sense of smell the day before my baby was born and none the day after! The only thing which has ever had an impact on my sense of smell has been steroid tablets which were prescribed for the asthma I also suffer from, but this relief is as short lived as the prescription. Now I continue to suffer from smells which I feel are "trapped" in my nose. The smells change every so often and this badly affects my sense of taste. These smells range from a burning smell to a metallic smell. I would badly like to recover my sense of smell.

# case 0076

I suffered from sinusitis for about 18 years. Suddenly, about five years ago, I found that I was absolutely cured. However, for the past few years, I found that I have lost my ability to taste or smell. This condition usually lasted for about two weeks and then it would be back to normal. However, now it has been more than seven months and things are getting from bad to worse. I cannot taste or smell anything at all, despite the fact that my sinuses are totally cured.

# case 0077

I have probably never been able to smell. When I was younger, I would pretend to be able to smell, and when someone announced that they smelled cookies, I would just go along and say, "Me too!" I had always figured a sense of smell was something that developed as you got older. For a long time I wasn't really sure if I had a sense of smell. I misinterpreted nasal irritation or the feeling of warm air in my nose as smells. However, I could never identify any smells and in in psychology class in college I learned that the sense of smell is not actually felt that way. I have also problems processing words and hypothesize that my difficulty in hearing is connected to my lack of sense of smell. I once burnt popcorn in the microwave and now I am worried about not being able to smell smoke. I also wonder what my sense of taste would be like, if I could smell. I can tell the difference between an apple and an onion: onions are more celery-like in texture. There are advantages, of course. Aside from pity and visually caused disgust, there is no reason for me to take particular note of road kill skunk. Overall, I regret not being able to smell, although it makes for a good conversation topic when I make a rare attempt to socialize.

# case 0078

I was exposed to the fumes of burning plastic and one month later I kept smelling smoke and ash on my arms and in my nose. I have developed a cough that won't go away. I have been to two different doctors many times. They gave me prescriptions which had no effect. It has been six months with no improvement. I have not been able to taste or smell since. Actually, I can taste and smell but nothing smells or tastes like what I am eating. Most things taste awful and nothing tastes like what it is. I know this is really strange and downright weird. I am not a crazy old woman... I wish I were.

# case 0079

I have always had an extremely heightened sense of smell. I could not go to a friend's house if they had a cat because the smell of cat litter hit me as soon as I entered the front door, yet no one else (including other friends visiting at the same time) even noticed. I have always been able to smell things at levels that are undetectable to others (except the occasional pregnant woman with heightened sense of smell). I could smell french fries in a car in front of me driving 65 mph down the highway in winter. I could smell cigarette smoke from four cars away. I could smell subtleties in aromas (wood, chocolate, carpeting, and just about everything) that others couldn't smell unless it was overwhelming. All that changed three months ago. I had flu-like symptoms but without any nasal congestion or stuffiness. I also had the worst headache I've ever had for eight days. The doctor thought it was the flu and told me to rest. Now, my sense of smell is much worse than it was before. I can still smell very faintly some very strong odors: when my baby poops, I can no longer smell it until I have actually taken off the diaper. And it's very faint. I used to be able to smell it from another room. I can no longer smell aromas such as food cooking, pizza, flowers, spices and others. I can no longer smell the mercaptan (gas-odor additive) when I turn on the gas stove. I worry now about our safety. My doctor first said "You're lucky! You won't have to smell the diapers!" When that upset me, he replied that loss of the sense of smell was "no big deal" and I would "probably get it back in a few weeks". That was more than a month ago and there has been no improvement. It is more terrible than I could have imagined to have lost almost all sense of smell, especially since I had had an incredibly sensitive and heightened sense of smell my entire life until four months ago. I can no longer tell if food is spoiled, if there is a gas leak, if the pancakes are burning, if the house is on fire — not to mention all of the pleasant aromas that make life enjoyable!

# case 0080

At 37 I noticed that I had problems smelling my grandson's nappy. Within two years I would not be able to smell any bad smells. I went to my doctor who poo-pooed it. By the age of around 45 I was starting to lose some of the nice smells, such as mown grass and some perfumes. I went to another doctor at 48 and got an examination and a brain scan that didn't show any underlying cause. My sense of smell disappeared completely a year after this. I am now 53 and cannot smell a thing. I hate it because it is like living in a plastic bag.

# case 0081

I passed out from paint fumes when I was a child. When asked why I didn't leave the garage when I smelled the fumes, I told my parents that I was "still working on smell." I thought it was something you learned at school. I have no sense of smell and doctors found no physical cause for it. I assume it's congenital.

# case 0082

My sense of smell and with it my taste went very suddenly after a cold and a series of violent headaches which I put down at the time — possibly incorrectly — to blocked sinuses. What also may be relevant is that at the time I caught the virus I was trekking in in very high mountains — over 13,000 feet. I once had an extremely developed sense of smell and could taste herbs and spices in food well enough to tell you what was in them. I also loved perfume. I can no longer smell perfumes nor taste food. I went to a specialist who confirmed that I lost my sense of smell and told me that there is no cure. Losing my sense of smell was a huge blow, smells evoke memories and enrich life. Life seemed very empty when there was nothing there, like I was living in a box and looking out at the world.

# case 0083

About three years ago I noticed that whenever my TV was on or whenever I was near a light turned on or whenever my forced air gas furnace was on I had a smell of burned wood. The smell got worse when I changed TV channels.

# case 0084

On occasion I would smell a bad odor. There was no evidence of a "real" odorant, as nobody else smelled anything. I was very persistent in that it was something "outside" of myself that smelled bad. I had our water checked, drains checked, changed laundry soaps, body soaps, etc. At first the bad smell would come and go but later it began to linger longer and longer. Finally, it became almost permanent. Now, the smell is worse than ever. Warm aromas smell bad, the cold interior of the refrigerator, the freezer, the blow dryer... Now I cannot smell things I used to be able to smell like peanut butter and coffee. Later I also lost my sense of taste and the bad odor persisted! I cannot taste sweet, salt, bitter...nothing! Sometimes, I can detect a different taste between foods, but cannot tell what it is I'm eating. I have lost 11 pounds since this began. The odor is so bad that it makes me nauseous at times.

# case 0085

After a concussion I noticed that my sense of smell and my perception of what things I liked to smell and taste changed. I went from strongly detesting the flavors of certain foods and flavors to enjoying them. Foods and drinks that I had liked the smell of, but not the taste of, prior to my car accident suddenly became not only things I enjoyed consuming, but my favorite things to order. When I go to my coffee shop they know right away to make me a chai without my ever ordering it, because it's the only thing I get. I hated the taste of chai before my accident. Smells that I did not like before suddenly seemed pleasant to me, and some of my favorite smells from before were generally less appealing to me. I tossed out some of my perfumes, and bought some new ones. I started trying new foods, and forgetting old ones. Embracing these changes and looking at them as an opportunity to discover new things to love was really the only way I could handle the situation without it upsetting or confusing me, since my brain checked out okay on scans and my doctor did not really know what to tell me about it. Certain elements of my personality have changed as well, but the one that has been the most quirky and the least troublesome has definitely been the change in my senses.

# case 0086

I had a severe bout of flu with a very heavy head cold. This was some six months ago and my sense of smell and taste have not returned. I went to the doctor after about a month but the prescribed medication did not help. As a cook of small talent with a nose for fine wine and a moderately convivial palate developed over the years, I would be most grateful for any feedback on how this divine retribution for a lifetime of over indulgence might be reversed. I have lost weight which is good but something of the zest for life is lacking.

# case 0087

I lost my sense of smell as a teenager. Initially, I put the anosmia down to having a cold thinking it would reappear within a few days but 21 years later, it still hasn't returned, and I seriously miss it. My sense of taste is perfectly fine, although I did try a taste test where my husband blindfolded me and told me to guess what five sauces he put into my mouth were. I didn't get any of them right, yet I know what chocolate, coffee, lemon and strawberries all taste like with my eyes open. When I was 25 I had surgery to trim away the nasal lining of my nose, but sadly the sense of smell hasn't returned. There is definitely something socially embarrassing about anosmia and there really needs to be more research done into it. What I would give to be able to smell again...There's definitely a feeling of being ashamed because I can't smell and a fear that I myself smell and don't know it. I'm forever using more deodorant, mouthwash, air freshener because I'm so paranoid that I can't smell myself. My husband has to be the one to tell me if I do. I can remember some smells but I truly miss being able to smell things like laundry, flowers, perfume, my husband, bread baking or cooking smells. It made me sad that I couldn't smell the flowers at my own wedding reception. Equally, it's a real pain when I can't smell nasty smells either. The number of times I have to ask my husband if the house or my breath smells okay or he'll tell me that the dog's messed on the floor and I've not realized it, or I've burned toast again because I haven't picked up the first traces of smoke, leaking gas or the smell of sour milk or gone off food. It worries me that one day if I have a baby I'll not be able to bond with it as I've read that the smell of a newborn and mother is supposed to be integral in the bonding process or smell its dirty nappies, although this may be a good thing.

# case 0088

Some time in third grade I realized I could not smell anymore. It might have been caused by a playground accident where I hit the top of my head against a bar extremely hard; I remember having the worst nosebleed of my life and feeling extremely disoriented. Interestingly I can occasionally smell things (1-3 times a month) but sometimes I smell odors others assure me are not there. For example I recently smelled strawberries for several minutes though no one else did and I could not find any evidence of strawberries anywhere nearby.

# case 0089

Around the time I was 35 I developed allergies and polyps. These were removed by an ear nose and throat doctor and for the first time in three years I could breathe nasally. However I have no sense of smell — I literally cannot smell anything. But there are two exceptions. First, I smell my body odor very acutely and find it disturbing. I constantly wash myself and apply fragrances (although I cannot smell them). Despite these efforts I still smell myself. I worry others smell my body odor as I do. Second, I experience snap shots of smells. For a very brief moment I smell something and comment on it but then within an instance it vanishes. This occurs at odd times, for example if I watch a cooking show on TV I will sometimes smell the cake being removed from the oven. I know this is not possible and put it down to an olfactory mirage or memory. The experience is vivid and enjoyable.

# case 0090

My sense of smell is really bad; it always has been. However, I was burning hand sanitizer one day and I could suddenly smell really well for a few days. I have done this a few times now and the result is always the same.

# case 0091

I am unable to smell or taste food! I did not realize how miserable it is not to be able to smell odors or taste food. One of life's greatest pleasure is being able eat a great tasting meal. Taste and smell is two of the five senses of the human body! I have felt like I am just existing and not really living! I would, if given a choice, rather have this than to lose my sight or hearing or sense of touch. I seem to get glimpses of smell every now and then as well as taste. Even foul smells would be welcome. Do I sound desperate? It's because I am!

# case 0092

I lost my sense of smell when I had a brain injury two years ago. I want it back so much that I cry sometimes. I get bad tastes in my mouth too, especially after sleeping.

# case 0093

I don't remember when I lost my sense of smell. But I remember that even when I was 13 years old, I could not smell. Sometimes it's hard for me, when I wake up and want to go to work, I don't know how my clothes smell. But I deal with this by changing my clothes regularly and showering daily. I have no major limitations. My condition does not bother me. If it could be cured, I would not do it. I enjoy my anosmia. Sometimes my friends say to me "it stinks here" and I say "I've noticed, the air is warm and I breathe more heavily".

# case 0094

Usually in the middle of the night, I smell something burning and I have to see if the house is on fire. I also smell brewing coffee. I always think my husband woke up real early to make it but that never turns out to be the case. I also have been smelling a sweet burnt smell on my body but if I put my nose to it there is no scent. My husband smells nothing. I'm not crazy.

# case 0095

A few days after my prostectomy surgery I realized a clear change in my ability to smell and taste specific odors. I started to find citrus odors (orange, lemon, and grapefruit) very distasteful. I also found what used to be my favorite beer distasteful and switched from Pepsi to Coke. I could no longer smell distasteful locker-room odors. However, over the last five years some of my smell and taste abilities have slightly recovered.

# case 0096

For the last six months I am smelling only a smell like burning vegetable oil. The smell is so strong that I use a mask, but it is of no help. I tried to smell other smells, but I can't. I am so hungry because I can't eat any food, even without oil. Everything smells the same. My life is hell. An ear nose throat specialist found nothing wrong with me.

# case 0097

I've always been sensitive to strong perfume scents and have had bouts of sinusitis throughout my life. Four years ago I started smelling strange smells in my house that no one else could smell. Then I started noticing that my favorite perfumes were smelling bad and were irritating to my nose. Gradually, every scent in the house was irritating my nose and it was even hard to sleep at night as these weird scents were assaulting my nostrils. The strangest thing I've found is that I can fry chicken, steak, etc. and can't even smell it cooking. But I can still taste it and it tastes good. The most irritating part is that when the distorted smells are the worst, I even seem to have an unpleasant chemical taste in my mouth. My mouth is also always very dry and I constantly chew gum for this or to mask the chemical tastes. I try to ignore these things but it does get depressing, especially since I can't enjoy the scents of my favorite colognes or just the simple smell of a rose or the baby powder on my grandchild. I have been to two ear nose throat doctors to no avail. Some days it seems worse than others. Going outside in the fresh air helps but once I'm inside a building or around people, my nose starts picking up strange, unidentifiable, irritating scents and the mystery continues!

# case 0098

I had knee replacement surgery two months ago and now I cannot smell certain smells and I have a slight metallic taste in my mouth.

# case 0099

I had a head cold 40 years ago and lost my sense of smell as a consequence. I had three surgeries for nasal polyps and used several nasal sprays and drops. All this time my nose was blocked most of the time. The only relief was when I was prescribed prednisolone. After a few days my sense of smell returned and my nose was back to normal. Because prednisolone is only prescribed for one week in 5 mg tablets the effects soon wore off after a week or so. My nose has been clearer for the last seven years, but no smell.

# case 0100

I had an accident and the back of my head hit the road. Four days later I realized that I could not smell anything, when I was carrying a bottle of bleach and it felt damp, I assumed it must be water on the bottle as I would have smelled it if it was bleach, but when I saw my coat was white where it had once been grey I realized it was bleach and that I could not smell. Over the last three years I have regained some of my sense of smell in that if there is a strong odor I will know that there is an odor but not usually what it is. It often smells unpleasant. Taste has been dramatically affected and so has my enjoyment of food and cooking. My life is far less rich and my enjoyment of things (food, going to the seaside, pretty much anything) is often greatly reduced. You don't know what you have until it is gone!

# case 0101

I suffer from allergic rhinitis and had several sinus surgeries. 15 years ago I lost my sense of smell. My sense of smell now comes and goes. When I take steroids my sense of smell comes back. The steroid is working like a wonder drug.

# case 0102

I first noticed an odor which I thought was in the house from cooking, etc. When it did not go away I realized it was inside my nose. It smelled like smoke laced with bleach. I even walked up to a lady at a gathering and told her I thought something was burning in the kitchen. Nothing was burning. After about a month of hoping it would go away, I went to an ear nose throat doctor to no avail. The odor is as strong as ever. It is very upsetting to have this odor continuously. I don't know if it is my imagination but it seems to be a little more pungent in the afternoon. Not a soul I have spoken with has ever heard of such a thing. I feel really alone in this!

# case 0103

I never have had sense of smell. I call it the hidden handicap. It's difficult at an early age.

# case 0104

I have no sense of smell. However, when I take prednisolone for my asthma within a day I can smell and taste again, but once the dose is completed, I lose the senses again. I have also had occasions where after eating garlic I have had a slight sense of smell. I would dearly love to be able to enjoy smells and food again.

# case 0105

I quit smoking five years ago and my sense of smell returned in all its glory within three years. A few months ago l started to smell a burnt smell. I thought it was coming from wood stoves that some people have in their homes. The smell was strongest at night. It was very strong in my living room. Every time I got close to my leather couch it was really strong. I smelled the leather and sure enough it was my couch. My friends say that the couch just smells like leather to them. But to me it gives off a burnt smell. Now that burnt smell can hit me almost anywhere: in stores, restaurants etc. Sometimes it is overpowering and makes me feel nauseous. It is an acrid, burnt smell. I don't understand why I am the only one that smells it and everyone else smells leather.

# case 0106

I smell cigarette smoke and no one is smoking but I also actually have the feeling of breathing it in, feeling it in my eyes, and getting a slight headache in the front of my head. It happens more when I work out and when I relax or try to sleep. Sometimes it is stronger than other times to the point of almost taking my breath away.

# case 0107

I was addicted to heroin for several years. When in withdrawal my sense of smell came back to the point of making me sick (I had forgotten how bad the world really smells). I am now completely free of opiates but since detoxing I have a strong sense of smell again even though I smoke a pack a day of cigarettes. I have been told by other opiate abusers the same kind of story.

# case 0108

My sense of smell was very sharp but after a cold I lost it. I couldn't smell coffee, or garlic, or anything else. Doctors, even ear nose throat doctors, didn't see anything to worry about and had no suggestions. I occasionally get flashes of smells, an orange or some spice, but most of the time I smell nothing at all.

# case 0109

About eight years ago I lost my sense of smell. I became aware of it only gradually, thinking at first that the flowers were too cold to be fragrant but when I could not smell anything at the perfume counter I realized the sense of smell was gone. However, my sense of taste is as sensitive as ever! I thought taste goes when smell goes. The only thing I ever smell is a faint aroma of coffee now and then.

# case 0110

For the last year I have had absolutely no sense of smell and very little sense of taste. I cannot smell smoke, burning oil. I have burnt food on the stove because I left the room and did not know that it had boiled dry even though the smoke was going through the house. I have mentioned it to my doctor but he hasn't even addressed the issue. I don't know what to do about it. It is depressing.

# case 0111

I hit the ice with my head after a fall and went to the hospital because of concussion symptoms. After 10 days or so I noticed that a drink did not quite taste right and soon after wondered if I was smelling anything. I picked up a ripe banana and couldn't smell a thing. I couldn't smell orange, garlic, or perfume. My taste seems fine, or at least not bad. Everything I read suggests that taste leaves with the smell so I can't narrow my symptoms to a cause.

# case 0112

On a flight about a year ago a batch of savory filled paninis were overdone and the very strong smell hung around in the cabin for a long time. I found that the smell seemed to be imprinted on the inside of my nose after that and it was all I could smell for a while. While on this trip we visited a couple of wild penguin colonies and unfortunately the smell of the guano was also very strong and also imprinted itself on my nose! For weeks these two smells seemed to alternate. They gradually faded and left me with either the scent of scorched linen, or the smell of cheap fruit jam, as found in a disappointing jam doughnut. I can't smell flowers, grass, perfume, coffee, etc. apart from a very fleeting and faint impression. Sometimes I notice the two smells above to an irritating degree, at other times they are fainter.

# case 0113

I started losing my sense of smell in my twenties, and it has gotten progressively worse since then. A year ago I could still smell strong things like gasoline but now I can't smell anything. My doctor tells me there is nothing to be done.

# case 0114

Twenty-five years ago I broke my nose. Afterwards, my taste and smell decreased slowly. Now I cannot taste anything nor can I smell even a skunk or a fire. I have occasionally had a tease of a smell or a phantom smell that no one else can smell (usually a very bad smell). I have a super life but find a part of life that most people enjoy is missing. I can cook very excellent meals but need testers to check seasoning. I find it very upsetting to have a wonderful meal such as a steak or lasagna and all my family or friends exclaiming how wonderful it tastes. I could eat cardboard and not know the difference. Everyone gets me to change my grandson's diaper when he has a bowel movement, although I can't tell when that has happened so I have to check all the time. I find this condition upsetting and dangerous.

# case 0115

During an episode of the flu I lost my sense of taste, which I considered normal for the flu. A few weeks later I noticed that I could not smell even though my nasal congestion had subsided. My sense of smell has not returned in the year since. Prior to the flu, I had a very acute sense of smell. I visited an ear nose throat doctor and am currently in treatment, however, the doctor thinks that my smell sensors were attacked by the flu virus and that I most likely will not regain my sense of smell.

# case 0116

About three years ago I started smelling diesel fumes which nobody else around me seemed to notice. This went on for about two years and I started to think that I was just oversensitive to fumes so I tried to live with it. Then, three months ago, I stayed for a few weeks in a home with a fryer that was giving off an awful chip fat smell (at least that was how I perceived it) and since then I have this smell with me everywhere I go. Until a few days ago I thought I was smelling of chip fat. I thought it was in my hair and on my clothes. I kept putting my clothes in the wash even before I wore them, kept washing my hair, really scrubbing it, and when I still smelled it, I sprayed my hair with deodorizing body spray. Whenever anyone came close to me I would pull away, conscious that they may be smelling "chip fat" on me. I then realized that there are times when I didn't notice the smell so I asked a family member to smell my hair. She said all she could smell was shampoo, which surprised me. I described what was happening to me and she assured me that there wasn't even the slightest whiff of chip fat. I visited my doctor today and told him about it and he explained the possible causes and prescribed a steroid nasal spray.

# case 0117

I had a traumatic brain injury seven years ago. I made candles for a living and I noticed that my sense of smell was gone. I could not really make a distinction between the scents for a long time... Later on, my sense of smell began to come back.

# case 0118

Two years ago I started smelling burning wood whenever I was near my TV, laptop computer, under a light, or when my furnace was turned on.

# case 0119

Seven years ago I was diagnosed with anosmia caused by "nasal block". Nasal polyps were surgically removed but the condition did not improve. I haven't ever smelled my kids! Very depressing to begin with but you adapt (but can never forget what you've lost...).

# case 0120

I experimented with cocaine. Now I have a reduced sense of smell. It's not as full and rich as it used to be.

# case 0121

I have no sense of smell. Now, in my early thirties, I am coming to the realization that not being able to sense smells in my everyday environment is really almost like a handicap for me. I cannot smell food when it goes bad; I regularly have to check the milk in my fridge by tasting it. I cannot smell the cat's litter box when it's time to change it. I cannot smell the perfume I bought my girlfriend. I have to trust other people for their idea of what smells good. I depend greatly on the name of deodorants. When my children were young, I couldn't smell when they needed to be changed. My regular physician doesn't seem to make an issue of my inability to smell. The only person that ever really expressed interest in it was a counselor (therapist). She considered trauma but I never had head trauma. I am confused and feel hopeless.

# case 0122

I had the flu and when the symptoms disappeared I noticed that I couldn't smell anything other than astringent smells (ammonia, cleaning liquids, perfume, artificial flavorings, etc.). My sense of taste was affected in the same way. Six weeks after the flu, an ear nose throat doctor diagnosed it as anosmia and said that I will never be able to smell again. I was treated with antiviral medication and steroid drops and my sense of smell has returned somewhat. The sense seems to be better at times and then will disappear completely again. After a while it would appear again. It seems that the sense varies to what type of smells I can detect at different times. Certain smells (like garlic) which I can detect are completely distorted. Others seem to be okay but very slight. Organic smells seem to be the most elusive. I can't detect body odor or feces at all, for instance. This has now been going on for eighteen months. Some days I'm confident that it is improving, just to be disappointed again the next.

# case 0123

My loss of smell and taste has been gradual. I noticed two years ago that I could not smell the scent of flowers. Lately I cannot smell or taste coffee or other flavors. I really enjoy a cup of coffee in the morning and now it is like drinking hot water.

# case 0124

The only treatment that I found effective for smell loss was prednisolone to reduce the swelling of the membrane followed by the use of drops to keep the membranes from swelling again. During this time my sense of smell returns. All other treatments were ineffective. After prednisolone administration, my sense of smell returned within 48 hours and stayed for about three weeks until the prednisolone wore off.

# case 0125

After a normal cold my sense of smell never recovered and I am really depressed about it. It appears that the authorities do not consider it an impairment, but I do. I can't smell if the gas is on and I can't smell something burning. I am very conscious of social gatherings because I don't know whether I smell too much. Before all these I had a better sense of smell than an average person. Now I feel disabled.

# case 0126

For the past five years anything like urine, coffee, and other things with a strong smell all smell the same inside my nose. It smells like rotten flesh. No one knows what it is or cares. I just live with it but it is not pleasant.

# case 0127

Three months ago I had my gall bladder removed. Since the surgery I have experienced phantosmia. Mine is the smell of cigarette smoke which of course doesn't exist. It is only experienced in air conditioned environments. If I go outside in the warmer air, I do not experience it. It is such a strong sensation that sometimes it makes me sneeze or cough. No one else in the room smells the smoke smell, just me.

# case 0128

32 years ago I was unconscious for a couple of days after being beaten. After getting out of the hospital I realized that, in addition to other problems, I could not smell or taste anything, not even a skunk. What I can perceive is sweet, sour, bitter, and salt (leading me to overuse salt), and spiciness.

# case 0129

One day, out of the blue, I started experiencing a horrid taste/smell while not eating/drinking anything. It only lasted for 20-30 minutes but I was quite thrown off because I never experienced anything like it before. A year ago the smell distortions (which at times smell like burning, garbage, bowel movements and many other noxious smells) had gotten so bad that I had the issue more often than not. It makes everything taste horrible and smell horrible. I have been able to deal with it but I have never been able to smell or eat chocolate and coffee while I have the smell/taste because they are just too bad to handle. Due to the intensity and length of time I finally decided to go see my doctor because it was beginning to weigh on me emotionally. Every time I would get it, I would drop into an annoyed/sad mood because I knew I wouldn't be able to enjoy anything I ate or drank, and everything would smell horrible.

# case 0130

I have undergone sinus surgery four years ago. My nasal obstruction was cleared but I started facing other problems like a gradual loss of my sense of smell that started last year. I have not lost the sense of smell completely, but it is dramatically reduced.

# case 0131

I lost my sense of smell after a head injury three months ago. Recently I have noticed on some occasions the smell of cigarette smoke but couldn't detect a source of the odor; no one in my house smokes. I hope that it will heal with time and will also try acupuncture. I feel very frustrated and depressed about this loss, I have always been someone who loves good food and the wonderful smells nature has to offer. I may have to live with this condition all my life but I truly hope that one day I will be able to smell our baby.

# case 0132

In the past year my sense of smell has greatly diminished. I began using a sinus flushing to see if this would help and now rubbing alcohol smells totally different (bad). Rubbing alcohol smells different but I notice no improvement in my overall ability to smell things.

# case 0133

Around the age of 18 I had head trauma after an accident. Around the age of 22 I realized that I had lost my sense of smell. I can't smell anything, and thus I am unable to identify the food that I eat. Once I was in a room with a very disagreeable smell and I was the only one not covering his nose with a handkerchief. I told my doctors about my smell loss, but they did not take the issue seriously. With this loss I always face many untold hardships in my married and routine social life.

# case 0134

I don't think I ever had a sense of smell. I have very few memories from childhood though, so I can't remember exactly when I noticed. I can sense certain smells if I stick my nose right up to the smell but distinguishing them from others is next to impossible. The fact that there are certain smells that I can get a sensation from is interesting to me. I do wonder sometimes if maybe I just had a weaker sense of smell when I was young but enjoyed the attention of not having this sense. I wonder if maybe I resigned myself to not having one...did not try and use it enough?

# case 0135

A year ago I realized I couldn't smell and hadn't been able to smell for about two months. I was diagnosed with rhinosinusitis and sinusitis. I was put on steroids and got my smell back. I thought I was cured, but after the steroids wore off, in three weeks it was all back in full force. I later got steroids on two other occasions and again they brought relief, but only temporally.

# case 0136

I used to work with cleaning chemicals including ammonia. Over time, I became very sensitive to cleaning products, petrolatum products, and products with fragrance. Now I avoid all fragrance in cleaning and laundry and personal hygiene products. Over time I have gradually lost most of my sense of smell. The last few years I have experienced a very annoying problem with my sense of smell. It is much distorted and once I get a certain smell in my nostrils, it won't leave for weeks. Coffee started smelling like motor oil. Cooking anything smelled like rancid fat. Everything smells like that particular smell and it's not a good smell. It is almost literally driving me crazy. I keep asking everyone if they can smell "that smell". No one can. It seems I'm locked in my own little world of foul smells. I am experiencing a smell right now that I can't identify. Every time the furnace comes on, I smell it. It permeates the house, but no one else can smell it. I smell it in the car. I smell it on other people. I don't know what it is, but it is making my life miserable. I wish I could change this perpetual odor in my nostrils to roses or something clean and pleasant. I'm at my wits end.

# case 0137

I always had a very acute sense of smell and never had sinus problems. Then I was caught in a dust storm and developed sinus congestion and used nasal sprays. I lost my sense of smell. After six years only small parts of it have come back. It has affected by enjoyment of life, tasting food, smelling flowers and even being excited by the smell of my husband. I can't smell spoiled food or leaking gas. It makes me very sad and I don't think others realize how it affects me.

# case 0138

When pregnant with twins, my nasal membranes went into overdrive producing vast amounts of mucus and swelling. The swelling and overproduction subsided once the pregnancy was complete; however, I have lost my sense of smell. Even more odd is that I now smell smoke periodically. When that occurs, it often lasts for a week or two and then dulls.

# case 0139

I came down with a severe case of the flu. I used several medications and lost my sense of smell completely. After about three days I regained a sense of smell but nothing smelled the way it should. I went to an ear nose throat doctor for help with my anosmia. The doctor's attitude was "it may come back and it may not". I can smell things but most everything smells the same. I have to ask my family what I am smelling when I smell something I can't identify. If I am putting gas in my vehicle and smell something I know that it is the gas but it does not smell like gas. I cannot tell if food or milk has gone bad because I smell nothing as far as food products go. I can taste things but nothing tastes the way that it should. I can tell if a food is sour, salty, or sweet. I am a firefighter and have always been able to use smell to identify hazards (natural gas, wood fire, etc.) but now everything I am around smells the same. There is no difference in the smell of urine, feces, burning wood, wet dog, gasoline, or even skunk.

# case 0140

I first noticed a change in my sense of smell when I was in my early teens. There were times when my sense of smell was very sharp and only I could smell certain things from a distance and other times when I could smell nothing. I never thought to get it checked out because, of course, everyone (myself included) thought that I just had a lingering cold. I could smell sometimes and was happy when I could. I tried many medicines for colds and allergies but nothing worked. I noticed that my smell finally went away completely about five years ago when I was cooking and left the room and returned to a grease fire in my kitchen. I couldn't smell the smoke and this is when I finally realized that there was something terribly wrong with me. About a year ago, I noticed that I could smell the soap while taking a hot shower but it only lasted about 30 minutes. I went to an ear nose throat doctor who told me that I had chronic sinusitis and gave me two weeks' worth or prednisolone. The very first day I took this steroid I could smell everything. This only lasted as long as I was taking it. As soon as I stopped taking it, I could no longer smell. The doctor would not give me any more steroids because he said it is very strong. I now take long hot showers, use a neti pot, and inhale the steam from a boiling pot of water. These remedies allow me to smell somewhat every few months. (They don't always work).

# case 0141

I fell down and hit my head in a parking lot. On the next day I realized that I can't smell or taste anything.

# case 0142

I constantly experience a burnt smell, like wet smoke or wet camp fire.

# case 0143

I cannot smell anything! I also cannot taste anything. My doctor says that I have had three minor strokes that have caused this. I have not tasted anything in three months.

# case 0144

I suffered a head trauma from a car accident thirty years ago. I needed craniotomy to repair a cerebrospinal fluid leak and as a consequence of the surgery I lost my sense of smell.

# case 0145

I don't have a sense of smell. I have congenital anosmia. It took me a while (several years) to even notice I was lacking the sense, since I have no frame of reference. I started asking my parents about it when I was pretty young (9 or 10) but they thought I was just trying to get attention. It wasn't until I was 15 that I got an MRI and found out that I completely lack the faculties to smell.

# case 0146

My sense of smell has changed. I still smell smells, but they are different. Everything smells the same, like the smell of smoke from fire. I have been to my doctor but no underlying cause was found.

# case 0147

I started to use prescription nose sprays to help with my sneezing caused by hay fever. During this time my sense of smell came and went. Then, ten years ago, I had sinus surgery. This gave me some relief with my smell improving a bit. The polyps returned and I now need another surgery. My sense of smell is now completely gone. It's made me quite sad at times as I can't get excited about a new restaurant, the smell of summer, or any other smell that brings out an emotive response.

# case 0148

Last year I fell on the back of my head and had a concussion. As a result I lost my sense of taste and smell. I still have no sense of smell and taste and experience strong phantom odors most of the time. I have discovered that smoking relieves the problem. Have tried taking nicotine pills but don't get the same result as smoking.

# case 0149

I smell a smoky/car exhaust smell almost all the time. I know it is in my sensory experience only, not actually present. It is as if the smell is right inside my own nose. I just now looked online in case anyone else had experienced the same phenomenon, and sure enough, there are lots of us! I found many questions as to the cause of this, but no definitive answers. The smell sometimes goes away, but usually it is present.

# case 0150

For the past two weeks I have had a phantom smell of cigarettes. It started on a day that I was around a lot of people smoking. I have had this smell ever since. I have washed everything and tried purging my sinuses with saline but nothing has changed the smell. I notice it more sometimes and on other occasions the smell is almost gone. Most of the time it is like I smell a stale ashtray.

# case 0151

I have never been able to smell anything. I appear to have a normal sense of taste to the surprise of many.

# case 0152

I lost my sense of smell about seven years ago. It started with phantom smells, a sweet odor that no one else smelled. Then I completely lost my sense of smell. Nothing got through anymore. Periodically it comes back for a few weeks, very strong, but then slowly drifts away. And often I can get an initial whiff of an odor. But as soon as I try to smell it again, it's gone.

# case 0153

At the age of 50 I started to gradually lose my sense of smell. It is now gone completely. My sense of taste is also gone, although I can identify sweet and salty tastes. I have nasal polyps but was told that they are too small to warrant surgery but large enough to disrupt sense of taste/smell. My condition is beginning to affect me psychologically. I can't taste food or drink.

# case 0154

A while ago I started noticing that I keep experiencing a burning smell even when there is nothing burning. I feel like the air has that burning smell. I recently stayed at a hotel and had that burning smell in the hotel room. When I opened the windows just a bit and felt the breeze come in, I did not feel it. When I am at home, I don't always have that burning smell. I sometimes go out to the yard at night and take a deep breath and don't smell it, just a fresh sense of air. So, I don't know if the burning smell is a hallucination or if it has something to do with the places I visit.

# case 0155

Apparently, I can't smell skunk. When there's a skunk, I don't smell the awful smell that others describe. Instead I usually experience some other, more pleasant smells when encountering a skunk. It's usually smells associated with a cook-out (charcoal, hamburgers, hotdogs), but it also has been things like pretzels, broccoli, or even string cheese!

# case 0156

I discovered at age 18 that I have no sense of smell. I was in fact born with no smell and have no recollection of smell. I have limited taste and rely on texture and visual presentation to entice me to eat. I do love to cook and create in the kitchen and there are no complaints when I entertain, thankfully...!

# case 0157

When I was ten I noticed that I can't smell anything whether it's good or bad. Instead of smelling I would be nauseated and wanted to vomit if the smell was bad. If the smell was good I would crave the smelling thing although I did not "see" it. Other children would run away from a dead dog and I would go straight to it and even see it but smell nothing. I'm still like that but now I can smell sometimes but it will be once in six months and then nothing.

# case 0158

I cleaned my house with a combination of bleach, vinegar, and bicarbonate soda. After the third cleaning session I found that my sense of smell had declined by perhaps 80%. The difference is extremely noticeable because my olfactory senses were so pronounced that sometimes their sensitivity would cause discomfort. For instance, if someone walked outside of the house with a cigarette in their hand, I would notice the unpleasant smell even if they were on the other side of the road and I was in the back bedroom. Friends would ask me to detect the specific spices and seasonings they'd used in foods and I would pick out elements such as certain types of brands of sauces and types of soy sauces. Now, I can barely tell what I'm eating. The other day I was about to call back a waitress and complain that she'd served me the fish dish, when I came across a tell-tale bone that revealed she had actually served me the chicken dish I had ordered. It's driving me crazy!

# case 0159

Four years ago I had a bad cold and as a consequence my sense of smell was reduced by about 80% and never recovered. The doctor said he didn't know when or if the sense would be restored. I was left with the impression that it was something I just had to accept and deal with. Currently my main concern is for my children — I am unable to tell if lunch meat, milk or other perishables are spoiled, and I wouldn't be able to smell a gas leak or other olfactory hazard if I had one. I want to do what is necessary to restore my sense of smell.

# case 0160

When I was 20 I noticed that my sense of smell is bad in the winter but normal during the summer. After a couple of years I realized that I had some problem with my sense of smell. Doctors were no help. Five years later my sense of smell was gone in summers as well. Now I am not able to smell anything. This doesn't mean that I can't smell at all. What I have observed is that after a workout or a sudden change in temperature my nostrils are back to normal. Within a minute or so it is gone again. I desperately want it fixed. I want to smell flowers, I want to smell my soap, deodorant, aftershave and I want to taste the food I eat. I want to be able to distinguish coffee from tea.

# case 0161

I fell and hit the ground with the back of my head. I was severely concussed for days and only noticed after a week when I was spraying on deodorant that I couldn't smell anything. Due to the trauma the nerves connecting my nose to my brain were severed and I no longer have a sense of smell, although by some small consolation my sense of taste has not been affected.

# case 0162

I have a very sharp, keen sense of smell. I have used a neti pot occasionally in the past but recently began using it twice daily. The last week I've been experiencing phantom smells upon waking in the morning. I have smelled toast, breakfast sausage and most recently a strong maple syrup scent. When I get into the kitchen, no one is cooking.

# case 0163

I lost my sense of smell as the result of a common cold. The smell loss occurred over a period of 3-4 months and is now complete. I have been unable to smell for two years and it is incredibly upsetting.

# case 0164

I had a bad cold which was followed by a much diminished sense of smell and taste. This went on for a month after my cold was gone but then I started to notice a mild weird taste in coffee. Within a few weeks the weird taste got stronger and started to affect more foods, including chocolate, yogurt, cottage cheese, fried foods, onions, green peppers, pancake syrup, beer, wine, grape fruit juice, and most snack foods such as chips (potato, corn, tortilla), crackers, pretzels, plus cakes and candies of all kinds. I could not even eat my birthday cake. Fruits of all kinds are at least a little bit weird with the taste becoming worse when the fruits become overripe. Water, skim milk, eggs, honey, and some cheeses taste normal. Most types of beans have a generally normal taste. It is difficult to describe the type of smell/taste other than it is unpleasant and not like anything familiar. At first I described it as somewhat of a moldy/mildewy smell and taste though I don't know what mold smells or tastes like so that probably is not quite accurate. Most fragrances, aromas, and odors all have the same weird smell to me. Examples of things that smell very bad to me are coffee, chocolate, smoke from a wood fire or from cigarettes, most perfumes and room fresheners, many lotions, hand sanitizer and soaps including dish soap. The distorted smell is so disappointing. It used to be that whenever my husband would grind coffee beans or when the coffee is brewing, I would inhale to take in the wonderful aroma. Now, I leave the room to avoid the unpleasant smell. When I take the bed sheets off the clothesline they no longer have that wonderful fresh scent. As I cooked up apples to make applesauce, the formerly delicious scent of cooking apples spiced with cinnamon and cloves now has that same weird smell like so many other things. I went to see an ear nose throat doctor who found no abnormalities and referred me to a neurologist. The neurologist is confident that this is due to damage from the cold virus. He called it dysosmia and told me there is really nothing he or I can do about it. It may heal up completely, partially, or not at all and if it does it will likely take a long time to heal. He assured me it will not get worse.

# case 0165

My sense of smell diminished rapidly at age 48 or so. I noticed an increase in what appeared to be cold sores in my right nasal passages. After several bouts back to back the outbreaks stopped. During this time I noticed smells changing. Several odors were noticeably distorted before becoming almost undetectable. I have had a medical exam. I was told my condition is age related with no treatment available. To me, this is an unacceptable diagnosis. I believe this condition could be slowed or possibly reversed with proper nutrition.

# case 0166

At the age of 35 I started to realize that I am not able to smell. Several physicians were not able to find a physical cause despite detailed investigation. They could not suggest a course of treatment. I am living with this.

# case 0167

When I was about 30 I noticed that smells seem to change and then change back. Grapefruit juice would smell delicious, the best thing in the world; then a few weeks later it would be nauseating. The same thing would happen to the bouquet of white wines. My then husband actually wrote down these changes and noticed that they happened on the same cycle as my menstruation. This phenomenon seemed to fade by my late 30s. In my late 30s I was pregnant and smells took on an entire new level of intensity. Much of the pregnancy I could not bear to eat meat because so much meat smelled off, then I would have a day clear of rottenness and could eat meat. Other smells either faded or took on great pungency; I particularly recall pickles and cucumbers smelling totally wonderful.

# case 0168

About ten months ago, after a regular cold, I noticed that I could no longer taste food or smell anything!

# case 0169

I suffered a subarachnoid hemorrhage ten years ago. Since then I noticed a progressive disturbance in my sense of smell and taste. In more recent years I have been suffering from olfactory hallucinations. These seem to occur mainly during the night time and are always unpleasant smells.

# case 0170

As a teenager, I frequently suffered from a running nose and sneezing and coughing. I also had allergies. My sense of smell came and went over the years. Nasal polyp removal surgery brought temporary relief (for a few days). Currently I have no sense of smell or taste.

# case 0171

Twenty years ago, when I was thirteen, my sense of smell started disappearing and reappearing. The occasions on which I can smell became rarer over the years and now I can only smell once a year.

# case 0172

I started to notice a smoky odor a number of years ago. It's not a cigarette smell but rather kind of a burnt wood smell. It comes and goes and it stays around for quite some time. I am currently experiencing a really strong bout of it. Added to the smoky smell is what I can only describe as a really unpleasant "stale old lady" perfume odor. Plus, this time I seem to have a burning sensation in my eyes at the same time. I believe what I have is "phantosmia". I mentioned it to my doctor a few years ago during a physical and he looked at me as if I were crazy. I haven't really said too much to people about it because how strange it seems. If I take several deep breaths through my mouth it will subside for a short time but returns quickly. I seem to be able to smell other odors okay along with the phantom smells.

# case 0173

Perhaps I have had this condition all my life, but only started noticing it. It seems that at times my smell has a "memory" and if I am exposed to certain smells, they linger with me for the rest of the day. Once I have gone to sleep they are gone by the following morning.

# case 0174

I didn't notice that I lost my sense of smell until one day when I went into a perfume shop to buy some and noticed that even though I didn't have a blocked nose I couldn't smell any of the perfumes! That's when I realized that I couldn't remember the last time I smelled something. I went to a homeopathic doctor and he diagnosed me with anosmia and gave me medicines. After seven months I regained my sense of smell but I have lost it again.

# case 0175

Since last year I have chronic sinusitis. My sense of smell has gradually deteriorated, and while I can still discern smells from one another if examined at extremely close distances (read: sniffing distance) my ability to smell has certainly diminished from what it once was. My olfactory memory has also been profoundly affected by this loss of scent, and I can no longer recall the smells surrounding intimate and sentimental moments of my past. Frankly, the loss of my sense of smell and the effect it has had on my perception of things has dampened the colors of the world for me.

# case 0176

I have never, for as long as I can remember, been able to smell. Not even the strongest of scents. I can still taste fine, but I love strong tasting foods for example curry, so I may not be able to taste as well as everyone else.

# case 0177

About 10 years ago I started to notice that when the dogs farted it did not have an unpleasant smell, rather a vaguely metallic odor, a bit like a lit match. Over the last few years it seems that my sense of smell has deteriorated considerably. Now I can smell very little; escaping gas, grandchild's dirty diapers, and other normally unpleasant smells pass me by. It has not reduced my appreciation of food, though I can't smell cooking unless something is burning.

# case 0178

For the past few months I have started to smell something burning, sort of like a cigarette smell, when there is nothing burning at all. I wake up at night thinking there is a fire in the house due to the smell but it is all in my head. This is getting worse all the time. I have more phantom smells at night but it also happens several times a day and will last for 5 to 10 minutes at a time. It is always the same smell. The problem seems to be getting worse.

# case 0179

I am really not sure exactly when I lost my sense of smell. I did however notice that when anyone said "that smells great" that I couldn't smell it. I cannot smell coffee brewing, bacon frying, chicken soup, or anything else. I am worried that I will not be able to smell a gas leak, as I live on my own. I have had sinus problems for many years, and have a nasal drip every morning, and sometimes my nose gets stuffed up. I used nasal sprays for years. I long to smell what others can smell. Because I have no sense of smell I have no appetite and all food tastes the same, unless it has a spicy taste, but I cannot distinguish what spice was used.

# case 0180

For the past week, I've been smelling the odor of gasoline fumes wherever I go. I first began to smell them in bed when I was sitting up with my laptop working on a project. Every five minutes or so I would notice the odor, and then as I would inhale deeper to identify the odor it would go away. This went on all day. I thought perhaps it was coming through my window from the street below. But then the following day, I was at a friend's house in a city twenty miles away, and I smelled it there too. Since then I continue to smell these fumes that don't exist, and it's beginning to make me feel really nauseous. I've changed clothes, showered, checked my breath, and still these distinct odors are present. I've asked others around me if they smell gasoline too, and they do not. I know there's something going on with my body that is causing my olfactory sensors to malfunction. I just don't know the cause.

# case 0181

I had a head injury after falling last year. I regained all my senses except for smell.

# case 0182

About a year ago I had the flu with fever, congestion, fatigue, body aches, and coughing. I used a neti pot with sea salt but it felt like burning in my nose which was different from my past experience using a neti pot. After recovering from the flu I lost my ability to smell most odors and I sometimes smell an unpleasant odor that I don't recognize. I've also lost some of my taste.

# case 0183

I lost a considerable amount of my sense of smell over a period of ten years or so. I think some, but not all of it is due to allergies and the use of nasal sprays. I am able to smell strong solvents, smoke, perfumes, bleaches and chemicals but often cannot smell everyday smells like body odor, flowers, and food smells. I also can't smell offensive smells like rotting substances or fecal matter. I often have to put my nose very close to things to get any smell at all. I've found over time that I make allowances for this missing ability in many everyday circumstances.

# case 0184

A couple of months ago I started perceiving a smell of smoke most of the time. It went away, but today I woke up in the morning experiencing the smell of vitamins.

# case 0185

I keep noticing a smell of burning rubber. Nobody else can smell it.

# case 0186

My nose has been congested for years now and I can only smell and taste something when the odors/flavors are very intense. I had surgery to correct my nasal septum but it did not affect my sense of smell. Doctors now tell me that my nose is simply too "narrow" and you cannot do anything about it. Strangely enough when I'm sleeping I seem to be able to breathe through my nose without problems. Sometimes I can smell something suddenly, like trees in flower, and in these moments I'm very happy. Another problem is that I'm often afraid that my body is smelling badly and that I disgust other people. When I am invited to eat and am asked if I like the food I have to lie because I do not know how it tastes. For me the consistency of food is much more important.

# case 0187

I suffered head trauma from a fall. After returning from the hospital all I could smell was a constant strong chemical odor similar to that of a commercial copier machine, and I could not taste anything! Eventually, after about a month, my sense of taste started to come back slowly. The first thing I could taste was ketchup and tomato sauce. Just recently, six years later, I smelled the smell of cow manure. Is there hope that I can regain my sense of smell?

# case 0188

It may have been longer than four years ago that I suddenly realized I could not smell anything. I have been to the ear nose throat doctor and they say there's nothing wrong. The doctor asked if I had any head trauma — which I really don't remember — I had fallen once and hit my face. I have done nothing more about it. And as much as I miss the nice smells I don't miss the bad odors — but I worry that I wouldn't smell smoke or gas leak.

# case 0189

I cannot recall ever having a sense of smell. But I recall the smell of pancakes and spaghetti, so I must have a sense of smell at some point. My taste buds work like normal, although I am very picky in what I eat.

# case 0190

About a year ago I started realizing that I can go past an area of terribly bad odor without feeling anything. Once I entered a room in which others covered their noses and gasping for fresh air, but I went inside as if nothing has happened. I do suffer from allergic rhinitis but it is well under control with medications.

# case 0191

I fell and injured my head. Sometime later I realized that I lost my sense of smell. I went to an ear nose throat doctor who told me that he couldn't do anything for me, and I should put more smoke alarms in my house. I was very disappointed with what he told me. I realized later that my sense of taste had diminished, too. I just went on the internet to see if there was any cure for the loss of smell. I was again disappointed to find out that there was very little study by the medical profession on the loss of the sense of smell. It is time something is done about it.

# case 0192

I lost my sense of smell after injuring my head in an accident. Over the years since, it has improved to the effect that I can sense certain smells and recognize them for what they are. There are however a lot of smells that I still can't detect, especially sweet smells like perfume as well as some unpleasant smells. It seems to have stabilized and isn't really changing any more. The strange thing is that I used to have a very acute sense of smell and was very focused on it before the accident.

# case 0193

I was repeatedly hit in the head. I woke up in the hospital a few hours later and haven't smelled a thing since.

# case 0194

For as long as I remember I have smelled smoke at times when no one else does. I got used to it. About a week ago something different happened, though. My husband was at work and I was home alone when suddenly I started to smell something "odd". I absolutely could not identify the smell. I was afraid something was burning like wiring in the walls or something. It was even getting into the back of my throat so I could taste it. When my husband got home he smelled nothing! To me it is so strong that at times it makes me feel sick. The experience has been coming and going. Right now it is incredibly strong. It does not smell like food but it also doesn't smell smoky. It is driving me nuts! That is concerning me too...am I going nuts?

# case 0195

When I was about nine I noticed I couldn't smell things as much as I used to. It was like this (decreased smell) until about three years ago, when I noticed I could only smell manure and skunk (along with some random flowers). My sense of taste has not — as far as I know — decreased whatsoever. Now I enjoy the smell of manure and skunk.

# case 0196

I lost my sense of smell gradually over 18-24 months. I did not notice it at first because it was a subtle process but over time when I did notice my loss I also realized that it impacted my sense of taste. I notified my doctor but he didn't seem to be alarmed about it — he told me that a lot of people lose their sense of smell; sometimes it comes back and sometimes it doesn't. I was concerned over his response since this is a big change for me — not being able to smell. I cannot smell anything (for about 3 years now).

# case 0197

Since I had bariatric surgery, I occasionally smell a strong odor of ammonia. People around me do not sense the smell.

# case 0198

My sense of smell just disappeared. I use perfumes so as to smell nicely but I, myself, don't smell it.

# case 0199

About a year ago I had a severe cold and sinus infection. As I recovered I noticed that everything smelled of burnt electrical wire, especially indoors. I couldn't smell perfume or aftershave as it gave off an intense odor similar to burning meat, wood, and plastic. About six months ago, I started to smell and taste individual odors again. However, the recovery was only temporary. It's only now that I am seeking medical help as I find I am becoming depressed by everything smelling the same and tasting the same. I used to be a chef and now I can't taste what I create. I have also noticed that my libido is not what it was. I'm wondering if this has to do with my condition, as they say that pheromones play a very important role in this aspect of our lives! All in all, it's a very peculiar feeling to lose one of the bodies major senses. And it's also becoming increasingly tedious.

# case 0200

When I was six years old I noticed people mentioning how good or bad something smells. I had no idea what they were talking about. I could recognize the skin of oranges, spirit, smoke or anything that stinks only because it suffocates me.

# case 0201

For the last two months I smell a smell like burnt plastic. Sometimes it's faint and sometimes it's very strong. No one else can smell it.

# case 0202

After a bicycle accident with head trauma I lost my sense of smell.

# case 0203

A couple of years ago I began getting a very bad constant smell of exhaust fumes. It bothered my eyes, my nose and my throat. I went into an emergency room at one point because it was so bad I couldn't concentrate. I get headaches and nausea from it. The emergency room doctor could not find anything wrong and pretty much dismissed me as someone who should not have wasted his time. It got so bad that when I tried to sleep it would keep me awake. One episode of this lasted for a full month. It doesn't stop gradually or begin gradually, it's sudden and totally unexpected. The only thing I can make any sort of connection to in regards to the current spell is that I have just come out of a very bad cold and my sinuses were in bad shape with plugging and drainage. The cold was two weeks ago, the exhaust smell began two days ago. I do get migraines and suffered one four days ago that made my stomach upset and nauseated for the two following days... when that stopped the exhaust smell began. I have not changed eating habits, and pretty much everything I do is about normal. I am a smoker, but the smell that I experience is totally different than cigarette smoke. Anyone who has been stuck in a garage or enclosed area with a car running or a bad muffler would know exactly what this is.

# case 0204

I lost my sense of smell. I had a CT scan that showed polyps and fungus in my sinuses. Homeopathic medicine over one year cured me. My sense of smell increased after using the homeopathic medicine, so now I have an overly acute sense of smell and this causes problems with my family members.

# case 0205

I had breast cancer 18 years ago. I went through all the required treatments and have been cancer free since. After my treatment, I noticed that my smell for all kind of odors was going in and out. I have been living with this the whole time. I have the taste of sweet, sour, bitter, salt, but no flavor. I do have some sinus problems but have not been bothered with it for some time.

# case 0206

Seven years ago I had a cold with a running nose. After recovery I had lost my sense of smell. I regained it after one year to some extend but I am not sure the smell which I perceive is the smell that is out there. My confidence in my regained sense of smell is low and I feel that my abilities gradually increase and decrease now and then. Today I am suffering again from a cold and I lost my sense of smell again.

# case 0207

I have not been able to smell for a while now. It is really bad because I can't smell good things like candles, flowers, etc. I went to a doctor who said he didn't know what could have caused it.

# case 0208

I first noticed that I was getting phantom smells about eleven years ago. I might be sitting in my office and smell paint fumes when no one else could. Over time I noticed that I couldn't detect the more delicate smells like flowers and only taste stronger flavors like curries and heavily flavored spicy foods. Now I taste very little, more textures and salty things. I occasionally get a sense of smell in the left nostril but can't identify what it is, just if it's pleasant or not. I have no idea what started it.

# case 0209

A few months ago, after a severe two week headache, I lost my sense of smell. The doctors could not find any reason but I can't smell anything anymore. I sometimes feel like I smell some of the different drugs I am taking to heal my headache. Right now I am not using any drugs but I feel uncomfortable to be in this situation. My sense of smell was very strong before this incident.

# case 0210

I had a very bad cold and used some outdated nasal inhaler. The cold went away after about ten days but my sense of smell did not return after the cold. About six months after this cold I started smelling a little better but I noticed some organic odors smelled really bad, like garbage. I continue to have this smell disorder. Many smells of food seem like they are rotten or a mild garbage smell. Some odors seem like something I've never sensed before. Coffee and orange Juice for example smell pretty bad to me. I used to love these smells. Orange peel smells awful. I don't know if my sense of smell will recover or if I will have this problem for the rest of my life.

# case 0211

My loss of smell has been gradual. I had to guess when I first noticed it. It is almost completely gone now. I sought out medical treatment but found no relief there. The only thing that I think I can still smell is ammonia. I worry that I might not notice a fire, or a gas leak, or any of the other things that might happen due to having no sense of smell.

# case 0212

I noticed that after staying in a house where there was considerable mildew I seemed to have a lessened sense of smell. This was at the age of 67. When I hold a flower I sense that something is in the air, but I really can't call it a smell or odor. It's like the form of a smell is there, but there really isn't a smell.
Occasionally if I pass a really strong source of smell such as a petroleum refinery I get a burst of smell, but it doesn't last very long. I went to see an otolaryngologist. He said that my sinuses were blocking smells from getting to my olfactory nerves.

# case 0213

Four years ago, during what I thought was a sinus infection but has later been found to be a migraine, I could smell a peculiar smell of burnt rubber. The burnt rubber smell did continue and waxed and waned over the months. If I breathe through my mouth, it is not noticeable. The smell does not correlate with migraine attacks. It is just there off and on for no real reason.

# case 0214

I was born without the ability to smell. This was confirmed by an ear nose throat doctor after getting an MRI.

# case 0215

One day about 10 years ago I realized that I could not smell anything. This happened after open heart surgery. What does happen is that I can detect paint fumes from rooms a week to two weeks after the room or item has been painted. My taste buds are challenged with sour foods. But most of my smells around food are memories. I have electric appliances as opposed to gas for fear of not being able to smell a fire.

# case 0216

As a child I had a decreased sense of smell and problems with overgrown adenoids so I am a mouth breather. Increasingly I have troubles because I hallucinate the odor of cigarette smoke and now I seem to smell foul odors in the house that no one else notices and I'm gagging my family with air deodorizers. I have chronic sinusitis and many times can't detect odors that would be helpful (like when the leftovers in the fridge have gone bad).

# case 0217

I had spinal meningitis twice. I developed a spinal fluid leak and had a craniotomy to fix the leak. I haven't been able to smell since.

# case 0218

I notice that I lost my sense of smell about a year ago. Now I can't smell anything. I put on tons of perfume. Even when I change diapers, I can't smell anything.

# case 0219

I am experiencing a loss of smell. I can smell only strong odors form a small distance. I cannot smell fragrances at all , except a few times when I literally have to hold it against my nose. Along with this loss, I am also losing my sense of taste. Subtle flavors are out for me.

# case 0220

I lost my sense of smell five years ago after a head injury. I could not smell or taste after my accident. It got a little better with time. Sometimes I have a memory of a smell and think I really smell again only to be disappointed when the feeling passes.

# case 0221

After a mild upper respiratory infection, I now suffer from hyposmia and dysosmia. I can smell some things and not others i.e. I can smell some essential oils however they are not as strong or as sharp as I remember them to be. I have also come to notice that I have trouble smelling things with strong odors like fish sauce, dog muck, curry... On the other hand I sometimes can smell the delicate smell of a rose. I also receive the same strange chemical (burnt citrus) smell, particularly when moving into a new area i.e. going outside from inside. This smell slowly subsides; however, a new odor in this vicinity can easily bring this smell back again. I also often experience phantom smells of cigarette smoke. My sense of taste has been somewhat effected. This condition has made me quickly fall into a depression and has altered my life completely.

# case 0222

About eight weeks ago, I lost my sense of smell and sense of taste. I went to an ear nose throat doctor and was told I have a very tiny cyst in my sinus. He told me that if I can breathe through my nose, I am not a candidate for surgery. Also, the doctor told me that my sense of smell could come back in three months, or six months, and that there is no pill that can make it come back. Finally, the doctor also told me that there is no medical explanation for my loss of smell. I wish I knew how to get my sense of smell and taste back.

# case 0223

I cannot pinpoint a date or event when I realized I lost my sense of smell; I would say it was approximately two years ago. I recall walking outside with my sister and she commented on a strong foul smell that I could not smell at all. Now, I only smell something occasionally if I put it to my nose and take a long sniff. These smells pass quickly and I can’t really determine what it is I smelled. Also, sometimes I can smell smoke/burnt smells but they pass fast. I do recall, at bedtime, phantom smells of smoke. I would say that happened fairly frequently but maybe it only lasted a month or two. I haven’t had the phantom smells in the last eight months. I still have a sense of taste. However I think it is starting to diminish. I have not lost my appetite but I am never satisfied either.

# case 0224

Four years ago I had a bad cold and used nasal spray. Afterwards, my sense of smell did not fully return. Eventually, I visited an ear nose throat doctor. Tests revealed that I have some limited sense of smell (I can detect some perfumes, incense, floral scents) but I would guess it has diminished by 75% along with the sense of taste.

# case 0225

I went on a cruise. For about three weeks afterwards I could experience a strange smell like that of the room I stayed in on the ship. The smell went away and so did most others. Now I can only smell certain things and sometimes I suddenly smell a strange sweet smell that no one else can. I don't know what happened but I am finding the ordeal disturbing.

# case 0226

One day, very suddenly (after a flight back from vacation) coffee smelled nasty. Other food did not smell nice either and flowers had no smell at all. The next day I realized that I had a problem and I began to test myself. I could not smell bleach at all. I could only smell a chalkiness and background unpleasantness of any of my perfumes. Chicken and fish taste acceptable. Strong flavors taste "nasty". Frying onions smell revolting. I am normally a good and enthusiastic cook — my sense of taste and smell has always been very acute. I have always cooked, not by measured recipe but by taste. This I am totally unable to do now. The subtle flavors of herbs just are not there or are different. I can taste sweet, and salt but sour isn't the same. I can feel the heat in peppers. Wines taste chemically. I can tell little difference between Brandy, Whiskey or Rum, while Gin and Vodka taste of nothing. A dry Martini tastes okay. Sometimes I think I get the hint of a smell but it does not last. Smells seem to alter too. However, the constant is that nothing smells nice. Life is miserable. My doctor seems to think that I have to learn to live with the problem. He says it is most likely "old age". This I could understand if it had happened slowly over a period of time, but it happened over a period of hours.

# case 0227

I have a history of sinusitis and allergy for decades. I had several surgeries and used a variety of medications for this condition. Now I have lost my sense of taste and smell.

# case 0228

I fell and hit my head. After the accident I had a hard time breathing and cold air made me cough. It finally got better although now I couldn't smell a thing? I also could barely taste. Now, a year later, I still have migraines and I definitely can't smell a thing...

# case 0229

I have bipolar disorder. Sometimes I have olfactory disturbances before an episode or even in a much milder phase of the disease. I use this heightened state of awareness of smell as an indicator for medication to avoid psychotic episodes. The smell of burnt almonds especially bothers me. I am not aware of where it originates in the brain but it is a definite indicator to me to be vigilant.

# case 0230

A few years ago I began noticing episodes of smelling something that I can only describe as something dead or rotting. In the beginning this was very rare but it has become more frequent in the past nine months. It is sometimes accompanied by a sense of nasal stuffiness or fullness, but I do not have cold symptoms, headache or facial pain or pressure. Whenever I ask somebody if they can smell the odor, too, the answer I get is "no".

# case 0231

I have congenital anosmic and only realized it when I was 16!

# case 0232

I had a cold and used nose spray. Even though my cold is over and it's been three weeks I still cannot smell or taste. There are moments when I think I've smelled or tasted something but it is short lived. I love experiencing the flavors in foods so this is very disturbing for my quality of life.

# case 0233

I experience a smell of cigarette smoke (I don't smoke nor does anyone in my family or friends). This odor began about five years ago and might last for weeks. I can smell other things but the smell of cigarettes is always with me. It might leave for six months but always comes back. It drives me crazy always having this scent.

# case 0234

Recently I noticed certain odors seem to get "trapped" in my sense of smell, and I can't seem to stop smelling the odor of either chemical or certain food odors. The odors make me feel sick and I smell them even after I have gotten away from the smells... I struggle with depression and now this "smell disorder" is really affecting the quality of my life on top of everything else. I thought at first it was all in my head but now I am not so sure anymore.

# case 0235

About two months ago, I started noticing that I was continually sensing a smell that seemed to be part cooking oil, part garlic, part fish oil — all combining to make a somewhat nauseating cheap restaurant smell. Everything I eat takes the same bad smell. Whenever I enter a restaurant, I'm overcome by the same smell, but at a higher intensity. Nothing I eat tastes the way it used to taste (everything I eat tastes bad with the new taste being the same as the new smell). I cannot think of anything that could have triggered the problem.

# case 0236

I had two severe colds within short intervals. By the end of the second cold I had lost my sense of smell.

# case 0237

I got punched in the head. The result was that I lost my sense of smell and taste. I had a brain scan and it showed nothing wrong with my brain. It was the punches to my head that caused me to lose my senses. I have been told it is permanent because my nerve-endings are damaged.

# case 0238

About a month ago I had a bad case of sinus drainage and throat and lung congestion after which I found that I had lost my sense of smell and taste. I had my head x-rayed and my doctor said that nothing showed up. He did not seem concerned. I however am concerned and feel that there should be something that I can do to improve my situation, but do not know where to turn.

# case 0239

I completely lost my sense of taste and smell. My sense of smell has slowly returned over the year. At first I could smell nothing, and then I could smell things that were brought close to my nose. After a year I am now beginning to smell environmental smells like popcorn at a movie theater.

# case 0240

About a year ago I noticed that I was not able to smell my perfumes and then my son brought a dog home and I was unable to smell when he had an accident or passed gas. I can't smell the odor of my foods when I'm cooking. When I go into a restaurant I can't smell the smell of foods or the fragrances of the room. If I put something close to my nose I can smell a little but it doesn't last.

# case 0241

I suffer from allergic rhinitis. I traced the allergy to tea and coffee and got desensitization treatment that worked. I can't smell very much and most of the food I eat now tastes like wet cardboard.

# case 0242

I lost my sense of smell step by step when I was 18 years old. At the age of 23, I had lost it completely. One of my brothers also has no sense of smell.

# case 0243

I fell and had to be treated in the hospital for almost a month. I realized, in hindsight, that I never could smell the hospital food, the flowers I received or even how I must have smelled due to not being able to take a bath for a couple of weeks. Not until I got home did I realize that my nose was burning very badly and everything I inhaled smelled like something burning, strong smoke, or electrical fire...I didn't even have to inhale. The smell is always there. I still go to smell things forgetting that I cannot. My taste was not affected at all. The rancid smell is not so strong as of recent but I still can't tell bleach from water. I notice when I am fatigued or stressed, I will get a series of a few hour of smelling that horrible smell again. It doesn't change. It is rather depressing because you cannot smell the grass, coffee, or the people you care about. Christmas is here and it will be the first time in my whole life I won't smell people cooking or the tree. This really is no fun. I have no idea if my sense of smell will ever come back, but from what I have read, it is highly unlikely.

# case 0244

About two months ago I did catch a cold and used nasal spray. After a while I started to notice that I can't smell well and taste very little. Now, two months later, I still don't have a sense of smell and taste and my nose is congested constantly.

# case 0245

About ten days ago I had a mild cold. I didn't have any nasal congestion, but I had developed a slightly sore throat and a cough and used nasal spray. This is when I lost my sense of smell. At this point I'm still hoping that I will get it back. The only things I've been able to smell faintly are lavender soap and fresh cat poop.

# case 0246

24 years ago I lost my sense of smell after head trauma as a result of a car accident. I have phantom smells.

# case 0247

After a viral illness I have lost my sense of smell. I cannot smell even strong smells such as garlic, lemon etc.

# case 0248

Two years ago I had the flu. The symptoms came and went four times over several weeks. I used an unapproved homeopathic product to treat it. After I recovered I had a much suppressed smell function. The taste function has not changed since. I have the sense that my smell is now "black & white" and no longer "in color". Anything strong smelling (skunk, feces, etc.) is just a stronger scent of the same smell... coffee smells slightly like coffee but also slightly like skunk/feces. Weak odors (flowers, perfume, etc.) have no smell to me. Rubbing alcohol smells like it used to.

# case 0249

Two months ago I had a cold — not a bad one, but I noticed that my sense of smell did not return, even after the congestion went away. Then a few weeks later I had another bout with viral meningitis. I now smell a slight pungent smell and this is for everything ranging from cookies baking to my cologne. I also have a bad taste in my mouth all the time, even right after brushing my teeth.

# case 0250

I am anosmic since birth. Until now I never experienced any odor, not even the smell of a strong perfume or a fart.

# case 0251

All of a sudden I can no longer smell aromas, perfume, food, etc. It's really puzzling to me. I've had a cold some time ago.

# case 0252:

After a fall and concussion I totally lost my sense of smell. Today I cannot smell anything at all.

# case 0253:

I never have had a sense of smell. I used to pretend in class at school when people farted that I could smell it, too. It was only much later that I actually realized I was missing something and started saying I couldn't actually smell anything.

# case 0254

I came down with a bad cold that seemed to work its way into the side of my face near the ear. Today I noticed that when I used vinegar to disinfect the counter I could not smell it.

# case 0255

I had a bicycle accident and two months later realized that I had a taste and smell disorder.

# case 0256

Recently I have been smelling chocolate. I love chocolate, so it's not an offensive odor. It happens anywhere, at any time...several times a day. This has only gone on for a week.

# case 0257

Earlier this year I felt that something about my smell and taste has changed. It just happened without any previous signs. Currently, my sense of smell and taste is very weak in the morning, but increases somewhat in the afternoon.

# case 0258

Last year I had acute nausea with extreme vomiting for 24 hours including a short episode of lost consciousness. One week after this episode I experienced the sudden loss of my sense of smell. I can still not smell anything. Since then I had two episodes during which I suddenly could smell something for a very short time (seconds) and then the sense of smell was gone again.

# case 0259

In my late 30s I had episodes where a single dominating smell would overwhelm me for days. No one else could smell it. Sometimes it was indistinct, even though powerful, and smelled like nothing in particular. Once, it smelled like detergent. When I was 40 I noticed that I really couldn't smell anymore. I had always had a very acute sense of smell and so I now miss it very much. I can, sometimes, pick up vague traces of very strong smells... like curry when it's cooking. I can't smell a skunk or a gas leak.

# case 0260

I was exposed to strong spices in my workplace for two years. Afterwards I had lost all sense of smell and to this day I cannot smell a thing. I did see a doctor about the condition when I started smelling odors that no one else could smell (because they did not exist). The doctor could not find a physical cause and the treatment with nasal sprays didn't work. I cannot even smell perfume if it is sprayed directly in front of me. I cannot smell skunks or liver cooking or coffee but I still once in a while experience a phantom smell and it is usually very unpleasant to the point that it makes me sick to my stomach.... I would so love to smell a rose again.

# case 0261

I noticed an awful gas-like smell last year. It comes and goes. I continue to have the smell. Right now, it has been with me for three full days.

# case 0262

I always had nose blockage and smell problems. My doctor discounted my smell problem with a "you need to just live with it"- approach. Since then, I had a septoplasty operation on my nose and this has cleared my nose, however, my sense of smell did not return and there seems to be a general medical approach in the UK of "tough luck". It's very annoying to be stuck with no taste of food other than the tongues ability to detect the basic tastes.

# case 0263

I smell the odor of wood burning or an electrical fire. In addition, my eyes burn all the time.

# case 0264

I had a head injury last year. For seven months I had no sense of smell. I have been using a corticosteroid nasal spray for about a month and have just now started regaining some of my sense of smell. Right now the only scent I can smell is some type of chemical smell. Maybe it is the smell of soap, because I first could discern the smell when handling soap. My sense of taste was not impacted by my head injury.

# case 0265

I lost my sense of smell when I had a really bad cold and used nasal spray. The thing that does bother me more than anything is that if I was in a fire I couldn't smell it.

# case 0266

I was exposed to paint fumes. When my sinuses finally cleared, I couldn't smell very well. Now I almost can't smell anything. Sometimes I think I can smell strong garlic or caramel-flavored coffee creamer. But that's the only smells I can smell. I still have my sense of taste.

# case 0267

I started noticing my loss of smell after I had the flu a few years ago. Over the years I didn't understand why I couldn't smell. On one occasion I asked my doctor and she didn't have an answer and wasn't too worried about it. It has worried me because I worry about fires or a gas leaks in my house at night? About a year ago I hit a skunk with my car and everyone couldn't stand being around my car. I couldn't smell it! There are times I can smell some things but most things I can't smell at all. After reading about an unapproved homeopathic nose spray that had been implicated in smell loss, I remembered using it and it burned so bad that I never used it again.

# case 0268

I lost my sense of smell after a heavy cold eight months ago. I realize if something is smelling, but cannot detect the actual odor.

# case 0269

I was walking dogs in the country when they were sprayed by a skunk. I thought the skunk was defective as they didn't stink. Weeks later I noticed a dead raccoon. He was there for weeks but no odor. Hmmm.... Then I noticed that onions and peanuts and boiled eggs tasted like crap — literally. My father had same thing about the same age. We assumed it was from working 16 years in a plating plant. I have no problems breathing through my nose. My ear nose throat doctor says they have hearing aids but no smelling aids; too bad.

# case 0270

After my husband had his first and last attack of hay fever, he lost his sense of smell. He was diagnosed with Alzheimer's soon after. His sense of smell has never returned.

# case 0271

Periodically, several times a week, I smell food cooking. I always ask people around me if they smell this odor and they always say "no". I told my doctor about this back when this first started at age 57. He found no physical cause of it. He took it lightly.

# case 0272

Ever since I can remember I haven't been able to smell. I can't smell bleach or ammonia. I can smell Vicks for two seconds before the smell goes away. I have no problem with taste. Sometimes I think that I smell something when no one else does. Maybe once a month I will smell something but won't be able to identify it because I've never smelled it before. I have a hard times with this because I can't even smell myself and people always ask me to smell something and they don't believe me that I can't smell, because I can cook. They think I can smell!

# case 0273

From around the age of 12, I have a few weeks every couple of years when it smells, tastes, and feels like my whole body reeks of petrol. It's a deep smell from within my lungs that seems to be coming out of my body rather than a smell from around me. No one else can smell this; I have asked people about this and they say it's just me. My depression started around the same time as the phantom smell. I haven't had it for a couple of years but now it's back again.

# case 0274

I began to notice more sweetness in my perfume collection about two years ago. Some perfumes became too sweet for me to wear. I could not stand the sickly sweet notes of some of what had been my favorite perfumes. Then I found out this year that I have diabetes Type 2 and I think this has affected my sense of smell.

# case 0275

I have lost the sense of smell gradually. Now, at 42, I can't smell anything! I do not know what caused this, but I had a serious head injury when I was four years old and I live in Dubai and suspect that my car's air conditioning system may have contributed to my condition. Surgery and medications did not help. I have given up hope. This is affecting my love for food and my sex libido is going down.

# case 0276

I have lost my sense of smell. I have suffered from allergies in the past and non-allergic rhinitis. My primary care doctor has told me that if I can taste I can smell. I can taste but I cannot smell anything. I have had every test imaginable but there is nothing wrong with my sinuses or nose. My ear nose throat doctor says they are perfect and I do not need any surgery of any kind. I just want my smell back. I miss it so much. My health is excellent except for this. I had several surgeries over the last years, including a gall bladder removal. I get estrogen injections and wonder if this has caused my loss of smell. I read somewhere that estrogen causes loss of smell.

# case 0277

My diminished sense of smell became apparent while shopping for candles. My friends and I would sniff the different candles and I was the only one who couldn't detect any fragrance. The same is true when sampling perfumes in a department store. Since then, I've volunteered to work with the homeless. Again, my friends can detect the strong body odors, but not me (thankfully). The last strong odor I can remember was about five years ago — diesel fuel. I haven't noticed a decline in my sense of taste.

# case 0278

About three months ago (around the same time I fractured my nose), I started experiencing phantom urine smells. I notice these smells almost every time after I take a shower. I cleaned the bathroom thoroughly and and asked my nephew, who lives with me, if he can smell anything. He doesn't, so I know it must be me. The smell stays with me as I go to work but then as the day goes on the odor goes away. Then it starts all over again the next day. It doesn't matter what time of day it is that I take a shower. Morning or night I smell urine. Sometimes I smell urine even if I haven't taken a shower. Sometimes I don't smell it after I take a shower but I haven't yet connected the odor with anything specific. Most bizarre thing to go through!

# case 0279

Immediately after surgery (lobectomy) and general anesthesia, my sense of taste and smell was altered. At first I was told that this happens often after anesthesia and medication and that it would go away in time. It's been more than three months since the surgery and nothing has changed. Tap water smells disgusting, people smell terrible, foods that I used to enjoy smell awful. I can't be in the kitchen or nearby when certain foods are being cooked or fried.

# case 0280

Seven months ago I fell downstairs and hit my head causing a concussion. After a couple of weeks I suddenly realized that I had lost my sense of smell. It has still, sadly, not come back. I do mourn my lack of smell and I do suffer from a sensation and taste at the back of my throat, from time to time — usually after I drink something.

# case 0281

Twelve years ago I was diagnosed with asthma and put on a mild steroid inhaler. I began having nose blocks often and gradually lost all sense of smell. Only when my asthma becomes acute and I have to be put on oral steroids does my sense of smell return. As my asthma gets better and I am taken off oral steroids, the nose block returns and I lose my sense of smell again.

# case 0282

For the past two months or so I smell smoke all the time. I am unable to smell the regular things like cologne or room fresheners. It seems as if something is burning all the time.

# case 0283

I noticed that I lost my sense of smell about a year ago, first subconsciously, then consciously, and then it was confirmed by a test at an ear nose throat doctor. I also suffer from very painful and very frequent migraine attacks during which my nose hurts badly. Medication helped with the migraine but the inability to smell remains except for very short reprieves where I smell something, but normally can't even say what it is. I am worried about eating and I miss the enjoyment. I also can't smell burning toast and don't like what this implies. On the other hand it's a relief not smelling everything.

# case 0284

I hit my head about five years ago and fractured the base of my skull. I lost my sense of smell and never regained it. I can tell if there is a dramatic change in smell but only that there has been a change not what it has changed to. Food pretty much has different degrees of tasting horrible as I seem to have one taste for everything be it toothpaste or a piece of chicken. Sometimes I think I can taste something like an orange but I think that really is from memory and not that I can actually taste it. I have not come across anything to make it any better and by now I can tolerate the horrible taste. But I will never fully get used to it. I just let myself get so hungry before I eat that the taste does not matter. It's just for fuel.

# case 0285

The only time in my life I have ever smelled is when I was pregnant. Naturally, since I never had this sense I did not know anyone "smelled" differently than I did. My first smell experience was a bus on the street. I finally understood what everyone was talking about — how nasty! I have seen doctors about this and their response was: "why not get pregnant again so we can test you". Eating is a challenge and I have eaten more rotten food than I care to share. Imagine not knowing if the milk is bad, when your child has shit their pants or that you stepped in dog shit. Now think about the bad smells and tell me there are days you would rather not have this sense as well.

# case 0286

I had what I thought was allergies and noticed that my sense of smell and taste was a little off. After recovering from the allergies (that I thought I had), I continued to notice that my sense of smell just wasn't the same. Not only was my smell off but it occurred to me that all foul odors smelled the same, kind of like a medicine/wood polish type of smell. I also began to taste the same smell occasionally. I am unable to smell my farts or when I go to the restroom. If I do smell anything it is the medicine/wood polish smell. Four months later I still cannot smell anything as before.

# case 0287

Two years ago I had an accident that left me unconscious for several days. I have recovered except that I permanently lost my sense of taste and smell. I only realized that I lost my sense of smell when I came home from the hospital and my husband was cooking the Christmas ham. I am a chef and losing my sense of smell has been a major drawback. Seasoning is now a guessing game and I have to rely on others. I know what's in my mouth is sweet or savory. But I can't identify flavors like cinnamon, rosemary, sage, or thyme. Food had become bland. I also feel like I am a health and safety risk in the kitchen as I cannot smell gas or anything burning on the stove.

# case 0288

I noticed my sense of smell changing in my 40s. I smelled burning rubber for a year and that stopped and now I smell only a few things like people who are wearing smelly, sweaty clothes. I can smell things if they are pressed right to my nose, but if it is even an inch away and the smell is gone. I can't smell odors around the house: mildew, mold, or cat urine. I don't smell food cooking and therefore burn a lot of my dishes because I used to rely on the smell to note whether it was done. I smell nothing at work, nothing outside, no fresh grass clippings, no flowers, no gasoline. My sense of taste is muted and I now prefer strong spice. It's really very sad.

# case 0289

I have a problem with my sense of smell. Since my childhood I find it difficult to smell. I am worried because I can't smell anything which is putting life to risk.

# case 0290

I had a very severe cold that resulted in an abnormal production of mucus. I used nasal sprays which helped a little. However, after the cold cleared up I had no sense of smell. I consulted an ear nose throat specialist. He gave me a cortisone injection to rule out nerve damage. I regained smell but this slowly disappeared. Sinus surgery made no difference. The odd thing is that just after having a cold I often have a period during which I can smell! It doesn't last long but it makes me realize my body can "cure" me. Possibly reduce swelling in my mucosa with the production of natural cortisone. I have never used a cortisone nasal spray for a long period as I have really given up on this very sad state of affairs.

# case 0291

I have never been able to smell farts. When I was in elementary school and people farted on the bus, everyone would say "eeeewwww" but I would never be able to detect the odor they were reacting to. Even now my boyfriend farts regularly and even after he stinks up the bathroom I am unaffected. It's funny because they are just funny noises to me, nothing more. I do not pass gas much but even when I do I cannot smell them either. I can smell "car farts" and sulphur-type odors outdoors near swamps and marshes but not human farts.

# case 0292

Two years ago I was very sick for a week or more. I used nose spray to clear my nose. Afterwards I noticed that I wasn't able to taste or smell. Unfortunately, I could still smell bad things like cigarette smoke, body odor, gasoline and similar stinky smells. On rare occasions I smelled other stuff but it was only for a moment and didn't last long enough for me to identify the smell. It was bad when I would eat with other people. They always ask about my food or my family would ask me to taste something and I would have to explain again that I still can't taste... It seemed that in some cases my loss of taste and smell took away their pleasures, so I just started lying if someone asked me how my dinner was. If my grandmother asked me to taste something, I would say "oh that is very good". Lying was easier than trying to explain that I ate textures now, not food. Trying to explain that I could almost always tell if something was sweet or salty or sour but had no flavor is like trying to describe the color white. Today, I tasted cantaloupe and barbeque! Now, I can't wait to start cooking again…trying new recipes… I want to eat all the foods that I have missed for the last two years tomorrow, in case my sense of smell will disappear again.

# case 0293

I started smelling cigarette smoke last summer. For a long time I thought it could be explained by people secretly smoking around the house. But recently I've had a slight smell like old cigarette butts which I thought might have been coming from my mouth. It gives me a bad taste in my mouth as well. Then, yesterday I was in a large auto supply/repair facility and I strongly smelled cigarette smoke for the many hours I was in there. It came on suddenly and was very strong. No one else smelled it but me. When I came home I looked it up on the internet and was surprised to find so many people dealing with this same thing. The one thing that I noticed with all the other people on the internet forum that I found online is that others dealing with the same problem, like myself, all have a history of some type of respiratory problem, whether it's bronchitis or sinus problems. Either way, this is definitely quite bizarre.

# case 0294

Seven months ago I began to smell an earthy smell that I thought was in the air. Then I realized that I smelled it indoors and outside. When I smell my skin, it has the same odor. As an experiment, I held a blanket up to my nose and the smell was stronger, so I deduced that it was something inside my nose. I saw my doctor yesterday. She thinks it's a sinus infection. The odor is always there and tolerable but annoying.

# case 0295

I had a very bad cold at about age 18. After that for a couple of weeks everything around me smelled awful, even the water I drank. That happened a couple of times over the years, but never that bad. Then, I had chemotherapy for breast cancer when I was 45. That caused some problems again. Especially when food was hot it smelled so very bad. But, even fabrics smelled bad. Now I'm 64 and taking chemotherapy for ovarian cancer and one of the most distressing problems is the bad smell of my food, and the fabrics in my home. I take chemotherapy treatments each week so there are very few days that I don't have this problem.

# case 0296

I had pneumonia three months ago. I notice since then that my sense of smell is weakened and less discriminating. I am wondering if this will be for good.

# case 0297

Sometime around the middle of seventh grade, my sense of smell degraded. I probably had a cold, and recovered without noticing my partial anosmia. I get colds a lot — three or four times a year, usually lasting between two weeks and a month. Anyways, my sense of smell seemed to be getting better about a year after it first worsened, but then I went back to partial anosmia. I can usually smell fine — if what I'm trying to smell is within an inch or so of my nose. I can smell stronger smells from farther away, and I can pretty much always smell the more penetrating scents (such as dog feces).

# case 0298

From what I can remember, I never had a sense of smell. Once in a while, I could smell gasoline. I remember one time when I smelled ham baking in the oven and knew what that was. Other than that I never smelled anything. I can't even smell a skunk. I have had sinus problems throughout my life, but not bad enough to need medication all the time.

# case 0299

About a year ago I had a head injury from an accident. Over the next few days I slowly started to recover. One day when I was starting to feel better my sister asked me to smell one of those perfume ads that are enclosed in magazines. I couldn't smell it. Next she got scented candles, garlic, and just about every other odorous object she could find in the house. I could smell nothing. For some time I had noticed that food didn't exactly taste right, but I didn't care much at that point. It suddenly became very clear: I had lost my sense of taste and smell. I asked the neurologist if this was common, he said it can be a result of head trauma. He offered me some optimism and informed me that it could return within the year. Life has been difficult over the past year. I still have no sense of taste or smell. I am told that after a year it is not likely they will come back. A year and a half ago I was training to be a sommelier. Today I am unemployed, unhappy, and inconsolable.

# case 0300

When I was 19 I was on a streetcar that was passing a slaughter house. I noticed a lot of the streetcar passengers were covering their noses and from their facial expressions, I knew the stench was pretty bad that day. That is when I first noticed that I could not smell anything. I did see a doctor about this concern but nothing was really known so I have just put up with it all my life. It was really great at "diaper" time but not so good when food was burning.

# case 0301

I was in a road accident four years ago. I fractured my skull and was in a coma for a week. I noticed at a later date that I had lost my sense of smell.

# case 0302

For twelve years I have had periodic phantosmia episodes. The episodes are triggered by coughing, shouting, and sneezing. While the common denominator here appears to be a sudden increase in pressure within the sinus cavity, introduction of water into the nasal passages/sinus cavity (underwater swimming, saline nasal rinse, etc.) can also trigger an episode. In rare cases, onset is spontaneous with no apparent triggering event. The phantosmia episodes will always last the entire day. Each episode has resolved only after a full night’s sleep. Frequency of occurrence is three to four times per month, sometimes spaced apart as closely as two or three consecutive days. The smell consistently associated with these triggers is of rotting garbage and the episodes themselves are frequently accompanied with an emotional state characterized by irritability and a sense of depression, given that the day is largely ruined by the temporary loss of the two major sensory inputs of taste and smell.

# case 0303

My sense of smell began fading over the course of a year. Ironically, it occurred during the same year that I quit smoking. I had a couple of colds/sinusitis symptoms before and after onset. The last odor that I experienced was a faint whiff of peanut butter after opening a jar. Since then, I have had no sense of smell whatsoever. I was evaluated by an ear nose throat physician. Cause was ruled possibly viral, but inconclusive.

# case 0304

I was born without a sense of smell. I didn't tell anyone until I was in my 30s.

# case 0305

I had a particularly vicious viral infection two months ago. Following recovery I noticed no more smell or taste. I took antibiotics and prednisone, but my condition did not improve. I can pick up a "sense" of very strong odors (incense); very spicy foods trick my brain to sense flavor but I don't taste them. I'm not depressed.

# case 0306

I noticed that I was losing my sense of smell a couple of years ago. Last year I started to get an unusual smell in my head. It smelled like I was stuck behind a school bus all the time. I would sometimes wake up with the smell. This smell has since gone away. The specialist said everything looked fine and there was nothing he could do. Now I virtually have no sense of smell and sometimes I get a rare taste of what I am eating.

# case 0307

Eight years ago I have undergone an operation due to meningioma that affected my eyesight. The tumor was right in front of the meninges that is covering the optic nerve. About a month after the operation my sense of smell started diminishing. Up to this day, my sense of smell is completely gone.

# case 0308

I first found out I could not smell in elementary school. My friends were sniffing scented coloring markers and judging whether the description was accurate or not, which was strongest, and which they liked the most. I could not smell any of them at all. I simply pretended that I could smell, following along with what others said or simply reading or identifying the photo on the side of the marker.

# case 0309

Just last week I started to think someone around me was smoking. This was while I was in my cubicle at work. I thought cigarette smoke from outside came into the building through the cooling system. Now at home today I am starting to smell the same thing. I am not sure what to think now.

# case 0310

Since I was a child I've suffered from seasonal allergies. Over the last two years, I've noticed my sense of smell rapidly decreasing. Today, I am not able to smell anything, even odors that would alert to danger, rotting garbage, spoiled food, smoke, animal excrement, my own body odor (after exercise) or excrement. Nothing! Strong odors such as those inside a hobby store where the candles, potpourri, and fragrant oils are kept go undetected by my nose. These types of overpowering, artificial scents would have given me great headaches in the past. I think I've gained weight as a result of my loss of my sense of smell. I get the taste for certain foods, but my appetite isn't satisfied because I cannot fully enjoy the foods I eat because I can't smell them. A sense of smell may seem like no big deal to some, but I truly miss it and am self-conscious that I will not notice offensive odors in my own house or on my person while others might.

# case 0311

I had sinus problems and used nasal spray. My sinus improved but several months later I discovered that I have lost my sense of smell. Occasionally I regain my ability to smell, but only temporarily.

# case 0312

Seven years ago I had a very bad cold. I took the standard cold medicines. I was also taking a steroid regimen to try to push my eczema into remission. After five days I was beginning to get better. My nasal passages were starting to clear. I picked something up to smell it out of habit and realized I couldn't smell anything. Most smells that come through do not smell like they are supposed to smell. The most common scent is mold/mildew. Salad smells like mold, melons smell/taste like mold. Needless to say the lack of smell greatly affects my sense of taste. I cannot discern between chocolate and strawberry ice cream for example. I now find I eat for texture rather than taste. Crunch is good. What I continue to find strange is that when I get a craving, say for a hamburger straight from the grill, and I fulfill that craving, I find that I am completely satisfied even though I didn't taste one bite.

# case 0313

Eleven months ago, I suffered brain trauma as a result of a fall. All of the problems I had after the fall have improved over time except my sense of smell which I have lost completely. It has not returned. Doctors tell me that it will not come back.

# case 0314

When I was 10, I underwent open skull surgery for aspiration of subarachnoid cyst in my brain which was above the hypothalamus and pressing on the optic nerves. After the surgery, although I had not noticed it first because of more severe problems, I found out that I had totally lost my sense of smell.

# case 0315

I never had a sense of smell. I've been to so many doctors here in New Zealand but they said that we don't have the resources for treatment available here.

# case 0316

After a hoarse throat and stuffy nose four years ago I lost my sense of smell. My doctor, allergy specialists, and acupuncturist could not help me. I still have no sense of smell.

# case 0317

I don't remember ever having a sense of smell. For me, trying to understand how something smells is like a person who has been blind since birth trying to understand colors. I cannot comprehend it. When I was younger it never really mattered. I can't smell, but I can taste. I don't know if my sense of taste is the same as other peoples'. As I've grown older, I always thought I'd never want to have it fixed. I always thought it would be some sort of "sensory overload" — always catching whiffs of things and never being able to concentrate, constantly being distracted. Not long ago someone commented about the idea of pheromones. They said that a big part of being attracted to someone is the scent of pheromones. Recently I have started to take a greater interest in this idea, seeing as I'm getting older and have always been curious about my struggles with lasting relationships. Basically I have become afraid: does my lack of sense of smell keep me from finding someone I'd like to spend the rest of my life with? Is there a relationship between anosmia and not detecting pheromones, causing one to fall out of "love" or "like" with partners very quickly?

# case 0318

My mother, who is 83, is not well. Her sense of smell is so sharp that we can't even do the laundry. She won't eat because when she smells the food it upsets her stomach.

# case 0319

Since I was in primary school I pretended to have a sense of smell. Until today I have never told anyone that I don't have a sense of smell. It is really bad because I sometime throw away food since I cannot tell if it is bad or not. I have never gone to a doctor because I am embarrassed.

# case 0320

I lost my sense of smell in a car accident 56 years ago. I have not been able to taste or smell since then.

# case 0321

My sense of smell changed. I now have three different smells: (1) normal (grass, lemon, etc.); (2) a harsh chemical smell (when I smell punch flavored Crystal light, all refrigerators smell like that, berries smell like that); (3) the smell of ashes from an ash tray. The last smell appears for some foods, when I smell the sewer, certain foods have the taste: some beef, popcorn, butter, egg whites. The change in my sense of smell has affected everything in my life.

# case 0322

One day I experienced an unbearable rotten smell. I found that the smell is coming from wet rotten onion. From that day on I smell the same kind of smell all over the place. It is very difficult to define but perfumes, incense sticks, and fresh food all smell to me similar to that dirty onion smell. I am panicking over this unusual problem.

# case 0323

Several weeks ago I started to smell cigarette smoke that was not present in my environment. This has progressed over time and I can wake up in the night and smell it in my own bedroom. No one has ever smoked in my house. I work in a smoke-free building and live in a state that does not permit smoking in public places. My family and friends do not smoke so I am very rarely ever exposed to cigarette smoke. Four months ago I had the worst upper respiratory infection that I have ever had and I remember thinking that the symptoms were very strange. I also gag up greenish yellow mucus several times daily.

# case 0324

I had phantom smells about 20 years ago. First, I experienced the smell of spices burning which lasted a few days or a week, then the smell of books, then garbage, and a few other smells I don't remember. Several years ago, I noticed that my sense of smell was going away. Try as I might, I couldn't smell strong flower smells. Sometimes I could smell some roses and lavender and wisteria. Now there are very few things that I can still smell. Just recently I have noticed that I don't really taste much beyond salty or sweet or tart or spicy. I still enjoy eating though for some reason. I find it a bit depressing not to be able to smell — although among the things I can't smell are dead rodents, skunks, and baby poop.

# case 0325

I suffered a short but bad cold a few months ago. I lost my sense of taste and smell and haven't regained it yet.

# case 0326

Two years ago I had colitis and was put on medications. When I was released from the hospital everything smelled horrid....fresh towels, pizza, perfumes, smells in my friend's house, smells in the mall and I could go on and on. It wasn't that I lost my smell, my smell became extremely acute and it seemed that things that normally smelled nice to me became awful and the awful things (baby's poop, urine, fish, and garbage) became 100% worse and totally disgusting. This extreme sense of smell lasted almost six months and gradually became lessened. However, I still have a very acute sense of smell. I can smell things that other people cannot smell, both good and bad.

# case 0327

I first noticed that my sense of smell wasn't as it should be after the birth of my first child. During the pregnancy I had an overactive sense of smell as many pregnant women do. After my baby was born, my sense of smell became impaired. The example that sticks in my mind the most is that people would tell me when he needed a diaper change, and I couldn't smell it unless I really tried. Over the next few years my sense of smell slowly started to fade away, and within about four years it was gone completely. On occasion I think that I may be able to smell something, but sometimes I wonder if it's the memory of that particular thing that I am "smelling"? I also get the phantom smells as many anosmics do, and sometimes this can be pretty terrible. At times I have had a very strong perfume smell stuck in my head for days, causing a constant headache. I have gained a substantial amount of weight and I am wondering if it is because I am never fully satisfied as a person with normal smell and taste is. I won't get into the difficulties and sadness anosmia has brought to my life (like not being able to smell my babies) because I could just go on forever about that. I got a clean bill of health from an ear nose throat doctor and an allergist. The ear nose throat doctor chalked my condition up to being caused by a virus, although I wonder about this since I don't recall having an infection prior to losing my smell, and from what I have read it seems that anosmia caused by a virus is usually a sudden loss, not gradual.

# case 0328

13 years ago I had a terrible bout of depression and noticed I could not smell. After taking antidepressants and coming out of the depression, I noticed that my sense of smell was returning. However, my sense of smell is not as sharp as it was before.

# case 0329

32 years ago I moved and realized that I couldn't smell the orange blossoms that other people said had an overpowering scent. I had smelled the blossoms in earlier visits. I started paying attention and realized my sense of smell was gone. My doctor speculated that a past sinus infection had destroyed my olfactory nerve. Early in my awareness of the problem I could smell a few things — jasmine, some perfumes and colognes, lemons — but not with any regularity. Now I essentially have no sense of smell left. Perhaps every three months I'll catch the merest whiff of something — like newly cut grass or something baking in the oven. I cannot smell natural gas or propane. Once, thirty or forty years ago I had a three day episode, not accompanied by a cold, of coffee and chocolate smelling just awful, and tasting worse. At the time I wasn't aware of any precipitating event. It would be wonderful to be able to smell again, although I feel that I get plenty of enjoyment out of eating. I smelled for long enough that I know I'm missing an important part of my life.

# case 0330

I fell and hit the back of my head. The next day I noticed that my sense of smell was completely gone. There was no improvement in the two weeks since.

# case 0331

A few weeks ago I started to smell something that reminded me of vermin. It smelled like a rat in just one part of my house. Now it smells like this everywhere I go: village halls, other peoples' homes... Anywhere I can smell this horrible smell. What has happened to my nose or sense of smell?

# case 0332

I don't ever remember being able to smell. I went to a specialist, and he told me I must have had a bad infection as a child. It worries me. I cannot even tell if the baby is dirty.

# case 0333

I fell ill with typhoid fever and later suddenly realized that I couldn't smell the cigarette smoke from a person sitting next to me. I didn't take it seriously at first and when I went to the doctor years later, I was told that there is no cure.

# case 0334

I was a fireman for twenty years and prided myself with a very acute sense of smell. I could pick out most things being burnt. I originally put the loss of sense of smell down to a dose of influenza but due to it not returning I now doubt this is the case.

# case 0335

I developed allergic rhinitis as a teenager and in my twenties noticed a change in my sense of smell and taste. I used to smoke but since going off cigarettes the sense hasn't returned. I have some degree of smell just not much. What I miss most is my sense of taste. I also worry sometimes that I may smell bad and not notice it myself.

# case 0336

After an accident two months ago I lost my sense of smell. It really hurts me that I can't smell anymore. My sense of taste also got poorer.

# case 0337

A few months ago when in bed, I smelled cigarette smoke. This happened every night. For a while I thought I was getting a signal from my aunt who had died of throat cancer. However, it has continued for months now and now it is also happening during the day. I am now trying not to take deep breaths as it feels like I am inhaling cigarette smoke. It is a very strange feeling.

# case 0338

Ever since I have been diagnosed with a kidney infection I have been noticing that my sense of smell has been off. Some things are normal to me, but others are so strong that they make me choke and gag. Everyday things like onions, chicken, cigarette smoke, and other smells I had been used to now make me sick to my stomach. I have to leave the house when I smell them.

# case 0339

I have phantom smells; I can't smell some things and some foods do not taste right. I couldn't smell when they were pumping out the septic tank or a dead skunk. I also have a taste problem. Chocolate mint ice cream tastes like a bitter chemical and fresh food doesn't taste like it should. I will think that some food item has gone bad and everybody else disagrees. The smell of lettuce is very bad. I am concerned that this is why my irritable bowel syndrome is so out of control.

# case 0340

In the last two years a phantom odor of stale urine has occurred a handful of times. It is so strong it wakes me up in the middle of the night. At first I wondered if I'd had an accident, but that would've been bizarre since that hasn't happened since I was 6 years old. I can never figure out where the smell is coming from. After a few minutes it disappears completely. I am left wondering if I'm going crazy!

# case 0341

I had a fever when I was 18. From that time my nose stared troubling me and now I can't smell anything, even when there is dead rat at front of me. And there is this smell in my nose.... I am 24 and I don't have any hope... I lost everything... it smells so bad that people don't want to be near me. I don't go anywhere; just sit in my home doing nothing. I lost my job. Because even I can't stand this smell. What is this smell?

# case 0342

I had a major concussion after a car accident. About a week after that, I discovered that I could no longer smell anything. Now, about six years later I still cannot smell anything and, within the past few months, have noticed a decrease in my sense of taste as well.

# case 0343

I got what I thought was a sinus infection. Three days later my sense of smell and taste was gone. My ear nose throat doctor told me it was a viral infection and my sense of smell probably won't come back. It's been almost five months now and it is very depressing. I can smell some soaps, lotions, and oregano. I can't taste most things. Coffee and chocolate taste horrible!! It is very confusing.

# case 0344

I had polyp removal surgeries and after the second surgery I noticed a complete loss of smell. I mentioned it to the surgeon but he denied that this could have been a consequence of the surgery. Whenever I am put on a prednisone regimen, by the third day my sense of smell returns. So I am encouraged that "someday, someway" I will be able to recover this most intriguing of senses.

# case 0345

I had sinusitis for several years, usually following a cold or flu. Three months ago I lost my sense of taste and smell. It improved, but after I discontinued the antibiotics and the steroids I lost my sense of smell again. A few weeks ago I had sinus surgery and since then my sense of smell comes and goes. This is a very difficult issue for me. I would rather lose my left arm than my sense of smell.

# case 0346

Two years ago I was sick. I had a sore throat. I used Vicks and an unapproved homeopathic nasal spray. As a consequence of the infection or the treatment I lost my sense of taste and smell. There is nothing wrong with my sinuses but I still can't smell or taste.

# case 0347

I smell cigarette smoke for almost one year anywhere I am; work, school, car, home. When I am driving I have to lower the window because I can't breathe well. When the smelling started, I started burping a lot.

# case 0348

I started to lose my sense of smell when I was pregnant with my son. I just thought it would come back, but it never did. On rare occasions I will smell certain things. I also sometimes smell a burnt odor that no one else can smell.

# case 0349

When my dog was sprayed by a skunk but wasn't malodorous I thought the skunk was defective. Then a dead raccoon by the road had no odor. As the summer progressed, I also noticed the absence of flower smells. Now, cucumbers and watermelon taste like vomit. I can still detect the ammonia when they are cleaning the manure pits. I also can smell sulfur compounds in onions, but don't enjoy the smell of onions anymore. Beef always smells unpleasant.

# case 0350

Three months ago I suffered a fractured skull and two frontal lobe brain lesions. I was in a medically induced coma for two weeks. One of the enduring consequences is that I cannot smell a thing. It has been explained to me that as my brain slid forwards and backwards in my skull, the nerves dangling from my olfactory gland into my nasal cavity were sheared. I can't smell gasoline, food, nail polish remover, etc. I lost 20 lbs. while in the coma and not being able to smell has greatly reduced my appetite. The weight has not returned.

# case 0351

I have really bad nasal allergies. My nose has been congested for most of my life. In the last few years I noticed it has gotten even worse, my breath smells bad and I have constant sinus drainage. One day several years ago I was at the movies with some friends when suddenly things began to smell bad. I asked my friends if they could smell that awful smell and they could not. That lasted for about two and a half years and for about one year I seemed to smell people's fear or more so anger. Almost like schizophrenia if I didn't know better. My smell is so heighten that if someone has been a place and left for several hours I can still smell them. It is so bad the odor lingers in my nose until I use a saline or hydrogen peroxide rinse.

# case 0352

It has been eight years since I smelled something. I think it may have been caused by a sun stroke. I can't remember smells, it's all air now to me. I haven't consulted a doctor yet.

# case 0353

About four months ago, I had a bad cold. Ever since then I can smell cigarettes. I smell them at work, church, in my car and there's none anywhere.

# case 0354

I've never been able to smell. I can't remember at what age I realized I couldn't smell. I was on a family trip in the car and all my family members were complaining about smelling a skunk. I couldn't smell anything; just air. I just recently read some stories about people who can't smell and that's exactly how I feel! I never had it so I don't really miss it. I can't taste food that well either. If my eyes were closed, I couldn't tell you what I was eating but might be able to guess because of the texture of the food. I can't tell what ingredients/spices were in a meal. I can't tell if liquor is in a drink. It just tastes different. My poor kids sat in dirty diapers longer than they should have because I couldn't smell the soiled diaper. Once I left by accident a gallon of milk in my car for over a week in the summer. I had no idea it rolled out of my grocery bag and under one of the seats. When my husband got in the car, he almost died of the stench. I've put on way too much perfume many, many, many times. I shower every day because I'm nervous I might have body odor. I always have mints in my mouth or chew gum in case I have bad breath. I do get nervous about gas leaks, eating spoiled food and smoke. Friends and even family members always forget I can't smell. I don't even think my doctor knows I can't smell/taste. It's one of those questions that I'm never asked and I never think twice about telling a doctor because it's just a part of my life. I've never had it so I guess I don't really miss it.

# case 0355

About three years ago I came down with a cold. It went on for about a month and after the cold passed I have not been able to smell. I can still taste, though. I often do wish I could smell. I had my second child a year ago and have not been able to enjoy that new baby smell or anything. Strong smells like pickle juice I can smell. Anything else I try and smell, but I can't.

# case 0356

I noticed about three years ago (when I was 13), that I could not smell anything. For example, say our family drove past a skunk on the road and instantly started complaining about the smell...I replied with: what smell? To some degree, not having a sense of smell is a good thing.

# case 0357

Seven months ago I had malaria fever and as a consequence I lost my sense of smell.

# case 0358

Half a year ago I was hit with the worst sore throat ever that culminated into my being unable to breathe. I spent a day in the emergency unit and woke up the next day with a very bad cold that lasted for about 4-5 weeks. I was prescribed a nasal spray at some point and used it. During this period I experienced a loss of my sense of smell and taste. Occasionally, I would smell inappropriate smells such as cigarette smoke for several days — this was particularly revolting. Now everything, including water, tastes and smells horrible. It tastes as if a small animal has nested and died in my mouth! I usually drink loads of water but I almost have to hold my nose to get any down. I can taste salt however and tend to want only salty things. My shampoo, bath soap and toothpaste all smell very odd. I have bouts of occasional hawking where I feel there is something in my throat.

# case 0359

About ten years ago I started to notice that I smelled things that other people weren't. It was usually a garbage smell or cat urine. My family used to tell me I was crazy. As time has gone by, my sense of smell has gotten worse. My ear nose throat doctor told me that my sense of smell was somewhat diminished, but that there was nothing that could be done. In the past few weeks, my sense of smell has almost disappeared over night. I am very discouraged as you don't realize how important smell is until you don't have it any more.

# case 0360

I had extensive nasal polyps. Sinus surgery resulted in the loss of virtually all sense of smell. It has been seven weeks since surgery. My sense of smell was perfect with the untreated polyps.

# case 0361

Since my 20s I had frequent spells of colds and nasal blocking at night. Later, I realized that my olfactory sense had considerably decreased. I underwent corrective surgery for deviated nasal septum — after which the sense of smell was totally gone. I am now unable to perceive even very strong odors.

# case 0362

Several times a day I have a cinnamon smell in my nose.

# case 0363

I had a lot of earaches as a young child. They improved greatly after removal of my tonsils. But every time I got a bad cold, it went straight to my ears. One of my eardrums burst in my early twenties. When the second one burst, I lost my sense of smell. It came back at times but always left when I had the slightest congestion. I then had chemotherapy and lost my sense of smell for over a year afterward. It now comes back, at times, but is never consistent.

# case 0364

I started to gradually lose my sense of smell about 14 months ago. I am currently going through menopause.

# case 0365

I have persistent asthma attacks. I also had pneumonia and other lung infections. In the last lung infection my sense of smell deteriorated. In the beginning I thought it was because the infection, but four months later it did not get any better. There are no polyps in my nose and my doctors are thinking it could be related to anxiety or stress.

# case 0366

I lost my sense of smell after the olfactory nerve was cut during a cancer-related trepanation surgery.

# case 0367

In the last month I have had some days where I smell stuff that's not there. On some days I smell wood smoke and on other days I smell something akin to diesel exhaust. This happens about every third day.

# case 0368

I cannot smell unless I hold something up close to my nose. I can smell my own perspiration slightly. I can smell ground coffee. I can't smell my dog's breath even though other people say he has bad breath. I have a slight smell of flowers if I put my nose right in them. I worked in a factory in which I was exposed to chemicals that irritated my sinuses. I really want to smell again.

# case 0369

I fell backward and hit the back of my head hard enough to lose consciousness for a moment. I spent two days in the hospital with a concussion and skull fracture. At a follow-up I was told that I had destroyed my olfactory nerves and that I more than likely would not recover my sense of smell.

# case 0370

Almost 30 years ago I started disliking musk-based perfumes. Through the years I also started disliking all oil-based odors, including paint thinners and solvents. Engine fumes and tobacco fumes became very ugly and annoying during colds and flues.

# case 0371

I can smell the air and it's driving me crazy. It smells like smog. The smell of lilacs in a small room is overwhelming.

# case 0372

I smell smoke off and on when there is no smoke present. Sometimes it actually seems to burn my nose just like real smoke. It comes and goes but seems to be getting more frequent. I think it began about a year ago. It is annoying but otherwise not causing any problems.

# case 0373

After several months of sinus infections, colds and continual sinus drainage I have lost my sense of smell and taste.

# case 0374

About two years ago I started to notice that my sense of smell and taste was not what it is supposed to be. I can still smell some things but nowhere near what I should smell. My taste buds are also not what they should be but I can still sense different hot spices.

# case 0375

I have an abnormal sense of smell and an almost complete loss of my sense of taste since a cold 2-3 months ago.

# case 0376

I started to lose my sense of smell gradually and now I realize that it is not coming back. I smell nothing except for rarely I smell smoke from a fireplace or pine trees. I only taste sweet and salty. I like textures in foods.

# case 0377

I have never had a sense of smell but can taste food. I would love to be able to smell one day.

# case 0378

I had a really bad cold/cough for one week nine months ago. By the end of the week I had completely lost my sense of smell and taste. I thought it would return soon as this has happened before when my nose has been blocked with a cold. It has not returned and I have not regained my sense of smell but I have noticed recently that my sense of smell/taste is returning but is distorted. Some things have an "ethylene" type taste. Nothing tastes as it should, it is as though the wires are crossed in my sense of smell or similar to being color blind! I did notice this morning that I could smell the true scent of my perfume so I am hoping it will return to normal eventually.

# case 0379

I gradually lost my sense of smell. It has not disappeared entirely, yet. I can still smell stronger odors. A strange phenomenon is that strong odors that I detect persist long after their source is no longer present — this "persistence" of an odor may remain for periods up to an hour or even longer.
I sneeze a lot and have blocked nasal passages at night.

# case 0380

I have noticed over the last few years my lack of smell. Strong lotions and perfumes that are overpowering to others barely register for me. Someone will say "that smells great" if I'm cooking or have something on my skin and I can't smell it at all. I just read online about a smell area of the brain and how a good "conk on the head" could affect that. I think this is what happened to me. One of the weirdest things I've experience is that I often smell a chemical smell...like plastics. I can't smell anything, but I smell a plastic chemical smell as if it's right under my nose. It happens a couple times a week and it's not dependent upon lotion or make up or anything.

# case 0381

In the last month I have noticed a certain smell that I find very unpleasant. At first I thought it was new coffee my husband had bought. Then I noticed the new cashews smelled the same. Then the peanut butter. Then pizza crust. Today it was cantaloupe. Interestingly, the food tastes like what the smell is. It's quite nauseating actually.

# case 0382

While cooking using a charcoal oven I inhaled heat in an attempt to blow the fire that was dimming. Since then, whenever I smell something I get the pungent smell of the dish that was being cooked. This has been going on for a month now.

# case 0383

I suffer from phantom smells. Every so often I'll start smelling something like overheated chemicals. I can't identify the smell, or tie it to anything I remember — it's a chemical smell, with oily undertones, and it smells like something's overheated (but not burning). I'll smell it everywhere, and I don't acclimate to it — it's not like perfume, where you smell it for a while and then it fades. And for me, it'll stick around for days or weeks, and then go away for months at a time. I might smell it just a little every so often for a short time — like fighting off a cold — but every couple of years I'll have a full-on case of it, like right now. A few years ago I started taking lots of supplements, and it seemed to go away — slowly — and I'll probably do that again. It doesn't affect my sense of taste, oddly enough. I was able to eat a bowl of chocolate ice cream last night, and I tasted the chocolate just fine, even though I smelled the smell at the same time. It's really aggravating just because it never goes away. Well it goes away, but while you have it, almost every breath in means smelling that smell. It's not a rotten smell, or completely repulsive, but it's unpleasant, and it's darn near every inhale. There are worse things in life, but it's really aggravating.

# case 0384

Some eleven months back I fell down and I think I got some unseen injury on my head. Four months ago I lost sense of smell. My ear nose throat doctor told me that there is no problem.

# case 0385

I have never been able to smell. I thought that the sense of smell became more pronounced at puberty and when mine was delayed I became worried. At 20 I was diagnosed with Kallmann's Syndrome and was put on hormones. I married and my wife and I had children. I eventually went back to my endocrinologist and was tested again. He said that I did not have Kallmann's. So my lack of smell is still a mystery to me.

# case 0386

I had a bad case of the flu and afterwards my sense of smell was all up the shoot. I could smell but could not distinguish the scents. Walking past the soap display in the supermarket nearly made me ill. Anyway it took ten years to come back about 80%. Now I have good days and some not so good days. It has never gotten me down but it would be great if it improved.

# case 0387

I cannot smell anything except strong odors of colognes and a lot of candles. When I smell those, I have difficulty breathing. I can taste some food but not all foods.

# case 0388

All of a sudden I cannot handle any perfumes/scents with an unidentified chemical... perfume, men's cologne, dish soap, fabric sheet, all smell exactly the same to me: very acidic. I also cannot drink any type of soda as it tastes just like the scents smell... I also sometimes experience burning eyes and severe headaches.

# case 0389

I've most likely been born without the ability to smell. I don't really care since I don't know what smell is.

# case 0390

When people complained about strong odors, I noticed that I couldn't smell anything. One night I was in a room where construction workers had been working earlier that day and had applied a finish coat on the floor that evidently had a very strong odor. I did not even know there was a smell. That was when I realized I had no sense of smell at all.

# case 0391

Recently I started smelling smoke. At first I thought that there was smoke in the air, or someone who was smoking nearby as the smell was not constant. For the past week I have been smelling smoke constantly. No one else smells anything out of the ordinary. My throat and eyes burn as if there is actually heavy smoke in the air. I suffer from migraines and am wondering if there is a link between the two.

# case 0392

I have a strong smell of smoke bothering me. Nobody else smells it. I even had the oven checked out, as I thought that might be the cause. The smell is not around me all the time and I can smell the normal smells, like perfume, cooking, flowers, and the smell of earth after rain. But in addition to these smells the smell of smoke seems to envelope me 75% of the time, even in the car. Now that I know that it is just me, it is not so threatening anymore, just very irritating.

# case 0393

Little by little I noticed that I could not smell a rose. By the age of 75 I could not smell anything. I had no head trauma, I just lost my sense of smell. It also affected my sense of taste. I can taste sweet, sour, and salt, but not vegetables.

# case 0394

I developed a sense as if something was burning or foul in my bedroom. I would ask people to come and see if there was something odd smelling in the room. No one ever found anything. Then I went to visit family overseas and found the smell there as well. This went on for almost one and a half years. Then slowly my sense of smell has almost vanished. I can still smell strong smells. My taste has also diminished somewhat.

# case 0395

Two years ago I first noticed my failing sense of smell. I could no longer smell the fragrance of wild roses on foggy evenings that I loved. Subsequently my sense of smell has deteriorated further. I have reported this to my family doctor but he made no big deal of it and suggested possibly using a steroid. My aunt of 82 years has reported to me a similar condition which makes me wonder if this is genetic or environmental. My sense of taste has suffered as well and I cannot get the same enjoyment of food as I once did.

# case 0396

I seem to be the only person nearby (within or outside my home) who notices a distinct odor of ammonia. It varies in intensity and is usually present but not always. I have had the house checked out by people with sensors and have asked others to tell me if they can detect the odor but no one but me notices. It is very disconcerting. I don't notice it away from my home. I still think it may have something to do with the house but also have tried to research to see if there is a connection to any internal health condition but so far no such information has come my way. Until now, I always have considered my ability to detect off-odors, rancidity, etc. to be good.

# case 0397

I used to be able to wear perfumes. Now I get the taste of it on my lips and down my throat and my eyes feel like I am wearing glasses that weren't prescribed for me. I also sneeze repeatedly. The worst is the weak swooning dizzy spells that come with this.

# case 0398

I first realized that my sense of smell had diminished when I could no longer smell the aroma of coffee from upstairs in the morning. Even as I stand in the kitchen now by the coffeepot I do not smell the coffee. Only ten years ago I could walk into my daughter's preschool building and smell the aroma of hazelnut coffee. I miss that wonderful aroma of coffee when I walk into a coffee shop.

# case 0399

When I was a baby I had numerous surgeries for my cleft palate. Today, I can hardly smell anything.

# case 0400

Eight years ago I had radiation treatment for breast cancer. Soon afterwards I noticed that my senses of smell and taste were distorted. Over time I completely lost these senses. My doctors said it was not from the radiation. I can taste salt and sugar and that is all. I had many colds and often used an unapproved homeopathic nasal spray, so I wonder if that created my problem. I have resigned myself to never smelling or tasting again but I keep hoping for a miracle.

# case 0401

I had heart surgery at the age of 79. One year later I bought my favorite perfume and realized that I could not smell it. I bought some other perfumes but could not smell those either. This is a dangerous situation.

# case 0402

About a year ago I began to notice that my sense of smell was lessening to such a degree that I could not smell my perfume, coffee, bacon, or anything else. At first it was just a little, but has now increased to the point that if a gas stove was emitting gas, unlit, I would probably die. Aside from the pleasure of these aforementioned smells, and the danger of dangerous odors, I am also not aware of personal body odors. My life is changing because of this minor, but possibly dangerous condition. I have mentioned the problem to my primary care doctor, and he just sort of brushed it off.

# case 0403

I've had allergies and nasal infections for many years. I've had two nasal surgeries to remove nasal polyps. I've had my sense of smell come and go over the years, but recently I haven't been able to smell at all for at least a year.

# case 0404

I had craniotomy to remove a tumor three months ago. After the surgery my sense of smell was completely gone. The neurosurgeons tell me that it is highly unlikely that it will return. As a consequence my sense of taste is limited to sweet, salty, sour and bitter and even those four are not too clear. It is certainly very sad to lose one's sense of smell.

# case 0405

In the past year I have noticed that when people complain about smells, I do not smell anything. It has to be very strong, like strong perfume, before I can smell it. I really miss being able to do this.

# case 0406

I have no sense of smell. I used nasal spray regularly for almost 30 years and I firmly believe that my loss of smell is due to the misuse of the nasal spray.

# case 0407

Last summer I had very bad hay fever and it took me two weeks to get better and during this time I started noticing that I couldn't taste what I was eating. I still had a stuffy nose even when the cold was over. When I went see my doctor he said that it does take time. However, later I was diagnosed with a chronic sinus inflammation. I still do not smell or taste things. This condition is stressful, especially when someone asks me if I can smell something bad.

# case 0408

In the last seven years, from time to time, I start thinking I can smell diesel fumes and taste them!! Most unpleasant! I used to work in the motor trade and used to believe that this was a real smell. Since I have left the motor industry, I have experienced the same thing on at least three occasions.... I have it at the moment. It started last Tuesday, when I had to sit near the back on the bus going to work and it is still with me one week later. I know now there is no smell of diesel, but I still smell it and I feel that I can taste it at the back of my throat.... Vile.

# case 0409

A few months ago I had flu-like symptoms (hot/cold temperature, runny nose, base of throat felt raw but swallowing not painful) that lasted for ten days. Afterwards I gradually realized that something is missing. I had no sense of smell anymore. My sense of taste has diminished by around 65% and is going downhill. I can sense spices, sweet/sour/salt. White wine tastes like vinegar. I can enjoy a few sips of red wine or beer but it becomes meaningless if I drink any more. I understand that there is no "cure". I have not yet visited my doctor because, over here, I don't think they know much about the problem.

# case 0410

I lost my sense of smell gradually. The problem started with sneezing in the morning. An ear nose throat doctor suggested sinus surgery but I did not agree to it.

# case 0411

I started smoking when I was 16 or 17. I woke up one day with no sense of smell. After a few weeks I consulted my pediatrician who confirmed my lack of smell. I also consulted with an ear nose throat doctor who found no underlying problem. I never pursued my loss of smell after this. I can taste. Later I was diagnosed with ankylosing spondylitis that I believe started to flare up around the same time I lost my sense of smell.

# case 0412

Two months ago I was awoken by a strong smell that I initially thought was burning of an electrical system although it did not necessarily smell like an electrical problem. Since then the sensation has persisted. It never goes away entirely but the severity does vary. It is a particularly rotten smell which when it is severe causes stomach upset and a gagging sensation. I was treated for a sinus infection after the onset of this with no discernible change in my smell symptoms. What helps me mitigate the symptoms is the chewing of strongly flavored gum and the coating of the inside of my nostril with petroleum jelly. There seems to be some feedback mechanism from the sense of taste to the sense of smell. This helps me to sleep relatively undisturbed as long as I breathe through my mouth. I did suffer a concussion due to a fall about 6 months before this incident.

# case 0413

Some years back my husband said "I can smell gas", but I couldn't. He went into the kitchen and found that I had not completely turned off the stove. It also made me think about other things I couldn't smell. I cannot smell coffee brewing, bacon cooking, and onions among many other things. I also have lost my sense of taste. I think because of that, I never actually get hungry, all food tastes the same. My doctor cannot explain it. For many years I have had sinus trouble, blocked passages, mucus draining almost every morning. I have used inhalers and nasal sprays for many years, and once or twice I have been treated for a sinus infection with antibiotics. I have just about given up now.

# case 0414

Nine years ago I suffered a very bad cold, and unlike previous times, when I lost my smell and taste, this time it didn't come back. I went to an ear nose throat specialist who checked my sinuses and conclude that the nerves had been deadened by the extreme hot temperatures caused by the cold. I later found another specialist who prescribed a treatment that did not result in any noticeable difference.

# case 0415

I don't remember ever smelling. I was born without a sense of smell. My father, aunt, other aunt, uncle, and grandmother couldn't smell either. I was the only grandchild that couldn't smell.

# case 0416

About a year ago I noticed my coffee had a strange taste. Then one day I was eating peanut butter pretzels and they smelled moldy and tasted musty. Other things that tasted like this: tuna fish, eggs, mayonnaise, roasts, turkey, sausage, popcorn, perfumes, and onions. The smell will be so strong it will wake me up at night. No underlying physical cause has been found. The smell and taste will make me gag and feel sick. I sometimes feel like I can't breathe because the smell and taste in my mouth are so strong.

# case 0417

At the age of 11, I had a subarachnoid cyst in my brain pressuring on my optic nerves. The cyst was aspirated by opening the skull. After the surgery I noticed after one year that I could not smell anything at all. I feel that it might have been the result of the surgery. I am not able to get any specific remedy for this. If I can, I shall be the happiest person.

# case 0418

Several years ago I had a bad cold and used an unapproved homeopathic nasal gel. Since that time my ability to smell things has decreased. I especially noticed this in regard to my ability to smell my cologne. My ability to smell things is not entirely gone, but it is diminished.

# case 0419

I do not know why I lost my sense of smell. I worked in a company where they painted engines for automobiles. The last smell I remember is the smell of baked paint.

# case 0420

I suffer three to four times a year from the common cold which lasts from two weeks to one month every time. During this my nose used to be stuffy. I started using decongestants four months ago and since have lost my sense of smell.

# case 0421

I was suffering from what I thought was a normal head cold and sinus infection. It ended up lasting almost six month and no treatment worked. I lost almost 40 pounds at a rate of 4 to 6 pound per week. I was weak, had no energy, and no appetite. I have permanently lost all sense of smell and taste. It caused depression for a while and I had to force myself to eat. I am trying to cope with this on a daily basis even though it has been over two years now, I will never be able to smell the roses again or taste my morning cup of coffee.

# case 0422

I smell toast. Today I was sitting at my computer and the smell was so strong that I thought it was being made right next to me. This has happened a few times in the past.

# case 0423

After having surgery to repair a deviated septum I noticed that I had a greatly enhanced sense of smell. This was particularly disturbing when I was riding on public transportation, where I was bombarded with dirty hair smell, underarm and genital body odors, strong colognes and especially bad breath and garlic odors emanating from my fellow passengers. At work the solvent smell from the copier was unbearable, and even seemed to stick to the newly printed paperwork after it was carried away from the machine. I smelled cigarette butts on the sidewalk as I walked from the bus to my office and rancid grease as I passed by restaurants that were not yet open for the day. When I mentioned this phenomenon to the surgeon he said, "That sounds like a good thing to have." Believe me, it isn't. Soap, shampoo, cleaning supplies and candles that used to smell good to me were overwhelming and cloying. The one smell that didn't bother me, surprisingly enough, was the smell of my dog. I wash him once a week and I was happy when a day or two after his bath the shampoo smell had faded and his doggy smell had returned. This situation lasted almost two years, but thank goodness my sense of smell has now returned to normal.

# case 0424

I have had a sinus infection and lost my sense of smell. I have very bad panic attacks over this.

# case 0425

I had severe bronchitis and sinus infections for a period of six months. Over that period of time I noticed that even when my nose was not blocked, I could no longer smell anything. My sense of smell has not returned.

# case 0426

About one year ago I began noticing that in the first moments when I wake up, I have a very keen sense of smell. It doesn't happen every day, but occasionally the scent of a candle four feet away strikes my nostrils a moment or two after waking up. Similar things happen with other lightly scented objects nearby. This heightened sense fades after about a minute of being awake.

# case 0427

I had a bad cold four months ago and noticed I couldn't smell when the cold was over. Then my smell came back gradually and only partially. I experienced weird smells, and still can't smell some things at all. It is selective smelling. This condition is really very annoying. Luckily I have partial smell and not all smell is gone, I guess it could be worse.

# case 0428

A year ago I suffered severe liver damage due to an infection. One week after the onset of the attack my sense of smell heightened to the point of being dizzying. It remains hyper acute. My doctors never asked about it, and when I told them they didn't seem to care much. Three hypotheses were offered by three different doctors. That's as far as it went: a mere curiosity.

# case 0429

I lost my sense of smell and taste several years ago and it drives me insane. I sometimes smell a certain smell and it stays with me for several days. Even my skin smells of it. It is depressing that all the pleasure of eating and smelling is taken away.

# case 0430

I think I inherited a smell disorder. My mother, her sister and my two sisters, we all can't smell anything...

# case 0431

I noticed a sudden and complete loss of smell five months ago. It was accompanied by a reduced ability to taste. I have type 1 diabetes and often experience acetone-like smell when blood sugar levels are absolutely normal. My enjoyment of cooking and eating — even socializing — is now greatly reduced. I have been advised by my ear nose throat doctor that the ability to smell is unlikely to return — how depressing.

# case 0432

I had a mild cold for a few days ten months ago and when that was over I noticed that I could no longer smell or taste anything. I also suffer extremely from gastroesophageal reflux disease.

# case 0433

I had a flu-like sickness and discovered afterwards that I had lost about 85% of my smell capability. An MRI showed no irregularities and the doctor said that there was nothing she could do. It may come back or not and if it comes back it could take years.

# case 0434

I recently caught a bad cold. I was sick for about two weeks and it was so bad I almost completely lost my voice. I have gotten over the cold three weeks ago but my senses of taste and smell have not returned yet.

# case 0435

I developed a sinus infection that was treated with antibiotics. Within a few days I had the most awful bitter taste in my mouth. After a few more days I lost my sense of taste and smell. Now three months later it has not come back. Even if I do still have a sinus infection, I believe I lost my smell/taste because of the treatment with antibiotics, and I don't believe it's coming back. I always had a keen sense of taste/smell, and this has just devastated me.

# case 0436

I fell and fractured my skull five months ago. Afterwards, my sense of smell and taste was gone. Three months ago I started having dreadful tastes and smells in my mouth which affect everything I eat and drink. No doctor can seem to help. It is depressing me.

# case 0437

I have a long history of nasal polyps and have had three operations over the past 15 years to remove recurrences of the polyps. After the first two operations my sense of smell would return soon after, however in the 24 months or so since my third surgery I have had no sense of smell at all. I do — very occasionally — get brief bursts of a full sense of smell. On examination it seems my sinuses are entirely unobstructed. However, with the exception of those brief bursts, I have no sense of smell. The biggest impact is on my sense of taste, and I'd dearly love to be able to get the full experience of eating.

# case 0438

I grew up and discovered that I could not perceive smell when people talked about pleasant or unpleasant odors that I couldn't smell. This condition has continued to this date.

# case 0439

I had an infection in my nose six years ago. I had completely lost my sense of smell by the next year and it has never returned. Over the past five years I've had a blocked nose and for the past few months I experienced a bad smell up my nose all the time.

# case 0440

I fell with the back of my head on the road. I didn't notice till about a week later whilst applying deodorant that I couldn't smell. A neurologist told me that my olfactory nerve was severed as a result of my brain shifting inside my skull when it hit the road. My sense of taste is surprisingly unaffected at all and food still tastes the same as always which is a positive thing.

# case 0441

I first noticed that I had a problem when I thought that my shower gel had gone moldy but very soon I began to notice that food also tasted bad or "off". Soon I realized that I am unable to sense any smells either pleasant or unpleasant. While cleaning my mother's oven using pure ammonia, I realized that it was only by the mild sensation in my eyes that I am able to sense pure ammonia. Likewise, I cannot register the smell of cat urine and can clean out very well used litter trays without smelling anything! However, when it comes to food, my world has changed beyond belief as the only foods that taste "right" to me are boiled potatoes and milk. Most vegetables and fruits taste like rotting compost, or rancid, burnt oil. I have lost half my body weight, most of my hair and all but one of my teeth. I have discovered that I can eat couscous with soured cream and sugar, chocolate chip cookies and milk. This comprises my entire diet and has done for the past year or so. I have yet to find any food that I can honestly say I enjoy. I used to keep trying old favorites, but the sense of disappointment when I find them to be completely unpalatable makes this a pointless exercise. Naturally, I am unable to appreciate the smell of summer rain, new mown grass, Christmas trees, or the ocean. Commercial perfumes and colognes can be so overpowering that they can cause me to heave — likewise the smell of barbecuing food or baking bread. I am told by my ear nose throat doctor that the condition may have been related to some virus and the prospects of my sense of smell ever returning are slim. Occasionally, I am treated to a brief whiff of apple pie but it is only a phantom and usually ends up with me hyperventilating in an effort to enjoy as much of the pleasant smell as I possibly can before it disappears again. Other than the odd phantom smell, there are no nice scents in my world. I have seriously considered (and attempted) suicide as my enjoyment of life has been so severely curtailed. I suspect that some of my healthcare professionals do not believe that my problems are real, preferring instead to ascribe some hysterical cause to my condition and I will admit that since the change I have become argumentative and angry with strangers. So few people can understand the full implications of my loss, and I am tired of trying to explain it to others.

# case 0442

Two years ago I had a serious accident. I was in a coma for three days. I woke up and was fed, but could not smell or taste. It has been that way ever since.

# case 0443

I first noticed that I lost my sense of smell about eight years ago when I walked into my home and my girlfriend told me the house reeked of natural gas. I could not smell this at all. I became concerned because it was not a gradual loss. I lost it all at once. I can taste somewhat, at least I think I can. I wish I could regain this sense someday; I really do miss my sense of smell.

# case 0444

I lost my sense of smell about a year ago when I had a cold. The cold went away but my sense of smell did not return. I went to a physician but no medication helped. I also lost some food taste.

# case 0445

One morning, all of a sudden, my smell and taste changed for the worse! I can't stand the smell/taste of onions, coffee, chocolates, pan fried meat, barbeque, some sausages, some fruits, cleaning fluid, toilet spray, even the fresh air that I breath has a vanilla smell.

# case 0446

I fell and suffered a fractured skull 13 years ago. The impact was at the back of my skull which pushed the brain forward. There was bruising to the front of my brain. I have been unable to smell or taste since.

# case 0447

Ten months ago I stayed in a hotel and all the rooms had the same sickening fabric dryer sheet smell to them. I told the manager I simply cannot stand this overwhelming smell and that I would need to stay at a different hotel. He looked at me like I had two heads, and agreed. I noticed that the other staff members were staring at me like I was a crazy man. I went to another hotel and I smelled the same thing. To make a long story short, the increased smell lasted about a month then went away. It went away until six months ago when it hit me again. This time it was accompanied by tremendous ringing in my ears, tingling in my legs and feet, pain in my right foot, and a tremendously unsteady gait. It lasted about two weeks. The symptoms hit again five months ago, then once a month. Now it is happening once a week. I now have a tremendous general malaise. I went to a neurologist and had just about every test known to man.

# case 0448

About six months ago I sensed that something was amiss with my sense of smell as I continually smelled a strange smell which seemed to come and go. It smells like a stale, musky, damp carpet. Since I have had this I am sure I have also lost my taste and often feel like I have a metallic feel in my mouth.

# case 0449

I lost my sense of smell for no apparent reason five years ago at age 72. I never noticed it until my daughter said my house had a terrible odor and we then discovered a dead rodent that caused the odor. I mentioned this to my doctor but she seemed unconcerned.

# case 0450

In the last five years my sense of smell has deteriorated. A couple of times I tried to get help from doctors. When I tell them about my problem, they look at me and smile. They have no answers or remedies. I cannot smell even strong perfumes, garlic, or ginger. Food does not smell. Sometimes, all of a sudden, I can smell some herbs like dried turmeric leaves or curry leaves. I miss the smell of food grilling, the smell of first rain fall and the smell of flowers. Now I know I have to live with it.

# case 0451

I had a cold that lasted about three weeks. I thought I was getting a sinus infection but didn't. Then I realized that I was losing my sense of smell. I have always had a good sense of smell but after the cold I would take a deep breath outside and smell a smoky smell when there was no smoke. The ear nose throat doctor says it could take six months for me to get my sense of smell back. Food also tastes off. I cooked onions in butter and it smelled bad. Cooking a pot roast in the crock pot smelled awful, smoky even though I could sort of taste it. A chocolate chip cookie tasted ashy. This past weekend the smoky smell was at its worst. It was such a strong sensation that it was as if I was sitting next to a chain smoker. Even my eyes were watering and my throat hurt. I used saline spray and the smoky smell is gone. Today my throat is still irritated. My theory is that the reason that the smoky smell is the first odor I perceive is that there is a biological, evolutionary advantage in being able to detect smoke. I can taste pickles, ketchup and milk chocolate, but that's it.

# case 0452

I noticed a change in my sense of smell. It is becoming more sensitive for stinky smells and weaker for sweet smells like flowers. Strong and unpleasant smells are more easily sensed than pleasant smells. In my childhood I could smell a rose from ten feet away but now it seems that my sense of smell has gone down.

# case 0453

I had an operation to remove my tonsils when I was 6 years old and that is when I lost my ability to smell. Since then I haven't been sure whether it was in my head or if there is something actually wrong with me. I have however, recently been able to pick up a couple of smells which are very dull to me but are very strong to the other people that smelled them. There isn't anything wrong with my sense of taste. In fact I have been able to pick up tastes quicker than other people, like when there was too much salt.

# case 0454

A few months ago I realized that I was starting to lose my sense of smell. This morning I baked a loaf of bread, and was unable to smell the bread baking. My family members commented on how awesome the smell of the bread baking, was. Some people may think this is a good thing, because I can't smell stinky things, but at the same time I cannot smell good things either!

# case 0455

I have a strong smell of smoke in my nostrils. I do not and have never smoked.

# case 0456

I began to notice that coffee, perfume, and spices do not have any effect on my senses of smell and taste. I can taste sweet, salt, sour, chili, and hot and cold temperatures. All flavors have gone, and very occasionally I can smell a cut lemon but this is not sustained. I have seen ear nose throat specialists and had a scan. No one can help and no one seems to realize just how sad it is to live without these senses. Not to enjoy good food or smell the roses is pretty tough. It can also present danger in the case of fires or gas leaks. I don't know how it will all end, but I will keep researching and looking for some advance in the knowledge and treatment of this condition.

# case 0457

I lost my sense of smell. I have had occasional treatment after which my sense of smell returned temporarily. Nasal sprays do not help. Occasionally, after a bout of cold symptoms my smell would return but this is rare.

# case 0458

I have never had a sense of smell. At a young age my mother realized that I could not smell anything and I still cannot smell anything good or bad. Growing-up was very hard, when most people can smell if they have body odor I could not, which made me ripe for being teased in school a lot. Not to mention it is dangerous because I cannot smell smoke from a fire!

# case 0459

Two months ago I broke my skull. My biggest concern is that I now have a total loss of taste and smell. I'm a huge fan of cooking, and an even bigger fan of eating, and I love red wine. I'm worried none of this will ever matter again and food will always be a memory to me now. The neurologists told me that I may regain these senses, and if they are to repair themselves, they will do so within a year.

# case 0460

Five month ago, at age 58, I started to notice that I was losing my sense of smell. I thought it was due to a cold. After a month I went to see a doctor who confirmed with a smell test that I could not smell. He checked for polyps and did an MRI. He prescribed a nasal spray that I take once a day and told me to make another appointment 6 months from now. I am desperate.

# case 0461

I smell cigarette smoke.

# case 0462

I take many medications and have a constant smell in my nostrils from my medications. It feels as if the odor is strong enough for others to smell. Sometimes I feel it also comes from my pores as body odor, or urine, but my husband says I absolutely do not smell of anything unusual.

# case 0463

During a bad cold as a child I temporarily lost my sense of smell and taste. I had further colds and allergy problems as an adult. Then at age 50 I lost all of my sense of taste and smell. A doctor recommended polyp removal which I have not done yet. The loss of my sense of smell and taste has led to obesity because I crave salt and sugar, which are the only sensations I get from eating.

# case 0464

I worked for about 2.5 years in a chemistry lab and now can no longer smell the solvents, etc. in the lab. I also cannot smell sweeter foods (like baked desserts, fruits, and breads), but I can still smell savory foods. A friend said that she had the same problem, but her sense of smell came back after she stopped working in her lab.

# case 0465

I moved to Australia from the US four months ago and had a hard time with getting used to the food. Now everything smells strange to me. It all has the same odor and taste. I found a store that sells imported American food and brought it home and it had the same strange smell and taste so now I know it is me and not the food. Even air fresheners, soap and my tooth paste which I brought with me from America have the same strange smell.

# case 0466

One year ago I lost my sense of smell almost entirely. I was changing my cat's litter box which I knew to have a very offensive odor, and I realized I did not smell it at all. I then held a bottle of cologne up to my nose and realized that I could not smell it. Over the last year I have caught a whiff of something here and there, maybe once a week. I have also experienced distorted smells a few times where there was this overwhelming smell of a dirty ashtray. I cannot distinguish flavors, but can tell sweet, salty, and bitter. I went to see an ear nose throat doctor who was convinced that once he sprayed decongestant up my nose I would be able to smell — he was wrong.

# case 0467

I have lost my sense of smell. I am a construction worker and thought that my loss of smell came from the dust and paints that I had to work around. But my brother who is two years older has the same loss of smell, so I now wonder if this may be genetic. Now I find this a real hazard as I cannot smell the gas from my stove.

# case 0468

In the past year I have noticed that my smell perception has changed. Certain things such as deodorants and soaps are now perceived differently by my sense of smell. Often the perception is that deodorants are sharper and less pleasant. Similarly, I seem to smell the sharpness of cooking oils from roasted peanuts. I still enjoy eating but somehow the smells are changing. My body odor also seems to have changed; the deodorant I use has a somewhat different odor, but not unpleasant. My wife has not noticed this and I therefore believe that it is my smell sense that has somehow changed.

# case 0469

I had surgery for a broken leg. Before the surgery the smell of my children's cockatiels' dirty water and cages didn't bother me but now I have a difficult time dealing with the smell.

# case 0470

About a year ago I started smelling smoke. I thought it may be my car. I work in a kitchen and started asking my employees if there was something burning. Nothing was burning. I was afraid to say anything to anyone for the fear that they would think I was crazy. Today, I talked to somebody who told me about her sister who has been smelling a weird smell for almost two years. Then she mentioned phantosmia. I looked it up online and found several stories about people smelling smoke just like me. I'm still not sure what the cause is, but it was nice to know that I'm not alone.

# case 0471

I never had a sense of smell. It wasn't a major issue. My parents never asked doctors to look into it and neither did I. I took an anthropology class a few years ago where I heard the word "anosmia" for the first time. I looked it up online and found out that I am not the only one who has never smelled anything.

# case 0472

Five months ago I suffered a fractured skull and ever since my smell and taste has gone completely.

# case 0473

I lost my sense of smell after a three-week cold. I have no ability to detect unpleasant odors, and limited sense of taste. I can no longer smell garlic (even though I can smell onions somewhat).

# case 0474

Two years after I lost my sense of smell polyps were discovered in my nasal cavity and surgically removed. It did not bring back my sense of smell. When I get colds, I sometimes can smell, but this is very rare.

# case 0475

I have had simple partial seizures which caused parosmia. The smells I am experiencing are like hyper-exaggerated versions of a smell. I can recognize what the smell is supposed to be, but the new smell is unbearably foul. It is triggered by chemicals (alcohol, perfumes, cleaning fluids, preservatives in food), lack of sleep, sudden fright, block in nasal passages, or allergic reactions. The foul smells happen all the time, just about every day. They became markedly worse after menopause. This morning I smelled moth balls which caused a bad seizure. For hours I kept having seizures which smelled like a grossly exaggerated moth ball smell.

# case 0476

One day I noticed that food did not taste normal. I no longer had the urge to eat the foods I liked the most. I would crave something and when I got it, the taste was not there. Later on I realized my smell was fading too. Over time I could not smell strong chemicals such as gas, paint, or odors of any kind and my family would ask me, don't you smell that, it is strong. I did not smell it. I can tell when something has sugar or salt in it but I do not taste it, I just know it's there. If you gave me a piece of steak and a piece of pork I could not tell the difference except for the texture and how they chewed. Every once in a while I get a whiff of something but still could not say what it is. I am 68 and I have no sense of smell at all. I wish I could get my sense of smell back and I talked with my doctor several times but he has not offered any treatment options.

# case 0477

I lost my sense of smell. I've had two sinus surgeries, but my smell comes back only after using prednisone, and that only lasts for about 1-2 weeks...

# case 0478

I have a lengthy history of post nasal drip, following a respiratory illness. I had a reduced sense of smell for many years and quite recently I noticed my sense of smell had almost disappeared.

# case 0479

I noticed a smell in my nostrils in my 30s. I have been to an ear nose throat specialist but to no avail. The smell is now giving me a headache and I find it very difficult to concentrate. I now feel uncomfortable to sit among people and even going to lectures has become a problem for me.

# case 0480

I fell and hit my head. I noticed that I lost my sense of smell the following day. A CT scan revealed some slight brain bleeding. This has been resolved but the sense of smell never returned. My neurologist claimed that my olfactory nerves were severed. I can smell certain types of odors. I can tell when popcorn has been microwaved; I can detect smoke and can tell if a restaurant is cooking seafood. My sense of taste is about 50% reduced. I can taste sweet and spicy foods but many spices have no taste at all.

# case 0481

A few days after being knocked unconscious I noticed I could not smell aftershave, but I just assumed that I had a sinus problem. Since then my sense of smell has been altered. It is not completely gone; however some things smell different than before. Also, as you would expect, my taste has been affected.

# case 0482

After a viral infection with fever I started smelling a funny chemical smell and I still have it today. The odor does get stronger at times.

# case 0483

For the past couple of weeks I have a distorted sense of smell. The one scent that I normally would enjoy is the smell of toast. However, now it smells like burning flesh. It makes me feel queasy and the thought of eating is difficult. Aside from the toast "trigger" it seems as though there's a general sweet-like rotten smell everywhere. I have researched this condition online and found that there seems to be a link with upper respiratory infections. I have had a dry raspy cough for about the same amount of time I've had the parosmia.

# case 0484

In the past month I have started to smell garlic in random places and on random people. The main places I notice garlic is on my toothbrush (which has been doused in alcohol, hot water, and Benzyl Peroxide several times), in eggs, on my boyfriend's breath (he swears he hasn't handled or eaten it in over a week), and in my coffee. I do have an extremely keen sense of smell, so this is driving me a bit crazy trying to figure out if I'm smelling something that normal people can't (which happens all the time), or if my brain is going haywire. I hope this isn't permanent as garlic is a fairly awful scent to be stuck with.

# case 0485

About 18 years ago I was in an accident that changed my sense of smell. Anything and everything for me just smelled of either chocolate or oranges... I had a consultation with a neurologist who said my normal sense of smell would return in time. Eventually, it did return. My sense of smell is now back to normal but not as sharp as before the accident.

# case 0486

I was diagnosed with sinusitis and I have totally lost my sense of smell as a result. No cure was suggested and I was told that my sense of smell may never return. It has not in the two years since.

# case 0487

A year ago, at age 58, I had a very bad cold and cough. I lost my sense of smell and taste. Three months later it still hadn't returned so I went to two different doctors and to the hospital. During this time I started experiencing distorted smells and tastes. I was told that nothing could be done to restore my sense of smell and taste. Now, a year later, I can taste salty, spicy, and sweet. Smell is really weird. I sometimes smell things that are not there or smell completely different from what they should smell like.

# case 0488

My nose does not know what smell is. I never cover my face for bad smells and never get excited for good smells. I feel my nose is just for breathing in and out.

# case 0489

Approximately eight years ago, when I was 44 years old, I started to notice that I couldn't taste my food. It was a gradual process. I also couldn't smell. It is the most devastating feeling in the world! Sometimes I want to just scream with frustration!! I believe that my loss of my sense of smell has something to do with my nasal polyps and asthma.

# case 0490

I had an accident nine months ago. I lost my sense of smell. It has not been restored yet.

# case 0491

My sense of smell diminished. Sometimes I can smell about half of what was normal for me, most of the time it is about twenty percent of normal.

# case 0492

I lost my sense of smell and taste due to sinus polyps. I regained it briefly after using prednisone on three occasions and after the first time I had surgery for removing polyps. After a second surgery to remove polyps seven months ago my sense of smell did not return.

# case 0493

Lately I am constantly smelling gasoline exhaust and my nose is very dry.

# case 0494

Approximately six months ago I fell and struck my head, losing consciousness for several minutes. I have lost all sense of taste and smell. Absolutely 100%! I never tied the fall and the loss of my sense of smell together until doing some research on the internet today.

# case 0495

I noticed a loss of sense of smell recently and through research on the internet have reasoned that this is possibly due to consumption of alcohol. Since reading this I have noticed that drinking just one or two glasses of wine leads to me losing my sense of smell for a few days. Five days ago I drank a glass of red wine and a glass of champagne. For four days I put perfume on my wrist and was unable to smell it at all. Then this morning my sense of smell returned and I could smell it well. I can't tell if something is burning in the oven or if I've trodden on any chicken poo, or if clothing smells and needs washing. Not being able to smell is really quite disabling.

# case 0496

I recently, at age 36, started smelling something burning, but there is nothing burning.

# case 0497

About six months ago I noticed an unusual smell that seemed to be inside of my nose. It has gotten stronger and masks other smells, like those of perfumes, cooking food, and smoke. The smell is stronger when my head is in a slightly elevated (semi-fowler) position. Any time I put my chin towards my chest the smell is intensified. Doctors examined my nose and no physical cause for the phantom smells was discovered. I cannot identify the smell. It is like nothing I have ever smelled before. It's just nasty and it is driving me crazy!

# case 0498

A year ago my wife and I both caught a bad cold. Shortly afterwards we noticed that we couldn't smell anything anymore. Some weeks later my wife regained part of her sense of smell but mine has not returned.

# case 0499

I have asthma which is controlled by medication. However, since this month I have lost my sense of smell, and occasionally my sense of taste.

# case 0500

I had a viral infection that lasted a few weeks. Afterwards I noticed that my senses of smell and taste had gone. I think they had gone before the virus but I was too distressed to notice. Since then I smell strange odors, some metallic and some smoky. I also suddenly get cravings to taste many flavors.

# case 0501

Three years ago I started smelling a persistent smell. It was with me everywhere. I finally figured out that it was a body lotion that I was wearing but the smell never quit. I quit using the lotion. Now if I smell a strong smell it stays with me for days. I have asked my husband if he smells it and the answer is always "no". It is not a phantom smell. I smell a cooking odor and the problem is that it stays with me for days. It is usually a sweet, slightly burnt smell. Like a roast that has been cooked just a little too long in the oven. It seems to be triggered by cooking smells. It drives me crazy.

# case 0502

For a decade or more I've experienced hyposmia. As a consequence I've felt a want for more flavorful food. For instance I never used salt in my cooking and as little sugar as possible, yet began over the last few years. The odd thing is that while many common everyday smells escape me, I seem to have a heightened sense of picking up chemical smells that others don't. When I walk past one of those dreadful nail salons or a new shop being outfitted or someone who's just had a cigarette outside then comes near me.... I'm thrown into nostril attacked revulsion. Perhaps this is more about chemical sensitivity, because I've used as-natural-as-possible products for many years to the point where I cannot sniff a bar of supermarket soap without the caustic soda hurting the back of my nostrils. Organic smells have to be quite bad for me to pick them up — and even then I am often not aware of them except for the odd whiff. This includes my own body odor, the compost pile in my kitchen, when the carpet in my old leaky car gets drenched and then stinks, or when a rodent carcass is hidden somewhere.

# case 0503

A year ago a number of my senses became heightened, including my sense of smell. In particular, I seemed to have an increased awareness of heating systems. My sensitivity to the fumes from wood burning stoves, gas furnaces, and oil furnaces increased immensely. I suddenly started smelling the exhaust from these systems while walking in neighborhoods I had been consistently walking in for years without ever noticing the smells. Now my sensitivity to these fumes is still heightened although I think it has slightly diminished from its height.

# case 0504

Approximately eight months ago, I noticed that I had started salting my food, which is unusual for me, and then, while preparing dinner, I noticed I could not smell dinner cooking. I then began consciously trying to smell various substances. I could smell virtually nothing. In these last months, I have only smelled three things that I can recall: horse droppings, my little grandson's feet, and onions. I still can taste food but my husband has to do the tasting when I am preparing dishes with subtle flavors.

# case 0505

I think I had a normal sense of smell as a child and young adult. At the age of 35 I became interested in cooking and realized how poor my sense of smell was. The more I got into cooking the more irritating this has become. I guard against overseasoning because I don't detect what others do. I can't smell spoiled milk or meat unless it is really gone over.

# case 0506

I injured my head and had pain for about a month. As that subsided I developed a head cold and blocked sinuses. I cannot breathe through my nose at night which is very uncomfortable. For the past month I have noticed that I cannot really smell anything, though my sense of taste is pretty okay.

# case 0507

I have a long standing cough and was recently diagnosed with bronchiectasis. My sense of smell has been fading for a long time. I now have practically lost my sense of smell completely, such that I cannot smell Vick on my handkerchief.

# case 0508

I had sinus surgery four months ago. Six days later I hemorrhaged. I have chronic rhinitis now. My smell has gradually gotten worse. I can't smell anything now. This is very depressing.

# case 0509

About two or three times a year I smell an unidentifiable smell. It started 15 years ago. The smell is always the same in nature and usually it is aggravated by other odors. It's really very unpleasant.

# case 0510

My loss of smell was gradual. I used to suffer from severe colds and I believe that this may have been among the causes. My mother also suffers from the same condition. Perhaps my condition is hereditary, though no-one else in our family has reported any loss of their sense of smell. I began to notice the loss of smell about five years ago. At that point I was unable to taste strawberries, vanilla ice cream and then mint, which are among my favorite foods. I went to the seaside two years ago, but was unable to smell the sea and I felt quite sad about this. Also, on rainy days, I am unable to smell the damp earth or freshly cut grass — things most people take for granted. The other day I went to house in which there was a gas leak. I was totally unaware of this. My loss of smell also means that I need to take extra care while cooking since I won't know if the food is burning. I used various nasal sprays, some of which worked temporarily. On a positive note, if a strongly perfumed item is placed close to my nose, I am often able to smell it. And on rare occasions, my sense of smell returns sporadically. I really appreciate and make the most of situations as such.

# case 0511

After my first pregnancy I became very sensitive to many things such as cold, allergy etc. I also noticed that I started to sense foul smells which are not noticed by others. This made me very conscious about cleanliness and I get annoyed with dirt, smell from toilets etc. Too much cleanliness affects the relations with the people I live with.

# case 0512

For the past nine years or so I have completely lost my sense of smell. It all began about 13 years ago when I used to have very frequent olfactory fatigue for very long spells. My nose also blocks very often with or without discharge. I was given drugs for curing colds by my doctor. Initially it seemed to be working but the relief is only temporary. Though I no longer have any sense of smell, once a while when I come into some very good news I can all of a sudden smell my surrounding for a brief moment but then it dies out again.

# case 0513

I cannot be around smelly activities like cooking. If I am around odors, all of a sudden I get a terrible smell coming from my left nostril. I have seen three ear nose throat doctors and had an MRI which showed enlarged blood vessels in my left nostril. The doctors say the enlarged blood vessel is not the cause of my problem, but I disagree. Now I always carry a syringe around with me just in case the smell occurs. When I irrigate my nostril, the smell is gone and my taste returns to normal.

# case 0514

I quit smoking cigars 30 days ago and since then I have completely lost my sense of smell. It is so bad that we had a gas leak in our back yard that everyone noticed but me.

# case 0515

I used to have a superb sense of smell until I was 18. Ever since then my sense of smell decreased. I noticed it first when I was around 22 and visited Hawaii for the second time but could no longer smell the flowers in the air. Nobody seems to know what is going on and my doctor says that I just have to live with it. I hate it, because smell is such a fundamental sense.

# case 0516

I lost my sense of smell after I sustained a blow to the head probably had a concussion (although I didn't go to a doctor for the head trauma). I noticed two days later while I was cooking that I couldn't smell the food. This prompted me to make a doctor appointment (one of many since then) and nobody can diagnose the problem. I can't say I've become depressed from this, as I have read many people do after losing their sense of smell, but I certainly miss it terribly. You don't know how important your sense of smell is until you've lost it.

# case 0517

I completely lost my sense of smell and never went to see a doctor as I assumed there was nothing I could do for it. Everything just smells like air! I still have my sense of taste.

# case 0518

Last year I started to have smell hallucinations though at first I thought the smells were real. I also started to suffer from a tingling in my hands and from visual disturbances. At first I thought I was having a stroke. I did some research and found that all these symptoms were associated with migraine. However, the smell is more or less constant. Sometimes it is a sweet, sickly smell, but it can also be a smell like burning rubber with overtones of kipper, and sometimes an unearthly electric/chemical type of smell. My perception of taste has also altered. The only flavors I can tolerate are cinnamon, cumin, and celery. The smell/taste problem is making me feel suicidal.

# case 0519

Four years ago I had a myomectomy which went very well. Since this surgery I have a heightened sense of smell which has slowly become worse. I am more sensitive to smells in the morning and I am particularly nauseated by human body odors. Strong body odors make me gag and want to throw up. Twenty feet away from someone I can tell if they have not washed their hair that day. Nice smells also bother me. Perfumes in detergents or on women cause me to have an allergic reaction, red itchy swollen eyes, stuffy nose which I must relieve with a couple of days of antihistamines. The condition is causing me to become socially dysfunctional and killing my appetite. I assumed it was from the surgery however I am concerned the cause is something else as it has not worn away and in fact is getting worse.

# case 0520

I first noticed that my sense of smell was not like that of many other people when I was about 13 years old. People would smell things that I couldn't, or smells that seemed good to me were offensive to other people (e.g. cat pee). I was in college before I could identify the smell of a skunk. When I was 24, I hit my head and had a concussion. Shortly after, I noticed that I could smell things easier. Sometimes while walking, I would stop and realize that there was a fruit tree nearby. I also began agreeing with people when they smelled something, sometimes being the first to notice. I assume that my sinuses opened up during the accident.

# case 0521

I was hooked on cough syrup which had codein phosphate and ephedrine in it for more than seven years. I am told that excess of ephedrine deposit in your body causes hypothyroidism, which in turn gives rise to anosmia.

# case 0522

I have had allergy symptoms for about a year now. In the last few weeks I cannot smell at all, even when my nose is not blocked. I am very concerned with this aspect especially after reading possible causes.

# case 0523

My sense of smell comes and goes. For three years I couldn't smell anything and felt depressed. I started drinking dragon fruit-flavored sugar water and noticed it really awakened my sense of smell. After I drink a bottle of it I start smelling everything. It really makes me happy to be able to enjoy smelling more often. I haven't recovered my whole sense of smell but it's coming and going again which is fine as long as I get some smell in occasionally.

# case 0524

Within a year after moving house I noticed a sharp decline in my ability to smell. I had not been sick or suffered any head injury. The entire process seemed to take only a few weeks. I went from having an acute sense of smell to nothing. Physical reasons like a brain tumor or polyps have been ruled out by my doctor. The best they can come up with is possibly old age.

# case 0525

I accidentally lost my sense of smell when I used bleach to clean. It took a year for things to become normal and for food to taste normal, but it was nothing compared to the bad smell I'm experiencing currently. For the last month the smell of moldy, rotten citrus fruit has been getting stronger and stronger. I thought it was something I smelled over a year ago that was very faint, from a new computer graphics card. Now, I am not so sure the computer graphics card ever really had a smell, but at the time it seemed so. Just before this happened, I had two sinus infections almost back to back, with a cold sore that invaded my nose instead of on my lip, and the stomach flu. It's getting hard to concentrate on things and to try not to think of the smell. I could close my nose on one side and have relief but only temporarily. Even when I go somewhere without the smell, my mouth tastes like I have had metal coins in it all day. Some foods still taste the same but the group that does is dwindling. I know I have to eat, but I do not know if I will be able to stomach it. This is often overpowering and makes me nauseous. I can see why people say that they are going crazy by it. I cannot imagine how some people have made it for years with this condition.

# case 0526

I was eleven years old before I realized that I did not have a sense of smell. Throughout the years I have been able to smell mint or strong chemicals but none of the beautiful scents of flowers or perfumes or food and drink. My sense of taste has not been affected, although I find I have to salt my food to enhance the flavor.

# case 0527

I have had sinus infections since I was a teenager and had surgery to clear out sinus cavities and fix my deviated septum. A week after the surgery I could not smell or taste anything. The doctor said he had no explanation for this because it was done using the balloon method and should not have disturbed the sense of smell. Well, I have not been able to smell since and can't figure out why.

# case 0528

Over a year ago I was in an accident in which I suffered severe head and brain trauma. As a result, I've been told, my olfactory nerve sheared, leaving me with no sense of smell. I've been told I have a 50/50 chance at getting my smell back permanently. I still have my sense of taste. Also, sometimes for a couple of days I have the feeling of a certain smell which may have once existed or not. It doesn't seem real and won't go away to the point that it becomes annoying.

# case 0529

I contracted a severe infection the effects of which lasted for over a month. It was one of the worst bouts of cold and/or influenza I ever had. Since then, my sense of smell has all but deserted me. Even the most pungent smelling products such as perfume, after shave lotions, petrol, or whisky do not register at all. Strangely enough though, there is one substance that I can smell very slightly, but I have to breathe it in vigorously to experience any effect. It's Heinz Original Sandwich Spread, "The tangy crunchy spread".

# case 0530

I started to notice the diminishment of my sense of smell about 25 years ago. 13 years ago my sense of smell was gone. I have no sense of food flavor or the smell of flowers. I had a small fire in my home and slept through it. I have gas heat and I'm scared to death when I have reason to suspect a problem. My gas company is very understanding but no one actually understands what the loss of the sense means, in a day by day way. I eat what I used to like and I cook with the same ingredients, spices and other taste factors. Why? I have no idea, every meal is a disappointment.

# case 0531

I have never been able to smell anything. Growing up, I used to forget to put on deodorant. However, once I got word that I smelled from other people, I became very conscientious about using deodorant (even though I get no benefit from it myself). I have been in two house fires, in neither of which I could even smell the smoke! Last year, I was cleaning a bathroom with a strong solvent. I started coughing but continued cleaning anyway. I ended up coughing up blood, because I didn't realize how strong the scent was. I was adopted at birth. When I first met my birth mother, she told me that her mother and sister were also anosmic. I felt connected!!!

# case 0532

Around the age of eight or nine I started to notice my sense of smell was diminishing. I felt embarrassed about not being able to smell anything. I would have never admitted this to my school friends or family. If someone reacted to a smell, I'd copy their reaction to hide the fact I couldn't smell it myself. In later years I told my parents. I did sustain a hairline fracture to my cheekbone around this same age and often wonder if this incident has anything to do with my loss of sense. Generally I don't notice not being able to smell. However, I do get paranoid about personal hygiene, how my house smells — i.e. something gone rotten in the fridge, musty smells — this has resulted in me being absolutely fanatical about both personal hygiene and household cleaning — to the point where people close to me have asked if I suffer from obsessive-compulsive disorder. Also, I passed out once when I was on my own and was inhaling fumes and didn't know about because I couldn't smell them.

# case 0533

Ten years ago I had gastric bypass surgery. Sometime after that I noticed I had lost my sense of smell. To this day I still have no sense of smell, although I can go for a period of several days where my mind seems to believe I smell something, e.g. oranges, but it is only the one smell and there is nothing around that smells like it. Along with the lack of smell, I have a lack of taste. I can only taste foods that are very flavorful/seasoned. I cannot taste bland foods at all.

# case 0534

I have chronic nasal congestion and used a decongestant spray for a long period. Now I have a constant sweet smell in my nostrils and a very bad altered sense of smell and reduced taste ability.

# case 0535

About a year ago I noticed a strange smell in the house: I thought it was due to the cavity wall insulation which had just been done. My husband did not notice anything. Then I found that I noticed the same smell in another house and also in my car, so I deduced that there must be something wrong with my nose.

# case 0536

I have been anosmic since birth.

# case 0537

About 10 years back I had a cold and was stuffed up real bad. I used nasal spray that burned but worked. A year or two later I noticed that I couldn't smell the smells my wife was commenting on. Now I can only faintly smell a few things like baking bread, boiling vinegar, or acetone (if I stick my nose in the can). I sometimes get odd whiffs but I can't identify them.

# case 0538

I lost my sense of smell after a sinus infection. I remember smelling things five years ago and then four years ago I had lost any ability to smell. This is depressing. Food tastes different now and I don't enjoy food like I used to. I also miss smelling my kids and perfume.

# case 0539

We were testing different chemicals in school and while most people recoiled from the vinegar used for the acidic properties, I couldn't smell it and could not distinguish it from water. When I told my mother this, she was slightly concerned, but passed it off as congestion. More recently I was watching TV, and my dog pooped right behind my chair, and I didn't notice until my mother came down and yelled at me for not picking it up. I can smell smoke because it burns, but not much else.

# case 0540

Within the last six months I have noticed a phantom smoky smell. It can occur any time of the day and in any location. It is not debilitating but it is annoying.

# case 0541

For some time my nose was running. A short time after it stopped I developed what I can only describe as an overdeveloped sense of smell. This has continued for the last year or two. This continued enhancement of smell is having a detrimental effect on my every moment. The condition gives me intermittent headaches, nausea and sleep-less nights. At first I blamed the computer for the strong smell I was experiencing, then the washing powder my clothes were washed in, books, newspapers, the inside of the car. Wherever I go this overdeveloped sense of smell mars my day. I have yet to find an answer.

# case 0542

I realized at the age of 15 that I nothing had a strong smell to me. Subtle odors I could not smell at all. Strangely I couldn't really remember if this was a long-term ongoing thing or not. I do have vague memories of smells a long time ago. This leads me to think it must be a degenerating process as I now can't smell much of anything except for very strong smells (perfume from the open bottle is faint). I now mostly rely on "tasting" the air for any hints of smell.

# case 0543

I got shingles four years ago. I had them on half of my head and face. I still have pain to this day. My eye was affected but it did not do any real damage to it. About a year ago I realized I couldn't smell what I was cooking.

# case 0544

I first noticed a change in my sense of smell when I was pregnant. During pregnancy I found the smell of my husband very off-putting. However, when my daughter was born I was able smell her baby smell very strongly, and found this very pleasant and attractive, so I had not lost my sense of smell at this stage. I recall the change I noticed in my sense of smell as being quite sudden. After a long bus journey I got off the bus and could smell a very strong, unpleasant "earthy" smell, which stayed with me for several weeks. From that time on I have had a much reduced sense of smell and taste, and sometimes can smell a "chemical" smell, or a "vegetal" smell, which other people can't smell. I feel that my loss of the sense of smell has affected my feelings of closeness to other people — I cannot smell them, which really affects my reaction to them. I can smell certain strong smells like onions, coffee and garlic, but more subtle smells are lost and I find this very depressing. It may also explain why I have not been attracted to any men since I lost my sense of smell.

# case 0545

Just recently, at the age of 60, I started smelling smoke when nothing is burning. It even makes my eyes sting and I keep clearing my throat. It just started again this week. The last time it happened was about a month and half ago. I have asked people around me if they smell anything but they don't.

# case 0546

I had a terrible bronchitis five months ago. I took antibiotics as well as many other medications. I noticed after taking the antibiotic that my mouth was very sour. Ever since then I can't smell nor taste anything. I have been to doctors but they told me they can't do anything about it. This has been bothering me very very much. I will never know if I burn anything or even if the house goes on fire as well as if the food is bitter or salty.

# case 0547

When I was pregnant with all my children I was extra sensitive to smell. After the birth of my child two years ago my sense of smell is much much more strong/developed than it ever was before. I remember smelling things when I was pregnant with her that I had never smelled before — and eating food I had never liked before, I assume because I could smell it differently. I don't smell as well now as I did while I was pregnant with her but I do smell a lot more than I did before.

# case 0548

I had a cold. I noticed that this cold was different from others that I had before. It was dry. I was having a painful cough and it felt very dry to breathe. I was very congested and it took me two weeks to feel better. I noticed right after I was able to breathe through my nose again that I was not smelling anything. At this point, I am very concerned and worried about not recovering my sense of smell! I want to recover it! I feel anxious and sad because I don't enjoy food anymore and I like to cook, but I can't tell if the food has the right or desired taste. I don't care anymore about eating, but I know I have to eat when I am hungry.

# case 0549

I am slowly losing the ability to smell. When others complained about foul smells while standing near, say, a public toilet, I felt that there is something missing because I didn't feel anything. Slowly I realized that I couldn't feel foul smells. At first, I could still smell good smells, but gradually that too was fading. After two years I lost all my sense of smell. The problem with that is that I cannot have full taste of any food item except sweet, sour, bitter, and salty. I cannot smell flavor. I cannot distinguish ice cream flavors or juices and I can't tell tea from coffee.

# case 0550

I've simply never had a sense of smell. I only really realized this around age 12 or so, when I actually stopped to try and recall ever having smelled anything. After my mother passed her entire spice collection under my nose without the slightest reaction from me, it became clear to us that I not only had limited ability to perceive odors, but none at all. This has never changed throughout my life, and while it has been convenient when driving past ranches, cleaning toilets, and remaining blissfully unaware of someone's impolite public flatulence, it has been occasionally frustrating in preventing me from detecting smoke, my own cologne, or even recognizing whether or not I stink. Also, the many descriptions of the scents of food, perfumes, incense, and natural environments make me lament over the loss of a sense I never even had. Something odd that I have noticed: I am sensitive to the presence of ammonia under my nose. I have been told that this is not really a scent, but more of a chemical reaction in the eyes.

# case 0551

I had a bad accident seven years ago in which I hit the back of my head hard. I was in the hospital for one month and after that I found out that I couldn't taste anything. All food and all drinks taste the same. I can't smell and that's really fucked up. I am only eating because I'm hungry, I don't enjoy any food.

# case 0552

Sometimes I get the smell of onions and garlic in my nose and I cannot get rid of it for maybe a day or so.

# case 0553

I have lost my sense of smell due to head trauma, which also caused me recurring seizures.

# case 0554

Since my pregnancy my sense of smell is getting more and more precise and elevated. My husband calls me "super sniffer"! The things I smell are powerful and almost always overwhelming. Things my husband cannot even smell. The smell of cow manure tells me when we are due for a storm. My own breath can distract me. The breath of someone else is usually repulsive. The smell of microwave popcorn makes me want to leave the house and never return. God forbid anyone dares to cook salmon in my house.

# case 0555

I notice some kind of smell in the mornings and evenings or when it is hot.

# case 0556

Two or three years ago I had surgery to remove excessive flesh in my right nostril. About four months back I realized that I lost my sense of smell.

# case 0557

I gradually became aware that I couldn't smell odors as well as I once did. About 30 years later I developed Parkinson's Disease. Apparently, a loss in smell is one of the early symptoms of this disease.

# case 0558

I was born with a very good sense of smell, but seven years ago, as I remember very distinctly, I lost my ability to smell anything at all. I think that this was due to a severe head injury that I sustained after being knocked unconscious for a few minutes. I did not notice anything strange until a few days later, when I realized that I couldn't smell my favorite lotion at all. I have since learned to live with it. My friends and family members are worried about this more than I am. Most of the time, I'm really glad I can't smell things like body odor, skunks and the like. In fact, I have noticed that my gag reflex has been greatly reduced, and for that I am grateful.

# case 0559

About six months ago I lost my sense of smell. I cannot remember how it happened, all of a sudden it was gone. I wish I had it back.

# case 0560

I noticed impaired taste first. One evening I ate prime rib and it had little or no taste. I commented on this to my dinner companions, and they all agreed that their meat tasted wonderful! Someone then tasted mine and said mine was also delicious! That's when I first became aware of developing ageusia and accompanying anosmia. It has gotten much worse over the years to the point where I have no sense of smell at all. It can be very dangerous, especially when smell is nature's way of alerting us to things that could harm us, such as leaking gas. It's the ageusia that is most annoying. Instead of becoming disinterested in food, I find myself eating very spicy things, or sweet or sour, all in the interest of just having a sensation. But nothing much gets through except texture. I keep searching, and have even experienced weight gain since I'm ever looking for something... just some taste that will wake me up again.

# case 0561

After an accident I lost my entire sense of smell. I am currently recovering. When my nose is very close to an odor source I can now sometimes smell something.

# case 0562

I had a very bad sinus infection. It took some time for me to completely recover and I began to notice that I did not smell things other people did. As years passed it became a family joke that I could handle the "bad smelling" situations because I did not smell a thing! On rare occasions I have smelled things in the air (or I thought I did) and sometimes I have thought that I smelled something that just was not there. I also developed a cough about the same time — maybe just before.

# case 0563

For the last two months I have noticed a change in my sense of smell. Smells that used to be offensive are now more sweet smelling. Coffee now smells different and has also some sweet smell to it. I burnt peat and it had the same smell.

# case 0564

I lost my sense of smell and taste approximately four years ago. Both my brothers have also lost their sense of taste and smell. We would love to get it back. We have all been to different doctors but nothing seems to help. We all have had polyps removed as well as sinus surgery.

# case 0565

I noticed that my sense of smell had gone shortly after a chest infection. I thought nothing could be done, so I ignored it until a few weeks later I noticed an awful taste in my mouth. Several months later still have no sense of smell and an awful taste in my mouth.

# case 0566

I used to be able to tell the difference between smells. But for some reason my sense of smell is declining, which is not good. I do not know if it has to do with the fact that I have developed asthma and have minor sinus issues which I cannot seem to find a remedy for.

# case 0567

I have a history of sinus problems and had sinus surgery about 15 years ago. After a bout of flu and upper respiratory issues, my ability to smell and taste changed. I always smell the same odor. It is sometimes mild and sometimes very pungent. It may be a small disability but can be quite depressing.

# case 0568

Since a particularly bad flu a year and a half ago my sense of smell has been bizarre. Anything from the onion family, including leeks and garlic, smells very acrid, almost like burnt rubber. This can include wine and some bitter beers. The smell was constant for a long period and I initially thought it had something to do with a food trap in my teeth but this is not the case.

# case 0569

Five years ago I cleaned the porch with bleach solution. I could smell the bleach the entire time, but I thought it would do no harm. After several hours, I noticed that I could no longer smell the bleach. I stopped work immediately, but the damage was done. I could smell almost nothing at first, and then it improved slightly over time. Today I can smell and taste only slightly. When I taste something strong, I can taste it well the first bite, but then the taste goes away on the second bite, and I taste nothing. This anosmia has left me depressed and sad.

# case 0570

My sense of smell changed after turbinate functional endoscopic sinus surgery.

# case 0571

About three years ago I started suffering from a constantly blocked and runny nose. Then I lost my sense of smell. I don't feel I'm getting enough help as my doctor seems uninterested when I mention my lack of smell.

# case 0572

I am suffering from anosmia. Fourteen months ago I began to think I smelled smoke when I woke up each morning; I thought that the neighbors were using their fireplace. About a year ago I discovered that I could not smell anything. I had not had any head injuries, sicknesses or even a cold during that time. The only incident that I can remember that might have something to do with my loss of the sense of smell is that I inhaled laundry soap dust a few months before the phantosmia started. It burnt my nose as I breathed it in and it was very unpleasant. I miss the scent of lilacs (my favorite), the taste of food, and the savoring of a good wine.

# case 0573

In the last two months I have become sensitive to smells. At first it wasn't so bad just a few odors that were making me feel sick to my stomach. Now the sensitivity has increased to the point that I feel like I am going crazy. I can't find any relief or go any place to escape from them. I have given up coffee which I have always loved the smell of but it makes me sick now. I also cannot stand to get close to my pets. I am miserable.

# case 0574

I've been having a smell of cigarettes for about a month and a half. The first week was strong and all the time, now it is on and off. I feel that my heart races when I have the smell. I feel unbalanced and like I can't breathe. I was diagnosed with epilepsy about seven years ago. I get really frustrated when I get the smell and I don't know what to do. I think all of this has something to do with epilepsy but my neurologist doesn't think it is related.

# case 0575

I always experience a burning smell or cigar smell in the afternoon, evening, and at night for at least 10 minutes.

# case 0576

I lost my sense of smell after a bout of suspected pneumonia and antibiotics treatment ten months ago. My doctor prescribed ten days of steroid treatment for the anosmia, but there was no change. There are no physical abnormalities. It is depressing and what I really dislike is the "false" smell of things, especially shampoos, soaps, bath oils that now all have a simply awful chemical odor. My sense of taste is not too badly affected but now I need more pepper, salt etc. for the taste to register. I never realized how important smell was till I lost the ability to sense it.

# case 0577

About a month ago I had a cold and had congestion for more than ten days. I thought I couldn't smell because of the congestion. But now, after the congestion is over, I still don't smell at all. I feel like I smell someone is smoking or something burning all the time. I have a hint of smell such as cleaning agent, but not all the time. Since I don't smell, I don't taste food well either.

# case 0578

I have been suffering from sinus infections and nasal allergies ever since I can remember. I believe that I have been through several periods of loss of sense of smell and recovery over the years. I don't know whether the stress of 9/11 (I worked in the area), or the inhalation of fumes were factors, but around that time I developed what seems to be permanent anosmia.

# case 0579

I was spraying some insecticide in my house and also put down some moth balls. The odor was very strong and since then my taste and smell have changed. Now I can't smell anything except gasoline or smoke but they don't smell the same as I remember them both smelling, the gasoline smells like old gas that was left in a lawn mower for a long time and smoke smells like something I have never smelled before. I can't explain that smell. So what do I need to do or what can I try to get two of my senses back on track?

# case 0580

My mother had measles while she was pregnant with me. As I was growing up I noticed weird things like not being able to smell things that others could. And I'm completely deaf in my right ear and hard of hearing in my left ear. It gets so old when people ask me to smell something and I have to try to explain that I can't and they think I'm a freak. Not being able to smell has disadvantages. I can't smell smoke if the house is on fire, can't smell foods, chemicals, my wife's perfume or hair, rain, fresh cut grass, or engine trouble. On the other side I don't have to smell farts and I can be around things or do things that would make others sick or vomit.

# case 0581

Several months ago I realized that I had completely lost my sense of smell. I have absolutely no idea why. The only possible explanation could be the blow to my head as a result of a fall last summer.

# case 0582

I have chronic nasal infections which are under control now with a surgery and medication. My sense of smell is gone for some years now. However, once in a while when taking antibiotics plus prednisone the smell came back. My ear nose throat doctor refuses to prescribe, understandably, prednisone just to get back the sense of smell for a while. But the loss of smell is annoying. I wished there was a cure. It's not life threatening, but imagine the house on fire... The smell of smoke will not alarm me.

# case 0583

I do not have the sense of smell. It probably is hereditary because my mom lacks the sense of smell as well.

# case 0584

I have suffered from bouts of sinusitis since I was a teenager. Whenever I had a cold it would be several weeks before my sinuses cleared and I have used different nasal sprays, probably to excess. My sense of smell always used to return when the sinusitis settled down but for the past two years I find that I can no longer smell anything other than the strongest odors.

# case 0585

Several weeks after a head cold had gone I noticed I hadn't regained my sense of smell. It has now been almost a year and my sense of smell has not returned at all. I often wake during the night unable to breathe through my nose. It is very unpleasant and seems to be getting slowly worse.

# case 0586

Three years ago I gradually lost my sense of smell after an allergy attack. Most of the time my nose is dry. Sometimes, when the weather is cold, or snowy, or after hard exercise, I can smell.

# case 0587

At one point I developed many nasal issues. I then started suffering from asthma and I had steroid pumps given to me. I then discovered that I had nasal polyps. After surgery to remove them, my sense of smell completely disappeared. I think the surgery damaged my nose in some way. I so miss my sense of smell and it has affected my taste also; things just do not taste as sharp! My mother makes this beautiful lemon cake and I used to be able to taste the zest but that no longer happens. I also miss the smell of fresh cut lawn, coffee, bacon, and fresh bread. I did have a spray once from one doctor and it did improve my sense of smell but he told me I could not use it for prolonged periods.

# case 0588

About two months ago I noticed a peculiar change in my sense of smell. I had always had a good sense of smell — not hyperactive but certainly adequate to enjoy the pleasant odors and sensitive enough to protect me from the bad odors like leaking gas. I always enjoyed the aroma of cooking food, fresh flowers, good perfume and could distinguish the difference between them. I was occasionally a little sensitive — like walking down the aisle of boxed laundry detergents in the grocery store, or restaurants filled with smokers — but nothing extraordinary. The change I noticed was that there was an unusual and pervasive smell to just about everything which was similar to old deep fryer oil. The smell could even be nauseating at time. It made fried foods seem repulsive and I cannot eat it anymore. But even without food around there is still this pervasive odor. I wake up to it — and go to sleep with it — and I hate it. It is destroying my joy of life and I really just want it gone. I have tried different approaches to dealing with this condition. [1] Thinking it might be sinus related (I have dealt with many sinus infections), I cleaned my sinuses out daily using a neti pot then applying an olive leaf spray (antibiotic, antiviral and antifungal). Weeks of this did not seem to help much. [2] I avoided all chemicals as much as possible (sprays, liquids, etc.) but with little effect. [3] I then started washing my nose out with a botanical soap and water using cotton swabs multiple times a day. This had the best results — but it was always temporary and the smell always returned. [4] I have taken to stuffing my nose with sterile cotton to block all odors. This has been the most effective in blocking the odor — but not very pleasant. It is like having a constant cold. It is my theory that the strong odor of moth balls that I was exposed to for six weeks has caused this condition. Now many formerly pleasant odors (cookies in the oven) smell like "old frying oil". Fried foods smell even worse and often I experience the "old frying oil" smell in the absence of a stimulus.

# case 0589

I was born with anosmia, but I actually first really noticed that I don't have a sense of smell, when I was about 13 years old. Of course I knew that I didn't feel what the others felt when they smelled at something but I didn't realize I just didn't have a sense of smell... When I tell people about it they always think I'm special because of my condition but they also keep forgetting about it and asking me how I like a perfume or so. It's a little uncomfortable not to know how I smell. But I have friends who tell me if my feet stink and stuff. I'm also really curious about how it feels to smell at something. I've tried asking friends but they always just tell me: "Well, I don't know... a tomato smells like a tomato..." That's annoying.

# case 0590

Two months ago I seem to have caught some sort of low grade virus. I don't feel unwell but have completely lost my sense of smell! I went to an ear nose throat doctor who has found nothing.

# case 0591

One day I realized that I couldn't smell the cooking pot that hade run dry and was burning a vegetable. I then began thinking about being able to smell or not smell lots of things. I think I was around 50 years old when I became aware of not being able to smell. It may have gone back to earlier days but apparently I was not aware of the loss of smell.

# case 0592

Recently, I have been "smelling" the odor of scorched food... like chili that has burnt to the bottom of the pan or a coffee pot that's been on the burner all day. It doesn't matter where I am, in my car, at the gym, in my office or at home. Even when nothing is cooking, the smell is there. The first day I had a really bad headache after it started, but it wasn't exactly a migraine (I've had them before so I know the difference). I looked it up on the internet and it was some comfort to find out that I'm not the only one who has phantom smells.

# case 0593

I used a local herb as a nasal drop. After using it I have completely lost my sense of smell.

# case 0594

I lost my sense of smell completely about 16 years ago. I have seen an ear nose throat doctor but they found nothing wrong. My sense of taste is also not as good as it should be. I have basically been told to accept it.

# case 0595

Four days ago I used wood stain on a piece of furniture. On the next day and ever since then I am experiencing the wood stain smell no matter where I am. I even have a mild headache such as you might get from being exposed to the actual wood stain. A few days before that I had a methacholine challenge to test for asthma and I wonder if this could have something to do with my condition.

# case 0596

Apparently my paternal grandfather and maternal grandmother had a very limited or no sense of smell. My sense of smell is incredibly limited, I can discern certain scents I "like" but I cannot name them. I have no strong recognition of the smell of feces, urine, or vomit. My husband also has a very limited or no sense of smell and is also color blind. Unfortunately, our son also seems to have inherited this lack of smell.

# case 0597

I can't remember ever being able to smell. I have inquired about it a few times with my doctor but was told that nothing can be done.

# case 0598

Five months ago I had a very high fever for a couple of days. There were no other symptoms but I was slightly delirious for about 24 hours. The next time I ate something it had absolutely no taste at all. Over the last six months, I sometimes taste and smell odd things but mostly my taste and smell have given up. Coffee and wine are disgusting; I am able to taste fresh produce better than most things, but not oranges. Meat and fish are tasteless but I can really enjoy smoked salmon and smoked mackerel. I can also taste pears really well. Bread, cakes, biscuits are tasteless. Onions, chili, and curry are impossible to detect. I think things have improved slightly as I smelled someone's cigarette smoke outside in the street recently. What is quite sad is that my doctor just said she had never heard of such a condition except in brain tumor patients.

# case 0599

I lost my sense of smell after I fell down and banged my head against the stairs.

# case 0600

I started smelling a burnt smell about a couple of months ago. It was a couple of weeks before I got a severe cold. At first, I thought it was other things like my computer, my daughter smoking cigarettes, but then I realized it was me. I still have it and it's very disturbing. When I hold something up to my nose the perception changes.

# case 0601

I had a bad cold six months ago. I lost my sense of taste and smell during this period. This seemed normal to me, however, after a few weeks these senses did not return. I must mention that I can taste sweet, sour and salt tastes and very occasionally can detect that a smell is present but cannot identify it, i.e. more often than not it smells repugnant or like scorched plastic. I visited my doctor four months ago but does not hold out much hope. I am upset by this news as it is affecting my enjoyment of life.

# case 0602

I got knocked out at age 20. I knew I lost my sense of smell two days after the accident. I could still taste but I could not distinguish flavors. My brain was not getting stimulated by smelling. There were no little bursts of feel good chemicals when smelling something nice. 23 years later my smell is still completely gone. I wish I had it back. It has a huge impact on my personality. I do not bother to make a big deal of this as it will not achieve anything.

# case 0603

I am smelling blood everywhere.

# case 0604

It was last summer for about one or two months when my sense of smell increased. I'm not sure what caused this change. At the time I was even worried that I felt like I could smell everything around me and was quite consumed with all these smells. I was quite pleased with this new way of perceiving the world. Also it wasn't just one smell, it was everything. I also don't believe it was phantom smelling or smell hallucinations. I usually could smell something first then find its origin or vice versa. Eventually it just went away and the only way I could justify this in my mind was that I was extremely stressed at the time. All in all it was a mind boggling experience.

# case 0605

Three months ago I started burping uncontrollably and experienced a strange metallic taste after meals. Two days later I came down with flu-like symptoms. I noticed that things tasted and smelled strange. Chocking it up to being sick and not eating much I thought these symptoms would pass. To my astonishment, however, my sense of smell is still damaged. Everything seems distorted: food, deodorant, car fumes...nothing is quite right. My sense of taste seems somewhat improved but still not quite there. The distortion in smell is really driving me bonkers. I am trying to stay positive and patient.

# case 0606

Four months ago I had an upper respiratory infection. The symptoms were severe and it lasted about two weeks. After the illness I had no sense of smell. I thought that perhaps it was something very temporary but finally went to see a doctor two months ago. He said there was no treatment that he could offer and suggested that I wait for a few months. My sense of smell has returned but it is not right. From the beginning I could smell cinnamon and cigarette smoke — the doctor told me that those nerve pathways had not been damaged. I have some very peculiar things that happen — for example, coffee, meat, tuna, salmon and excrement all smell the same — not necessarily bad but the same. It is a scent that I cannot identify. I can no longer wear perfume because the scents are unpleasant — one popular perfume smells something like liverwurst (and I haven't smelled that in probably 30 years). Hairspray smells like sulfur, many scented products smell the same — almost like rotten eggs. So far I haven't lost any weight, but I am pretty habitual in what I eat. I can taste sweet, sour, bitter and salty but none of the nuances. I had dinner with friends several weeks ago at a restaurant I like but the food was terrible and I couldn't finish it. All I could taste was the sensation of heat from pepper. I cannot smell burnt popcorn or burnt food, but I can smell wood smoke if I am very close to it. Sometimes I am not certain if I am really smelling something or if it is just a memory. For example, if I walk past a restaurant I can't smell anything but if I glance at a grill I may get a flash scent of grilled chicken — whether or not chicken is on the grill. At first I felt very out of touch with myself — like I was out of step or like I had constantly forgotten something important. I am more relaxed now and find the process and differences in my sense of smell very interesting. I have read that some people can get depressed but I don't feel a great sense of loss — if one has to lose a sense my choice would be smell. Still I am hopeful that I will regain my sense of smell and am encouraged that many people eventually do regain it.

# case 0607

I suffered a depressed skull to the back of my head at age 17. I noticed that I couldn't smell about a year later when others were complaining about foul odors that I could not detect.

# case 0608

I suffer from congenital anosmia — I've been unable to smell since I was born. I didn't really realize it earlier on, though. It only hit me when one day in school, it was lunchtime, yet none of my classmates were going out to the hallway to fetch their lunchboxes. Of course, I only became aware of this when I came back into the classroom with my food, and realized that everyone was staring at me. Apparently, there had been construction going on these past few days, and it must've caused a leak in the bathroom pipelines down the hallway or something, resulting in a horrible stench that filled the entire corridor. I had no idea of what I was doing until everyone explained it to me.

# case 0609

For the last weeks I have been sensing an unusual acrid odor. It varies in intensity and it is persistent. It just won't go away.

# case 0610

A bit over three years ago I started smelling car exhaust frequently. It became overwhelming at times. I would get nauseous. I was so distracted by the smell I could barely pay attention to my job. I checked all my smoke alarms and carbon monoxide detectors multiple times. I reported the smells to the maintenance department at work. They checked all vents, air conditioning units etc. and nothing was found. Finally a friend went with me for hours and every time I smelled car exhaust, she smelled nothing. This lasted for ten months. Then the odor changed to a very strong sweet burnt yeasty smell that was also nauseating and lasted for seven months. The odor was not constant but extremely frequent; it may have been once every five minutes or so but not in a pattern. I felt it was more a misperception of smell than fantasy smell. For example when my children smelled cow manure I smelled a very strong smell of sweet burnt yeast. But many other odors would cause me to smell yeast also. Some odors I could smell behind the yeasty smell. After I got a cold I took zinc lozenges and the smell of yeast intensified enough that I wanted to pass out. I read an article about zinc causing smell disorders so I stopped taking zinc. Then it seemed to improve. It was going well until two months ago. Suddenly I began smelling intermittent sweet burning yeast again. It is not as frequent as previously maybe only 20-30 times a day with each lasting 10-30 sec. Again I think it is misperception of smell since if I cover my nose, the smell is lessened.

# case 0611

I have congenital anosmia. As a young child, of course I did not realize that anything was different about me until a few years into grade school. Classmates would make comments about pungent odors and I would play along, acting like I knew what they were talking about. It wasn't until later that my parents realized that I really could not smell anything at all. I've never been officially diagnosed. To this day, I have never been able to smell a thing. It doesn't bother me as much as you think it should.

# case 0612

I started smelling cigarette smoke about a week ago. It has become progressively stronger and is now constant. I am a non-smoker and live in a non-smoking home and work place.

# case 0613

I lost my sense of smell when I was 47 years old. Doctors could not treat me and they said I will not get the smelling sense back. However, whenever I am sick and doctors prescribe antibiotics I get my sense of smell back. It only lasts for a few days. After getting five injections of vitamin B12 the sense of smell also came back. It also came back after I took a medicine against a dry cough.

# case 0614

After working in an air-conditioned office for a year with one cold after another, I lost my sense of smell and most of my taste. I was diagnosed with viral anosmia.

# case 0615

Eight months ago I fell on my head. I had a massive headache for two days. When I cooked scrambled eggs I noticed that I could not smell the onions and garlic sautéing in olive oil. I love to cook and immediately noticed my sense of smell was almost totally (maybe 95%) gone. To this day my sense of smell has not come back. Food tastes differently. The flavors are not as strong as they used to be; most flavors taste watered down. Food has lost its richness.

# case 0616

Twice in the past three years I have had an episode of acute smell sensations that made me feel like I am going to faint. The odor is so strong that it takes my breath away for several seconds. The first incident occurred while I was being treated with antibiotics for a spider bite. I am currently experiencing a similar episode. Yesterday I bought a room deodorizer that you plug into an electric outlet. I woke up in the middle of the night feeling ill. All day today I have had acute smell sensations. It is a horrible feeling because you do not know when you are going to feel the sensation.

# case 0617

A couple of weeks ago I started smelling a burning odor anywhere I went. It can be very annoying.

# case 0618

Following knee surgery I began having a horrible smell which I first thought was hospital smells, then at the rehab center I thought the smell was from the facility, but when I went home I kept smelling and it got worse. The smell seemed to be coming from my skin and everywhere. No one else could smell it which made me question my sanity. A doctor visit at least helped me to know I was not crazy and we have a plan of action to address the issue. He believes it is related to the high doses drugs I was on for an extended time due to complications. The hope is that it will resolve now that I am detoxing from those drugs.

# case 0619

I had polyp surgery. Several months after the surgery, I started getting whiffs of rancid or rotting meat constantly. Since my first sinus surgery I have had two other surgeries. My polyps are gone or under control, but I have no smell. On occasion in the past ten years, with the help of steroids, I have been able to lightly smell for about three times. My doctor now says there is nothing he can do to bring back my smell.

# case 0620

I can't remember ever having been able to smell. I can taste, however. I'm not sure if I can taste as well as others, but I can taste. I have a deviated septum and it's very hard to breathe. I can't sleep without nose spray. I had septoplasty surgery and it helped for about two weeks and then my nose redeviated. My doctor said that I have a 50/50 chance of being able to smell afterwards, but it didn't change one bit. I still can't smell and I'm not able to breathe without my nose spray. I tried to see if I could even smell anything but nothing worked at all.

# case 0621

Six months back I noticed that I constantly smell sandalwood. I took zinc supplements and they helped. This kept happening once in a while but got cured immediately using zinc supplement. Since last week I have started getting a rotten smell instead of the sandalwood scent. It is not constantly but at times I get this smell. I have started with Zinc again. It is helping.

# case 0622

A couple of years ago I said to my doctor that my sense of smell sometimes drops out for no apparent reason and then it can return and I have sharp smells of everyday odors, like a woman's strong perfume or car fumes. Now, at the tail end of a cold, my sense of smell came back strongly for half a day but now it's gone again. Not that I am too worried about it, as it can be handy not to smell. It's a minor ailment in the greater scheme of things.

# case 0623

I badly banged the back of my head and fractured my nose and skull. Since I came out of the hospital after my accident I have no sense of smell and it is really getting me down. Food is not as good as it used to be...

# case 0624

More than 20 years ago, I had a severe case of the flu. I then realized that I lost my sense of smell. With time it returned a little but is has never been the same. I think the air conditioning in houses, business and cars worsens the problem. When I travel overseas and I am more in an open environment, I can smell some of the nice smells and less of the bad smells.

# case 0625

I am not sure that I ever had a sense of smell. My earliest memory of not having a sense of smell was when I was about 8 when my aunt asked if a particular plant was lemon or orange, I had no idea how to tell the difference by smell. When I was about 13 someone in my class let off a stink bomb in assembly and our class was made stay behind until someone confessed. I had no idea what the fuss was about. When I was about 19, I went to the doctor about it and was sent for an X-ray and was told that everything was in the right place and therefore I should be able to smell. I was given a few vials to sniff, vinegar, ammonia and others. Needless to say, there could have been water in the containers as far as I was concerned.

# case 0626

My sense of smell declined, which I noticed because my experiences of dog waste and food that I was cooking changed over the course of two years.

# case 0627

A steak dinner was good to everyone else, but tasted bland to me. Then I noticed everything was bland. Nothing had taste, and I could not smell either. I can still taste sweet, bitter, sour, spicy, and salty, but I can't distinguish the difference of lemon or lime, chili powder or ground pepper, or taste any flavors. I live on textures of food, and try my best to remember what it used to taste like so I can enjoy it. Sometimes I get depressed about not being able to taste, especially in a group of people but that is just how my life is so I try not to get down about it. Smelling is more of an everyday problem than tasting. I am always worried if I stink even though I shower and use deodorant. I am always worried I will not smell smoke in the house, so I check my smoke alarms often. Once I forgot I had oil on in a pan while I went into another room, and it was only when I looked up a few minutes later that there was black smoke pouring from the stove. I only eat leftovers the day or two after I make them for fear of it being spoiled.

# case 0628

When I was younger I noticed that when people would tell me to smell certain flowers that I couldn't notice anything different about them. They just smelled like the air. I can only smell garlic, coffee, vinegar, and nail polish. Some things smell hot or cold but other than those four items everything smells the same and I describe it as it smells like air.

# case 0629

I noticed that after taking medicine for high blood pressure I lost my sense of smell. I am no longer on the medication but my sense of smell has not return.

# case 0630

I have this unusual chemical in my nose. At first I thought that I smelled my own body odor because the smell is more intense when I am sitting down and more body heat is rising up. I eliminated this theory by taking a thorough bath and using plenty of deodorant. Then I sat down and still had the strange smell in my nose. My nose is clear and there is no infection. Sometimes the smell is more intense than other times. The whole situation seems to be getting worse. As a photoengraver I worked with a lot of nitric acid fumes for 50 years.

# case 0631

I have this terrible smell in my nose. Everything around me seems to stink. I also feel nauseous most of the time.

# case 0632

During a cold I experienced the usual loss of smell and taste. When I started feeling better from the cold symptoms, I realized my taste and smell were gone. My ear nose throat doctor didn't find anything and concluded the loss was caused by the virus. For the first few months everything (food, toothpaste), if it had any taste, would taste like perfume. I also realized that the only thing I could detect was if something was sweet. I found myself eating more sweet things, just because I could at least sense something instead of nothing when I ate. The loss of taste greatly altered the taste of coffee, chocolate, and any kind of soda. In the past year I have been able to drink coffee without sensing a strange taste. I still do not taste the true flavor of the coffee, but it is bearable, most of the time. Whenever it starts tasting unpleasant I just quit drinking it for a few weeks. (The same thing has happened with eating anything with chocolate.) Sometimes I think I can sense something when eating or drinking, but it is never the real taste of the item. I wish I could find something that would bring back these two senses. I feel many times like not eating, or like when I am eating that my mouth may as well be full of sawdust for all the satisfaction I am getting.

# case 0633

After I had a heart attack one year ago I have a continual problem with my sense of smell that also affects my sense of taste. Something as ordinary as water can cause a horrible smell.

# case 0634

I have had a really bad cold. Initially I assumed I could not smell because I was blocked up but when my nose was unblocked I still could not smell anything.

# case 0635

Most of the time I feel my sense of smell is gone. I use medications for allergies and asthma as needed. I also use a continuous positive airway pressure machine.

# case 0636

I suffered from severe cold/flu symptoms resulting in respiratory congestion. As the symptoms subsided my regular olfactory sense never returned. For about a year or two thereafter I experienced "phantom" smells related to paints and solvents and could barely detect some of the more pungent food smells (onion, garlic and fruits such as peach and citrus). Some of the early phantom odors would persist for hours at a time. As time progressed, all of these sensations ceased to the point that there is no odor detection of any kind at this point.

# case 0637

I don't remember ever being able to smell. I remember my mother having flowers in the house and she would smell them and say how nicely they smelled and I would put my nose all the way into the flower and try to smell them, but nothing happened. I didn't know why I couldn't smell. When I realized that I could not smell anything I tried to smell everything that I could, but nothing worked. I tried to smell scratch n' sniff stickers and I couldn't smell them. But, when I stuck my tongue out and touched it to the sticker I thought I tasted something. So, I've tried this with other things that aren't necessarily edible just to see what it smells like. I still can't smell anything. I burn food, I've eaten rotten chicken — a mouse died underneath a piece of furniture and I never knew it was there. The list goes on and on.

# case 0638

One day I used perfumed body oil and noticed that I could smell it but it wasn't as strong as usual. I asked my wife to smell it and she said it was all right. About three months later my son was burning something in his room but I couldn't smell anything. Now I can't smell my younger son when I'm right over him changing his diaper.

# case 0639

Over the last couple of months, it seems that 80% of the time when I breathe, I smell tobacco smoke (like a cigar or pipe) and it inhibits me in taking full breaths. It seemed to go away for a while right after I had a cold and my sinuses were very clear.

# case 0640

After I contracted a sinus infection I lost my sense of smell.

# case 0641

I'm not sure how this happened, but I lost my sense of smell at 14, or at least that's when I first noticed it. If the odor is strong, I can smell it, but that's about it. I used a nasal spray beforehand while sick.

# case 0642

Since I had a work accident I developed a heightened sense of smell and taste. Sometimes it is more acute than other times and it is becoming problematic as I feel nauseous sometimes with particular smells.

# case 0643

About five years ago I lost my sense of smell so I went to an ear nose throat doctor and he told me I needed surgery because one of my nostrils was blocked and I had polyps. After the surgery he said it may take a while for the sense of smell to come back. It still has not come back.

# case 0644

I had a septoplasty four months ago. Since then, I smell nasty stale smoke odor.

# case 0645

I had atrophic rhinitis since I was a teenager. Despite that, I had a normal sense of smell. I lost my sense of smell after a heavy cold two years ago when I was 45. Since then I lost my sense of smell completely. I am very sad about it and God knows what I will not do to get my sense back.

# case 0646

I knocked myself unconscious after falling. After leaving the hospital many hours later I noticed I couldn't smell anything. The next day I mentioned the lack of smell to the doctors at the hospital at a follow up, and they said it's common. This has been three months ago and I still haven't been able to smell a single thing.

# case 0647

When I was younger, whenever I cooked a meal I always smelled the aroma of the food and was able to tell if it was good, or delicious, or burning. But as I grew older my sense of smell changed. Now I can't tell anymore if food is burning or if I put on too much perfume.

# case 0648

As far back as I can remember, I have never had a sense of smell. I have never had a medical examination to determine the cause. I figure, I was born this way and I've survived this long!

# case 0649

Following a bout of sinusitis three months ago I have been left with a reduced sense of smell. What is worse are the phantom smells, consisting of acrid smells of acetic acid and also bad drain smells. This condition is driving me to depression.

# case 0650

I believe I lost a great deal of my sense of smell due to using an unapproved homeopathic product. I noticed this when I was in the car with my family and we came across the smell of a skunk, which I could not smell.

# case 0651

Gradually I noticed that I constantly could smell only bad smells. I felt like I had to constantly clean everything and be sure that I was very clean about myself because I felt everything smelled bad. It started to make me very nervous. Then I realized that when I walked into a restaurant, I didn't smell the aromas anymore. I realized that I couldn't smell cleaning products. Although my eyes and body will react if I smell something harsh, I can't smell it. I have to be extra careful about keeping everything clean because I can't smell body odors or home odors. I can't smell perfume. I can taste food, but cannot smell it. It's kind of sad because I can no longer enjoy the smells of flowers or of a baby, fresh clean clothes, the ocean. I can sense the smell of cold before a snowstorm or rain storm, but I can't smell it anymore. I miss the smells of a bakery and good food. Nobody in my family has this as far as I know. I have never had a doctor ever ask me about it. I never think to even mention it to a doctor, because it sometimes seems so normal.

# case 0652

I used to have an amazing sense of smell. I could tell who was coming around the corner before I saw them since I could smell their odor. But then I realized that I couldn't smell after on several occasions people commented on smells that I couldn't perceive. It may have been caused by head trauma since I have been in several accidents. I have not been able to smell for over two years and I miss it so much...

# case 0653

I can hardly smell anything, only very intense smells. I cannot smell gas leakage, burning smell, rotten and stale food, breath smell, body odor, etc. I believe it has been like this since my childhood.

# case 0654

I started to perceive a smell six months ago. It came on rapidly, in a few days. I first thought that my computer was burning up, because the smell came when I was sitting in front of it. As the days went on, the smell continued and started to appear in other places, down stairs in my house, outside, in my truck and everywhere else I went. After an aneurysm has been removed the smell seemed to go away for some time. However, it did come back and is still with me today.

# case 0655

I noticed that my libido was way down and then I realized I had also been having problems smelling things. I can smell things if I place it next to my nose but I have not been smelling much lately unless it is a very strong odor. I felt there was a connection between the libido and the change in olfactory sensation... so I looked it up and there is.

# case 0656

I started using an unapproved homeopathic nasal swab nine years ago. Around seven years ago I started smelling what I perceived to be mild cigarette smoke at work, home and sometimes in different stores. That dissipated after a couple of years. Two years ago I found out that I had an infection in my lower sinuses so I had nasal surgery to make the sinuses drain better. Infections have still plagued me. My sense of smell has slowly been going away for about a year and a half. Last spring I remember being able to taste strawberries and to taste the vanilla in cake icing. Slowly over this past winter and now into spring I can't smell anything. Hence I only perceive the taste of sweet, sour, salty, and bitter. I can't identify the particular foods that I might be eating. I can't smell garlic or onions. I can't identify any fruit juices, etc.

# case 0657

Twelve years ago I was in an accident after which I lost my sense of smell. Only sometimes I'm able to smell a strong odor like that of dry fish.

# case 0658

I realized that my sense of smell was diminishing when friends said they could smell something burning and I couldn't smell anything. Now I can't even smell solvent or diesel. Last year I have had blocked sinuses and had to take nasal spray. If only I could smell the roses again.

# case 0659

I was born without a sense of smell. I can taste with my tongue, or at least it seems like I can. All air is the same to my nose.

# case 0660

I use to love the smell of almost anything but recently everything I smell makes me get sick to the point where I want to throw up. I don't know what could have caused this, but I wish I still could smell things without getting sick. The smells are also much stronger than usual.

# case 0661

I was having sinus problems and infections. I was taking medication for the infection. Now I cannot smell anything and have almost lost my sense of taste. I am really sad about this.

# case 0662

At the age of twelve or maybe even before I told my mom that I can´t smell anything anymore. First she ignored my concerns but after a while she took me to a doctor who did a test which was negative. When I was ten years old I had infectious mononucleosis. The doctor told me it could be a consequence of this that I lost my olfactory sense. For many years I did not even think about this. I can´t remember ever smelling anything and I also don't remember when I actually realized that I can´t smell. In my early 20s I realized that I am really unable to smell and got sad about it. There is not much food in the world that I don´t like. But due to my loss I developed other possibilities of tasting food. I feel it. And if I don´t like the consistency, I don´t eat it. I can also "taste" the differences of cheese for example. Not by taste but by consistency.

# case 0663

I developed a very severe upper respiratory infection after visiting somebody in the hospital. Later I noticed that my sense of smell had gone almost totally. My consultant is of the opinion that my anosmia was caused by the "neurotoxic" effect of the infection.

# case 0664

I can remember smelling things when I was little, such as chocolate chip cookies baking in the oven. Then I stopped being able to smell most things. Now I can smell only one or two things. One of them is gasoline. I cannot smell other strong odors though — such as when a family member says that there is a strong smell from a skunk in the neighborhood — I can't smell anything at all. I cannot smell foods or cooking odors.

# case 0665

I had problems with the smell of smoke/burning for some time. I was diagnosed with olfactory groove meningioma and had surgical resection of same. I have lost my sense of smell completely now.

# case 0666

I had a poor sense of smell for many years. Currently I can smell almost nothing. I generally fake being able to smell because when I say that I can't smell something that everyone else can most people respond, "I can't believe that!" or "What? That's impossible, can't you smell it at least a little?". I use unscented products when at all possible, because I can't tell if something smells too strong. I worry about body odor, although not obsessively. I can't smell sour milk, or spoiled food, or skunks, or natural gas, or mown grass, or cat urine. I can't smell freshly baked goods or citrus fruits or steak grilling. The only break in this that I've had was when I took Topamax for prophylaxis of migraine headaches. I walked into a bodega and thought, "Wow, that bacon smells great!" and then realized that while I'd gone there every morning for years, I'd never noticed the scent. That day I opened a carton of orange juice and it smelled wonderful! My sense of smell never fully returned, though, and eventually I stopped taking Topamax because my headaches were controlled through other means. I was back on Topamax briefly a year or so ago, but I didn't have the same reaction as the first time. This has been unpleasant and hard to explain to people, and is generally dismissed as unimportant. But just recently one of our cats urinated on a piece of carpet, and it apparently reeked, and the smell was making my boyfriend nuts, and I couldn't smell it at all. His reaction to me was complete disbelief, as if I was faking that I couldn't smell something horrid. Oddly, I do still enjoy food, and can taste things reasonably well. I can't distinguish spices or herbs in a dish the way some people can, but when I cook, it seems that I don't make anything taste awful because of my lack of smelling. Occasionally I can smell extremely sharp chemical odors, but even that has become infrequent. It used to be that commercial hand soap would smell extremely strong to me, and that I would keep smelling it on my hands for hours. I can't remember the last time that happened, though.

# case 0667

About two months ago I got body pain and coughing at night. I did, however, not have a runny nose. Since that time I lost my feeling of smell and food does not have any taste to me. I am so upset that I can't enjoy smelling and tasting the food. I have anxiety and do not sleep well.

# case 0668

I had the flu for a month and when the flu was over I noticed that I lost my sense of smell.

# case 0669

I had a head injury from an accident seven years ago. I had a fractured skull with multiple hemorrhages but recovered fully except that I lost my sense of smell.

# case 0670

I sometimes feel that I smell a burnt odor that comes from inside my nose. I'm not sure if it really comes from my nose, but I can smell it and the people around me cannot. Sometimes the odor is a burnt odor and sometimes it is a foul smell. I fear it's coming from my brain.

# case 0671

I think I lost the ability to smell subtle smells first and then the ability to smell strong odors like petrol. I used to have regular colds and especially in the months following monsoon and in the winter I used to have a terrible time with colds. Now I have stopped enjoying food and the aroma of particular dishes, the smell of flowers, and all other smells. I also find it dangerous that I can't smell a gas leak in the kitchen or something getting burnt.

# case 0672

I had the occasional loss of smell with colds and congestion. In the last few months I have noted a continual loss of all smell. I can't even smell solvents or any foods.

# case 0673

Last month I got a severe upper respiratory virus. I noticed odd smells and tastes. Later it became apparent that I had completely lost my sense of smell. It appears I can kind of sense the alcohol in say, my cologne, but not the smell of the cologne, nor any foods. I have had many respiratory viruses and have never lost my sense of smell before. I previously had a very acute sense of smell.

# case 0674

I had a long-lasting bad cold. After it cleared up I realized I could no longer smell anything except a single peculiar odor, rather like rotten peanuts soaked in vinegar. Everything smelled like this in varying intensities: fresh-baked bread, cat food, mown grass, gasoline — all just the same. Only a very few powerful smells, like menthol, are still partially recognizable through the masking odor. My sense of taste is also very diminished. Very salty, very spicy, very sweet still gets through, but no nuance, and certain things, like apples, now have a foul taste to me. It's a real drag! And according to my ear nose throat doctor there is a very poor prognosis for recovery.

# case 0675

Last winter I used hot salty steam. I covered my head with a towel and inhaled the hot salty vapors. The very hot vapors caused damage to my sense of smell.

# case 0676

About two years ago I noticed that while I was cooking people commented on the smell but I couldn't smell anything. That's when I realized I couldn't smell anymore. I still can taste things like sweet, sour, bitter, salty, but if I was blindfolded and you ask me what I was eating I had no idea.

# case 0677

I lost my sense of taste and smell after a bout of flu. A specialist told me that there is nothing he can do. I don't appear to be congested and can breathe through my nose normally.

# case 0678

I first became aware of phantom smells about three years ago when I would suddenly get a very strong whiff of burning paper. This was so strong and persistent that I would often get up at night and search for the source, certain that something was burning. This varied in intensity, but never went away. Later the smells took on differing characteristics running the gamut from the odor of fresh baked cinnamon buns to fetid swamp smells. As I write, I'm being plagued by the smell of car exhaust. These are not slight odors. In fact, they are sufficient to wake me from a deep sleep.

# case 0679

I am an anosmia patient. I have consulted many ear nose throat specialists and homeopathic doctors. But no one was able to detect the reason for my anosmia. They had given me a lot of medicines, nasal sprays etc. but there is no detectable change in my condition.

# case 0680

As long as I can remember I haven't been able to smell. It's not a problem for me, and it has never been. I live with my parents now, and I hope that I won't let the house burn down when I'll live on my own.

# case 0681

At the age of 21 I felt that I had a bad sense of smell. It gradually became worse. I consulted various doctors. A growth inside my nose was removed by endoscopic surgery. This did not improve my sense of smell. Now my sense of smell is completely gone. I can't smell anything. I can't even identify the flavor of tea and coffee.

# case 0682

I lost my sense of smell and taste during a very bad cold. My smell is very different now, very strong and sickening, if I smell at all

# case 0683

I have never experienced or perceived any smells, good or bad, from food or from other sources.

# case 0684

I completely lost my sense of smell after rhinoplasty ten years ago. I also have frequent phantosmia.

# case 0685

To my knowledge I have never been able to smell. My parents assert that I was able to smell at a very young age but I have no memory of that. Early in elementary school I realized through interactions with classmates that I in fact was missing out entirely on the sense of smell. My parents never took me seriously until about the age of 11, at which point I made several visits to an ear nose throat doctor. I was told that a diagnosis would be unlikely as there could have been many causes for either congenital anosmia or loss as an infant. Now as an adult I am again seeking to investigate my nose problem and hope to receive better treatment. Doctors always seem skeptical at first that I have absolutely no sense of smell.

# case 0686

I was born without a sense of smell. I noticed around the age of five that other people my age did perceive things to have a scent, and I pretended until I was around 13 that I could smell, as I didn't want to be abnormal. It worries me a little bit that I won't be able to smell if there is a fire or gas leak, or if food smells bad, or is on fire in the oven.

# case 0687

I had a moderately severe sinus infection at age 35, and even when all other symptoms had gone, I still had no sense of smell. After a month or so, it began to return gradually over a span of several months. At first, everything had a strange burnt smell. Then, the smells were different, but weren't right... Something like roasted chicken would smell different than steak, but neither food smelled the way my brain remembered it should. After a few months of this, I was finally able to smell most things correctly, but there were still some odors (ammonia, for one) that were completely non-existent. Three years later I've had another sinusitis episode, and am back to the "burnt" smells stage. I imagine the recovery of my ability to smell will follow a similar pattern.

# case 0688

I have allergies and a constantly running nose. I only smell strong odors such as hydro chloride. A week ago I went to a doctor and complained about my reduced ability to smell. He said I got hyposmia and anosmia. I still can smell, but the ability is reduced a lot. For example if normal people can smell something from 5 inches, I can only smell it from 1-2 inches away.

# case 0689

For the last three years I had problems with my sense of smell. Sometimes I can smell nothing and other times I can smell only very little. Petrol or perfume very close to my nose I can usually smell but not much else. My ear nose throat doctor found no problems inside my nose. The smells I smell are different from how I remember them.

# case 0690

Prior to a sinus infection my sense of smell was unusually keen, but since then I have anosmia. About a month after I contracted the sinus infection, I saw an ear nose throat doctor, who, after treating me with antibiotics and steroids, performed sinus surgery. Since the initial infection I've had chronic sinusitis and rhinitis. As with any other significant change in one's ability to perceive the world, anosmia has become something I don't really think about unless someone says something about smell. As for eating, well, I just experience food differently than I did before. One thing that remains a puzzle to me is that I'm not able to recall smells the way I can sounds or images.

# case 0691

I have no sense of smell. I can regain the capacity to smell by doing hard exercises, but when I stop exercising, the smell dysfunction comes back. I took tablets and nasal spray that helped my get my sense of smell back, but when I stopped taking the medication, I returned to not smelling anything.

# case 0692

Following a major operation for the treatment of pseudomyxoma peritonei at the age of 38 I experienced a loss of smell and taste. This lasted for around two months although on occasion foods tasted and smelled "wrong" for some time. Three years on my smell has returned to normal however my memory of foods and drink at that time repulses me now and I am unable to eat and drink certain foods I enjoyed prior to the surgery.

# case 0693

My hearing has progressively worsened over the years. To investigate this problem I was given an MRI after which I ended up having anosmia and ageusia. The onset was immediate. It has persisted to the present time.

# case 0694

Two years ago I started noticing strange, irritating smells in my home. Nobody else smells what I smell. It isn't just at home but anywhere I go and it appears that my nose has become supersensitive to everything and the distorted smells are not only irritating but depressing. I can't even define the smells that come through my nose except that they are very unpleasant and sometimes irritate my throat also. I am constantly chewing gum or using cough drops to disguise the irritation. No more wearing my favorite perfumes or using scented lotions or soaps. Some of the so-called "unscented" products still have an unpleasant odor to me. I have been to an ear nose throat doctor but the prescribed nasal sprays did not affect the distorted smells. This situation has truly frustrated me but I know there are much worse things in the world than this problem.

# case 0695

I used to enjoy the smell of food, coffee, flowers and good perfume, the smell of a baby's skin. Now I can smell nothing, not even if something is burning or putrid.

# case 0696

About seven months ago my sense of smell started to change slowly. At first, I only noticed it in the late evening a few hours before I would go to sleep. I would sense that I smell a sharp chemical smell. It sometimes even provoked me to rub my nose. I can't really describe it. It's not like anything that I can pinpoint. No one else around me smells anything. I do have slight sleep apnea and seasonal allergies but I have never experienced this. It used to happen every few days and slowly it has now increased to every night and now even during the day when I am at work.

# case 0697

I don't recall ever having a sense of smell. I recall as a small child putting ammonia in a bottle under my nose to try to "smell" something.

# case 0698

When I had cold-like symptoms my doctor thought it was rhinitis, then sinusitis. I developed polyps which were surgically removed three times. I developed pneumonia which left me with asthma. I have suffered from hay fever since about age 25. I have become quite down and lethargic because of my various illnesses. My sense of smell and taste has never come back.

# case 0699

Till the age of 12 I had a sense of smell. At that age, I had a problem with my tonsils that was resolved using some injections. My tonsil problem disappeared, but thereafter I lost my sense of smell permanently. However, it did not affect me in my day to day life. The only loss was enjoyment of good smells.

# case 0700

I have a smell disorder since last year. When I smell deeply I feel that there is some burning or chemical smell which is not bearable. The problems began when I was slapped in the head.

# case 0701

About two years ago I noticed that I didn't smell lemon when I was cooking pancakes. Then I realized that I wasn't actually smelling most things. Some smells would still get through, for example toilet smells and garlic. At the time it started, I had hip surgery and — irrationally — thought it might be connected. It improved slightly and I forgot about it until yesterday when realized I really missed the subtle spring scents, like bluebells, grass.

# case 0702

I have had olfactory hallucinations for more than a year. They sometimes last a few hours up to several days. Some weeks I experience no smell at all and then it comes back. The smell is like bad perfume and lots of it. I had an accident prior to the onset of this problem. To me, the olfactory issues are very bothersome and they are affecting my daily life. I have not been able to get a diagnosis or find a specialist or someone experienced in my area.

# case 0703

From the age of 22 my nose regularly gets blocked on exposure to cold air or after drinking a chilled drink. I have taken many nasal sprays and I work in the menthol industry. Today I am unable to smell any good or bad smell.

# case 0704

My mom was unable to smell and my sister and I are both unable to smell. It is congenital. Sometimes I can smell strong peppermint or perhaps an orange when it is being unpeeled but I can't smell perfume, flowers, natural gas, trash or anything like that. I enjoy food but think I just taste a combination of salty, sweet, sour and bitter. I can't tell the difference in spices or in fruit flavors of candy or soft drinks.

# case 0705

Last summer I lost the ability to smell or taste. I have recovered a little, but not much. I still can't tell what ingredients to add to a recipe. I still can't smell perfume unless it is really strong and held under my nose. So I don't know if I am wearing too much perfume or if I stink. But my condition also has advantages; I am a nurse and when changing colostomy bags I don't smell a thing! I have to check my patients because I don't know if my aides are doing their job. I wouldn't be able to smell a gas leak or fire. I miss simple things like smelling fresh cut grass.

# case 0706

I was taking nasal spray for about ten weeks. Shortly after I noticed first my smell diminishing and then my taste. It hasn't recovered yet. It's very annoying. I miss the smells around me!

# case 0707

Four months ago I had a cold with fever and inflammation of the sinuses. After the cold I thought that my sense of smell would come back. But I was wrong. I smell only things which are close at my nose. Before this cold I could smell always very well but now I don't even smell when I'm cooking. Recently, eggs started to taste sour.

# case 0708

I visited my brother and he and his wife could smell something burning that I couldn't smell. That's when I realized that I had no sense of smell.

# case 0709

In my forties my senses of smell and taste seemed to diminish gradually. Earlier in my life my taste and smell were extremely acute and precise so not having these senses is a real loss. I occasionally experience smells clearly but more often than not I can only identify a smell as being strong, not whether it's nice. I feel I have lost a lot of memories along with my loss of smell.

# case 0710

Twelve years ago, when I was 25, I started to intermittently lose my sense of smell. A few years later I could only smell if I put the smelly item right under my nose. Now I cannot smell anything anymore.

# case 0711

I noticed a sudden loss of smell and taste six months ago. There are several things I can smell: smoke, gasoline, strong perfume, and a certain type of incenses that are placed in water. As for taste, I can only sense the texture of foods and feel some hot seasoning like hot pepper.

# case 0712

I believe I have congenital anosmia. I don't ever remember being able to smell and just sort of "went along with it" when I was younger. It was in high school that I began to wonder. Once I said something to my mom and she was only slightly surprised because my aunt, my grandpa, and great grandpa also have had anosmia. It seems to hit one person per generation. I grew up next to a dairy and wouldn't notice the cows if I didn't see them. People would comment "oh you can sure smell the dairy today" and it was never different than any other day or place to me. If I don't see what type of soda pop I am drinking I don't know. I can tell if it's regular or diet due to sweetness/lack thereof. But Coke, Pepsi, Sprite and Cream Soda all taste the same to me. I am picky about the textures of food. If it is super strong I can sort of taste mint if there is a lot of it added.

# case 0713

I had skull fractures after an accident. Now, four months later, I have largely recovered, but I have lost my taste and smell. I have a constant metallic odor and taste in my mouth. Is there anything I can do to change that and get my sense of smell and taste back?

# case 0714

A few years ago I was described a new medication for diabetes. After taking the medication for a month I realized that I lost the sense of smell and taste. I was told that the medication may have caused the problem. It was confirmed after my brain scan that some of my nerves were damaged by the reaction to the medication. I was told that the sense may come back after a few years but it didn't. Life is not the same. Anything I eat is the same as eating or chewing cardboard. My life is now full of depression.

# case 0715

I recently realized I can't smell anymore. The last smell I remember smelled like something was burning. A few days later I couldn't smell anything. I still can't.

# case 0716

I don't recall ever having a sense of smell. Over the years I have noticed it more at work as people often mentioned the smell of the microwaved popcorn. I also work on a candle business and have no way of assessing the fragrance in a candle...try as I might. I also noticed that when I cook (which I don't enjoy at all), I have to always set the timer because I cannot rely on my sense of smell to tell me when food is done. I burn food regularly and have gotten used to the taste of burnt toast.

# case 0717

I recently was admitted to the hospital and diagnosed with aspiration pneumonia. I was in the hospital for five days during which time I was on oxygen. Around the third day I noticed I could not smell anything anymore. I mentioned this to the nurses but they said it was probably because I was congested and would be better soon. That was almost two weeks ago and I still cannot smell anything.

# case 0718

For the last few years I have had varying smells: smoky, dusty, rotten, astringent... These smells seem to come out of nowhere. At the beginning it was only at night, then they would come in the car, not often at work.

# case 0719

For two years I have not been able to tell the difference between smells. People say that I smell funny and I don't smell anything. When I cook I don't smell anything. Some things, like fresh fruit, I can smell but not many other things.

# case 0720

About ten years ago I noticed that I could not taste what I was eating. Now that I was aware of this I missed all the flavors and scents that I used to enjoy. I went to a neurologist, an ear nose and throat specialist and a general practitioner. I was told that sometimes the sense of smell just wears out and sometimes it comes back on its own.

# case 0721

For the last 13 years I experience a sweet sickly smell everywhere I go. I sometimes go for 18 months without the symptoms and then it returns out of the blue. I had liver failure 16 years ago and I never got this smell before then and therefore wonder if this has anything to do with it. It smells like one of those plug-in air fresheners and can last for up to two weeks before it disappears. I haven't told my doctor because how do I explain a funny smell in my nostrils?

# case 0722

It started with a sore throat and ended up with a common cold. On the third day I began smelling a sweet perfume that no one else could detect. It was constant. I could still smell other odors, but the perfume was always there, too. My sense of taste was still okay. It is now day five and the perfume is still there. At this point I don't know how long this will last, but I am concerned because about eight years ago, I had the flu. At that time, I had a severe sore throat that ended with me losing my sense of smell for over a year. Going hand in hand with this, I also lost my sense of taste, except for sweet, salty and spicy tastes. While I had the sore throat, I smelled rubbing alcohol very strongly, though there was no rubbing alcohol present. This lasted for a couple of days and when that smell left, I lost all sense of smell completely. Gradually, after a year, it did return, however never as strongly as before. Now I am worried that the same scenario will happen again.

# case 0723

I can't recall ever really having a sense of smell... at least, not at the same level or intensity as those around me. I feel that my sense of smell has always been similar to the situation of a blind person that can recognize silhouettes — while I can occasionally detect a smell/odor, I can't identify what it is. By this, I mean that occasionally I might walk past a bakery and detect a smell, but I can't tell if it's bread or meat pies cooking. I have rarely smelled human odors. I've heard others around me complaining about a person that smells of body odor or garlic breath... it's all news to me! I can smell strong perfumes but I would never be able to identify the brand... not a clue about that... they are all the same to me. Most foods have no discernible flavors — roast beef and roast lamb are all the same to me. My food tastes are usually related to texture, and sweetness, sourness or saltiness. I certainly can't detect if a particular dish contains, for instance, ginger, or cinnamon, or cumin, etc. I tend to like foods with very strong flavors and very noticeable textures — smooth, crunchy, crispy etc. I've never had this condition diagnosed, or even consulted a doctor about it. I've just accepted it, and sometimes consider it advantageous. After all, I'm not burdened by intense, overwhelming bad smells. I've always thought that my anosmia was related to my deviated septum, though I'm happy to acknowledge that this could well be wrong.

# case 0724

I always had a better sense of smell than other people. I am sensitive to odors. I could smell food burning before other people in the house who were closer to the kitchen, and I always was sensitive to the smell of some objects: clean sheets, bleach, and I could tell if the food was salty just by the smell. After I turned 50 I became even more sensitive to smells, and being more and more able to identify odors that other people could not even smell, and now it has become definitely unique: I enter a building and I smell what someone is cooking; then I concentrate on the smell and I can tell it is banana bread, or meat stew... while others can smell nothing at all. I can distinguish the smell of pee, fruits, vegetables, sugary foods, sweat, wet earth, rotting vegetables, etc. at a certain distance, such as being in another room...

# case 0725

I was diagnosed with essential tremor and with it I gradually noticed a loss of sense of smell. I have been trying acupuncture as an alternative therapy. The effects of acupuncture are not long lasting but it was a revelation to me as I would be able to smell things for a couple of days after the treatment. I no longer get acupuncture. I just shake. Every once in a while for no apparent reason I can smell for a short time. I am really interested in this as it is so odd that I can smell once in a while. Does this mean my nerves aren't damaged?

# case 0726

I have had chronic sinusitis for the past year and intermittent sinusitis for many years. Recently I noticed that I am unable to smell odors like cow dung or dead mice or accurately smell wine. I have a mucus problem and a metallic taste in my mouth occasionally.

# case 0727

I lost my sense of smell ten years ago when I was accidentally hit in the face with a bat.

# case 0728

While camping a coyote sprayed/marked our large tent during the night. The smell was so intense that it woke us all up from a dead sleep and caused us to vacate the site and head home for the rest of the night. To me the smell could best be described as rotten onions and caused my eyes to burn and me to sputter/cough. The rest of my family seemed to have similar reactions; however I found that my sense of smell was altered. For years after the incident I could either not smell unpleasant odors or registered them as something else completely (the same odor could smell like something different each time I came across it). For example, skunk would smell fruity/flowery one day, then like burning tires the next. (In middle/high school you can imagine what people thought when I commented on the "pleasant" smell during a bus ride home). For the past six years or so, the smell of skunk has kind of settled on a musky/tire-like odor, and only occasionally can I detect rotten food. Typically, I find that rotten food has little-to-no smell, however there are random instances when it seems to have a normal rotten odor. Pleasant odors seem to have been unaffected, with the exception of roses whose smell has become more pungent.

# case 0729

I noticed that my sense of smell has changed to the point that when I smell certain scents I feel sick to my stomach. For the past nine months I have smelled a stale cigarette smoke at my workplace and I am the only one that seems to smell it. It is so weird! My co-workers think I am crazy!

# case 0730

Since I was a young boy I noticed that I could smell things no one else could. It wasn't extreme, but enough to put me off things like overcooked french fries. Throughout my life this happened many times, like in the summer when the water from the tap began to smell bad. No one else could smell it. I have been susceptible to allergies and a long period of upper respiratory infection would cause me to lose my sense of smell. I started keeping track of the number of times that I lost it and regained it when I was about 50, because a sinus doctor told me it was unusual to regain it several times, and I just got it back after a long head cold, for the seventh time.

# case 0731

When I was around eight a skunk sprayed our sleeping bags and they smelled really bad. Everyone was trying to wash them. I could not figure out what the big fuss was. I could not smell anything bad! Something like this happened many times since and each time I did not smell anything; or if I did smell something it was just a bit "off".

# case 0732

I have had asthma for eight years. I use anti-inflammatory drugs (inhaler) on a daily basis to prevent the asthma attack. After using it for about four years I noticed that I started losing the ability to smell. I never recovered it.

# case 0733

Sixteen years ago I had an accident. I didn't realize my sense of smell was affected until I made turkey and smelled nothing. My doctor told me my sense of smell would most likely come back. It has on very rare occasions. I will get a whiff of bread baking when I pass a bakery, or of onions cooking. But it is fleeting. I don't think I lost my sense of taste. My sense of smell has been gone for so many years that I am resigned to being without it. When I do get a whiff of a scent I treasure it.

# case 0734

For many years now I have had episodes of smelling fumes. They are almost like car exhaust and it sometimes will get bad enough that my eyes burn. I usually get a really bad headache either a day or two before or a day or two after these episodes. This past weekend I had an attack of vertigo, which I was diagnosed with about ten or more years ago. The next morning I awoke with a splitting headache that lasted all day. The next day I had the fumes back and have had them now for the past three days. My longest bout with the fumes was two weeks straight. They can last anywhere from a few minutes off and on up to the one that lasted two weeks.

# case 0735

I am writing this on behalf of my son. He had a perfectly normal sense of smell until he had a head injury when he was three years old. It wasn't until he was five that we realized that he can't smell. After CT scan and MRI the Neurologist said it was probably due to the injury years ago. Now, he can smell very strong odors, but he says that he "smells it in his throat", as opposed to smelling it through his nose.

# case 0736

Two years ago I had sinus surgery for sinusitis. After the surgery I had fewer and less frequent infections, but when I got an infection it was far more intense and painful. Last year I had a bad sinus infection and since that time I have had little to no ability to smell. Because I had an unusually acute sense of smell before the surgery, I do blame the surgery. I think it allowed infection to get higher into my sinuses. My sense of taste has also been greatly compromised.

# case 0737

I cracked my skull in an accident 48 years ago. From then on I have been unable to smell anything, even rotten meat. Occasionally I see something and I get the sense of what it used to smell like. My sense of taste is acute.

# case 0738

Twenty years ago, when I was 35, I had ongoing sinus/nasal irritation. I noticed that my sense of smell had been affected by this. I could no longer smell when milk had gone sour and that is still the case. It is very embarrassing because there is a certain kind of "off" smell which I am no longer aware of. This gives rise to bad breath issues because it is difficult for me to know when certain foods — dairy — is causing an odor. I do not have a problem with any other smells, so I just put up with it.

# case 0739

Last fall I began to feel like I was breathing in dust. No one else who came into my house had this sensation. It has gradually gotten worse and now my sense of smell has gotten so much worse that sometimes it makes me sick to my stomach. I feel as if I have pepper in my throat and it causes my throat to burn. I have seen an ear nose throat doctor about this, but he says it is all in my mind. I am beginning to wonder about how much of this is real and if my mind is playing tricks on me. I wish I could get an answer for this.

# case 0740

Four weeks ago I had a case of acute cold associated with nasal discharge for over a week. I have not been able to smell since that cold.

# case 0741

I had chronic sinusitis for 30 years and underwent several nasal surgeries. Whenever my sinusitis worsened my sense of smell vanished and with a shot of steroid it used to come back. Last time I lost my sense of smell was a year ago, when I was 58 years old. I consulted my doctor and got steroid shots again. My sense of smell returned, but it was very weak. I underwent laparoscopic surgery which cured my sinusitis. Now I have a very weak sense of smell. I cannot smell stinking water, stale food, dead animal, gas leaks, smoke, flowers, good food, etc.

# case 0742

I realized that I had lost my sense of smell when I was driving in a car with my wife and she said something was burning. I couldn't smell anything and kept driving and five minutes later the motor blew up. About five years on I realized that I was losing my sense of taste. I have virtually no sense of taste unless it is something very strong like chili peppers or ginger.

# case 0743

I have recently noticed I have lost my sense of smell. I haven't been asked by my doctor about this at all. I really miss my sense of smell and worry that it has to do with my nervous system, as I have been finding it difficult to cope with doing anything. The slightest thing makes me anxious. I really hope my sense of smell comes back as it is wonderful to smell nice scents and flowers.

# case 0744

Five years ago I fell and fractured my skull. When I was released from the hospital, I couldn't smell anything. It was depressing. Before, I had a very sensitive nose and loved food and cooking. I told myself to quit whining my sense of smell was nothing compared to other neurological conditions. I tried taking zinc and after about two weeks smelled something while pumping gas. It was the gasoline but it smelled sickeningly sweet. The next week going through a grocery store I smelled this extremely strong smell of dirt or fresh soil. It was chocolate in a large candy display. During the next year I quit taking zinc because I started to smell the super sweet smell all the time. But I started trying to practice smelling things, concentrating and trying to remember what they smelled like. Nothing happened for a while but about ten months later I started to smell real smells again. I kept practicing and today I would say I've got a large part back. But some things still have no smell at all and sometimes I smell stuff no one else does. I can smell our dog's flea medicine through the whole house; it has a choking chemical smell. Gas still smells sweet and chocolate only sometimes smells like dirt.

# case 0745

Six years ago, when I was 51 years old, I started smelling foul odors like rotten food, fish, and garbage. I always ask the people around me if they can smell some unpleasant smell. They always say that they don't, but I can. I can smell strong body odor coming from me, but I don't have stinky arm pits, bad breath or vaginal infection. What is the cause of this? Do I have a psychiatric problem? In my family history, my elder brother also went through this problem.

# case 0746

Sometimes I detect odors that have no source. Sometimes I experience food odors, other times the formaldehyde odor from high school biology lab when we dissected frogs, wet soil, insecticide, or other chemical odors. Not as frequently, I also taste things that aren't there. Usually these tastes are sharp or pungent — blue cheese, garlic, onion. About the same time the odor/taste hallucinations started, I began having vertigo/dizziness issues. Exams and tests by several doctors indicated that the balance function in each ear functions as it should, but the two sides of the brain do not communicate the information as it should. The odors and vertigo seem to coincide. They come and go and change in intensity together. I realize it might be coincidence, but maybe there is a correlation.

# case 0747

I could never smell. I pretended to when I was younger and my parents couldn't believe it when I eventually told them that I can't smell. It didn't really bother me until I had children of my own, sometimes I don't realize they have a dirty nappy and I do worry about not being to smell food that may have gone off.

# case 0748

I just lost my sense of smell. Now I can't smell anything at all. All my life I always said my sense of smell was the one I valued more than the others.

# case 0749

I have always had a weak sense of smell. Approximately ten years ago I totally lost my olfactory sense. I do not miss it. It has not changed my enjoyment of food. I don't know if this is a factor, but I was born with a cleft lip and palate.

# case 0750

One year ago, when I was 68, I had a cold that lasted approximately two weeks. Sometime after that I noticed that food started to taste differently. For example tomato sauce tasted more acidy. I mentioned this to my doctor at my annual physical. He asked me about my sense of smell and did a test. Without seeing what he put up to my nose I thought the smell was spicy but couldn't detect that it was coffee. He said not much can be done about this and hopefully I would regain my sense of smell and taste over time. This hasn't happened yet. Instead it is getting worse and I now smell the same smell all the time. I cannot describe the smell but it isn't pleasant. My sense of taste is decreasing. I am getting frustrated but I'm learning to live with it.

# case 0751

About six months ago I started noticing that my farts seemed to be getting less smelly. I tried several smells, fruity, honey, lemon, etc. and found that my sense of smell had diminished. I’d measure the decrease to over half sensitivity (from a 1.0 to a 0.4).

# case 0752

My sense of smell has gotten weak. Sometimes I smell weird odors. They smell like paint fumes but there is no new paint around.

# case 0753

When I turned 30 I started suffering from anosmia. I thought it was just temporary but it has been going on for one year now. My ear nose throat doctor gave me a corticosteroid. It helped, but I had to stop taking it.

# case 0754

Over the last eight months I have noticed that I have lost the ability to smell certain smells. I can still smell most things like grass, spring, summer, perfumes, petrol, paint, burning materials, food, animal waste, and others. Surprisingly, I can't smell my own bodily gas and toilet functions or that of any other human anymore.

# case 0755

I've never had a sense of smell. Never.

# case 0756

Last year my sense of smell began to diminish — quite profoundly and quite quickly. In a matter of a month or so, it was gone completely. In fact, I didn't notice it at first. It was only a few months later that I realized I wasn't smelling anything anymore. A month before my sense of smell began to diminish, I was hospitalized and had a nasogastric tube inserted and I wondered if there had been damage. Around the same time, I sprayed a lot of anti-flea pesticide and used a lot of flea repellant on myself. I wondered if the loss of smell was due to poisoning by these pesticides. I may get the slightest whiff of some scent, if I am right on top of it, for just a few seconds — then it is gone. I would describe the condition as "having no input from the world of smell" at all. Neither good nor bad input. I cannot smell food, flowers, rain, soap, etc. I cannot smell rot, mildew, dirt, my own body, smoke, gas, fire, the litter box... well, really anything. I do notice that the anxiety and depression around the loss of smell grows greater by the week. I am especially anxious about my home because I can't smell dirt and "cleanliness" (for lack of better word) and have become somewhat fixated on not letting people in.

# case 0757

About ten years ago I began to notice that I was losing my sense of smell. This gradually became worse. Now I cannot smell anything. However, I still find that food tastes good even though I cannot enjoy the smell of it. What I miss the most are the fragrances of flowers, such as lavender, lilac, and lily-of-the-valley.

# case 0758

I fell on the back of my head and fractured my skull. Now I have anosmia.

# case 0759

I cannot smell bad odors like the odor of dead rats or dirty toilets. After my wife points out the odors to me, I can smell them weakly.

# case 0760

I had a pituitary gland tumor removed three months ago. After surgery when I got home I lost my sense of smell and taste.

# case 0761

Twenty-three years ago I had a flu and started losing my sense of smell slowly. Sometimes I can smell a little but it only lasts for five minutes. Then I can't smell anything for two weeks.

# case 0762

For a long time I had severe nasal congestion problems. To overcome them I used an inhaler that gave me occasional relief. After several years I gradually discovered that I had lost my sense of olfaction but was quite ignorant about the cause.

# case 0763

About ten years ago I started to notice I was getting a lot of colds. I was blowing my nose a lot and I had lost my sense of smell. I was diagnosed with a deviated septum, nasal polyps, and a lot of infections. I had surgery to rectify the problem and it worked. I got my smell back for about two years. I had two more surgeries and in both cases regained my sense of smell. About two years ago I had the last surgery and since then I have had no sense of smell. Only after I exercise I do get a sense of smell for about five minutes.

# case 0764

I lost my sense of smell and taste in a head trauma accident. My sense of taste came back. My sense of smell has not.

# case 0765

I had a terrible sense that I smelled like cigarette smoke all the time and went to many doctors, but nobody could figure it out. Then I got an antidepressant prescribed and within days the constant smell of cigarettes was gone. However, over the next few years I found that I couldn't smell anything. Now, five years later, I can only smell very heavy perfume or cigarette smoke. The weird thing is also that if I see someone smoking, even from a distance, I can smell the smoke. It is horrible to lose the sense of smell and I am hoping there is some way to get it back.

# case 0766

I am told that when I was two I fell and injured my head. When I was five I noticed that others were not pleased when a certain bodily function occurred. I could not smell except for three things: 1. I smell fish when I open the door of a car that sat in the sun and I try to get in the car and sense the presence of excessive heat in the car. 2. I smell alcohol when I use excessive amounts of hand sanitizer. 3. I can smell the steam from ramen.

# case 0767

I have for many years suffered from sinus problems. My sense of smell has never been good since my forties. Recently I lost my sense of smell entirely. I am just left with one very strange rather sickly smell. I am concerned I will never smell again.

# case 0768

I have had five treatments of electroconvulsive therapy for a depression episode over the last three weeks. The main outcome of the treatment so far is that I appear to have gotten back my sense of smell. I was not aware that I had lost my sense of smell, but over the last week, I am very aware of all the smells around me and the memories they invoke. The smells/memories have all been happy/positive. So far, no professionals have been able to explain the reason for this, but I am seeing it as a positive experience/outcome of the treatment.

# case 0769

I keep smelling the same odor of an electrical fire. I thought it was something wrong with my heating and air conditioner but it checked out to be nothing. I smell it everywhere I go. I smell it in cars, outdoors and in other houses. It is worse in my own home. No one else smells it.

# case 0770

I noticed that my sense of smell is either non-existent or overly sensitive many months ago. I wonder if exposure to exhaust fumes at work had exacerbated the problem. I also have allergies, especially in the spring. I don't know what to think but it has become worse lately and is causing me concern.

# case 0771

I lost my sense of smell when I was 18 as a result of a skull fracture. Two days later I felt well enough to eat and that's when I first realized something with my taste was off. When I tasted the ketchup, it was very bland, and I kind of thought that was weird but didn't think too much about it. After two more days I noticed I couldn't smell my shampoo or conditioner or soap. When I explained this to my neurologist he was kind of like "yeah, that's probably permanent". I was pretty shocked, scared, and upset. I was trying hard not to cry. He said I had a 10% chance that my smell would return in the next 9-12 months. For the first three months I could barely taste anything. Now, five years later, I think I can taste just fine but if you had my taste buds for a day you probably wouldn't be able to taste anything or it would be very bland. I think my taste has just adapted. I do put a lot of salt and pepper on everything. It's a little embarrassing to eat with people I just met or who don't know I can't smell and my taste is decreased. I have "phantom" smells sometimes. I smelled burnt chocolate chip cookies a lot in the beginning. I also smelled a greasy meaty smell. Sometimes you just have these random smells that are very hard to explain and they can last for a few days at a time. I can't explain what it's like to not have a sense of smell. Believe it or not I can actually tell when some smells are in a room or if the smell just changed. The best example is popcorn. Every time someone pops popcorn I can tell right away that the smell of popcorn is in the air. Even if I had no clue it was being popped. It's something about the way the air feels when you breathe it in. I can't actually smell the popcorn. I think it's just me smelling the air around me changing. As much as not being able to smell has changed my life, it's not the end of the world and I constantly remind myself of how lucky I am that anosmia is the only permanent damage I have from the head injury.

# case 0772

Some ten years ago I started noticing that it was becoming harder for me to smell flowers, body lotions, etc... I did not pay much attention but some two years ago I noticed that I had completely lost any sense of smell.

# case 0773

First I noticed a strange odor, more or less metallic or chemical. Later my sense of smell became reduced and that odor was all I could smell. Today I seldom can smell anything... maybe once a month a very strong spice, but unrecognizable, and less often than that for a brief second I might smell something strongly odorous as I eat or drink. Of course that could be memories. I miss smells... cooking has been my hobby for many years. Now I cannot tell how much basil or oregano I have used. Even cinnamon, nutmeg and cloves are no longer possible. Dangerously, I cannot smell "bad" chicken or beef; I cannot smell the release of natural gas (on the bright side, I cannot smell methane either).

# case 0774

I don't know what happened. One day I was making salad for my family dinner, and the cucumber smelled funny. So I tasted it... and it tasted bad. I threw it in the garbage. Then I started noticing that a lot of chemical items, like shampoo, dish liquid etc.... all smelled the same. They all smelled very similar to the cucumber. No matter what fragrance the shampoo is, it smells the same to me. It's almost like I can breathe in an overwhelming chemical that is in those items and it is dominant. Every day (this started after the cucumber smell) I smell a smell that resembles the powder in instant hot chocolate. It's just not going away and I can't find anyone else with the same problem.

# case 0775

I experienced a gradual decline in my sense of smell for the last 12 to 15 years. I can smell pungent odors sometimes and other times I do not. At times, other people point out to me that there's a smell in the air and I cannot smell it. I pretend that I do smell it, but it's embarrassing when I'm asked to identify it and I cannot. I do suffer from seasonal allergies and take some medications. Overall, it's not a big problem but I hope to find something to help me regain my sense of smell.

# case 0776

For the last 15 years I have lost my sense of smell and as a result I have no appetite. I force myself to eat. I want to be able to smell the food and enjoy eating again.

# case 0777

I have an odor in my nose! Everything I smell is the same odor, even when I take showers. I even tried to put soap in my nose, sometimes it's like a wet dog smell.

# case 0778

Four years ago, when I was 60, I had a severe cold. I was very, very sick. I entered a beach house and there I smelled perhaps the last smell of my life: fungus and mold. After that, I never had a sense of smell again. I think that not only the cold was the reason of my anosmia, but the fungus too.

# case 0779

In my thirties I started having seasonal allergies. I used nasal spray and got weekly injections for my allergies for over ten years. During this time I lost my sense of smell. For about ten years I would get ten minute windows about once a year when I could smell and I would go around sniffing jars of coffee, toothpaste, orange juice. But for the last ten years I have not had any sense of smell at all. I do get phantom chemical smells, usually when I am asthmatic. I have trouble tasting foods, but I can sense tangy foods, so I tend to enjoy tart and tangy foods.

# case 0780

A year ago I suffered from a cold. After prolonged treatment it could not be cured and subsequently developed into a sinus infection which resulted in the loss of my sense of smell.

# case 0781

On more than one occasion I smelled something that others couldn't smell. The odors range from floral/perfume, wood or paper smoke, petroleum or solvent based (petrol, WD-40, butane) to cooked foods. When this does happen I always look for a source of the odor but never find it, so now I simply attribute it to what I call a "nasal hallucination". About five years ago I used unapproved homeopathic nasal swabs for a few months and my sense of smell seriously deteriorated. The olfactory hallucinations diminished.

# case 0782

Ever since I took an antibiotic I have no sense of smell. I am a nurse and this is a great disadvantage.

# case 0783

I have memories of my very early childhood, but I don't remember ever being able to smell and I was always confused by people's reaction to scents. I have never had allergies or sinus issues, and I can breathe clearly through my nose. It is a sad state of being, in some ways, but I like to think my other senses are more finely developed, even if they are not.

# case 0784

I have complete anosmia. I never recovered after a normal cold 18 years ago. I was checked by two specialists and told that it would be permanent because of neuronal damage. I lost my olfactory memories after a few years.

# case 0785

I keep smelling something weird, like old dogs, and chemical smells. The smell is throughout the house, the floors, the air. I think sometimes the air smells, and I really think it is me that smells, but I never have body odor. I am really clean. Smells can make me sick, I am always cleaning and spraying. It really drives me crazy.

# case 0786

I was exposed to paint fumes and dust for many years. I first noticed that I couldn't smell gasoline, paint thinner, and exhaust fumes. Now I can't smell much at all. I can still enjoy tasting food and am not really bothered. I am worried that my loss of smell is due to another medical issue.

# case 0787

I have had a reduced sense of smell since I had a cold a few years ago. It wasn't gone entirely but almost. It took a long time to recover and I used to smell a metallic smell every now and then. Recently I had another bad cold and smelled first a chemical smell that wasn't real, then a dirty smell. I didn't smell any real smell for around three months. It's very frustrating.

# case 0788

I lost my sense of taste and smell after a fall. The worst part is that now I always only feel one smell, which almost makes me throw up. It's always the same smell.

# case 0789

At age 16 I started to lose my sense of smell. In the beginning I didn't realize it but then other people said something about a smell and I couldn't smell anything. Recently I found that I lost my sense of taste also. I've been visiting doctors for this reason many times, and they don t have any answer to my problem. Here in South America there are no specialist in this subject at all. The only thing different in my nose is that I am always congested and often I feel that my nose is burning inside.

# case 0790

I fell and fractured my skull. One of several long term effects of the accident was that I lost my sense of smell.

# case 0791

My sense of smell has deteriorated over the past seven months and is now very weak. Smells used to be very strong and easily perceived. These days I seem to have a strange odor (in the right nostril only) which reminds me of soft cabbage, not offensive but not fragrant either. This happens frequently throughout the day. I often smell odd odors that no one else can detect.

# case 0792

While visiting Hawaii two years ago I realized that I was not appreciating floral scents nearly to the level of my previous trip a few years prior. Since then, I observed that I had an overall diminished sense of smell. I chronically have some minor sinus congestion. My doctor recommended allergy medication without any definitive testing or diagnosis.

# case 0793

I have lost my sense of smell. I can still taste foods, but cannot smell even pungent odors (skunk, cigarette smoke, cologne). I have bad sinuses. I noticed this condition about six months ago and don't know what to do about it.

# case 0794

Five years ago I noticed that I couldn't smell a bad smell everybody else could smell. I realized that I lost my sense of smell. Since that day I can't smell anything but I can recognize tastes.

# case 0795

I recently began to smell a sweet odor, not necessarily a good sweet smell. I have gone to an ear nose throat specialist but have not noticed any changes.

# case 0796

Losing your sense of smell is one of those things you do not readily notice. It was not until I could not detect overwhelmingly strong odor of gas that I knew something was wrong. I have been to every doctor and have had every test done. As of now there is no explanation as to why I lost my sense of smell. I cannot smell anything at all. I can still taste, although foods are not always as flavorful as I remember it. I do not even have mild days of smell. The only good that has come of it is that I don't crave anything based on smell.

# case 0797

For a long time I have been smelling something burning. It was so strong that I have been known to go look for something burning in other people's homes. I have not smelled anything burning for the past 10 years. I frequently, however, get a strong sensation of an odor that I know I have smelled before, when it was real, but it passes too quickly for me to identify it. I had an episode just now. It is usually a sweet odor, but not perfume. I do not like to wear anything that is scented — perfumes, deodorant, hairspray, soap, shampoo, etc. Several perfumes give me migraine headaches. I used to have a very strong sense of smell, but today rarely can smell normal things, i.e. foods cooking, flowers, etc. The decrease in my sense of smell has not had a particularly deleterious effect on my sense of taste, however.

# case 0798

I hit my head three months ago and lost all taste and smell. It has affected my eating and cooking witch I used to love.

# case 0799

I had a head cold four months ago. It was a normal cold with four to five days of a running nose, itchy eyes, head congestion, sneezing, etc. Afterwards I noticed a complete loss of smell when I started burning things in the kitchen. It took me a few times to understand what had happened. Now I can't smell bleach, horseradish, cooking onions with butter, or anything else. My doctor was unconcerned and said it would come back. It hasn't come back yet.

# case 0800

Four months ago I became ill starting in my sinuses. It turned into bronchitis and then pneumonia. I recovered, but now I smell a horrible sweet smell. Even my lipstick has this horrible smell. It is as though my brain cannot distinguish smells. I smell it as soon as I wake. If I take medication to loosen the mucus in the lung it eases, but then comes back strongly.

# case 0801

Over a year ago I suffered a series of upper respiratory infections, followed by a very bad bout of influenza. During that time I lost my sense of smell. My sense of taste was also significantly affected. I assumed that I would fully regain these senses when I returned to full health but this did not happen. An ear nose throat specialist informed me that the cause of my anosmia was probably the repeated infections I had suffered and that my sense of smell and taste may return gradually, or not at all. He also said that there was nothing he could do to treat the problem. I am one of four girls and two of my sisters as well as my maternal grandmother also have lost their sense of smell after infections.

# case 0802

I noticed a progressively diminishing sense of smell about a year and a half prior to my diagnosis of benign meningioma which was located in part in the olfactory groove. Subsequent to my craniotomy I have no sense of smell. I have been told by the neurosurgeon that it will not return.

# case 0803

I am suffering from bad smells from my left nostril for the past year. I had sinusitis and my doctor cleared the sinuses with a nasal wash. After that a bad smell started to irritate me.

# case 0804

I had an enormous amount of dental work on my upper teeth. Afterwards, my sense of taste and smell began to gradually deteriorate. Everything I eat tastes like paper and glue and it has a very negative impact on the quality of life. I want to get back my enjoyment of food and help others in this situation.

# case 0805

The other day, I woke up in the morning smelling and tasting bacon, but have had neither, nor do I have any in the house. The taste was so strong, as if I'd just eaten it. A little while later, maybe 30 minutes or so, I smelled pizza with bell peppers and onions. I could not tell if it had any other toppings. I have not had a pizza in a very long time. I've also noticed, over the past several months, a heightened sense of smell, but these are not phantom smells, unlike the bacon and pizza.

# case 0806

I have been aware for about two years that I am losing both my sense of taste and smell. I have pursued medical help to no avail.

# case 0807

My sense of smell started to decrease at a young age. When I was in my 40s I noticed even less smells. About a year ago, at the age of 55, I noticed I couldn't smell anything at all. I finally told my doctor about it when I went in because of increasing headaches, slight sensation of something stuck in my throat.

# case 0808

I was diagnosed to have obstructive sleep apnea three months ago and I have been using a continuous positive airway pressure machine since. I have noticed that my smell sensation diminished slowly and since last fortnight I can't smell anything.

# case 0809

I lost my sense of smell. With prednisone it returns after about three days. When I get off it, I lose the sense of smell about three days later. I had sinus surgery which helped with the sinus but not with the sense of smell.

# case 0810

I have had polyps since I was 18 years old. As a consequence, I lost my sense of smell.

# case 0811

I abruptly lost a lot of my sense of smell about two years ago roughly around the time I quit smoking. Accompanying that loss, as you would expect, was a change in my sense of taste. Most notably, I used to drink a lot of coffee but it just doesn't taste the same anymore and I'm down to a cup in the morning. I have not completely lost my sense of smell, but it is impaired. I don't smell odors that other members of my family do.

# case 0812

I fell down and had a concussion and a small crack in my skull. Since then I cannot smell anything.

# case 0813

About five months ago I had a severe respiratory infection and since then I have had problems with my sense of smell. The first two hours in the morning I can smell but then I lose it. When I take a deep breath I can smell.

# case 0814

About a month ago I started to develop an acute sense of smell that is very disturbing. I am becoming hypersensitive to all smells. It is making me feel sick and smells have become very strong. Even the nicest scents have become unpleasant. Pretty perfumes that I previously enjoyed, the smell of Tide detergent that I've always loved, clean, fresh scented cleaning supplies... everything! Unpleasant odors are driving me crazy. I have had acute sinusitis for many years and I'm wondering if this may have anything to do with it. I would just like an answer. Good or bad, smells seem to cling to my clothing. I take them with me wherever I go. This is most unpleasant.

# case 0815

I always had a very good sense of smell. About 7 months ago, when I was 59 years old, I started to experience bad odors that no one else noticed. Then I noticed that I couldn't smell a lot of things. Now I cannot smell anything and my sense of taste is decreasing, too.

# case 0816

I had a severe upper respiratory infection and quickly lost my sense of smell. Unbelievable!

# case 0817

I lost my sense of smell following a cold two years ago. After about six months I suddenly smelled something but then it would go away again immediately. Also if I try to smell something, i.e. sniff at it, I can never smell it but I can walk along and suddenly I can smell say a flower or someone cooking but it is very fleeting. This year I went to Colorado (USA) and within a couple of days my sense of smell came back quite strongly and stayed with me for about a fortnight but as soon as I returned to the UK it went again within a day or so.

# case 0818

I can't smell. To me, smelling is like eating chick peas; you can feel it but it doesn't have a taste. I can feel smells in my nose but they don't smell.

# case 0819

Three months ago, at the age of 55, I had an attack of acute sinusitis. Since then I lost my ability to smell. Only sometimes I can smell sharp odors and I did not lose my sense of taste.

# case 0820

I'm unable to smell. When I go candle shopping I can't smell the candles. Sometimes, which is very, very rare I can smell very strong odors. These would be terribly strong for the normal person, but for me I can barely smell them. I can't smell food cooking, etc.

# case 0821

Two months ago I noticed that although I could smell all odors as they presented (strawberry smelled like strawberry) the fact is that when no odor presented itself to me I smelled a distinct constant unchanging odor. This odor was only masked but as I said revealed itself after any other odor presenting itself has passed. Likewise, I have an unpleasant taste in my mouth which did not interfere with my diet as, whatever I ate, I tasted well enough. But in the absence of any food, the foul taste returns to my mouth.

# case 0822

I was prescribed a nasal spray for my asthma. About eight months later I noticed that I had lost my sense of smell and also experienced other side effects of the drug.

# case 0823

I had a bad flu and lost my sense of taste while I was sick. My sense of taste came back but I can no longer smell. If a product has a strong chemical smell (say, a bathroom cleaner, or gasoline) I can detect a sensation that tells me I am smelling something strong but I cannot identify the smell. I can absolutely not tell if I smell clean after a shower or sweaty after gardening. It is distressing.

# case 0824

I lost my sense of smell and went to three ear nose throat doctors. The only treatment that helped temporarily was prednisone. Now, two years later I still have no sense of smell.

# case 0825

At the beginning of this year I noticed that certain things, mostly food, began to smell very unpleasant. This makes it difficult to eat. I cannot describe the smell. It is just very unpleasant and unappealing. Also, very interestingly, the same smell attaches to my perfume which I have been using for 15 years and to my spray net which in the past was very mild smelling. At other times during the day, I will smell this same odor when there is no food or other cause for odor to be around.

# case 0826

After I had the flu I noticed that my sense of smell had disappeared. I went to my doctor and he said it was unusual to lose your sense of smell unless you had a head injury such as in a car accident. My sense of smell sometimes returned but recently it has gone altogether. I have tried nasal sprays and these worked for about an hour but then the smell was not very strong. Two years ago I was diagnosed with asthma and I noticed when I was on steroids for an asthma attack my sense of smell comes back but as soon as I am off the steroids it goes again. I am very frustrated that I can't smell anything most of the time. Recently I was cleaning the cooker and I must have turned the gas knob on but didn't realize until my husband came home and noticed the gas had been on. I find this very worrying as I have also burnt pans etc. At the moment I have no smell sensation at all.

# case 0827

I smell a musty, moldy, stuffy smell after a sinus operation two years ago. The problem is that there seems to be a smell from my nose or mouth. The ear nose throat doctor says it's phantosmia, but can you have that if there is a smell? No specialist has any ideas.

# case 0828

I remember that when I was five I told my teacher that I had no sense of smell. That is the earliest memory of me telling someone I've lost my sense of olfaction. Ever since then I've never been able to smell a single thing.

# case 0829

I have gradually lost my sense of smell over the last few years. I had a bad cold, allergy and asthma symptoms at the same time. When I recovered from the cold, my sense of smell was completely gone. I have recovered a very small portion of it since. I occasionally get a whiff of a fragrance... like when my husband first puts on his cologne, or a whiff of something burning, or even my own perfume. I sometimes get a whiff my husband or myself when we've been working outside in the heat. It is very, very frustrating to not know if I smell okay when I go out in public. I helped my husband bale hay last week and ever since then all I can smell is exhaust fumes....night and day! Awful! I also have put on weight during this.

# case 0830

I experienced a complete loss of smell and taste following use of an unapproved homeopathic product. Now eating food is similar to eating textured water. I have tested my sense of smell using fishy canned cat food and Vicks vapor rub and still have nothing. I do not know yet if this damage is permanent. I found out after the fact that this product had been recalled although I never saw anything about the recall in the news.

# case 0831

About two years ago after a bout of flu I noticed that I had lost my sense of smell. It has never returned and I have seen my doctor who told me that the flu virus had killed my olfactory nerves. I had treatment of nasal drops containing steroids, but to no avail. Very occasionally I get a whiff of something, but it passes in a second. Generally I can detect if there is a change in the atmosphere e.g. if someone sprays perfume, but I cannot distinguish the difference between smells — the whole world smells the same to me!

# case 0832

I caught a severe upper respiratory infection five months ago. I first developed a hacking cough and fever and then the symptoms went into my nose where the illness ended up as a nasty cold. When the cold entered my nose I lost my sense of smell and taste and they have since only returned to about 25% of my sensitivity before the cold. I have seen an ear nose throat doctor who told me that if my senses do not return to normal in a year the loss is probably permanent, and there's nothing that can be done. I can't smell subtle smells and strong, organic smells (feces, garbage, vehicle exhaust, skunk odor, coffee) all smell the same. They all smell like burnt vegetables in a fetid swamp. I tend to be able to "taste" acidic items like citrus (especially lemons and oranges), and vinegar. I can sense sweet, sour, salt and umami. I can actually pick up many smells if I bring the items close to my nose and inhale deeply, but the odors usually extinguish quickly.

# case 0833

Three weeks ago I fell and had a concussion and a fractured skull. I have no sense of smell or taste now and am wondering if it will return.

# case 0834

I suffered severe traumatic brain injury after an accident. The recovery was quite remarkable with nearly all functionality returning about two weeks after the accident. Approximately two months afterwards I was made aware that I had no sense of smell when I was asked if I smelled something. I also noted that my sense of taste had recently departed. Seven months after the accident there is little progress in smell or taste. Nose spray and nose irrigator (salt solution) have not brought improvement. Recently I tried a faint sniffer over the counter medicine (ammonia based-15%) and that seems to have sparked a smell improvement, which also led to an improvement in taste.

# case 0835

All my life I have had severe nasal allergies and used nasal spray several times a day to relieve the congestion so I could breathe. Over the years I gradually became less sensitive to odors of every kind and about four years ago I started noticing a major loss of smell. Now I can't smell anything.

# case 0836

Since I had a an accident during which my head hit the road my smelling capability is gone. Now, after one and a half years, there is no improvement.

# case 0837

Three months ago I had a severe sinus infection/cold/flu that was treated with antibiotics. Afterwards I could initially not smell anything but within the last week or so there is a specific smell in my nostrils. It is a burning sweet smell. If I wash my hands, take a shower, use a sanitizer, eat food, it all smells the same. Most of the time it intensifies if the smell of the item is strong but the true smell is never detected. I am afraid at this point that my sense of smell is not coming back and I will still have this sickly sweet smell forever.

# case 0838

I have occasional events of "phantom smells" ever since I completed chemotherapy and radiation for breast cancer. The smell is always the same... an acrid burning smoke... but it has different intensities… sometimes faint and lasting a few seconds, other times stronger and lingering for many minutes. It seems to ebb, building up over several days and then it begins to dissipate. At this point I don't experience it all for several weeks and then it begins again. The pattern is best described as a bell curve.

# case 0839

About seven years ago I realized that sometimes I cannot smell. My sense of smell sometimes returns for a short while then goes away again. I have had an operation but it didn't help. I still do not know the reason why I lost my sense of smell. It disturbs me.

# case 0840

Fourteen years ago I noticed that everything started to smell bad (like sewage). Water from the shower, outside, inside, everywhere and it affected my ability to eat almost anything. After the torture of this for six months my neurologist put me on seizure medicine. Within three days I could tolerate smelling most things. Problems are burning fires, cigarettes, car exhaust, and oily substances. Food has remained a problem... especially eating a balanced diet. I find something I can eat and usually eat it until I just cannot eat it again. This can last from six months to several years. The drug dose doesn't change this. I am unable to eat any poultry, seafood, ham, hotdogs, and very little beef within the last year. There are some things I can eat if I wear a swimmers clip on my nose. I can't tolerate vegetables, especially tomato and onion.

# case 0841

Many years ago I had a very bad sinus infection and it was a few weeks later that I started noticing that I couldn't smell scents. It eventually got so bad that you could put a gasoline can under my nose and I wouldn't be able to smell the fumes. I just got used to the idea of not smelling. However, I have noticed in the last few years that I am able to get faint scents of certain smells like grape bubble gum or if someone is wearing a certain smell that maybe is really strong I will get that smell up my nose for several hours and can't seem to get rid of it. I have never lost my taste; only my ability to smell.

# case 0842

About a year ago I realized that I have problems smelling when I didn't notice the smell of burning rice. After that I started noticing all the things I couldn't smell. I can't seem to smell what I'm cooking, which is irritating. Some really sweet smelling perfumes I can smell very faintly.

# case 0843

When I was 15 I injured my head. One of the results of the accident was that I lost my sense of smell. I only noticed it months later. At first my family didn't believe me but it later was confirmed by a doctor.

# case 0844

I have been diagnosed with depression in my early teen years. Later this developed into bipolar spectrum or schizoaffective disorder. In addition to many other symptoms, I experience phantom smells. They only became apparent in the past few years. The one interesting thing about it is that the false aromas are not nasty or putrid. Most times the odors were exceptionally fruity smells. Two smells in particular that I recall are an intense lemon smell and the distinct candy-version of what grape smells like.

# case 0845

Sometime around age 70 I began to lose my sense of smell and taste. I can smell some things like burning grass and gasoline. I can taste only some things like sweet and sour spices and some candies.

# case 0846

I used to be very sensitive to smells. But now I can't smell anything. Sometime when coffee is grinding I can smell it a little. But other than that I can't smell anything. My 76 year old husband lost his sense of smell about the same time that I did. I sure wish we could get it back.

# case 0847

Last year I had a severe respiratory infection for about a month. At the time, I lost my sense of smell and taste, which I thought was normal given the infection. Since then I have not had a full sense of smell or taste and about two months ago I started to get a very unusual smell. When I brush my teeth, drink coffee, beer or wine, I have the same taste/smell. Sometimes my skin smells like it and sometimes food. Not everything I eat or drink has the same smell. I still do not have a full sense of taste when I eat.

# case 0848

At the age of 27 I had a severe cold with fever that lasted for more than 15 days. Within three days I lost my sense of smell and now, at 50, I still can't smell.

# case 0849

Ten months ago I started to smell smoke and waste smells even when I was in my room. I only used to smell this during some minutes when sitting down after a long walk. Now I sometimes smell the smell of alcohol and smoke when I breath...

# case 0850

I have always had sinus issues but never did anything about them until just a few years ago. For a month or so all I could smell was something resembling a dead mouse. Then it would be an overwhelming smell of flowers. A CT scan showed that there was some blockage and my septum was crooked. Now I can't smell things like bleach and a dirty diaper, yet I have an overwhelming smell of perfume at random times, a smell no one else even standing next to me can smell. I do not wear perfume for this very reason. It's just strange for me, being able to smell some things, yet not others. It's frustrating. The strangest thing is, I wear a positive airway pressure machine at night and during some nights I can smell the strong perfume smell with my full face mask on. At times I literally have to cover my nose because the smell is so overwhelming. But why can't I smell food or the dirty diaper? That's what confuses me.

# case 0851

I suffered a traumatic brain injury six months ago. I can no longer smell scents and I cannot taste any flavors. I can taste salty, sweet, tangy and bitter, but for example, all ice cream is the same: cold and sweet. I can recognize if a smell is fruity, but all fruit-scented things smell the same, sort of like spoiled strawberries. It's very frustrating, and at times depressing.

# case 0852

I had a viral infection with flu-like symptoms earlier this year. I took antibiotics and after this bout I became aware that I could no longer smell anything. I used to cook by smell — now it's by memory.

# case 0853

For the last two years I could not smell anything. Earlier this year I then had laparoscopic surgery to remove a gallstone. After the surgery I could suddenly smell again, but it only lasted for five days.

# case 0854

For a few weeks I have this really bad smell up my nose. It's making me feel sick. The actual mucus smells when I blow my nose. I never had this before. I can smell this most of the time.

# case 0855

I had a terrible cold and lost my sense of smell and taste for about a month. It slowly came back, but I could only taste the first few bites of food and then nothing. Now some of my favorite foods taste and smell really horrible. Coffee, chocolate, peppers, raw onions, peanut butter are some of the most potent. They all have the same smell....unlike any food that I've ever smelled. It's sort of rotten, and a bit on the chemical side, not appetizing at all. The savory foods seem to have been more affected, as I can still taste fruit and sweet things. Wine still tastes good...thankfully!

# case 0856

A few months ago I developed a cold and lost my sense of smell. I saw two ear nose throat doctors but I still do not smell anything. I was a person with a beautiful and sensitive smell power, so I get frustrated with this condition many times. Even the strongest of perfumes I cannot smell.

# case 0857

It's been now two weeks since I have not been able to smell or taste food or anything else for that matter. I was found unconscious on the floor. Ever since the incident I have not been able to taste or smell anything.

# case 0858

I noticed that I can't taste or smell anything. I can smell cigarette smoke only. I cannot taste any kind of foods. I smell charcoal and my food tastes like charcoal.

# case 0859

I had a real bad cold half a year ago and ever since then I can hardly smell anything. Now I have noticed my taste has changed. I taste an awful vinegary taste in things.

# case 0860

It started when I was 23 years old. Colognes started to smell bad for me and so did alcohol. Every time I smell something that has a strong scent or odor my nose hurts.

# case 0861

About the age of 11 I had chicken pox and problems with allergies and since that time I have not been able to smell. However, there were times when I thought I could smell shampoo when sitting next to my daughter after washing her hair, or gas, when I was pumping gas. My sense of taste is okay.

# case 0862

About 15 days back I had a cold with cough and other symptoms. I overcame the cold, but a few days later I noticed that I can no longer recognize the various smells and tastes.

# case 0863

I got hit on the front of my head. I noticed after a few days that I could no longer smell my "Sniff and Scratch" books! In school our teacher found out my tongue could tell sweet and sour over the entire tongue! Once, as an adult, I smelled something best described as "shit"... We lived near a packing plant with distinct odor. This ability to "smell" only lasted a few hours. I have been unable to smell since!

# case 0864

I have had asthma for 15 years and can't smell anything.

# case 0865

I used to smell things that nobody else smelled. I received electroconvulsive therapy and I am well now and on the way to a good and prosper life.

# case 0866

A few days ago I started to smell this very sweet smell in my nose. I don't know why or where it is coming from. All I know is that I can't stand the smell and it is actually starting to make me feel sick. Sick to the point that it upsets my stomach severely.

# case 0867

I started experiencing unusual smells nobody else can smell after a severe influenza. It does happen about three to four times a day.

# case 0868

I lost my sense of smell after a concussion after head trauma.

# case 0869

Last year, at the age of 44, I had an infection of the upper respiratory tract and lost my sense of smell completely for an entire year. Slowly I regain it, but now I notice that a lot of things smell very different than they used to. The perfumes I used to use, which smelled very pleasant to me prior to anosmia, now don't smell so pleasant. Some foods don't taste as good as they used to (for example I can't stand bananas now), and other foods taste much better than they used to. Cigarette smoke smells almost minty to me, and not nearly as unpleasant as it used to. There are many examples like this.

# case 0870

Many years ago I noticed that I did not get scents from flowers. Now I can only get a few smells but not many. I have a history of sinusitis and I worked in close proximity to a histology lab, with formalin.

# case 0871

At first I noticed that if I smelled a flower or food that the smell only lasted a few seconds. As time went on I would get a "whiff" of something but the smell would be gone before I could identify it. I was in a very stressful phase of my life at that time and my doctor said the loss of smell was stress related and my sense of smell would come back when things were better... but she did not do any test. Now, over two years later, my stress level is way down but I still cannot smell most of the time. When I can, it is just for a second or two... then the smell is gone.

# case 0872

Presently, for no known reason, I can't taste anything and smell bad smells that no one else seems to notice.

# case 0873

Following a head injury three years ago at the age of 30 I lost my sense of smell. I can still taste just fine, I know if it's a shiraz or a merlot and if there is sugar in my tea. Now I have a very mild soapy smell in the back of my nose all the time. It changes on a daily basis with undertones ranging from caramel to pine needles.

# case 0874

I used to use the car air freshener and observed that I could not get the full scent of the air freshener while other occupants of the vehicle would complement me on the scent. I use the air conditioning in the car all the time. I suspect that my inhaling the air freshener all the time contributed to the gradual loss of my sense of smell. This gradually grew worse. Last year a rat died in my office. I did not detect the odor. Others who entered the office complained about the foul odor. This was embarrassing. For Christmas this year I went to buy some perfume for my wife. The clerk brought some perfumes for me to sample, I could not discern the different scents. I bought the perfume simply on the appearance of the packaging. While my sense of smell has not completely gone, I am severely handicapped by my very weak ability to detect odors.

# case 0875

Three years ago at the age of 30 I had a head trauma. After recovering and without anyone including doctors noticing, my sense of smell is completely gone. I can taste sweet and salty but everything sweet and everything salty tastes and smells the same. Ammonia and a rose would smell the same to me. It is a little weird, but at the same time I think it is fairly interesting. I now only eat to fill up and for no other reason. I am alive and happy but a sense of smell would be awesome.

# case 0876

I used nasal sprays for nasal allergies for a few days and now I can't smell anything.

# case 0877

I started losing my sense of smell when I started having problems with a bone growth pressing on my eyes. Since then I have had two accidents to my head and have lost a lot of my sense of smell and some of my sense of taste. It is getting worse as I get older.

# case 0878

About six months ago I got a bad sinus infection. Since then I am experiencing a terrible smell and it is getting worse. At first it smelled like burnt oil. Then it changed to sickening flowery smells. These changed in severity and are worse now that they were before. I also have ringing in my ears since all this happened. I saw an ear, nose and throat doctor and he said I have a deviated septum. I don't know...

# case 0879

I noticed earlier this year that my senses of taste and smell come and go but are more often than not absent. I am not sure if it is due to an allergy such as hay fever or my sinuses, which seem to be permanently blocked.

# case 0880

I am not entirely sure if I lost my sense of smell, but the smells seem to smell different from which they used to smell.

# case 0881

I was handling old moth balls and thought the odor was gone while others were overwhelmed by the remaining scent. I've also noticed a lessened ability to taste that seems to be related to smell. I saw a recommendation on TV for a chemical that will help remedy this lost sense. That idea is interesting to me.

# case 0882

I smell paint when there is no paint. I thought it would go away but so far it hasn't. It is some times stronger than others. I have no idea what is causing it.

# case 0883

I do not recall ever having a sense of smell. When I was little, others would complain about some smell, and I wouldn't react at all, except with some embarrassment at not being able to perceive something that apparently everyone else could detect. This perhaps happened when I was very young and fell out of a tree, but since I do not recall having a sense of smell, it could have happened earlier, or it could be congenital. In general, the anosmia is not a problem, but people just don't know how to react when they offer me something to smell or comment on a smell, and I explain my condition. I often fake it or avoid the smell conversations, especially if I will not see the person again or am not close to them. I used to teach writing classes and I made this into an interesting assignment. I explained my condition, and then had my students explain a smell in writing.

# case 0884

I have been anosmic from birth. It never bothered me and my sense of taste is normal (I hope!).

# case 0885

I have been losing my sense of smell over the last five years. I have nasal polyps on both sides. Five months ago I could smell but now I can't smell any type of smell again.

# case 0886

I gradually lost my sense of smell over the last six years or so. For at least three years I have had no ability to smell whatsoever.

# case 0887

Three years ago I was seated at my computer when I began to smell a strange sort of burning smell. It seemed chemical in nature and at first I ignored it. Later, when the smell persisted I looked around me thinking that "something" was hot and giving off the odor. There was a little teddy bear sitting on the modem and I quickly removed it thinking that the heat from the box was causing the fabric to stink. That did nothing to stop the odor. I gave up trying to find the source and told myself that it would go away. The next day the smell was still there and it has changed character over the three years since. Sometimes it is perfumery, sometimes it is more like rotten cheese. It is not really smoky as it was briefly at first. It's usually a chemical type smell. I perceive odors which should be appetizing as very bad ones. The odor of sautéing peppers and onions is nauseating to me, where once it was delicious. All foods do not smell the same as before and do not taste the same either. Coffee is not the satisfying beverage it used to be. I still drink it but do not enjoy it. When I have cooked a dish I cannot taste if it is good or not. So I perceive odors differently now and taste them differently, too. I went to an ear nose throat doctor who told me that my sinuses were normal. I went to a neurologist who suggested I take antidepressants because I must be depressed. I left his office thoroughly disgusted. So...I just go on trying to not think about it. A couple of months ago it seemed that the "stinks," as I call them, were fading a bit. But...lately they have come back. I have nasty, crusty boogers in my nose almost all the time now and the interior of my nose is often bloody. I have read that sometimes this condition eventually goes away so am just waiting and hoping.

# case 0888

Ten months ago I fell on the back of my head. It wasn't until a few days later when I was "smelling" perfume that I realized I could not smell anything. I tried smelling food, candles, laundry detergent and a variety of other household items before it really sank in that I could not smell a thing. I was always very aware of smells, and loved both pleasant and earthy smells. I quickly realized the dangers of not being able to smell. I realized the dangers of spoiled food and the embarrassment of stinky garbage. I sunk into a depression about my lack of smell. I learned online that there was a name for it. I had never heard of the name or the condition before. I just "survived" my first holiday season without enjoying the smell of Thanksgiving turkey, and the wood burning fire at Christmas. I so miss the smell of breakfast, dining out and good wine. I have no real interest in food, although my waistline has grown trying to find an answer to the insatiable craving of a food that will bring back my sense of smell and taste. I'm relieved to know I'm not alone, but I realize what an invisible handicap I've acquired. What a drag! I have since the accident acquired some sort of smell, but it is indescribable. It's not good or bad, sweet or bitter, it's not chemical or organic, it's just there. It's amplified in a restaurant or a place that would obviously emit many smells. Honestly, I wish I had no smell as opposed to this mystery scent. Things I used to love, peanut butter, coffee, chocolate, spicy food... are hardly tolerable. I avoid peanut butter and chocolate altogether. It's much more difficult to deal with than I'd have ever imagined.

# case 0889

I was devastated when I lost my sense of smell at age 68. When I first apply perfume I can smell it in the air but only for seconds and I can't smell anything else. I developed an ear infection about the same time I lost my sense of smell and I now think the two are connected. It's so miserable not being able to smell and whilst there are times when I'm glad I cannot smell things, I often feel left out not being to hold a new baby and get that lovely smell, or not being able the smell grass cuttings or my favorite food being cooked.

# case 0890

An outbreak of the shingles resulted in the loss of my sense of taste and smell. I got a life-changing diagnosis of anosmia. Through a neurogenesis-promoting approach including tai chi, diet, and mindfulness I can now "sense" most smells. A good example is that I can feel the smell of chlorine on my skin.

# case 0891

I'm suffering from this problem since the age of four: I can't recognize any kind of perfumes, or petrol, or any other smell. I have problems with colds and cough the whole year.

# case 0892

I suffered from sinusitis many years ago. They found that I had a polyp. I was prescribed nasal spray and over a period of time I noticed that I could not smell as well anymore. When I was in a garden, where everyone commented on the lovely aroma from flowers, I had to smell the flowers close up to get any smell. I get smells of something and then it just disappears; it's weird and difficult to describe. I have this problem and live with it, as the alternative is surgery, but polyps grow back, so what's that point? I live with it and just have to get close to the smell.

# case 0893

For a long time sometimes I smell something which is not there. Also, my sense of smell is blocked.

# case 0894

I never remember ever being able to smell but my mother says that I did. Oddly, I can taste and I love to cook and I love food and wine.

# case 0895

I suffered a head injury and lost my sense of smell two years ago. Within a month I started to get phantom smells which ranged from the smell of the tumble dryer to oil and petrol. Six months after the accident, I started to pick up certain smells but they were inaccurate. I started to smell coffee but not like I used to smell it. Within weeks I was getting the same smell for cigarette smoke. I still get these smells — also for chocolate. The smell is a sort of burning smell but not too unpleasant. I am able to drink coffee now but cannot eat chocolate as the taste is so bad. Many other smells like peppers, celery, and perfumes also smell different than they used to smell. Eggs and certain other foods smell like urine. I get phantom smells first thing in the morning and last thing at night when I am tired or stressed. It is a sweet and overpowering smell.

# case 0896

One day I realized that I can't smell my daughter's soiled diaper. One month later I still wasn't able to smell it. I have no diagnosed diseases but I am not feeling well.

# case 0897

I am unsure when I lost my sense of smell, but I only started paying attention to it when one day someone aggressively accused me of ignoring the burning meal on the stove on purpose. I then started to smell things such as fruit, herbs, perfume, bleach... and realized that I could not smell them. I went to an ear nose throat doctor and he gave me a nasal spray. It did not work and I felt he did not take me seriously. I decided to just live with my smell loss.

# case 0898

I am a mother of three children and I have a problem with my nose. Since five years back I have a blocked nose and cannot smell anything. Only sometimes can I smell. I hope I don't get nose cancer.

# case 0899

I now find it almost impossible to detect smells such that they are are unidentifiable and very difficult to describe . Different surroundings produce different sensations but essentially the sensations can best be described as mildly chlorine chemical smells . My appetite is very poor with very little interest in food even to the point of nausea.

# case 0900

I lost most of my sense of taste first; some things, i.e. lemons, tomatoes, anything tart, tasted really bad. I had a concussion and a few months later, my sense of smell totally disappeared. I have been taking zinc supplements and my sense of taste seems to be recovering somewhat, but no change in the sense of smell.

# case 0901

I've never been able to smell.

# case 0902

Loss of smell — accompanied by phantosmia —came about ten months after I tried to eliminate a bacterial infection in my nasal passages with antibiotics. The bacterial infection is now gone, but I have had no sense of smell, along with a strong phantom stink in my nose. This has been a huge challenge to my quality of life.

# case 0903

I was born without a sense of smell... No one knows the cause. They believe my olfactory bulb is not functioning properly.

# case 0904

After a head trauma I lost my sense of smell.

# case 0905

Since four years ago I have no sense of smell at all. Ear nose throat doctors confirmed that I have no sense of smell and taste, but they have no remedy.

# case 0906

A few months ago I started smelling a metal smell. I noticed, though, that it was in my nose, not in the air. This lasted for a couple of months, then stopped, but has now returned again. I have always had a strong sense of smell, and am now wondering if this is normal as we age.

# case 0907

I lose my sense of smell and taste whenever I take acetaminophen of any kind for three days or more. I was recently switched from codeine medication for pain to acetaminophen. Within a week I could no longer smell or taste anything. My tongue still distinguishes between the basic salt, sweet, sour and bitter but other than that I cannot tell raspberry jam from peanut butter (except for the texture of course). It is very annoying and I would love to find an alternative to the acetaminophen.

# case 0908

A few weeks ago I noticed a sharp smell in my nose and got dizzy. It only lasted for a few seconds but it keeps coming back more often now.

# case 0909

I had diabetes for three years. At the same time the diabetes started I also lost my sense of smell.

# case 0910

I have allergies and as a consequence I often had problems smelling. Then, three years ago, my sense of smell was completely gone. No treatment helped but it has started to spontaneously come back occasionally.

# case 0911

I woke up one morning and immediately I could not stand the following smells and tastes: coffee, chocolates, all meat except processed meats like salami, peaches, watermelons, etc. Now my life is hell. To go to a restaurant is not pleasant and I have to plug my nostrils to block the various smells.

# case 0912

A head trauma at the age of 17 resulted in a concussion. I was in the hospital for a while, eating hospital food, and never noticed anything. I then asked parents to bring a hamburger (which I used to like) but when I ate it, it tasted like cardboard. That was first indication something was wrong. Later my doctor informed me of correlation between concussions and anosmia. It didn't bother me for over ten years. But in my late 30s it became more and more of an issue. I noticed a lack of strong memory for anything after the age of 17. It also has led to reduced feelings of intimacy with my partners. When I was 16 I had a girlfriend, and when she was away for stretches of time I was very cognizant of the way she smelled and would imagine it while on phone with her. I've never had that and the ensuing emotional attachment with anyone after the head trauma. I also have phantom smells, of course, like a missing limb. I'm very prone to suggestion.

# case 0913

After many years of constant sinus infections and congestion I lost my smell at the age of 30. I also have asthma and nasal polyps. At one point I suddenly started getting severe blockage and lots of blood when blowing my nose and my sense of smell vanished. Since then I have no sense of smell except for short periods when I'm treated with prednisone which used to help get my sense of smell back. Along with my sense of smell I also lost my sense of taste. I love to cook and my life has always involved cooking and tasting. Now I cook from memory. Salty, sweet, spicy, or sour are now the only tastes I have to go on and this kills me. I work with chemicals and have to be careful to avoid overexposure to solvents and other volatiles. I think the labeling of anosmia as a low risk disability is rather absurd. I never get the rush of memories anymore when that pot of sauce is simmering, I don't get that endorphin rush when I smell that certain perfume on a woman. I never know if there are bad fumes in the air. One time I almost passed out do to using a mislabeled bottle of solvent. I come across many things every day that have a huge effect on my body and brain that I didn't know about before losing my sense of smell. Every day is just bland without smells.

# case 0914

I am a lifeguard and part of my job is to put chlorine in the swimming pool. Seven months into this job I lost my sense of smell and I suspect it is because of the chlorine.

# case 0915

I noticed about four years ago that I couldn't smell most things. I only could faintly smell some perfume odors if they were placed on my hand and I placed my hand under my nose. Even strong odors like coffee or gasoline I can hardly smell.

# case 0916

I experienced a change in my sense of smell. Coffee, cigarette smoke, and car exhaust all smell alike. I can't taste chocolate. Shampoo and soaps smell weird. Aromatic hydrocarbons smell somewhat like pepper mixed with rust dust.

# case 0917

Over the last couple of years I've noticed that upon being exposed to cigarette smoke, I have a mysterious reaction that lasts two to three weeks. I have a persistent sensation of smelling cigar smoke. I am not a smoker and don't normally expose myself to smoky environments. It's a constant background scent, and often causes me to think that I'm actually smelling a real odor. The first time it happened I was convinced the odor was something lingering in my clothes or some sort of horribly persistent body odor that wouldn't wash off. This began after I had been to a house where people were smoking on the porch and there was a noticeable smoke odor in the house. The next day was when the cigar smoke scent began to bother me. No one had been smoking cigars, only cigarettes, so the odor is related yet different. Over the last two years I've had incidents like this occur about five times. I used to smoke but quit several decades ago. For the past ten years or so I have suffered fairly constant rhinitis and post nasal drip, persistent but mild. I know that my sense of smell is diminished compared to when I was younger.

# case 0918

Gradually, over the last five years, I have lost my sense of smell of coffee, fresh flowers, perfumes, and food cooking on the stove. I cannot even smell smoke from an open fire at a barbeque. I have gone to an ear nose throat doctor but I am still unable to smell anything.

# case 0919

Two years ago I had an accident and injured my head. During treatment I noticed that I had lost my sense of smell. There were no other lasting effects. My doctor told me that my sense of smell may return in the future and he suggested yoga and pranayama. There was no improvement. I now have no sense of smell for two years and I am worried about it. Travelling and cooking has become very difficult for me.

# case 0920

For the last eight years I cannot sense any smells. My doctor told me that it is because of a small tumor in my nose. I'm taking medicine that brings back my sense of smell, but as soon as I stop taking the medicine, I lose my sense of smell again.

# case 0921

About four years ago I started losing my sense of smell. Now I have no smell at all. I have been diagnosed with a spur in the left nostril and polyps.

# case 0922

The first time I realized that there was something wrong with my sense of smell was somebody could smell gas from the stove and I couldn't. Since then I noticed that I cannot smell coffee brewing, bacon cooking, or anything else for that matter. Now I live alone and have to triple check all the gas jets as often as needed. I have had two ear nose throat specialists look into the problem. I now go through life not able to smell anything.

# case 0923

I always had a somewhat poor sense of smell. Last week I noticed, when trying to catch a whiff of citronella fragrance from a bottle, that I was barely able to catch the scent.

# case 0924

I tend to associate my gradual loss of my sense of smell with the dental surgery that I had about two years ago. Soon after the surgery I began to have problems with mouth ulcers and a saliva gland became blocked. These problems gradually eased following a range of treatments, but I began to notice a deterioration in my sense of smell. I did nothing about this as it didn't particularly trouble me at the time and I believed it would resolve itself eventually. It didn't and after about a year I decided to consult my doctor who referred me to a specialist. Nothing was found to be wrong and so no further help was offered. I have a permanent odor in the nose which is extremely difficult to describe and varies in strength at no particular intervals. I can only detect certain types of strong odors such as menthol. My taste buds are also impaired. This condition shows no sign of improving and I'm becoming extremely distressed and depressed. The loss of olfactory perception is affecting the quality of my life and I'm desperate to find a new source of treatment that might restore normality.

# case 0925

At the age of 12 I learned that something is completely wrong with me. It was not until then that I discovered that I couldn't smell. Today, I am 21 years old and until now the only thing I figured out is that this "illness" has an actual name: "anosmia". Since I was born with congenital anosmia there is no help for me. I tried several things, have seen millions of doctors and specialists and even had surgery last year. But nothing helps. I have to live with that. But it is hard and unfortunately society doesn't really know what the loss of smell means for us affected people. I can't smell coffee, I can't smell perfume, I can't smell my own body and I will never be able to smell my own children. Sometimes, this feeling of being left out in a world that is totally focused on smell-related things makes me want to cry. I feel like an outsider. But since you can't see my illness nobody thinks about it and everybody forgets. So people keep asking me how I like their new perfume. Sometimes I just lie because telling my story makes me feel even worse and I don't want pity, I don't want to respond to the same questions over and over again, and I don't want the questioning looks in people's eyes starring in disbelief. Due to anosmia, my world is smaller, darker and sad sometimes. At times I have difficulties meeting new people because I am so unsure about myself, how I smell and how they will think I smell. It is a vicious circle you can't get out of. And it's hard to trust people and to love myself. My greatest wish (obviously right after being able to smell) is that society will know more about anosmia and that they are aware of us and that there is a broader support for affected people.

# case 0926

I had a cold that lasted for about 25 days, during which I could not smell anything which in the circumstances (blocked nose) I thought was only natural and expected. What got me worried was that even after the cold had subsided, I was unable to smell anything. I realized a little later that I had also lost the sense of taste — I could only experience bitter, sweet, and sour tastes. Loss of the sense of smell at such a young age was something I had not heard of, although I did know that at an advanced age a diminished sense of smell is to be expected. I visited a specialist. I had no difficulty breathing, yet he told me that my nose was blocked and prescribed a nasal spray. There was no improvement whatsoever and I was referred to a neurologist.

# case 0927

Six and a half months ago I fell and cracked my skull. I spent two weeks in the hospital before I left for rehab. Shopping toiletries for rehab, I noticed that all the body lotions smelled the same — like nothing. I had an eerie feeling, went home and stuck my nose in a bag of coffee: nothing. To this day nothing. Only phantom smells that usually last one or two weeks, changing from plastic-like to sickeningly sweet to soda bread. Sometimes I think I can taste things properly, but it's only things of which I know how they should taste. I can't determine anything I don't know, and I can't distinguish anything blind-folded or at the doctor's. I feel I can taste better somehow over the last months, but still nothing happens when I stick my nose in a bag of coffee or anywhere else.

# case 0928

I was born without a sense of smell and it frustrates me because I do not know even the smell of my perfume. Only close family members know about my situation. I don't really discuss the problem with anyone because people don't believe that someone can be born without a sense of smell.

# case 0929

I have nasal allergies and use medications to control them. A month ago I noticed that I can't smell anything anymore and that I have a bad taste in my mouth. It makes me very sad. I have seen a doctor for it, but there was no progress. This puts me in a very bad mood.

# case 0930

I got a very bad upper respiratory infection and was given antibiotics, zinc, and a steroid nasal spray. When I first noticed I couldn't smell or taste anything, I thought it was normal. However, after three months I became worried and saw my doctor about it. He tested me by having me close my eyes and try to smell what I believe was fainting salt. I then saw a specialist and was checked for polyps and a tumor. From then on any doctor I saw didn't really want to talk about it because there's "nothing they can do about it". For the last six years my life has been very strange and depressing and I have a serious feeling of gloom and doom. I know I breath in things I would otherwise run from i.e. cleaners, smoke, etc. I used to love to cook and now it makes me feel vulnerable. The list of the ramifications of anosmia in my life is almost too long to spell out. I drag the thought of having no sense of smell around with me every minute of every day. I feel it is as great a disability as blindness for instance. I've sort of lost my will to live yet I wouldn't hurt myself due to the fact that it would hurt the people I love. My life as I knew it is gone forever.

# case 0931

I woke up one morning 26 years ago and noticed that something was wrong. Among other problems, my eyes were blurry, and my sense of taste and sense of smell were off. I found little information about my condition and the information I found scared me. I was given medicine for a stuffy nose but have never been satisfactorily treated. Now I'm disabled. I still have sense of smell problems. I miss the way I could detect odors.

# case 0932

I believe that my olfactory senses were overstimulated while working in a laboratory. Now, I immediately smell any chemical smell. It overwhelms me, I get an instant headache. It really limits my life. I cannot go into a movie theater or hair salon because the odors are so overwhelming.

# case 0933

I currently suffer from anosmia as a result from a fall and serious hit to the back of my head. Approximately five days after the fall I realized I had no sense of smell. I could absolutely smell nothing. In the 10 months since I've noticed that I do indeed smell something, but it is indescribable. It doesn't smell like anything familiar. Based on the strength of the smell of the environment around me, the intensity of the smell varies. If I walk into a coffee shop or restaurant the unpleasant smell I experience is magnified. As it would be if I stood beside someone smoking. I cannot discern good smells from bad. It's just varying degrees of this unpleasant smell that I now experience. I am battling depression because of my lack of smells and tastes.

# case 0934

Nearly a year ago I was on vacation. The smell of lavender incense was pervasive everywhere — sometimes pleasant, sometimes overbearing. I could smell it all the time. After we returned home I would notice the smell at different times of the day and at different places. Sometimes it was faint other times very strong. When it is particularly strong, I have asked others if they could smell it and they couldn't. I checked everywhere for a source but it is winter here and no lavender would be blooming anywhere. This doesn't occur all the time but often. I can't find any explanation why I smell it sometimes but not others although I think I notice it more in the evening and when I am relaxing.

# case 0935

Around the time I graduated from college I noticed that I was no longer able to detect aromas or smells as well as I used to. I first started noticing it when I was cooking and wasn't able to differentiate between different smells as well as before.

# case 0936

Five months ago I developed a dislike for yogurt. The smell of yogurt in my mouth triggers a sensation of vomiting. Both the smell and taste are kind of funny. Ever since this malady I have lost my appetite. The perfumes that I loved to wear produce the same uncomfortable smell.

# case 0937

After two infections of the upper respiratory tract in quick succession my sense of smell faded over a period of two weeks. It didn't disappear immediately, but I noticed that smells became less strong, until eventually I couldn't smell any aromas at all. That was just under three years ago, and since then I have been unable to distinguish any aromas at all — even really strong ones such as eucalyptus or wintergreen. Although I don't believe that I have allergies, and I don't experience any other symptoms of sinusitis or colds, I continually blow my nose. It gets very blocked if I lie on my front, and often during the day or night I will find that I can't breathe through my nose.

# case 0938

Around the time I moved and had my first child about a year ago I have lost my sense of smell. I have been to my primary doctor and a specialist and the treatment helped with the sinus congestion but not with my sense of smell.

# case 0939

I had anosmia since I can remember and it's not always easy, but I've learned to deal with it and accept it. Is particularly bad for me that I have a reduced sense of taste and that I cannot smell my own body odor.

# case 0940

Since I was born I cannot smell anything.

# case 0941

It was two years ago when I fell into depression. I love perfumes and I always used to buy different fragrances and I could recognize always a perfume that I wore in the past and I have worn more than a hundred until today. But the time I was sick I could not smell or sniff all my favorite fragrances. Every scent seemed to be indifferent and sometimes bothering or disgusting. Fortunately now I' m recovered and I find again pleasure and joy in sniffing new fragrances and wear everyday my favorite perfumes.

# case 0942

I lost my sense of smell when I was about 19 years old. I had just finished working in a laboratory with chemicals such as formaldehyde, pyridine, etc. for half a year. I had taken a few knocks to my head a few years prior, but still had a sense of smell afterwards. I can still smell some unpleasant odors, but not pleasant ones. My doctor treated me as a waste of his time, and was very rude — I haven't mentioned my problem since.

# case 0943

I've suffered from allergies and sinus issues for many years. About two and a half years ago I started having pain in the right side of my face. Now I smell fumes. This comes and goes. I have had the gas service out on three different occasions to check for any gas leaks — there were none. This is driving me crazy. Sometimes it lasts for weeks, then it will clear. Right now I feel like I'm sitting next to a running vehicle in a confined space. It's awful and I'm miserable.

# case 0944

When I drank more than six or seven alcoholic drinks I couldn't smell anything the next day. Now I don't drink anymore, but the condition worsened. Now I have no sense of smell at all. It returns for short moments once or twice a month, though.

# case 0945

I had hay fever and my sense of smell came and went. For the last two years I can't smell anything. I feel very frustrated.

# case 0946

Recently, I have been getting up at three in the morning to a very strong mint/toothpaste smell. It lasts for at least an hour. This morning I even checked outside to make sure it was not coming from outside. Now I know it isn't outside but in my room.

# case 0947

I cannot smell, but don't know why. I painted houses and rooms without a mask many times and suspect that this may be the reason why I lost my sense of smell.

# case 0948

I cannot smell food or any other odors. If I put lotion on, I can smell something but cannot tell what it is. I cannot smell sour food, gas, or any foul odors. I cannot smell flowers.

# case 0949

I keep smelling lead.

# case 0950

Eight months ago I got sick with bronchitis and sinusitis. I took an antibiotic and got over it. A short time later I was driving in a car when someone with me said "We must have passed a skunk, it smells so strong!" I didn't smell it at all!!! So I went to an ear nose and throat doctor. I then went to a neurologist who had me sniff coffee and rubbing alcohol, which I could not smell. I also had spray painted some wicker baskets in the backyard and bleached some clothes but smelled neither. At Christmas time I could not smell the wood burning in the fireplace nor smell the tree or the fresh greenery. I can taste sweet, sour, bitter & salty to a point.

# case 0951

I had a head injury from a fall. After a while I noticed smells in the nostrils that were not there, almost like a gravy smell, but odd... not pleasant. Tastes changed mostly with anything fried and with oils and butter. Even chocolate, which I used to love tastes like fried food. Foods that are spicy or fruity taste fine.

# case 0952

I have a bad smell in my nose all the time, like bad potatoes. It started while I was on holiday. I have headaches with it and my eyes look puffy.

# case 0953

For two weeks now I have had a runny nose. It usually is congested but even when my nose is clear, I cannot smell anything. I only can sense salty tastes. Food seems tasteless. I am quite worried that I could permanently lose my sense of smell and taste.

# case 0954

I have anosmia because of nasal polyps.

# case 0955

I had pneumonia and shortness of breath and started using inhalers. I have no sense of smell at all except every few months I have a flash of smell for half a minute.

# case 0956

I have noticed a loss of my sense of taste and smell. I can only taste sweet, salt, bitter, and sour. Around the same time the smell of my favorite perfumes started getting weaker and weaker... I no longer can taste flavors like ginger or curry.

# case 0957

I lost my sense of taste and smell the week I had a turbinectomy. My doctor said he never heard of it.

# case 0958

I was born without a sense of smell. When I was about twenty years old I developed polyps which were surgically removed. I thought the doctors could find my lost sense of smell, but they did not. It embarrasses me when I am around very bad smells that I cannot smell but others can.

# case 0959

I have nasal allergies. I began receiving treatment for it when I was 27 years old. Now I have been about ten years (since age 47) without allergies thanks to the treatment. At age 35 I started losing my sense of smell. Now I have barely any sense of smell.

# case 0960

For two months now I smell a chemical smell. I have had this problem every year before winter for the last five years.

# case 0961

Thirty years ago I underwent uvulopalatopharyngoplasty and had polyps removed. After the surgery I had lost my sense of smell and taste and the feeling in my teeth. After a few years, my sense of taste and the feeling in my teeth returned, but I never was able to smell again. I am 78 years old now and worry about eating spoiled food or being in a fire.

# case 0962

About six months ago I noticed strange smells all the time that no one else would smell. I seem to relate the smells to hot plastic for some reason. During this same period of time I also got infected with methicillin-resistant Staphylococcus aureus. I was put on tetracycline and sulfur. After the treatment the smells were gone.

# case 0963

The problem started when I thought I could smell smoke or something burning. Other times I can smell oranges even though there are none in the house. Another problem was smelling and "tasting" ammonia. I just kind of live with these weird smells as when I ask if anyone else can smell anything they say "no".

# case 0964

Three years ago I had a number of viral infections over the winter. In spring I noticed that my sense of smell had disappeared. A little later I was diagnosed with nasal rhinitis and, a little later still, asthma. I take corticosteroids for these conditions. I still get "glimpses" of smell for about half an hour after I have been on a fairly vigorous run or when I am in the mountains. This is all very frustrating because my doctors can't seem to help me capture my sense of smell permanently?

# case 0965

I don't remember ever being able to smell things, unless they are super strong in which case I can faintly smell them. My mom read on the internet that hitting the dashboard during a car crash can cause anosmia and when I was three or four years old that happened to me so now she says that this is probably why I can't smell. I don't really care but my mom always tells me that it is bad and that I am missing out on a lot of wonderful smells. She also tells me that I stink sometimes.

# case 0966

Since the age of 30 I suffer from a smell and taste disorder. My doctor told me that the cause of my problems are polyps and that surgery would be necessary to correct the problem.

# case 0967

About four weeks ago I noticed that I could no longer smell food cooking or my wife's perfume. I didn't think much about it at first but now I start to get worried.

# case 0968

I feel there is some spirit in my house and sometimes I feel a foul smell like toilet in my house. The smell disappears with time.

# case 0969

For the past week I have had the overwhelming feeling that there is cigarette smoke. I checked my home, the attics, sheds and could find nothing that would alarm me to a fire. My husband checked everything and does not smell the smoke. I have gone to several stores and the smell is following me everywhere, it has also taken away my sense of taste and is causing my head to pound continuously.

# case 0970

I lost my sense of smell as the consequence of an accident.

# case 0971

Five months ago I suffered a slight fracture and mild concussion after falling on the back of my head. Three months after the accident I noticed that my sense of smell was lessening, until it had completely disappeared. Along the way I have also lost much of my sense of taste.

# case 0972

34 years ago I lost my sense of smell when living in a very dusty house. I still can't smell anything. Just once in a while I might enter, say, a store selling potpourri and the perfume would be so strong that I would briefly smell it.

# case 0973

For the past three to four years I cannot differentiate among smells. I can only tell if people fart if I hear the sound.

# case 0974

I have always had a very keen sense of smell and taste. I always smell everything before anyone else and can identify specifics. Since two or three months ago, I am smelling cigarette smoke around me. Not just the smoke, but the lack of air that you feel in a smoky room. It makes me feel like I cannot get enough air. I was worried there may be a carbon monoxide or other problems in the house and placed carbon monoxide detectors on each floor. No one else smells this. Can you hallucinate smells?

# case 0975

Sometimes I do smell while most of the time I don't. I don't know what caused this, maybe my smoking.

# case 0976

I have chronic sinusitis and asthma. As a result my sense of smell has been weakening gradually. I still have a relatively normal sense of smell but it is not as keen as it used to be.

# case 0977

I noticed recently that I've lost my sense of smell. I won't know if I am hungry or full. I can't even smell the food that I'm eating and once I've eaten I don't know whether I am full or still hungry.

# case 0978

I have been suffering from anosmia for 20 years. I have growths in my nose and need surgery.

# case 0979

Five months ago I had a flu which was severe enough to have me in bed for five days. It took seven weeks after these five days before I felt normal again. It was during the five days in bed that I noticed that my smell was not as it used to be. It didn't return to normal and now I cannot even smell a rotten egg! My taste doesn't seem to be affected to any great deal.

# case 0980

I smell celery in all foods.

# case 0981

I suffered traumatic brain injury as the consequence of an accident. After being released from the trauma facility, I became aware that I lost my sense of taste. The only thing I can taste is salt and sweet. I have also been unable to detect hunger since that time. My sugar levels can dip to 49 before I become aware of the condition. This is problematic because I have diabetes.

# case 0982

I started experiencing the intense smell of fuel oil about two weeks ago after passing a field which may have been sprayed with fertilizer. The fuel oil smell has lingered sometimes intense and other times not so intense. No matter what I do to squelch the fuel oil smell, it lingers. I have tried perfumes, scented sprays, etc. but nothing seems to work. I read about odor disorders and am considering going to see my physician but it sounds so weird to have this problem I am a little reluctant to go. I might just wait it out and see if it will eventually go away.

# case 0983

I noticed that I couldn't smell urine, feces, even onions after I fell and hit my head.

# case 0984

I cannot enjoy the smell of flowers like I used to when I was young. Some people tell me my breathe smells, but I do not notice it. Aroma from other nice flavors is not for me anymore. Is there any way I can get my sense of smell back?

# case 0985

I have no ability to detect odor, not even the strongest odors such as gasoline and ammonia. I have no recollection of ever being able to smell. When I was young I thought people were making up this thing they referred to as "smell". When I was in high school, I thought maybe smell was one of those things you need to learn how to do. No physical exams test for smell so it is a handicap (I have walked into a room filled with gas and failed to detect it) that goes unspoken.

# case 0986

Ever since I was a child I cannot smell at all!

# case 0987

My doctor has been changing my medications and since then I have this sickening, bitter smell that only I smell. It never goes away. It is so strong that sometimes I want to scream.

# case 0988

I was put on blood pressure medication. This is when I discovered that I had no sense of smell. I no longer take the medication but my sense of smell has not returned.

# case 0989

I don't know when exactly I realized this, but I have never been able to smell well. I can smell sometimes, but only things that are really strong or anything citrus. Even if I put something right up to my nose, I usually can't smell even a hint of anything. I would love to figure out what is wrong with me.

# case 0990

I have never been able to smell except for vinegar, smelling salts, dough improver, raw onions when cut, and really strong chemical smells that hurt my nose... Most of the time I don't even remember to smell so I won't pick up a smell unless someone asked me to smell. For me to smell it, it would have to be right up against my nose. I can smell better in steam, but my sense of smell is very very poor indeed.

# case 0991

My sense of smell comes and goes as the sun rises, some days I can't smell anything and some days I'm super sensitive. All my life I have had nasal drip and chronic sore throat.

# case 0992

I lost my sense of smell and taste just before my finals of a three year year aroma & holistic therapy course.

# case 0993

I'm a man of 21 years and I've observed that my sense of smell is not working. I had the flu but now the flu symptoms are gone and I still have no sense of smell.

# case 0994

I frequently suffer from sinus infections. Following a severe infection, lasting about two months, I noticed a reduced sense of smell. My doctor diagnosed sinusitis and started treatment but I haven't noticed an increase in my sense of smell.

# case 0995

Fifteen years ago when I was 53, I noticed that I could not smell the flowers and over time I now can't smell much of anything. I miss the smells of coffee, bacon, and fried onions. It has also affected my taste buds. I cook mostly on memory and my husband tells me I'm a good cook, but if I was to not see what is on my plate, I would do a poor job identifying what I am eating, except for the textures of food. I was making oatmeal and cinnamon for my breakfast one morning and not until I was finishing my oatmeal, did I notice I had the paprika sitting on the kitchen counter and not the cinnamon. I could not tell the difference.

# case 0996

I had an anxiety attack. About six months later I realized that my smelling power has worsened and now I can't smell anything.

# case 0997

I travelled abroad about 15 years ago. What hit me when I travelled was the smell of the cities, they were so different. They had a sharp pungency which I felt I could not get rid of even a week after I returned. The smell was so overpowering that I used to experience flashes of the smell like an action replay even after my return home. Simultaneously around the same time I notice that my sense of smell was getting fainter and fainter. I hate the smell of petrol fumes during peak hour traffic. In fact, the smell makes me want to puke. I remember being thankful for my decreased sense of smell for this reason. Then over a period of a year or so, I realized that I had totally lost my sense of smell and what was left behind was the occasional replay of the pungent petrol smell which I first felt so strongly when I travelled abroad. The memory of smell used to help me smell for a while. I saw a cake and I knew how it would smell and then I used to feel a faint twinge of the smell. Slowly over a period of time I have lost that too. I really do not miss it except for the memories of the smell of rain, shoe polish, anything that would really bring back old childhood memories. Interestingly, a friend told me that she had also been travelling abroad when she came back she found to her great distress that she had lost her sense of smell. She is a great cook and absolutely loves cooking and she was in tears when she told me that she used to cook by the sense of her smell. She says she has lost both her sense of smell and taste and she was so so distressed and has been going from one doctor to another desperately. I still remember my surprise when I heard that. I luckily somehow have retained my sense of taste and I can still taste good food and don't really miss my sense of smell; the bad odors so much outnumber the good ones. Another interesting thing is though I can't smell a thing, if I walk into a room that has been freshly painted or someone nearby has been using a particularly strong perfume, I can't smell it but I still get a headache.

# case 0998

I was in an accident and suffered skull fractures. Afterwards I was never able to smell again. At first I thought I was smelling urine everywhere, all the time. That went away after a while and I couldn't smell anything. For years I still had smell "memory", but now that's gone too. I realized fairly quickly how much smells had affected my mood. After my loss of sense of smell I would conjure up memories of certain smells, like the smells of Christmas, when it was Christmas time. I can no longer conjure those smells.

# case 0999

15 years ago I had a very bad flu, which caused hyposmia and then partial anosmia. My olfaction never came back. My life has changed a lot, I divorced, lost my job... It is very difficult for me now to make plans, feel desire, feel good and happy. I live in a permanent present, I have lost the sensations linked to memories, I have no particular desire for the future....

# case 1000

Since I was born, I have never perceived any smell except choking smell.
