# Supplementary figures and images for: Hidden consequences of olfactory dysfunction: a patient report series
Source: BMC Ear Nose Throat Disord. 2013 Jul 23;13:8. doi: 10.1186/1472-6815-13-8 (PMC3733708; doi:10.1186/1472-6815-13-8)

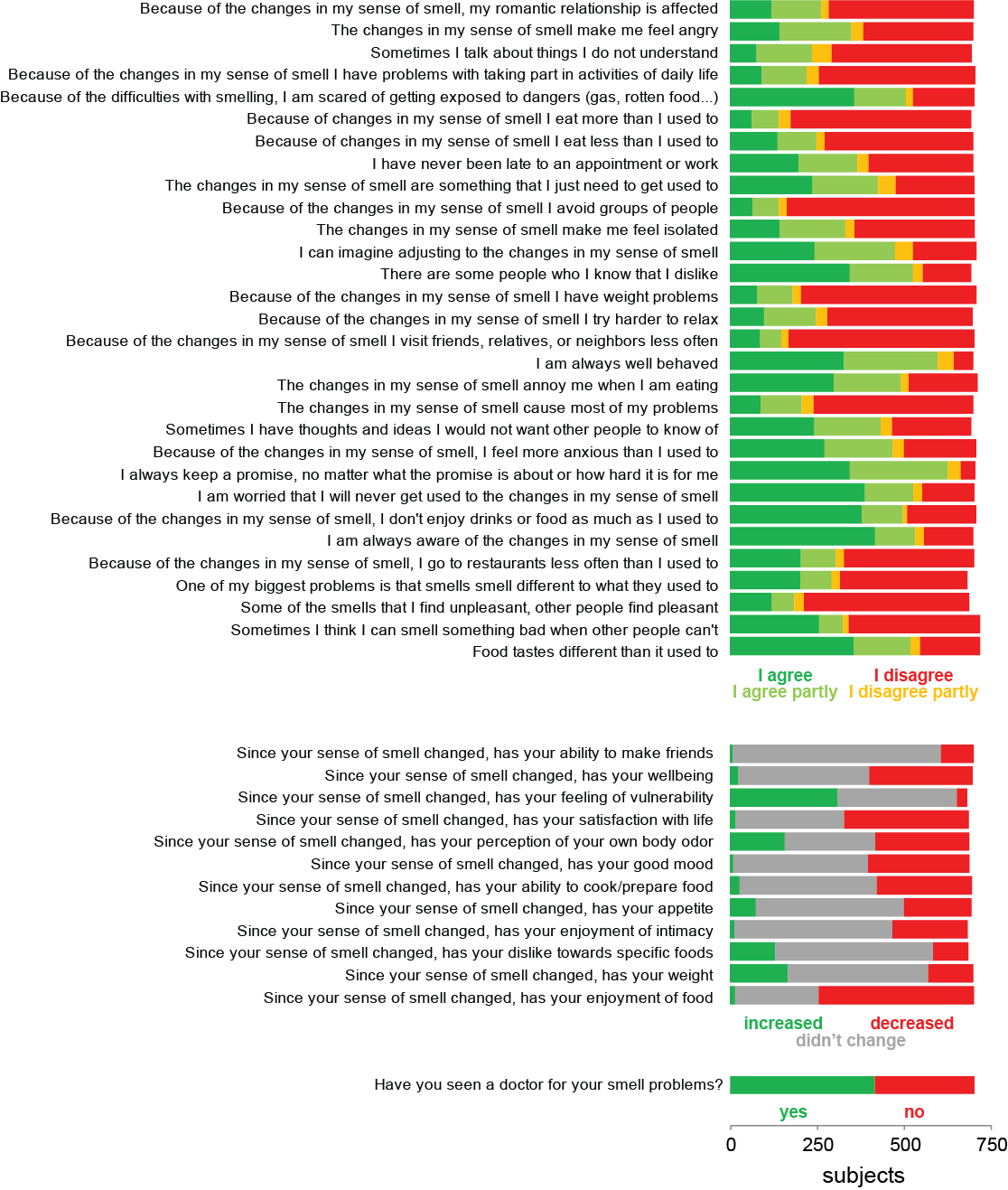

Supplement: Additional file 2 — Results of the questionnaire: The complete results of the questionnaire are shown. The 43 questions shown here include the 27 questions shown in Figure 3. [file 1472-6815-13-8-S2.png]
