# Supplementary material for: Hidden consequences of olfactory dysfunction: a patient report series
Source: BMC Ear Nose Throat Disord. 2013 Jul 23;13:8. doi: 10.1186/1472-6815-13-8 (PMC3733708; doi:10.1186/1472-6815-13-8)
Supplement: Additional file 3 — Methods: A more detailed description of the methods. [file 1472-6815-13-8-S3.docx]

# Methods

Between 10/16/2009 and 08/08/2012, subjects submitted their experiences with olfactory dysfunction online under the IRB-approved protocol NYU-SoM 09-0226. There was a Google Ad campaign that directed those searching for key words like "smell loss" or "anosmia" to the website of this study. One thousand subjects were selected for inclusion in this paper based on the relevance for the topic of this paper. Reports that had nothing to do with olfaction, were not in standard English, or consisted only of a few words were not included. The subjects' reports were then edited for language and clarity. In addition, for the sake of brevity, all information not central to the topic of this paper (for example about negative diagnostic tests that the patient underwent) has been removed. All potentially identifying information has also been removed.

## Questionnaire

In addition to submitting a free-form report, subjects were given a chance to complete a 43-point questionnaire that asked questions about specific aspects of life that are known or suspected to be affected by a change in olfactory acuity. The questionnaire was adapted from the one used by Frasnelli and Hummel [[108](#_ENREF_108)]. It included "negative" statements, which give information about how much patients suffer from their olfactory impairment, "positive" statements, which indicate how well they cope with the impairment, and "socially desired" statements to assess the degree to which the subjects give answers they believe they are expected to give.

725 of the 1,000 subjects included in this paper completed the questionnaire which included 30 statements, for which the subjects could indicate their agreement by choosing between four options: "I agree", "I agree partly", "I disagree partly", and "I disagree". The 20 statements were: "Food tastes different than it used to.", "Sometimes I think I can smell something bad when other people can't.", "Some of the smells that I find unpleasant, other people find pleasant.", "One of my biggest problems is that smells smell different to what they used to.", "Because of the changes in my sense of smell, I go to restaurants less often than I used to.", "I am always aware of the changes in my sense of smell.", "Because of the changes in my sense of smell, I don't enjoy drinks or food as much as I used to.", "I am worried that I will never get used to the changes in my sense of smell.", "I always keep a promise, no matter what the promise is about or how hard it is for me.", "Because of the changes in my sense of smell, I feel more anxious than I used to.", "Sometimes I have thoughts and ideas I would not want other people to know of.", "The changes in my sense of smell cause most of my problems.", "The changes in my sense of smell annoy me when I am eating.", "I am always well behaved.", "Because of the changes in my sense of smell I visit friends, relatives, or neighbors less often.", "Because of the changes in my sense of smell I try harder to relax.", "Because of the changes in my sense of smell I have weight problems.", "There are some people who I know that I dislike.", "I can imagine adjusting to the changes in my sense of smell.", "The changes in my sense of smell make me feel isolated.", "Because of the changes in my sense of smell I avoid groups of people.", "The changes in my sense of smell are something that I just need to get used to.", "I have never been late to an appointment or work.", "Because of changes in my sense of smell I eat less than I used to.", "Because of changes in my sense of smell I eat more than I used to.", "Because of the difficulties with smelling, I am scared of getting exposed to dangers (gas, rotten food...).", "Because of the changes in my sense of smell I have problems with taking part in activities of daily life.", "Sometimes I talk about things I do not understand.", "The changes in my sense of smell make me feel angry.", and "Because of the changes in my sense of smell, my romantic relationship is affected.".

In addition, there were 12 statements that had to be completed with "... increased", "...didn't change", or "... decreased". These statements were: "Since your sense of smell changed, has your enjoyment of food...", "Since your sense of smell changed, has your weight...", "Since your sense of smell changed, has your dislike towards specific foods...", "Since your sense of smell changed, has your enjoyment of intimacy...", "Since your sense of smell changed, has your appetite...", "Since your sense of smell changed, has your ability to cook/prepare food...", "Since your sense of smell changed, has your good mood...", "Since your sense of smell changed, has your perception of your own body odor...", "Since your sense of smell changed, has your satisfaction with life...", "Since your sense of smell changed, has your feeling of vulnerability...", "Since your sense of smell changed, has your wellbeing...", and "Since your sense of smell changed, has your ability to make friends...". Furthermore, subjects were asked "Have you seen a doctor for your smell problems?" (possible answers: "yes" and "no"). The complete results of the questionnaire are shown in Additional File 2.

## Subjects

All the participants in the study gave informed consent. Due to the fact that this was an anonymous online study, they consented by responding with "I agree" in response to the question " By submitting your story you give us the right to use your story for our research and to include it in research presentations and in publications." This procedure of obtaining informed consent has been approved by the New York University Medical School IRB IRB # 09-0226. The 1,000 subjects were from 64 different countries from around the world. Because the website was set up in English, most reports were from English-speaking countries (Figure 2a). Five hundred forty subjects were from the USA, 131 from the UK, 81 from India, 46 from Canada, and 38 from Australia. Sixty-two percent of the subjects who reported their gender were female and 38% male (Figure 2b). Almost three quarters self-identified as White or Caucasian (Figure 2c). The 1,000 subjects range in the age at which they submitted their report from 6 to 85 with a median of 52. The median age of onset of the problems was 46, with a range from 0 to 83 (Figure 2d). Fifty-nine percent of the subjects had previously seen a doctor for their olfactory dysfunction (Additional File 2). A third to half of the subjects reported experiencing smell distortions in addition to smell loss (Figure 2e).
